# Did α-Synuclein and Glucocerebrosidase Coevolve? Implications for Parkinson’s Disease

**DOI:** 10.1371/journal.pone.0133863

**Published:** 2015-07-27

**Authors:** James M. Gruschus

**Affiliations:** Laboratory of Structural Biophysics, NHLBI, NIH, Bethesda, Maryland, United States of America; Hertie Institute for Clinical Brain Research and German Center for Neurodegenerative Diseases, GERMANY

## Abstract

Mutations in the *GBA1* gene are associated with increased risk of Parkinson's disease, and the protein produced by the gene, glucocerebrosidase, interacts with α-synuclein, the protein at the center of the disease etiology. One possibility is that the mutations disrupt a beneficial interaction between the proteins, and a beneficial interaction would imply that the proteins have coevolved. To explore this possibility, a correlated mutation analysis has been performed for all 72 vertebrate species where complete sequences of α-synuclein and glucocerebrosidase are known. The most highly correlated pair of residue variations is α-synuclein A53T and glucocerebrosidase G115E. Intriguingly, the A53T mutation is a Parkinson's disease risk factor in humans, suggesting the pathology associated with this mutation and interaction with glucocerebrosidase might be connected. Correlations with β-synuclein are also evaluated. To assess the impact of lowered species number on accuracy, intra and inter-chain correlations are also calculated for hemoglobin, using mutual information Z-value and direct coupling analyses.

## Introduction

The synucleins are small (≤ 144 residues), membrane interacting proteins found predominantly in neurons, especially in their presynaptic regions, though small amounts have also been detected in non-neural tissue.[1] They are intrinsically disordered in solution and can adopt helical structure when bound to membranes. The most studied member of the synuclein family is α-synuclein (α-syn), due to its connection with Parkinson’s disease (PD).[2–4] In PD α-syn forms amyloid, polymerizing into long fibrils via beta strand interactions between the monomers. These fibrils are the major component of Lewy bodies and neurites, proteinaceous inclusions seen in the neurons of Parkinson’s disease patients.[5] As for its normal biological function, α-syn appears to be involved in the maintenance, exocytosis and regeneration of presynaptic vesicles,[6–10] interacting with the v-SNARE protein synaptobrevin-2 (also known as VAMP2).[11,12] Other potential interaction partners include Rab3a and Rab8b,[13,14] calmodulin,[15] dopamine transporter,[16] parkin, [17] phospholipase D1,[18] synphilin-1 (SNCAIP),[19] and tau.[20] In addition, α-syn interacts with the lysosomal enzyme glucocerebrosidase (GCase),[21] described below.

**Fig 1:**
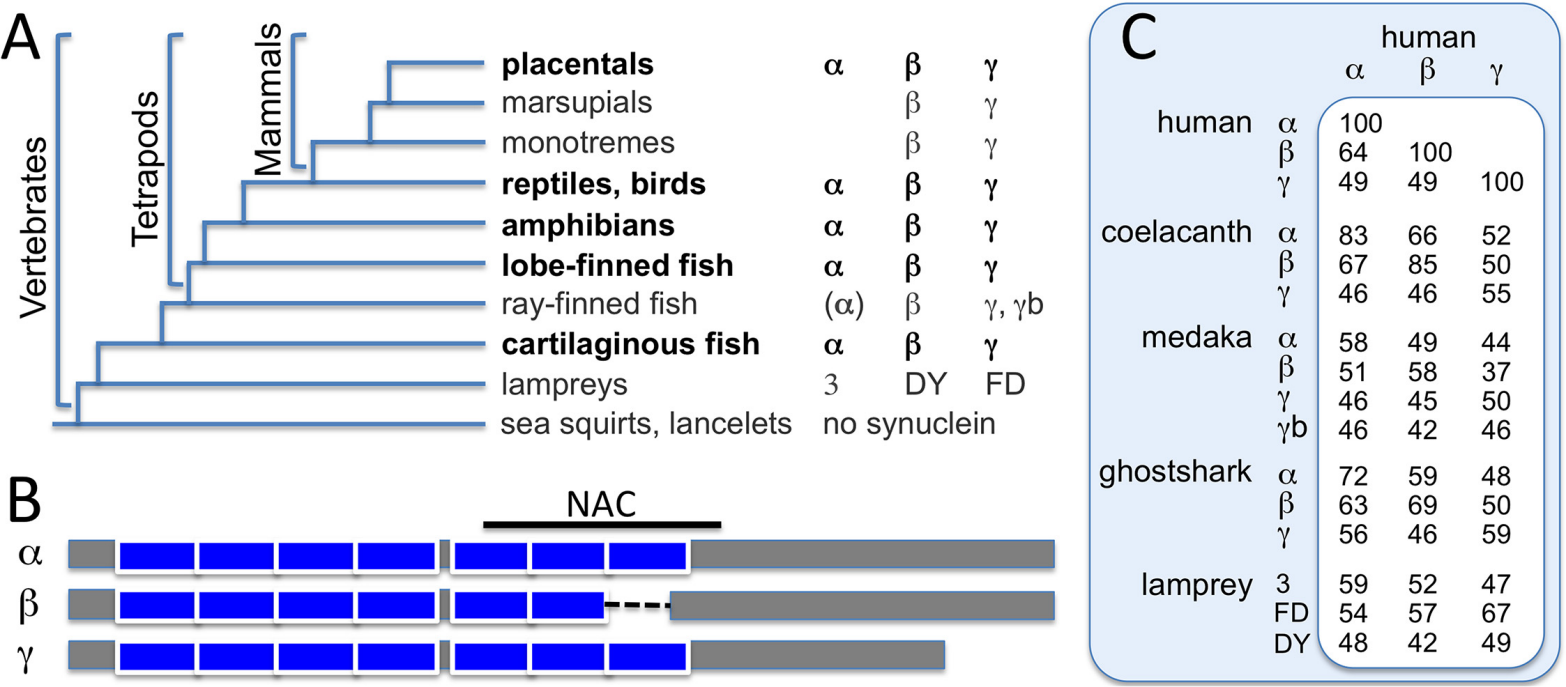
Synuclein evolutionary tree and sequence features. A) The types of synuclein proteins found in different branches of vertebrates are shown. Some ray-finned fish have a fourth synuclein that resembles α-syn in the NAC region. B) The sequence features of α-syn, β-syn and γ-syn are diagrammed. The boxes represent the imperfect amphipathic repeats, and the dashed line for β-syn indicates the gap in the NAC region, which is indicated by the line above α-syn. C) Table of sequence identities (%) comparing synucleins in tetrapods (human and coelacanth), ray-finned fish (medaka), cartilaginous fish (ghostshark) and jawless fish (lamprey).

The synuclein family of proteins arose after the common ancestor of vertebrates diverged from other chordates. Typically, three genes for synuclein are present in each species. For example, humans have α, β and **γ**lampreys have three synucleins designated 3, DY and FD, and zebra fish have β, and two **γ**-synucleins, though many ray-finned fish also have a fourth synuclein with some αfeatures.[22] Fig 1A shows a simplified evolutionary tree with the types of synuclein found in vertebrates. Most have α, β and **γ** types of synuclein. The exceptions are the jawless and ray-finned fishes, and curiously, monotreme and marsupial mammals, which appear to lack α-syn. Monotreme and marsupial genomes have not yet received the full extent of study that other genomes have, such as the zebra fish and human genomes. It might be that an α-syn gene does exist for non-placental mammals, but that it has somehow eluded discovery to date.

All synucleins bind membrane via their N-terminal regions, which include six (β) or seven (α and **γ**) imperfect amphipathic helix repeats (XKTKEGVXXXX) (Fig 1B). The α and β forms of synuclein share more sequence identity, being 64% identical in humans, while they both differ more from the **γ** form, with 49% identity between α or β and **γ**(Fig 1C). An important difference between the α and β forms is that α-syn has an additional 11 residue region that forms part of what is known as the non-amyloid-β component (NAC) region. The NAC region, residues 61–95, which was first detected as a component in amyloid beta plaques,[1] is part of the core beta sheet region in human α-syn amyloid fibrils.[23,24] In contrast, β-syn typically does not form fibrils and can even inhibit α-syn fibril formation.[25]

Mutations in several genes have been linked to increased risk for PD.[26] One of these genes is *GBA1*, the gene encoding glucocerebrosidase (GCase), a 60 kDa enzyme that cleaves glucose from glucosyl-sphingolipids such as glucosylceramide. Inheritance of mutant *GBA1* from both parents results in Gaucher disease, the most common of the lysosomal storage diseases.[27] Carriers of *GBA1* mutations are found fivefold more frequently among PD patients than in non-PD controls,[28] and GCase has become an important therapeutic target for PD, as lowered activity has been linked to elevated α-syn levels.[29,30] The GCase enzyme is more ancient and widely expressed than α-syn. GCase is active in lysosomes, organelles found in nearly all types of animal cells, where proteins, nucleic acids, lipids and carbohydrates are broken down for reuse in the cell. The connection between GCase and α-syn might seem surprising, given that the substrate of GCase is a glycolipid, and that lysosomes and the presynaptic regions of neurons are distinct cellular milieus. Evidence suggests, however, that lysosomal degradation of α-syn plays an important role in maintaining normal α-syn levels in cells.[31] Intriguingly, α-syn interacts with wild type GCase under the acidic conditions found in lysosomes, and interaction is reduced for a common mutant form of GCase.[21] These observations hint that α-syn/GCase interaction might play some biological role that mutation of GCase disrupts.

In this study we explore the possibility that α-syn and GCase might have coevolved. That is, if during vertebrate evolution the interaction of α-syn and GCase acquired an important biological role, then subsequent mutations disrupting this interaction will have been selected against. Such selection should lead to two consequences. First, residues at the interaction interface should be more conserved. Second, if a mutation does occur at the interface, there is a chance that a corresponding mutation occurs in the interacting partner, one that either maintains the interaction or otherwise enhances fitness via a modified interaction. Of course, the first mutation must not affect fitness so much that it is selected out before the second mutation occurs. This means that when such correlated mutations do occur, their effect on interaction could be subtle when compared to other residue-residue interactions at the interface.

Several methods for identification of correlated mutations have been developed, reviewed in [32] with most used for *de novo* structure prediction,[33,34] though methods developed for inter-protein interaction are increasing in number.[35–38] The vetting process for these methods has often employed proteins for which 1000+ homolog sequences are known, and the importance of having very large numbers of sequences has been demonstrated repeatedly, with the minimum number of sequences for robust predictions estimated at 150 or higher, depending on protein length and the analysis employed.[32,39] However, this study analyzes the vertebrate specific protein α-syn, and only ~100 vertebrate genomes have been sequenced to date. To gain perspective on how effective these analyses are at identifying correlated mutations for vertebrate proteins and how the limitation imposed by lower number of sequences impacts the prediction, correlations between the alpha and beta chains of hemoglobin are also analyzed.[40] Hemoglobin is especially well suited for this analysis since its tetrameric structure has been confirmed by x-ray crystallography for 42 vertebrate species. Two of the most successful correlated mutation analyses are employed, direct coupling analysis (DCA) and mutual information (MI) Z-values,[41–44] and based on the results with hemoglobin, the MI Z-value approach has been chosen to identify putative correlated mutations in the 72 species where complete sequences of both α-syn and GCase are known.

## Methods

### Direct Coupling Analysis (DCA)

Direct coupling analysis was formulated to correct an issue encountered in other correlation analyses, namely, often ‘indirect’ correlations could obscure ‘direct’ correlations.[37] That is, in situations where A interacts with B and B interacts with C, sometimes the [A,C] correlation coefficient could be larger than the [A,B] and [B,C] coefficients, due to extraneous factors such as differing residue mutation rates. To avoid this DCA takes all the single site and pairwise joint amino acid frequencies in the multiple sequence alignments (MSAs) of the proteins, combines them in a covariance matrix, and using a maximum entropy approach, corrects the joint frequencies in order to reduce the correlations of indirectly interacting pairs. Details of the derivation can be found in [41]. The correlation coefficients or “direct information” (DI) scores are given by the following mutual information formula:

$${DI}_{ij}=\sum_{m,n}P_{ij}\left(m,n\right)\text{ln}[\frac{P_{ij}\left(m,n\right)}{f_{i}\left(m\right)f_{j}\left(n\right)}]$$

where *DI*_*ij*_ is the DI score between residues positions *i* and *j* in the MSA, *P*_*ij*_ *(m*,*n)* is the corrected joint frequency of amino acid *m* in position *i* and amino acid *n* in position *j*, and *f*_*i*_ *(m)* and *f*_*j*_ *(n)* are the amino acid frequencies at position *i* of amino acid *m* and position *j* of amino acid *n*, respectively, and the sum is taken over all amino acid types including gaps.

For the hemoglobin test case, the set of pairs considered to be in contact included all residue pairs with closest atom distance ≤ 7 Å in either oxy or deoxy forms. All sequences in this study were aligned using Clustal Omega.[45] All sequences were identified from blastp searches[46], repeated several times using the most divergent sequences in each set as query sequences, with incomplete sequences and any with unidentified residues eliminated from the set. For species with multiple entries for the same protein, the sequence with the most entries was chosen. The hemoglobin, α-syn, **β**-syn and GCase MSAs are provided in the Supporting Information (S1 Text). The DI values for hemoglobin were calculated with the MATLAB (The MathWorks, Inc.) script for DCA downloaded from dca.rice.edu.[41] For the intra-alpha chain DI calculation, only the alpha chain sequences were used, while for the inter-chain alpha-beta DI calculation, the input consisted of concatenated alpha and beta chain sequences, following protocol,[37] and the subset of DI values corresponding to inter-chain residue pairs used to assess the accuracy of predicted inter-chain contacts.

The DCA theta (*Ɵ*) and pseudocount_weight variables were optimized for both the intra and inter-chain calculations using the 314 species hemoglobin MSA. Varying the pseudocount_weight had little impact on accuracy, and the default value of 0.5 was used. Reducing theta improved both the intra and inter-chain prediction accuracies. The theta variable determines the sequence identity threshold in reweighting highly homologous sequences in the MSA.[41] For the intra-chain calculation, reducing theta from the default value of 0.2 to 0 resulted in the top-ranked correct contact prediction going from second at *Ɵ* = 0.2 and 0.1 to first at *Ɵ* = 0. The number of correctly predicted intra-chain contacts increased from 1 in the top ten and 20 in the top 100 ranked DI values for *Ɵ* = 0.2, to 4 and 22 for *Ɵ* = 0.1, to 6 and 24 at *Ɵ* = 0. For the inter-chain calculation, the top-ranked correct contact prediction went from 24^th^ at *Ɵ* = 0.2 to second at *Ɵ* = 0.1 and 0. The number of correctly predicted inter-chain contacts went from 0 in the top ten and 2 in the top 100 ranked DI values for *Ɵ* = 0.2, to 1 and 1 for *Ɵ* = 0.1, and to 1 and 3 for *Ɵ* = 0. Because *Ɵ* = 0 produced the most accurate predictions for both intra and inter chain contacts, this value was used to calculate the DI values in this study. A value of zero for theta means all hemoglobin sequences are weighted equally in the DI calculation.

### Mutual Information (MI) Z-values

The expression for mutual information used in the MI Z-value analysis is the same as that used for the DI values in DCA, except the joint frequencies are not corrected for indirect correlations. The expression contains a correlation term *f*_*ij*_ *(m*,*n)*/*f*_*i*_ *(m)f*_*j*_ *(n)* within an entropy-like expression *f*ln[*f*]. The entropy-like aspect of MI results in larger values when more pairs of correlated mutations occur, reflecting the likelihood that the correlation is not just due to chance.[39,43]

$${MI}_{ij}=\sum_{m,n}{f\prime }_{ij}\left(m,n\right)\text{ln}[\frac{{f\prime }_{ij}\left(m,n\right)}{{f\prime }_{i}\left(m\right){f\prime }_{j}\left(n\right)}]$$

where *f’*_*ij*_ *(m*,*n)* is the joint frequency that amino acid *m* occurs at position *i* and amino acid *n* occurs at position *j* in the same species, and *f’*_*i*_ *(m)* and *f’*_*j*_ *(n)* are the individual frequencies of amino acid *m* at position *i* and amino acid *n* at position *j*, and the sum is taken over all pairs of amino acid types including gaps. The frequencies include a pseudocount correction

$${f\prime }_{i}\left(m\right)=\left(\frac{s}{s+21\lambda }\right)\;\left(\frac{\lambda }{s}+f_{i}\left(m\right)\right)$$

$${f\prime }_{j}\left(n\right)=\left(\frac{s}{s+21\lambda }\right)\;\left(\frac{\lambda }{s}+f_{j}\left(n\right)\right)$$

$${f\prime }_{ij}\left(m,n\right)\;=\;\left(\frac{s}{s+42\lambda }\right)\;\left(\frac{\lambda }{s}{f\prime }_{i}\left(m\right)+\frac{\lambda }{s}{f\prime }_{j}\left(n\right)+\;f_{ij}\left(m,n\right)\right)$$

where *λ* is the pseudocount, *s* is the number of species in the sequence alignment, and *f*_*i*_ *(m)* and *f*_*j*_ *(n)* are the actual frequencies of amino acid *m* at position *i* in the first protein sequence and amino acid *n* at position *j* in the second protein sequence. That is, *f*_*i*_ *(m)* equals the number of times amino acid *m* occurs at position *i* divided by the number of species, and similar for *f*_*j*_ *(n)*. Likewise, *f*_*ij*_ *(m*,*n)* is the number of times the amino acid pair (*m*,*n*) occurs divided by the number of species. For the pseudocount, *λ* = 1.5 is used. A discussion of the importance of the pseudocount variable, especially in cases where sequencing errors might be present, is given in Appendix I in the Supporting Information (S2 Text). DCA also uses a pseudocount correction, but its implementation is more complex.[41]

MI Z-values, or Z-scores, tell one how many standard deviations the MI value for a particular pair of positions differ from the average MI for those positions with all other residue positions in the protein, and the Z-values are calculated as

$$Z_{ij}\;=\;0.5\left[\left({MI}_{ij}-\;{MI}_{iav}\right)/\sigma_{i}+\left({MI}_{ij}-\;{MI}_{jav}\right)/\sigma_{j}\right]$$

where *MI*_*iav*_ is the average MI value between position *i* and all positions in the second protein sequence, *MI*_*jav*_ is the average MI value between position *j* and all positions in the first protein sequence, and *σ*_*i*_ and *σ*_*j*_ are the corresponding standard deviations.[42,43].

## Results

### Hemoglobin test case: intra *versus* inter-chain and the effect of reducing the number of sequences

**Fig 2:**
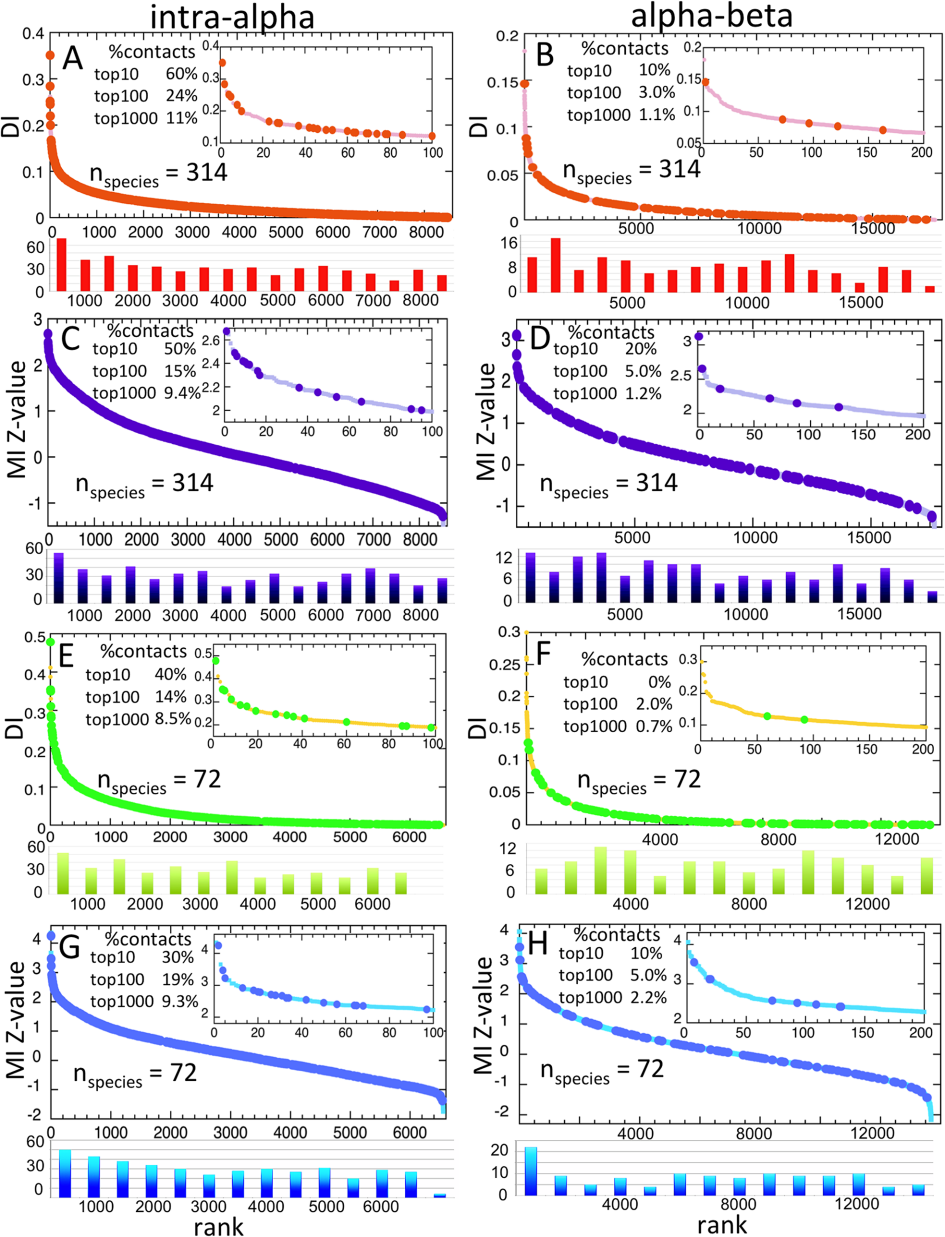
DCA and MI Z-value correlated mutation analyses of hemoglobin. A) Intra-chain and B) inter-chain DI values (y-axis) for the 314 species hemoglobin MSA, ranked largest to smallest on the x-axis, between all non-invariant residues (light red dots), with those corresponding to contacts shown as orange circles. The inset shows an expanded view of the top 100 (intra-chain) or top 200 (inter-chain) results. The percentages of correctly predicted contacts for the top 10, 100, and 1000 results are shown. Because of the large number of contacts, histograms are shown below, with the number of contacts binned every 500 DI values (intra-chain) or 1000 DI values (inter-chain). C) Intra-chain and D) inter-chain MI Z-values for the 314 species hemoglobin MSA, shown similarly to A) and B). E) Intra-chain and F) inter-chain DI values for the 72 species hemoglobin MSA, corresponding most closely with species for which complete α-syn and GCase sequences are known, shown similarly to A) and B). G) Intra-chain and H) inter-chain MI Z-values for the 72 species hemoglobin MSA, shown similarly to A) and B). Note that for the 72 species hemoglobin MSA, there are fewer non-invariant residues, and thus fewer potential intra- and inter-chain pairs along the x-axis.

Both DCA and MI Z-value analyses performed quite well at predicting intra-alpha chain contacts for the 314 species hemoglobin multiple sequence alignment (MSA) (Fig 2A and 2C). The DCA analysis performed best, with six of its top ten ranked DI values corresponding to contacting residues. Details on the optimization of the DCA parameters are given in the Methods. Both analyses gave the same number one ranked pair, the contacting residues 30 and 50. The situation is completely reversed for the inter alpha-beta chain contact prediction (Fig 2B and 2D), with both analyses performing more poorly, and with the MI Z-value analysis outperforming DCA, with two of the top ten Z-values corresponding to contacting residues. In this case, the number one Z-value ranked pair, corresponding to contacting residues 111 and 115 in the alpha and beta chains, differed from the top correct DI pair, ranked at number two, corresponding to residues 120 and 33. Mathematically, however, one expects the inter-chain case to be more challenging; hemoglobin has 537 intra-chain *versus* 149 inter-chain residue contacts but 8515 intra-chain *versus* 17685 inter-chain potential residue pairs. Viewed in this light, the correct prediction by the MI Z-value analysis of 20% inter-chain contacts is more encouraging. In fact, the 20% correct prediction is comparable to that obtained by intra-protein methods when one restricts the set of residue pairs to include only long-range contacts (> 24 residues separation in sequence).[47]

To test the effect of reducing the number of sequences, the DCA and MI Z-value analyses were repeated using a subset of 72 of the 314 hemoglobin proteins corresponding most closely with the 72 species for which complete α-syn and GCase sequences are known (Fig 2E and 2H). For the intra-chain case, both analyses performed more poorly than with the 314 species MSA, but still performed moderately well, with DCA again outperforming the MI Z-value analysis with 4 of the top ten DI values corresponding to contacts. Interestingly, the number of correct predictions in the top 100 Z-values actually increased, however. Both analyses also performed more poorly for the inter-chain calculation, especially DCA. The MI Z-value analysis still gave one correctly predicted pair in the top ten, and the same number in the top 100 as for the 314 species case. The spread of DI and Z-values increased significantly for the 72 species MSA, due to the larger percentage of invariant residues and the greater effect of statistical fluctuations in the smaller data set. Notably for the smaller species set, the DI top correctly predicted inter-chain pair is the same as the Z-value top pair, alpha 111 with beta 115. The possibility that this could happen by chance is very low. The results show that while it is still possible to obtain correctly predicted correlated residue pairs with fewer sequences, increased “noise” can potentially reduce the number of highest-ranked, correctly predicted pairs. Given its better performance in predicting inter-chain hemoglobin contacts, the MI Z-value analysis has been chosen as the main method for the correlated mutation analysis of α-syn and GCase. Still, one should not necessarily generalize, and the implications of the intra and inter-chain results are addressed further in the Discussion.

### Correlated mutation analysis of α-syn and GCase

**Fig 3:**
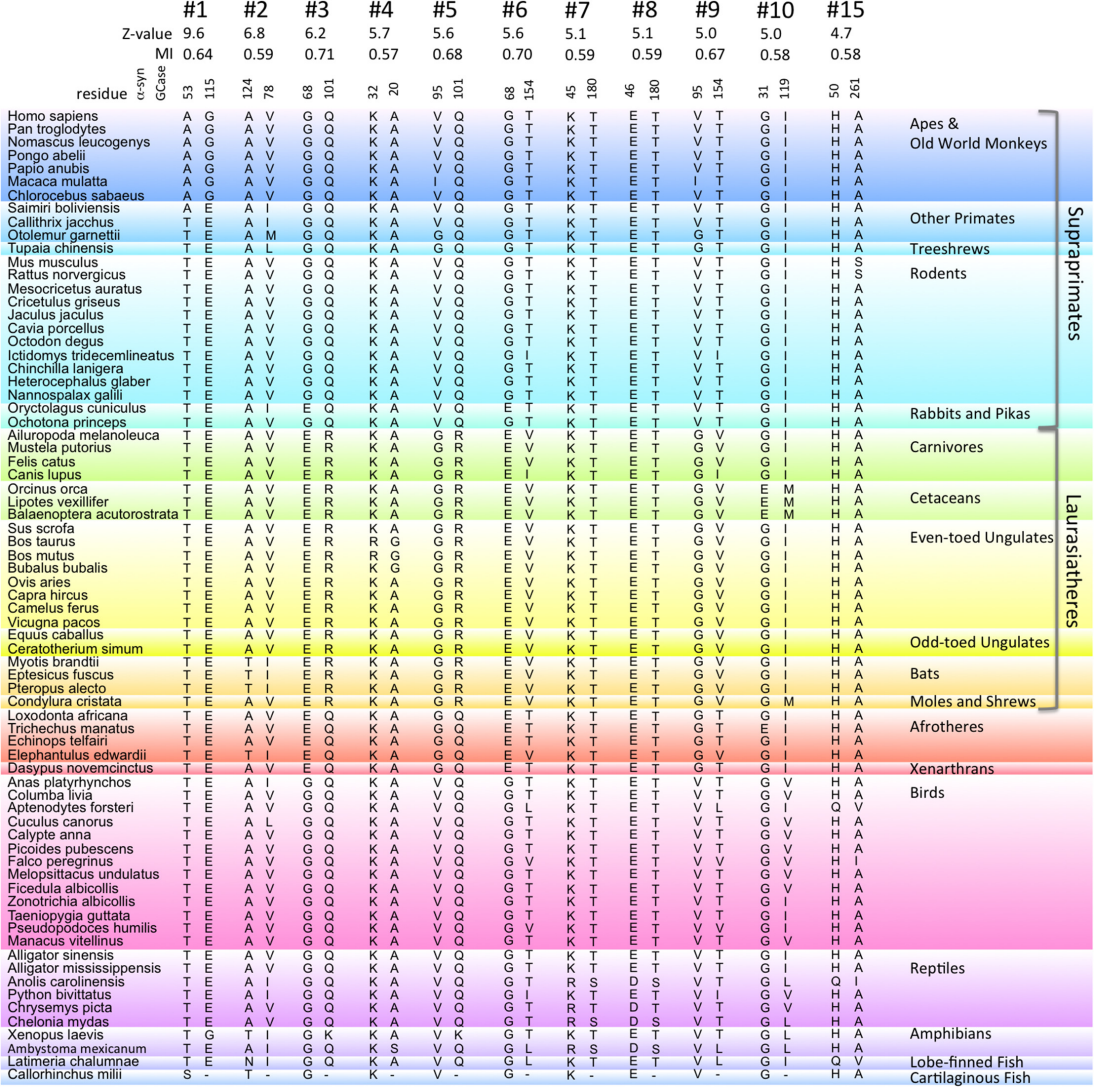
Top ten MI Z-value ranked α-syn, GCase correlated residue pairs The pairs of α-syn and GCase residues for 72 vertebrate species with the most highly correlated mutations, as determined by the Z-value analysis, are displayed, with the Z-values and MI values shown. Also shown is the #15 ranked correlated pair, since it, along with the #1 and #8 ranked pairs, has an α-syn residue corresponding to a PD-associated mutation. The species are organized on the class, sub-class, or infra-class levels to highlight phylogenetic aspects of the residue variations. The α-syn residues for the cartilaginous fish, C. milii, are also shown, though since its complete GCase sequence is not known, C. milii was not included in the analysis.

An MI Z-value correlated mutation analysis was performed using sequences from 72 vertebrate species where complete sequences of both α-syn and GCase have been reported. The α-syn-like sequences found in some ray-finned fish were not included; these sequences lack the conserved C-terminal region known to interact with GCase.[21] Fig 3 shows the top ten MI Z-value ranked correlated pairs. The choice of ten is somewhat arbitrary, and views differ on the number of top-ranked predicted pairs that should be considered as candidates for correlated residues.[39,48] The analysis of hemoglobin shows the odds of actual contacts beyond the top 10 ranked pairs falls rapidly, from 50% for the top ten intra-alpha pairs dropping to 15% for the top 100, and to 9% for the top 1000 (Fig 2C). The trend is even more pronounced for the alpha-beta inter-chain results, with 20% for the top ten, 5% for the top 100, and 1.2% for the top 1000 (Fig 2D).

The top ten MI Z-values for α-syn and GCase (5.0–9.6) are much higher than those obtained for the alpha and beta hemoglobin correlated mutation analysis of the 72 species subset (3.4–4.1). However, this does not mean the α-syn, GCase correlations are “stronger” than the hemoglobin ones. The higher Z-values are a consequence of the greater sequence conservation of α-syn and GCase compared to hemoglobin. Human and coelacanth α-syn sequences are 81% identical, 63% identical for GCase, but only 51% identical for hemoglobin. MI Z-values show how many standard deviations MI values differ from average, and the more conserved the set of sequences is, the more that correlated residue pairs can potentially differ from the baseline average. The actual MI values are more similar, 0.57–0.71 versus 0.58–0.67, for the top ten α-syn/GCase and alpha/beta chain Z-value ranked pairs, respectively. Correlations were also calculated using the DCA method. Consistent with the hemoglobin results, several top ten Z-value α-syn, GCase pairs are amongst the top 100, but not top 10 DCA pairs. To be precise, Z-value ranked pairs #1, #2, #3, #5, #6, and #9 correspond to DCA ranked pairs #43, #46, #20, #32, #51, and #81, respectively.

#### #1 [α-Syn 53, GCase 115]

The top MI Z-value ranked pair is α-syn residue 53 with GCase residue 115. This pair is intriguing because the A53T and A53E mutations in humans are associated with PD.[49,50] Even more curious is the fact that except for apes, Old World monkeys and one New World monkey (squirrel monkey, S. boliviensis), nearly all other species have threonine at position 53, the same amino acid as the PD-associated mutation A53T. Only the Australian ghost shark C. milii has a different amino acid, serine. Unfortunately, GCase residue 115 of C. milii is unknown; its sequence is known for only residues 231–497. The Z-value of 9.6 for this pair is quite high, much higher than the highest values seen for hemoglobin. The value is high because this pattern of residue variation, seen just for apes and Old World monkeys, is not seen for any other residues in α-syn or GCase. Intra-protein Z-value and DCA correlations for α-syn and GCase were also examined, confirming that the pattern is unique.

To test whether the [α-syn 53, GCase 115] Z-value is influenced by over-representation of Old World monkey and ape sequences, the calculation was redone with the closest human relatives removed one by one. Removing chimpanzee (P. troglodytes), orangutan (P. abelii) and gibbon (N. leucogenys) sequences had a negligible effect on the Z-value. Only after also removing baboon (P. anubis) and rhesus macaque (M. mulatta), leaving just human and green monkey (C. sabaeus), did the Z-value fall to number two in the rankings. Thus, the high Z-value does not appear to be significantly inflated by over-representation of Old World monkey and ape sequences.

**Fig 4:**
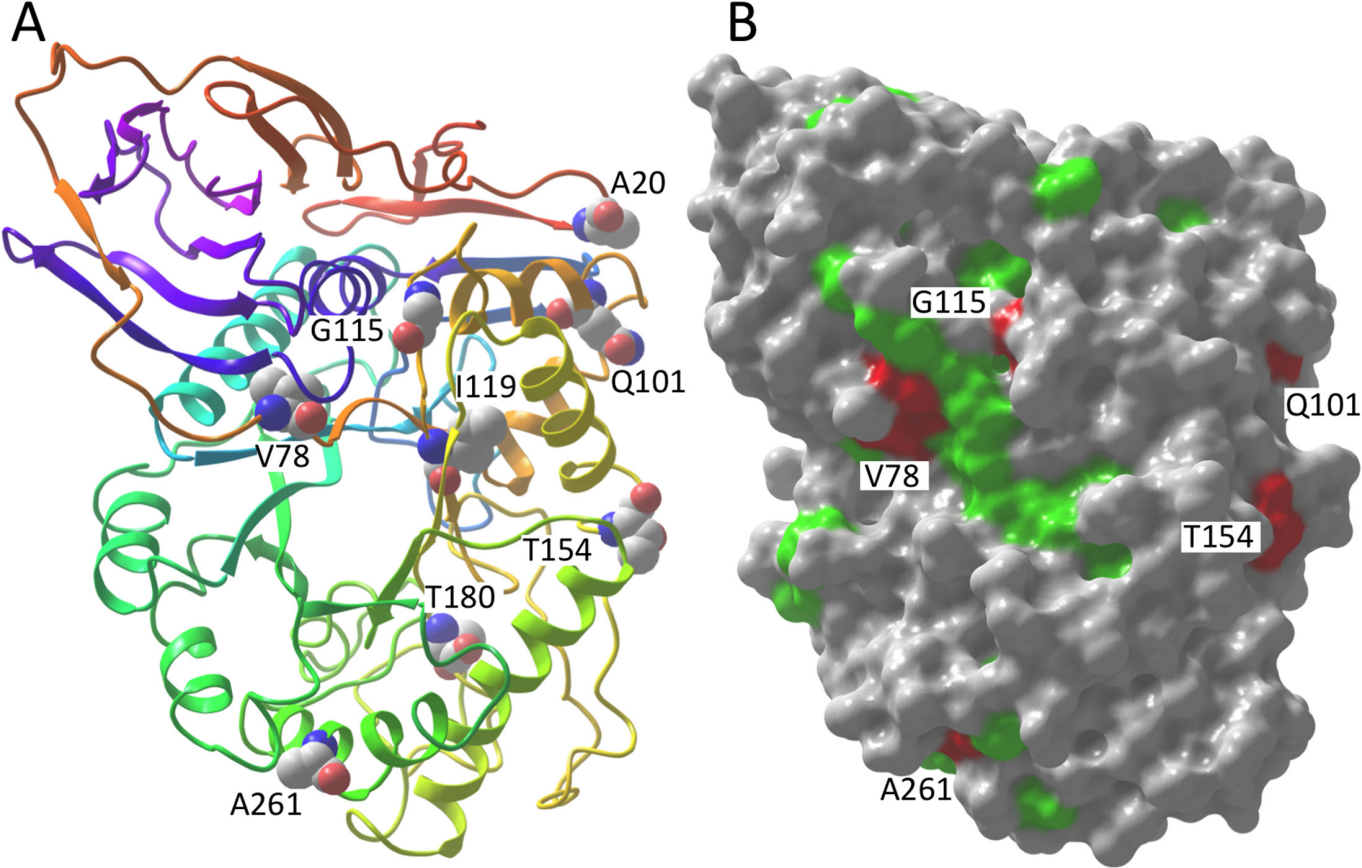
GCase residues for the top ranked correlated pairs and conserved surface regions A) A ribbon structure of human GCase (pdb 1OGS) with the residues from the top correlated pairs shown. B) The surface corresponding to invariant GCase residues is shown in green, with the surfaces of selected correlated pair residues in red and labeled. A large patch of invariant surface lies between V78 and G115.

Fig 4A shows the locations of the top ten ranked GCase residues in the crystal structure of human GCase (1OGS).[51] Interestingly, G115 is not exposed in the structure, though it is near the surface, and its alpha carbon is situated such that a glutamate substituted at this position would almost certainly be surface-exposed. Since G115 is buried for human GCase, this residue cannot directly contact α-syn residue A53 in humans unless there is a change in GCase structure upon interaction. However, while residue contacts are used as a proxy to test how well different analyses identify correlated mutation pairs, it is not necessarily the case that all truly correlated pairs are in direct contact. For instance, residues could be in contact in some species with a particular pair of amino acids and not in others with a different pair. Alternatively, a correlated pair might not be in contact in any species, but instead allosterically influence protein interaction.[52,53] Thus, many scenarios leading to the [α-syn 53, GCase 115] correlated pair are possible.

It is also possible that the [α-syn 53, GCase 115] pair do not interact, neither directly nor allosterically, and that the correlation arose solely by chance. Since only one correlated mutation is seen for this pair, the odds that the pair arose by chance are not insignificant. An estimation of the probability in a simplified test case is given in Appendix II of the Supporting Information (S3 Text), yielding roughly a 1 in 3 chance that such a correlation might have arisen between non-interacting residues. This sort of chance correlation is an aspect of what is termed phylogenetic noise or bias,[54,55] and since it may be relevant to the putative [α-syn 53, GCase 115] correlated pair, a bit more explanation is warranted here.

In general, the MI Z-value approach removes much of the influence of phylogenetic bias. In particular, the more common a pattern of sequence variation, due to shared ancestry for instance, the lower the corresponding Z-values for residue pairs displaying the common pattern.[56] For example, differences in the α-syn sequences between mammals and non-mammals are fairly common, likewise for GCase, so residue pairs displaying a mammal/non-mammal sequence difference pattern score lower Z-values. On the other hand, if the pattern is rare, as is the case with [α-syn 53, GCase 115], the Z-value will be high. Therefore, additional criteria are needed to identify which potential correlated pairs are less likely to have arisen by chance.

One common criterion to distinguish pairs that are less likely to be due to chance is that the correlated mutation pair occurs in at least two non-sister groups, that is, the mutation pair must have arisen at least twice independently.[57,58] The [α-syn 53, GCase 115] pair fails this requirement since the correlated pair of mutations occurs only in the Old World monkeys and apes clade. More sequences from cartilaginous fish could help to make a stronger case for correlation, since the one currently known, that of the C. milii, is the only α-syn sequence with neither alanine nor threonine in position 53. As for GCase 115, the African clawed frog (X. laevis) has the GCase E115G substitution, like apes and Old World monkeys, but it retains α-syn T53, and thus does not provide additional support for a true correlation. Therefore, despite α-syn 53 and GCase 115 being the top-ranked correlated pair, it cannot be ruled out that the correlation might have arisen by chance.

#### #2 [α-Syn 124, GCase 78]

**Fig 5:**
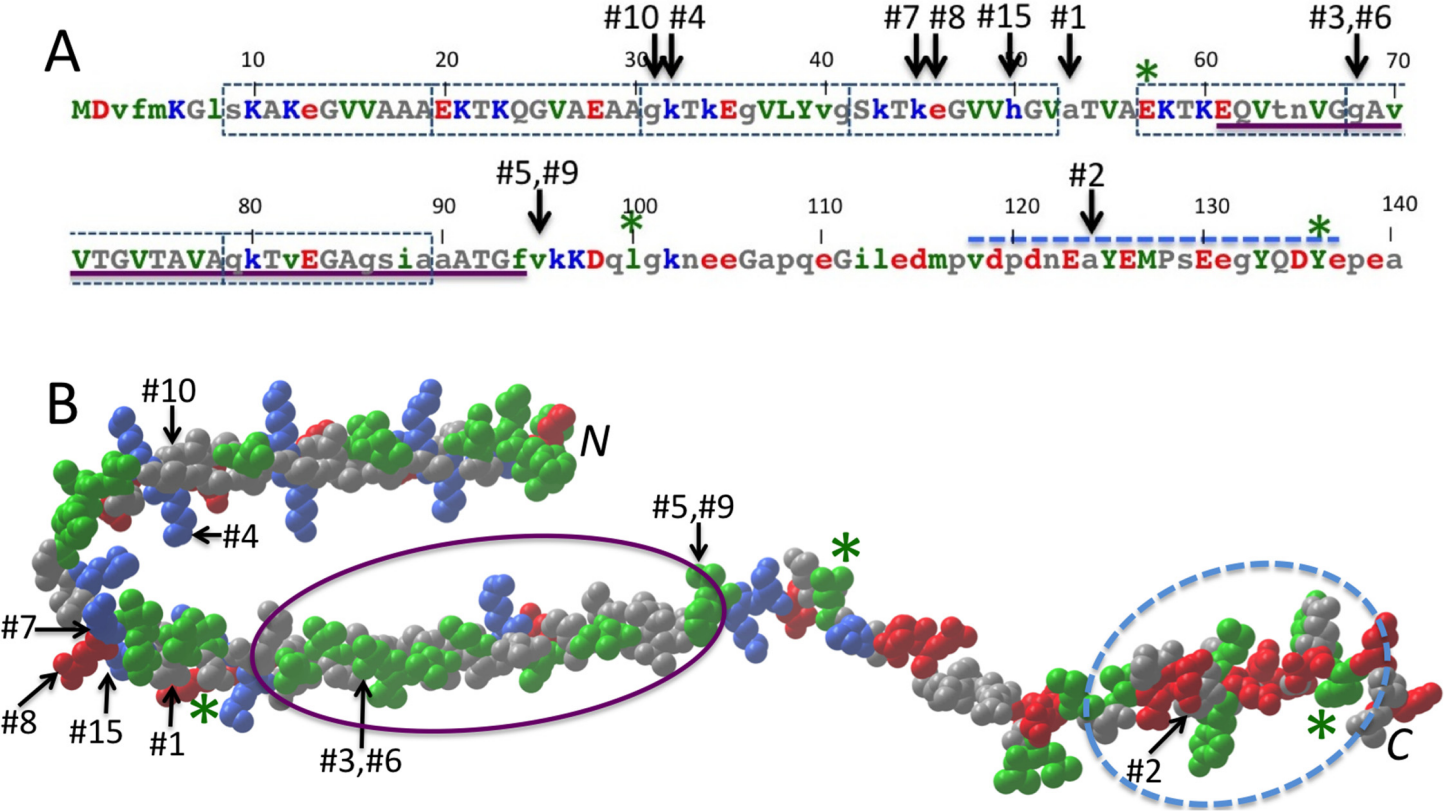
Sequence and structure of α-syn showing top correlated residues and regions known to interact with GCase A) The α-syn residues of the top ranked correlated pairs with GCase are indicated. Invariant residues are capitalized, negatively charged residues in red, positively charged in blue, hydrophobic in green, all others gray. The seven imperfect repeat regions are indicated by the dashed boxes and the NAC region is underlined (purple). In solution only residues 118–137 (dashed blue line) interact with GCase. In the presence of lipid vesicles, fluorescent labels at residues 57, 100, and 136 (green asterisks) showed interaction with GCase, indicating a much larger region of interaction than in solution. B) The α-syn structure with residues of the top ranked correlated pairs indicated. The residue coloring is the same as for the α-syn sequence. The NAC region is indicated by the purple oval, and the C-terminal region that interacts with GCase in solution is indicated by the dashed blue oval. The green asterisks indicate the locations at residues 57, 100, and 136 where fluorescent labels showed interaction with GCase in the presence of lipid vesicles. The structure shown is micelle bound α-syn (pdb 1XQ8).

The second highest MI Z-value ranked pair is α-syn 124 with GCase 78. At first glance, this potential correlated pair is not very compelling. Specifically, the mutations for GCase 78 are all conservative substitutions, valine, isoleucine, leucine and methionine. Furthermore, the side chain of residue 78 is buried in the GCase structure, with only the backbone exposed (Fig 4). On the other hand, this residue precedes K79, a surface-exposed, invariant residue, and mutations of V78 and K79 are associated with Gaucher disease.[27,59] Even though correlated mutation analyses cannot directly identify contacts involving invariant residues, correlations involving neighboring non-invariant residues could still allow one to deduce their presence. Note that α-syn 124 lies between two invariant glutamates, E123 and E126 (Fig 5), which could potentially form a salt-bridge with GCase K79.

The α-syn A124T substitution is only present when GCase 78 is isoleucine. There are more occurrences of isoleucine for GCase 78 than threonine for α-syn 124, suggesting that the α-syn A124T substitution could be accommodated only after GCase I78 was already present. Valine is the most common residue for GCase 78, so perhaps the size difference between isoleucine and valine at residue 78 perturbs the positioning of K79 in a way that alters its interactions with α-syn residue 124 and its neighboring glutamates. The threonine, isoleucine correlated mutation occurs not just in one clade, as was the case with [α-syn 53, GCase 115], but in three: bats, afrotheres and amphibians. None of these three clades are sister groups. Therefore, the chance that the same random mutations occurred in these unrelated groups is quite small, suggesting that the [α-syn 124, GCase 78] correlation arises from a real interaction.

#### #3, #5, #6 & #9 α-Syn 68 & 95 with GCase 101 & 154

The third highest MI Z-value ranked pair belongs to a group of four of the top ten pairs, between α-syn residues 68 and 95 and GCase residues 101 and 154. Groups of correlated pairs are not unusual; in fact, they are expected. While the aim of the DCA and related methods has been to disentangle direct from indirect correlations, one quite successful correlated mutation analysis, SCA, capitalizes on indirect correlations, identifying correlated “sectors” of proteins, that is, sets of residues that are inter-correlated.[48] The α-syn 68 & 95 and GCase 101 & 154 pairs also rank in the top ten intra-protein correlations in both the DCA and Z-value analyses. In the structure of human GCase, residues 101 and 154 are separated by 11 Å, both surface-exposed with most of the space between them unoccupied (see Fig 4A). Thus, it is plausible that either α-syn residue 68 or 95 or both might lie between the two GCase residues. In addition, the T154 side chain is hydrogen-bonded to the backbone at the beginning of a helix, thus a T154V mutation could cause a significant local structural change.

For the number three ranked pair, [α-syn 68, GCase 101], the putative correlated mutation involves an α-syn G68E mutation in Laurasiatheres, Afrotheres and Xenarthrans, and a GCase Q101R mutation in Laurasiatheres, suggesting the possible evolution of a salt bridge. One problem with this particular correlated pair is that it fails the phylogenetic bias test. For instance, a single G68E mutation in α-syn in the common ancestor of placental mammals, which happened to later revert back to glycine for most Supraprimates, plus a single Q101R mutation in GCase in the common ancestor of Laurasiatheres is all that is needed. Hence, there is a significant probability that [α-syn 68, GCase 101] could be correlated by chance.

The number five ranked pair, [α-syn 95, GCase 101] also fails the phylogenetic bias test, but the number six and nine ranked pairs, [α-syn 68, GCase 154] and [α-syn 95, GCase 154] have an additional mutation that suggests the correlation might not be due to chance. One Afrothere, the elephant shrew (E. edwardii), has the same GCase T154V mutation as seen for Laurasiatheres. The other three GCase sequences for Afrotheres have T154, suggesting the T154V mutation in E. edwardii might have arisen after the other mammal clades split from Afrotheres. In this case, elephant shrews and the Laurasiatheres would not be sister groups, suggesting the correlations with T154 might be real.

#### #8 and #15 [α-syn 46, GCase 180] and [α-syn 50, GCase 261]

The number eight and fifteen ranked pairs include residues corresponding to the PD-associated α-syn mutations E46K and H50Q. Also, the number seven ranked pair [α-syn 45, GCase 180] shows a similar substitution pattern as α-syn 46. The seven and eight ranked pairs show correlated mutations occurring for one amphibian (A. mexicanum), one turtle (C. mydas) and one lizard (A. carolinensis), but not in the other amphibian and reptile sequences, so it is likely the correlated mutations arose independently. However, the mutations are conservative, α-syn K45R, α-syn E46D, and GCase T180S. Moreover, GCase residue 180 is completely buried, so if these pairs are truly correlated, the interaction would have to be allosteric. The number fifteen ranked pair also passes the phylogenetic bias test, in this case the H50Q, A261V variation seen for penguin (A. forsteri) and coelacanth (L. chalumnae), and additionally H50Q, A261I seen for anole (A. carolinensis). Curiously, as with the A53T mutation, the PD associated H50Q mutation mirrors the substitution seen in the different species. The two other PD-related α-syn mutations, A30P and G51D, occur in invariant positions in the species studied, so the analysis cannot address them. Finally, the number four and ten ranked pairs involve variations only in bovines and cetaceans, respectively, so they fail the phylogenetic bias test.

### Conserved surface regions of α-syn and GCase

Protein interaction interfaces are known to contain more conserved residues than non-interacting regions of protein surfaces,[60] hence α-syn and GCase were searched for conserved regions. α-Syn is intrinsically disordered in solution, and while transient intra-protein contacts occur,[61] all its residues are potential candidates for contacts with binding partners. The most conserved regions encompass residues 6–85, containing the seven imperfect amphipathic repeats, and residues 123–136, the region that interacts with GCase in solution (Fig 5).[21,62] All but the number five and nine ranked correlated pairs have α-syn residues that lie in these conserved regions.

In contrast to α-syn, GCase is a folded protein with a well-defined surface that can be searched for conserved regions. The largest region lies in and around the enzyme active site, with invariant residues comprising an area of roughly 300 Å^2^ of the surface. The second largest area, covering roughly 200 Å^2^, lies on the opposite side of GCase and includes invariant residues 79, 227, 228, 429, 452, and 454. This second region is shown in Fig 4B, along with the surface regions of GCase residues from selected top Z-value ranked pairs. The region lies between GCase residues V78 and G115, from the number one and two ranked correlated pairs, supporting the hypothesis that α-syn has coevolved with GCase to maintain contact with this region.

### Comparison with β-syn

**Fig 6:**
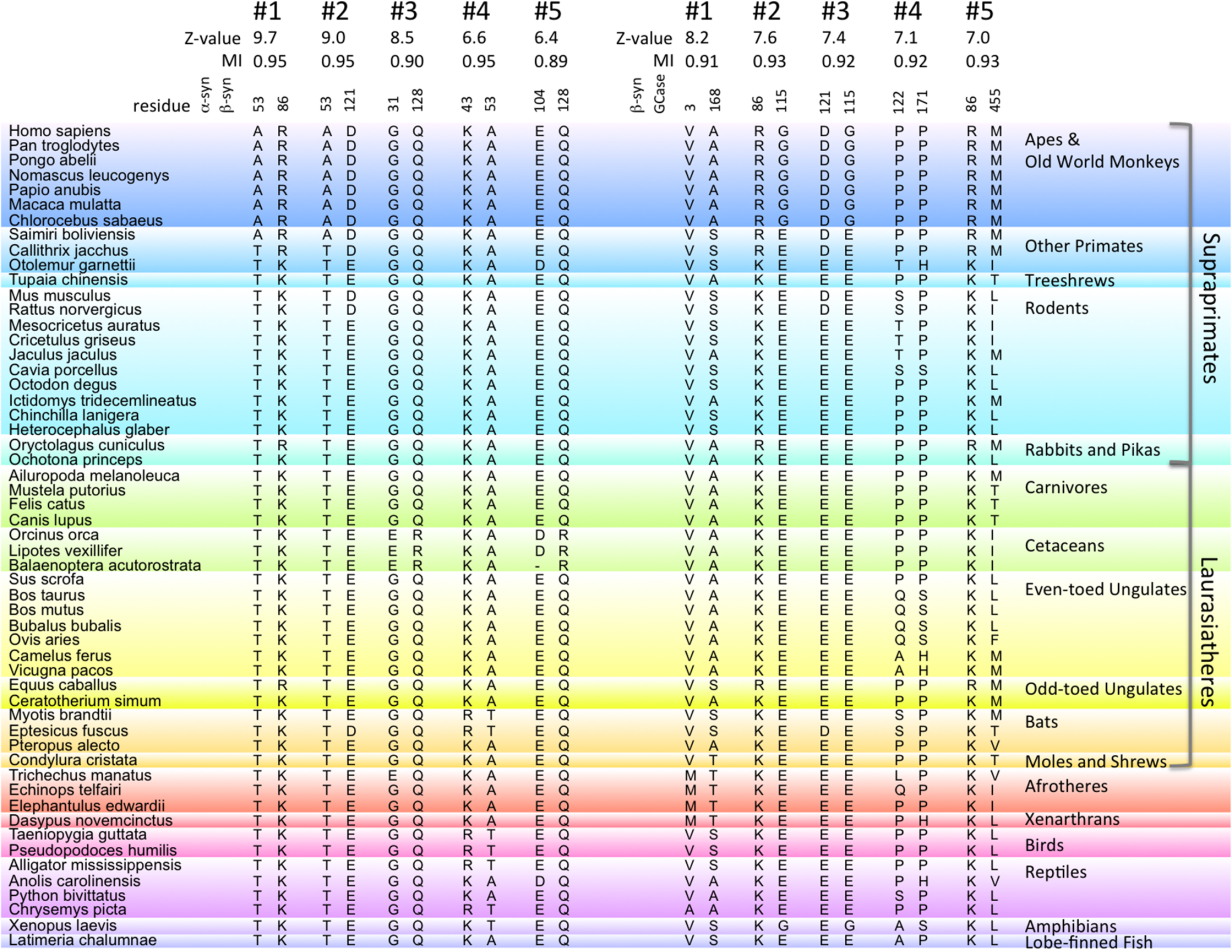
Top MI Z-value ranked α-syn, β-syn and β-syn, GCase correlated residue pairs. Residue pairs of β-syn with α-syn and GCase residues for 55 vertebrate species with the most highly correlated mutations, as determined by the Z-value analysis, are displayed, with the Z-values and MI values shown. For β-syn, sequences for E. caballus and C. ferus were not found, so the closely related E. ferus przewalskii and C. bactrianus were used.

The closest homolog of α-syn is β-syn, and correlations between them and with GCase were also examined by MI Z-value analysis in the 55 species where complete sequences of all three are known. α-Syn and β-syn are known to interact *in vitro* and *vivo* [25], and both are present in high amounts in the presynaptic spaces of central nervous system neurons. β-Syn can also interact with GCase, inhibiting its activity *in vitro*, though 4-fold less so than α-syn.[63] Fig 6 shows the top five ranked MI Z-values for the α-syn, β-syn and β-syn, GCase analyses. Interestingly, the most highly correlated α-syn, β-syn pairs involve α-syn 53, just as with α-syn and GCase. The top two pairs are α-syn 53 with β-syn residues 86 and 121, with Z-values of 9.7 and 9.0, respectively, though keep in mind that the smaller data set, 55 vs. 72 species, is expected to yield larger Z-values. In particular, residue 86 involves a Lys to Arg substitution in apes, Old and New World monkeys, rabbit (O. cuniculus) and horse (E. caballus), that is, the same species as for the α-syn A53T substitution (Fig 3) plus marmoset (C. jacchus), rabbit and horse. β-Syn residue 121 involves a Glu to Asp substitution in apes, Old and New World monkeys, mouse (M. musculus), rat (R. norvegicus), and bat (E. fuscus). Recall that the top ranked α-syn, GCase pair showed the GCase E115G substitution only in apes, Old World monkeys and frog (X. laevis). Because the β-syn residues have even more substitutions than α-syn 53, they are not as strongly correlated with GCase 115, with Z-values of 7.6 and 7.4 for β-syn 86 and 121, respectively. As a final note, like α-syn 53, the most common residue for β-syn 53 is alanine, though bats, birds and reptiles can have threonine, and the most highly correlated residue to β-syn 53 is α-syn 43, which involves a Lys to Arg substitution, just like the β-syn 86 substitution.

## Discussion

### DCA and MI Z-values

In the test using hemoglobin, DCA was superior at identifying intra-chain contacts in hemoglobin, while for inter-chain contacts the MI Z-value method was superior. Reducing the number of species in the analysis from 314 to 72 resulted in lowered accuracy for both methods; however, they both still performed quite well. For example, the percentage of correctly predicted intra-chain contacts in the top ten DCA and MI Z-value ranked pairs went from 60% to 40% and from 50% to 30%, respectively. Surprisingly similar results were obtained in an analysis of metazoan G-protein coupled receptors, where using MIp, a method similar to MI Z-values, a drop in accuracy of 52% to 28% was seen analyzing alignments consisting of 283 and 107 species.[64]

The DCA and MI Z-value methods differ in their formulation and implementation, but both utilize mutual information terms, and both are “global” correlation methods, that is, the final residue pair correlations are the result of a calculation influenced by all the other residue pairs. In DCA this occurs in the initial stage, when the joint amino acid frequencies are adjusted so that DI values exclude contributions from indirect correlations.[37] In the MI Z-value method, the globalization occurs in the final step, when the residue pair MI Z-value is calculated by how many standard deviations its MI value deviates from the MI values of other pairs with one residue in common. One difference that might influence to the inter-chain result is that inter-chain DI values are calculated in the context of both intra and inter-chain pairs, while MI Z-values are calculated with inter-chain pairs only.

Of course, there are many other methods of correlated mutation analysis, and hemoglobin is just one complex. Any generalization of these results should await tests with additional methods and complexes, and factors such as the impact of interacting partners with differing mutational rates must be assessed. Hemoglobin was chosen since as a protein present in vertebrates, the species list could be reduced to closely match those in the α-syn and GCase sequence alignments. While hemoglobin may be unique among vertebrate protein complexes in the number sequences known and structures solved,[40] there are many bacterial protein complexes where 1000+ sequences and multiple structures are known. DCA, the related evolutionary couplings (EVcouplings), and other methods have been successfully applied to these bacterial systems.[35,37,38]

### Comparison with experimental data on α-syn/GCase interaction

Several experiments have shown a physical interaction between α-syn and GCase. Their interaction has been measured both in human tissue homogenates and *in vitro*. GCase has been co-immunoprecipitated with α-syn in lysates prepared from brain tissue, and they co-localize in neuroblastoma cells that over-express both proteins.[21] *In vitro*, residues 118–137 in the C-terminal region of α-syn contact GCase in solution,[21] and when the proteins are membrane-bound, a much larger α-syn region, including residues 57, 100 and 136, interacts with GCase.[63] Fig 5 shows the α-syn sequence with the regions that interact with GCase indicated. In solution, only the number two ranked correlated pair [α-syn 124, GCase 78] involves an α-syn residue in the observed interacting region. However, for α-syn interacting with GCase in the presence of lipid vesicles, eight of the top ten correlated pairs (#1 –#3, #5 –#9) involve α-syn residues near ones observed to interact.

While the precise location on GCase where α-syn interacts is not known, α-syn binds more weakly to GCase with the common N370S mutation.[21] The weaker binding was measured in the absence of lipid, so the interaction must involve C-terminal α-syn residues 118–137. Residue 370 is not solvent exposed, but based on crystal structures, it is thought to influence the surrounding structure, including a loop near the active site.[65] Of the GCase residues in the top ten Z-value ranked pairs, the one closest to N370 is V78 of the [α-syn 124, GCase 78] pair, with a distance of 10 Å. Taken together, the weaker binding to the N370S mutant and the correlated mutation analysis are consistent with the α-syn C-terminal region interacting with a GCase surface region that includes V78.

### Biological implications

The most interesting pairs of residues identified by the correlated mutation analysis are the number one, eight and fifteen ranked pairs, [α-syn 53, GCase 115], [α-syn 46, GCase 180] and [α-syn 50, GCase 261], since mutations of these α-syn residues are known Parkinson’s disease risk factors.[49,50,66,67] In addition, the GCase residue of the number two ranked pair is associated with a Gaucher disease mutation, V78A.[27] In a correlated mutation study involving over 1000 human proteins, residues with known disease-associated mutations occurred in highly ranked intra-protein correlated pairs with a much higher frequency than expected by chance, especially at the protein surface.[68] The results here hint that this observation will likely extend to inter-protein correlated pairs too.

It could not be ruled out that the [α-syn 53, GCase 115] correlation might be a phylogenetic artifact, via genetic drift or perhaps due to evolutionary selection related to primate neural development or function, but not involving a direct α-syn interaction with GCase. Nevertheless, experiment has shown that α-syn 53 must be near GCase when membrane bound (Fig 5),[63] and GCase 115 lies adjacent to the second largest region of conserved GCase surface (Fig 4B), consistent with this region being involved in intermolecular interaction, so an interaction between these residues is plausible. In addition, the other α-syn mutant-associated correlations appear less likely to have arisen by chance. If any of these mutant-associated pairs are truly correlated, then the implications are two-fold. First, this would mean that α-syn and GCase have co-evolved to maintain a beneficial interaction that involves these pairs in some way. Second, it suggests that GCase might be directly involved in PD pathology caused by the alteration in interaction due to their mutation.

The set of residue pairs [α-syn 68, GCase 154] and [α-syn 95, GCase 154] also have possible implications regarding how the α-syn/GCase protein interaction might be related to PD etiology. Residues 68 and 95 of α-syn lie in the NAC region of α-syn (Fig 5), a portion of α-syn critical for the amyloid formation in PD.[23] GCase and α-syn interact in the lysosome, one of the cellular locations where α-syn is degraded, so interaction with the NAC region could affect how α-syn gets degraded. GCase mutations that interfere with this putative interaction or that result in less GCase reaching the lysosome could alter the accessability of α-syn NAC region to lysosomal proteases, which could promote PD pathology. GCase also has an enzymatic cofactor, saposin C, that can compete with α-syn binding, rescuing α-syn-induced inhibition of the enzyme, thus GCase mutations that modify saposin C interaction could also impact α-syn interaction.[62,69]

The analysis of β-syn showed weaker correlation than α-syn with GCase, but also revealed a strong correlation between α-syn residue 53 and β-syn residues 86 and 121, the same α-syn residue in the top α-syn, GCase correlated pair. Colocalization of α-syn and β-syn is observed the presynaptic termini of neurons, and β-syn can mitigate α-syn induced toxicity and accumulation.[25] *In vitro* β-syn can interact with both α-syn and GCase, which might suggest some sort of competitive interaction.[25,63] However, there is no experimental evidence of any significant β-syn, GCase interaction *in vivo*.[31] The residue substitutions in β-syn residues 86 and 121 might have occurred earlier in primate evolution than those in α-syn 53, since the β-syn substitutions are seen in an additional primate species, marmoset (C. jacchus). The substitution for GCase residue 115 occurs for just Old World monkeys and apes, that is, one less primate species than the α-syn 53 substitution, so the GCase substitution could have occurred later. Thus, a causal chain is possible, with the β-syn 53 substitution leading to the α-syn 53 substitution, which then led to the GCase substitution. However, while it is conceivable that the α-syn A53T mutation could somehow alter interaction with β-syn, possibly impacting PD etiology, the genetic link between PD and β-syn is weak at best.[70]

## Conclusion

The coevolutionary analysis of α-syn and GCase has shown that PD-associated alpha-synuclein mutations mirror highly correlated residue substitutions across vertebrate species, providing evidence linking altered α-syn/GCase interaction to PD pathology. Correlations between α-syn and β-syn hint that the same α-syn residue of the top ranked α-syn, GCase pair, α-syn 53, might also be involved in interactions with β-syn. The GCase residues of the two top ranked pairs lie adjacent one of the largest, highly conserved regions of the GCase surface, as one would expect for interacting proteins that have coevolved. While the predicted pairs are consistent with current experimental knowledge of α-syn/GCase interaction, more mutational and structural work is needed to verify whether the correlated pairs truly influence the complex *in vivo*. This study illustrates how sequences of non-human genomes can potentially help us understand human disease, but also highlights how more genome sequencing, especially of vertebrate genomes, is still needed for more robust analyses.

## Supporting Information

S1 Table Hemoglobin DI values. (TXT)

S2 Table Hemoglobin MI Z-values. (TXT)

S3 Table α-syn, GCase MI Z-values. (TXT)

S4 Table β-syn, GCase MI Z-values. (TXT)

S5 Table α-syn, β-syn MI Z-values. (TXT)

S6 Table α-syn, GCase DI values. (TXT)

S1 Text Multiple sequence alignments for hemoglobin, α-syn, GCase and β-syn. (DOCX)

S2 Text Appendix I. Sequence errors and the importance of the pseudocount correction.
(DOCX)

S3 Text Appendix II. Probability estimate of a chance correlation.
(DOCX)
