# Supplementary material for: Did α-Synuclein and Glucocerebrosidase Coevolve? Implications for Parkinson’s Disease
Source: PLoS One. 2015 Jul 27;10(7):e0133863. doi: 10.1371/journal.pone.0133863 (PMC4516260; doi:10.1371/journal.pone.0133863)
Supplement: S1 Text — (DOCX) [file pone.0133863.s007.docx]

**Hemoglobin, α-syn, GCase, and β-syn MSAs**

Hemoglobin alpha chain

>human

VLSPADKTNVKAAWGKVGAHAGEYGAEALERMFLSFPTTKTYFPHFDLSHGSAQVKGHGKKVADALTNAVAHVDDMPNALSALSDLHAHKLRVDPVNFKLLSHCLLVTLAAHLPAEFTPAVHASLDKFLASVSTVLTSKYR

>chimp

VLSPADKTNVKAAWGKVGAHAGEYGAEALERMFLSFPTTKTYFPHFDLSHGSAQVKGHGKKVADALTNAVAHVDDMPNALSALSDLHAHKLRVDPVNFKLLSHCLLVTLAAHLPAEFTPAVHASLDKFLASVSTVLTSKYR

>bonobo

VLSPADKTNVKAAWGKVGAHAGEYGAEALERMFLSFPTTKTYFPHFDLSHGSAQVKGHGKKVADALTNAVAHVDDMPNALSALSDLHAHKLRVDPVNFKLLSHCLLVTLAAHLPAEFTPAVHASLDKFLASVSTVLTSKYR

>orangabe

VLSPADKTNVKAAWGKVGAHAGEYGAEALERMFLSFPTTKTYFPHFDLSHGSAQVKGHGKKVADALTNAVAHVDDMPNALSALSDLHAHKLRVDPVNFKLLSHCLLVTLAAHLPAEFTPAVHASLDKFLASVSTVLTSKYR

>orangpyg

VLSPADKTNVKTAWGKVGAHAGDYGAEALERMFLSFPTTKTYFPHFDLSHGSAQVKDHGKKVADALTNAVAHVDDMPNALSALSDLHAHKLRVDPVNFKLLSHCLLVTLAAHLPAEFTPAVHASLDKFLASVSTVLTSKYR

>gorilla

VLSPADKTNVKAAWGKVGAHAGDYGAEALERMFLSFPTTKTYFPHFDLSHGSAQVKGHGKKVADALTNAVAHVDDMPNALSALSDLHAHKLRVDPVNFKLLSHCLLVTLAAHLPAEFTPAVHASLDKFLASVSTVLTSKYR

>gibbonlar

VLSPADKTNVKAAWGKVGAHAGDYGAEALERMFLSFPTTKTYFPHFDLSHGSAQVKGHGKKVADALTNAVAHVDDMPNALSALSDLHAHKLRVDPVNFKLLSHCLLVTLAAHHPAEFTPAVHASLDKFLASVSTVLTSKYR

>gibbonleu

VLSPADKNNVKAAWGKVGAHAGDYGAEALERMFLSFPTTKTYFPHFDLSHGSAQVKGHGKKVADALTNAVAHVDDMPNALSALSDLHAHKLRVDPVNFKLLSHCLLVTLAAHHPAEFTPAVHASLDKFLASVSTVLTSKYR

>mandrill

VLSPADKKNVKAAWDKVGGHAGEYGAEALERMFLSFPTTKTYFPHFNLSHGSDQVKGHGKKVADALTLAVGHVDDMPQALSKLSDLHAHKLRVDPVNFKLLSHCLLVTLAAHLPAEFTPAVHASLDKFLASVSTVLTSKYR

>baboon

VLSPDDKKHVKAAWGKVGEHAGEYGAEALERMFLSFPTTKTYFPHFDLSHGSDQVNKHGKKVADALTLAVGHVDDMPQALSKLSDLHAHKLRVDPVNFKLLSHCLLVTLAAHLPAEFTPAVHASLDKFLASVSTVLTSKYR

>gelada

VLSPDDKKHVKDAWGKVGEHAGQYGAEALERMFLSFPTTKTYFPHFDLSHGSDQVKKHGKKVADALTLAVGHVDDMPQALSKLSDLHAHKLRVDPVNFKLLSHCLLVTLAAHLPAEFTPAVHASLDKFLASVSTVLTSKYR

>mangabey

VLSPDDKKHVKAAWGKVGEHAGEYGAEALERMFLSFPTTKTYFPHFNLSHGSDQVKGHGKKVADALTLAVGHVDDMPHALSKLSDLHAHKLRVDPVNFKLLSHCLLVTLAAHLPAEFTPAVHASLDKFLASVSTVLTSKYR

>marmoset

VLSPADKSNVKAAWGKVGSHAGDYGAEALERMFLSFPTTKTYFPHFDLSHGSAQVKGHGKKVADALTNAVAHVDDMPNALSALSDLHAHKLRVDPVNFKLLSHCLLVTLAAHHPAEFTPAVHASLDKFLASVSTVLTSKYR

>marmosetarg

VLSPADKSNVKAAWGKVGSHAGDYGAEALERMFLSFPTTKTYFPHFDLSHGSAQVKGHGKKVADALTNAVAHVDDMPNALSALSDLHAHKLRVDPVNFKLLSHCLLVTLAAHHPAEFTPAVHASLDKFLASVSTVLTSKYR

>langur

VLSPADKTNVKAAWGKVGGHGGEYGAEALERMFLSFPTTKTYFPHFDLSHGSAQVKGHGKKVADALTNAVAHVDDMPHALSALSDLHAHKLRVDPVNFKLLSHCLLVTLAAHLPAEFTPAVHASLDKFLASVSTVLTSKYR

>macaquefus

VLSPADKSNVKAAWGKVGGHAGEYGAEALERMFLSFPTTKTYFPHFDLSHGSAQVKGHGKKVADALTLAVGHVDDMPNALSALSDLHAHKLRVDPVNFKLLSHCLLVTLAAHLPAEFTPAVHASLDKFLASVSTVLTSKYR

>macaquefas

VLSPADKTNVKAAWGKVGGHAGEYGAEALERMFLSFPTTKTYFPHFDLSHGSAQVKGHGKKVADALTLAVGHVDDMPHALSALSDLHAHKLRVDPVNFKLLSHCLLVTLAAHLPAEFTPAVHASLDKFLASVSTVLTSKYR

>macaquemul

VLSPADKSNVKAAWGKVGGHAGEYGAEALERMFLSFPTTKTYFPHFDLSHGSAQVKGHGKKVADALTLAVGHVDDMPQALSALSDLHAHKLRVDPVNFKLLSHCLLVTLAAHLPAEFTPAVHASLDKFLASVSTVLTSKYR

>macaquenem

VLSPADKTNVKAAWGKVGGHAGEYGAEALERMFLSFPTTKTYFPHFDLSHGSAQVKGHGKKVADALTLAVDHVDDMPQALSALSDLHAHKLRVDPVNFKLLSHCLLVTLAAHLPAEFTPAVHASLDKFLASVGTVLTSKYR

>macaquespe

VLSPADKTNVKAAWDKVGGHAGEYGAEALERMFLSFPTTKTYFPHFDLSHGSAQVKGHGKKVADALTLAVGHVDDMPHALSALSDLHAHKLRVDPVNFKLLSHCLLVTLAAHLPAEFTPAVHASLDKFLASVSTVLTSKYR

>spidermonkeygeo

VLSPADKSNVKAAWGKVGGHAGDYGAEALERMFLSFPTTKTYFPHFDLSHGSAQVKGHGKKVADALTNAVAHVDDMPNALSALSDLHAHKLRVDPVNFKLLSHCLLVTLAAHHPADFTPAVHASLDKFLASVSTVLTSKYR

>spidermonkeyfus

VLSPADKSNVKAAWGKVGGHAGDYGAEALERMFLSFPTTKTYFPHFDLSHGSAQVKGHGKKVADALTNAVAHVDDMPNALSALSDLHAHKLRVDPVNFKLLSHCLLVTLAAHHPADFTPAVHASLDKFLASVSTVLTSKYR

>capuchinape

VLSPADKTNVKTAWGKVGGHAGDYGAEALERMFLSFPTTKTYFPHFDLSHGSAQVKGHGKKVADALSNAVAHVDDMPNALSALSDLHAHKLRVDPVNFKLLSHCLLVTLAAHHPADFTPAVHASLDKFLASVSTVLTSKYR

>capuchinceb

VLSPADKTNVKTAWGKVGAHAGDYGADALERMFLSFPTTKTYFPHFDLSHGSAQVKGHGKKVADALSNAVAHVDDMPNALSALSDLHAHKLRVDPVNFKLLSHCLLVTLAAHHPADFTPAVHASLDKFLASVSTVLTSKYR

>tamarin

VLSPADKSNVKAAWGKVGGHAGDYGAEALERMFLSFPTTKTYFPHFDLSHGSAQVKGHGKKVADALTNAVAHVDDMPNALSALSDLHAHKLRVDPVNFKLLSHCLLVTLAAHHPADFTPAVHASLDKFLASVSTVLTSKYR

>tamarinnfus

VLSPADKSNVKAAWGKVGGHAGDYGAEALERMFLSFPTTKTYFPHFDLSHGSAQVKGHGKKVADALTVAVAHVDDMPNALSALSDLHAHKLRVDPVNFKLLSHCLLVTLAAHLPADFTPAVHASLDKFLASVSTVLTSKYR

>tamarinnmys

VLSPADKSNVKAAWGKVGGHAGDYGAEALERMFLSFPTTKTYFPHFDLSHGSAQVKGHGKKVADALTNAVALVDDMPNALSALSDLHAHKLRVDPVNFKLLSHCLLVTLAAHHPADFTPAVHASLDKFLASVSTVLTSKYR

>greenmonkey

VLSPADKSNVKAAWGKVGGHAGEYGAEALERMFLSFPTTKTYFPHFDLSHGSAQVKGHGKKVADALTLAVGHVDDMPHALSALSDLHAHKLRVDPVNFKLLSHCLLVTLAAHLPAEFTPAVHASLDKFLASVSTVLTSKYR

>colobus

VLSPADKTNVKTAWGKVGGHGGEYGAEALERMFLSFPTTKTYFPHFDLSHGSAQVKGHGKKVADALTLAAAHVDDMPSALSALSDLHAHKLRVDPVNFKLLSHCLLVTLAAHHPAEFTPAVHASLDKFLASVSTVLTSKYR

>blackwhitelemur

VLSPADKNNVKSAWNAIGSHAGEHGAEALERMFLSFPPTKTYFPHFDLSHGSAQIKTHGKKVADALTNAVNHIDDMPGALSALSDLHAHKLRVDPVNFKLLSHCLLVTLASHHPAEFTPAVHASLDKFFAAVSTVLTSKYR

>lessbamboolemur

VLSSADKTNIKTAWGAIGSHAADHGAEALERMFLSFPTTKTYFPHFDMSHGSGQI-AHGKKVADALTNAVGHIDDMPGALSALSDLHAHKLRVDPVNFKLLSHCLLVTLASHHPAEFTPAVHASLDKFFAAVSTVLTSKYR

>loris

VLSPADKTNVKAAWEKVGSHAGDYGAEALERMFLSFPTTKTYFPHFDLSHGSAQVKAHGKKVADALTNAVSHVDDMPSALSALSDLHAHKLRVDPVNFKLLSHCLLVTLACHHPADFTPAVHASLDKFLASVSTVLTSKYR

>loristar

VLSPADKTNVKGAWEKVGGHAGEYGAEALERMFLSFPTTKTYFPHFDLSHGSAQVKAHGKKVADALTTAVSHVDDMPSALSALSDLHAHKLRVDPVNFKLLSHCLLVTLACHHPADFTPAVHASLDKFLASVSTVLTSKYR

>galagocra

VLSPTDKSNVKAAWEKVGAHAGDYGAEALERMFLSFPTTKTYFPHFDLSHGSTQVKGHGKKVADALTNAVLHVDDMPSALSALSDLHAHKLRVDPVNFKLLRHCLLVTLACHHPAEFTPAVHASLDKFMASVSTVLTSKYR

>tarsier

VLSPADKTNVKAAWDKVGGHAGDYGAEALERMFLSFPTTKTYFPHFDLSHGSSQVKGHGKKVADALTTAVGHIDDMPNALSALSDLHAHKLRVDPVNFKLLSHCLLVTLACHHPADFTPAVHASLDKFVASVSTVLTSKYR

>tarsierbac

VLSPADKTNVKAAWDKVGGHAGDYGAEALERMFLSFPTTKTYFPHFDLSHGSSQVKGHGKKVADALTTAVGHIDNMPNALSALSDLHAHKLRVDPVNFKLLSHCLLVTLACHHPADFTPAVHASLDKFVASVSTVLTSKYR

>orca

VLSPADKTNVKGTWAKIGNHSAEYGAEALERMFINFPSTKTYFSHFDLGHGSAQIKGHGKKVADALTKAVGHIDNLPDALSELSDLHAHKLRVDPVNFKLLSHCLLVTLALHLPADFTPSVHASLDKFLASVSTVLTSKYR

>dolphin

VLSPADKTNVKGTWAKIGNHSAEYGAEALERMFINFPSTKTYFSHFDLGHGSAQIKGHGKKVADALTKAVGHIDNLPDALSELSDLHAHKLRVDPVNFKLLSHCLLVTLALHLPADFTPSVHASLDKFLASVSTVLTSKYR

>botlledolphin

VLSPADKTNVKGTWSKIGNHSAEYGAEALERMFINFPSTKTYFSHFDLGHGSAQIKGHGKKVADALTKAVGHIDNLPDALSELSDLHAHKLRVDPVNFKLLSHCLLVTLALHLPADFTPSVHASLDKFLASVSTVLTSKYR

>spermwhale

VLSPADKTNVKAAWAKVGNHAADFGAEALERMFMSFPSTKTYFSHFDLGHNSTQVKGHGKKVADALTKAVGHLDTLPDALSDLSDLHAHKLRVDPVNFKLLSHCLLVTLAAHLPGDFTPSVHASLDKFLASVSTVLTSKYR

>minkewhale

VLSPTDKSNVKATWAKIGNHGAEYGAEALERMFMNFPSTKTYFPHFDLGHDSAQVKGHGKKVADALTKAVGHMDNLLDALSDLSDLHAHKLRVDPANFKLLSHCLLVTLALHLPAEFTPSVHASLDKFLASVSTVLTSKYR

>waterbuffalo

VLSAADKSNVKAAWGKVGGHAADYGAEALERMFLSFPTTKTYFPHFDLSHGSAQVKGHGAKVANALTKAVGHLDDLPGALSELSDLHAHKLRVDPVNFKLLSHSLLVTLASHLPNDFTPAVHASLDKFLASVSTVLTSKYR

>cow

VLSAADKGNVKAAWGKVGGHAAEYGAEALERMFLSFPTTKTYFPHFDLSHGSAQVKGHGAKVAAALTKAVEHLDDLPGALSELSDLHAHKLRVDPVNFKLLSHSLLVTLASHLPSDFTPAVHASLDKFLASVSTVLTSKYR

>bison

VLSAADKGNVKAAWGKVGGHAAEYGAEALERMFLSFPTTKTYFPHFDLSHGSAQVKGHGAKVAAALTKAVGHLDDLPGALSELSDLHAHKLRVDPVNFKLLSHSLLVTLASHLPNDFTPAVHASLDKFLANVSTVLTSKYR

>gayal

VLSAADKGNVKAAWGKVGDHAAEYGAEALERMFLSFPTTKTYFPHFDLSHGSAQVKGHGAKVAAALTKAVGHLDDLPGALSELSDLHAHKLRVDPVNFKLLSHSLLVTLASHLPNDFTPAVHASLDKFLANVSTVLTSKYR

>yak

VLSAADKGNVKAAWGKVGGHAAEYGAEALERMFLSFPTTKTYFPHFDLSHGSAQVKGHGAKVAAALTKAVGHLDDLPGALSELSDLHAHKLRVDPVNFKLLSHSLLVTLASHLPSDFTPAVHASLDKFLANVSTVLTSKYR

>hippo

VLSANDKSNVKAAWGKVGNHAPEYGAEALERMFLSFPTTKTYFPHFDLSHGSSQVKAHGKKVADALTKAVGHLDDLPGALSDLSDLHAHKLRVDPVNFKLLSHCLLVTLAAHHPSDFTPAAHASLDKFLANVSTVLTSKYR

>sheep

VLSAADKSNVKAAWGKVGGNAGAYGAEALERMFLSFPTTKTYFPHFDLSHGSAQVKGHGEKVAAALTKAVGHLDDLPGTLSDLSDLHAHKLRVDPVNFKLLSHTLLVTLACHLPNDFTPAVHASLDKFLANVSTVLTSKYR

>barbarysheep

VLSAADKSNVKAAWGKVGGNAGAYGAEALERMFLSFPTTKTYFPHFDLSHGSAQVKGHGEKVAAALTKAVGHLDDLPGTLSDLSDLHAHKLRVDPVNFKLLSHSLLVTLACHLPNDFTPAVHASLDKFLANVSTVLTSKYR

>goat

VLSAADKSNVKAAWGKVGGNAGAYGTEALERMFLSFPTTKTYFPHFDLSHGSAQVKGHGEKVAAALTKAVGHLDDLPGTLSDLSDLHAHKLRVDPVNFKLLSHSLLVTLACHLPNDFTPAVHASLDKFLANVSTVLTSKYR

>moose

VLSATDKSNVKAAWGKVGGNAPAYGAEALERMFLSFPTTKTYFPHFDLSHGSAQVKAHGEKVANALTKAVGHLDDLPGTLSDLSDLHAHKLRVDPVNFKLLSHTLLVTLAAHLPSDFTPAVHASLDKFLANVSTVLTSKYR

>reindeer

VLSAADKSNVKAAWGKVGGNAPAYGAEALERMFLSFPTTKTYFPHFDLSHGSAQVKAHGEKVANALTKAVGHLDDLPGTLSDLSDLHAHKLRVDPVNFKLLSHTLLVTLASHLPSDFTPAVHASLDKFLANVSTVLTSKYR

>camel

VLSSKDKTNVKTAFGKIGGHAAEYGAEALERMFLGFPTTKTYFPHFDLSHGSAQVKAHGKKVGDALTKAADHLDDLPSALSALSDLHAHKLRVDPVNFKLLSHCLLVTVAAHHPGDFTPSVHASLDKFLANVSTVLTSKYR

>alpaca

VLSSKDKANIKTAFGKIGGHAADYGAEALERMFLGFPTTKTYFPHFDLSHGSAQVKAHGKKVGDALTKAADHLDDLPSALSALSDLHAHKLRVDPVNFKLLSHCLLVTVAAHHPGDFTPAVHASLDKFLANVSTVLTSKYR

>kudu

-LSAADKGHVKAAWGKVGSHAAEYGAEALERMFLSFPTTKTYFPHFDLSHGSAQVKGHGAKVAAALTKAVDHLDDLPGALSDLSDLHAHKLRVDPVNFKLLSHSLLVTLASHLPGDFTPAVHASLDKFLANVSTVLTSKYR

>pig

VLSAADKANVKAAWGKVGGQAGAHGAEALERMFLGFPTTKTYFPHFNLSHGSDQVKAHGQKVADALTKAVGHLDDLPGALSALSDLHAHKLRVDPVNFKLLSHCLLVTLAAHHPDDFNPSVHASLDKFLANVSTVLTSKYR

>rhino

VLSPTDKTNVKTAWGHVGAQAGEYGAEALERMFLSFPTTKTYFPHFDLSHGSAQVKAHGKKVGDALTQAVGHLDDLPGALSALSDLHAYKLRVDPVNFKLLSHCLLVTLALHHPQDFTPAVHASLDKFLSNVSTVLTSKYR

>indianrhino

VLSPTDKTNVKTAWSHVGAHAGEYGAEALERMFLSFPTTKTYFPHFDLSHGSAQVKAHGKKVGDALTQAVGHLDDLPGALSALSDLHAYKLRVDPVNFKLLSHCLLVTLALHNPQDFTPAVHASLDKFLSNVSTVLTSKYR

>horse

VLSAADKTNVKAAWSKVGGHAGEYGAEALERMFLGFPTTKTYFPHFDLSHGSAQVKAHGKKVGDALTLAVGHLDDLPGALSNLSDLHAHKLRVDPVNFKLLSHCLLSTLAVHLPNDFTPAVHASLDKFLSSVSTVLTSKYR

>mountainzebra

VLSAADKTNVKAAWSKVGGNAGEFGAEALERMFLGFPTTKTYFPHFDLSHGSAQVKAHGKKVGDALTLAVGHLDDLPGALSNLSDLHAHKLRVDPVNFKLLSHCLLSTLAVHLPNDFTPAVHASLDKFLSTVSTVLTSKYR

>donkey

VLSAADKTNVKAAWSKVGGNAGEFGAEALERMFLGFPTTKTYFPHFDLSHGSAQVKAHGKKVGDALTLAVGHLDDLPGALSNLSDLHAHKLRVDPVNFKLLSHCLLSTLAVHLPNDFTPAVHASLDKFLSSVSTVLTSKYR

>kulan

VLSAADKTNVKAAWSKVGGNAGDFGAEALERMFLGFPTTKTYFPHFDLSHGSAQVKAHGKKVGDALTLAVGHLDDLPGALSNLSDLHAHKLRVDPVNFKLLSHCLLSTLAVHLPNDFTPAVHASLDKFLSTVSTVLTSKYR

>tapir

VLSPTDKTNVKAAWSKVGSHAGEYGAEALERMFLGFPTTKTYFPHFDLSHGSAQVQAHGKKVGDALTQAVGHLDDLPGALSALSDLHAYKLRVDPVNFKLLSHCLLVTLALHHPDDFTPAIHASLDKFLSNVSTVLTSKYR

>badger

VLSPADKANIKATWDKIGGHAGEYGGEALERTFASFPTTKTYFPHFDLSHGSAQVKGHGKKVADALTNAVAHLDDLPGALSALSDLHAYKLRVDPVNFKLLSHCLLVTLACHHPAEFTPAVHASLDKFLSSVSTVLTSKYR

>ferret

VLSPADKTNVKSTWDKIGGHAGEYGGEALERTFASFPTTKTYFPHFDLSHGSAQVKAHGKKVADALTNAVAHVDDLPGALSALSDLHAYKLRVDPVNFKLLSHCLLVTLACHHPAEFTPAVHASLDKFFSAVSTVLTSKYR

>otter

VLSPADKTNVKSTWDKIGGHAGEYGGEALERTFVSFPTTKTYFPHFDLSHGSAQVKAHGKKVADALTNAVAHMDDLPGALSALSDLHAYKLRVDPVNFKLLSHCLLVTLACHHPAEFTPAVHASLDKFFSAVSTVLTSKYR

>mink

VLSPADKTNVKSTWDKIGGHAGEYGGEALERTFASFPTTKTYFPHFDLSHGSAQVKAHGKKVADALTNAVAHMDDLPGAMSALSDLHAYKLRVDPVNFKLLSHCLLVTLACHHPAEFTPAVHASLDKFFSAVSTVLTSKYR

>marten

VLSPADKTNVKSTWDKIGGHAGEYGGEALERTFVSFPTTKTYFPHFDLSPGSAQVKAHGKKVADALTLAVGHLDDLAGALSALSDLHAHKLRVDPVNFKLLSHCLLVTLACHHPAEFTPAVHASLDKFFSTVSTVLTSKYR

>giantotter

VLSPADKTNVKATWDKIGGHAGEYGGEALERTFASFPTTKTYFPHFDLSPGSAQVKAHGKKVADALTNAVAHMDDLPAALSALSDLHAYKLRVDPVNFKLLSHCLLVTLACHHPAEFTPAVHASLDKFFSTVSTVLTSKYR

>harborseal

VLSPADKTNVKATWDKIGGHAGEYGGEALERTFTAFPTTKTYFPHFDLSHGSAQVKAHGKKVADALTTAVAHMDDLPGALSALSDLHAHKLRVDPVNFKLLSHCLLVTLACHHPADFTPAVHASLDKFFSAVSTVLTSKYR

>furseal

VLSPADKTNVKTTWDKLGGHAGEYGGEALERTFTAFPTTKTYFPHFDLSHGSAQVKAHGKKVADALTTAVAHMDDLPGALSALSDLHAYKLRVDPVNFKLLSHCLLVTLACHHPAEFTPAVHASLDKFFSAVSTVLTSKYR

>weddellseal

VLSPADKTNVKTTWDKIGGHAGEYGGEALERTFMAFPTTKTYFPHFDLSPGSAQVKTHGKKVADALTTAVSHIDDLPGALSALSDLHAYKLRVDPVNFKLLSHCLLVTLACHHPADFTPAVHASLDKFFSAVSTVLTSKYR

>walrus

VLSPADKTNVKTTWDKLGGHAGEYGGEALERTFMSFPTTKTYFPHFDLSPGSAQVKAHGKKVADALTTAVAHIDDLPGALSALSDLHAYKLRVDPVNFKLLSHCLLVTLACHHPAEFTPAVHASLDKFFSTVSTVLTSKYR

>cat

VLSAADKSNVKACWGKIGSHAGEYGAEALERTFCSFPTTKTYFPHFDLSHGSAQVKAHGQKVADALTQAVAHMDDLPTAMSALSDLHAYKLRVDPVNFKFLSHCLLVTLACHHPAEFTPAVHASLDKFFSAVSTVLTSKYR

>lynx

VLSAADKSNVKACWGKIGSHAGDYGTEALERTFCSFPTTKTYFPHFDLSHGSAQVKAHGQKVADALTQAVAHIDDLPNALSALSDLHAYKLRVDPVNFKFLSHCLLVTLACHHPAEFTPAVHASLDKFFSAVSTVLTSKYR

>jaguar

VLSSADKNNVKACWGKIGSHAGEYGAEALERTFCSFPTTKTYFPHFDLSHGSAQVQAHGQKVADALTKAVAHINDLPNALSDLSDLHAYKLRVDPVNFKFLSHCLLVTLACHHPEEFTPAVHASLDKFFSAVSTVLTSKYR

>amurleopard

VLSSADKNNVKACWGKIGSHAGEYGAEALERTFCSFPTTKTYFPHFDLSHGSAQVQAHGQKVADALTKAVAHINDLPNALSDLSDLHAYKLRVDPVNFKFLSHCLLVTLACHHPEEFTPAVHASLDKFFSAVSTVLTSKYR

>persianleopard

VLSSADKNNVKACWGKIGSHAGEYGAEALERTFCSFPTTKTYFPHFDLSHGSAQVQTHGQKVADALTKAVAHINDLPNALSDLSDLHAYKLRVDPVNFKFLSHCLLVTLACHHPEEFTPAVHASLDKFFSAVSTVLTSKYR

>lion

VLSSADKNNVKACWGKIGSHAGEYGAEALERTFCSFPTTKTYFPHFDLSHGSAQVQAHGQKVADALTKAVVHINDLPNALSDLSDLHAYKLRVDPVNFKFLSHCLLVTLACHHPEEFTPAVHASLDKFFSAVSTVLTSKYR

>tiger

VLSSADKNNVKACWGKIGSHAGEYGAEALERTFCSFPTTKTYFPHFDLSHGSAQVQTHGQKVADALTKAVAHINNLPNALSDLSDLHAYKLRVDPVNFKFLSHCLLVTLACHHPEEFTPAVHASLDKFFSAVSTVLTSKYR

>panda

VLSPADKTNVKATWDKIGGHAGEYGGEALERTFASFPTTKTYFPHFDLSPGSAQVKAHGKKVADALTTAVGHLDDLPGALSALSDLHAHKLRVDPVNFKLLSHCLLVTLASHHPAEFTPAVHASLDKFFSAVSTVLTSKYR

>redpanda

VLSPADKTNVKSTWDKLGGHAGEYGGEALERTFASFPTTKTYFPHFDLSPGSAQVKAHGKKVADALTLAVGHLDDLPGALSALSDLHAHKLRVDPVNFKLLSHCLLVTLACHHPAEFTPAVHASLDKFFSAVSTVLTSKYR

>polarbear

VLSPADKSNVKATWDKIGSHAGEYGGEALERTFASFPTTKTYFPHFDLSPGSAQVKAHGKKVADALTTAAGHLDDLPGALSALSDLHAHKLRVDPVNFKFLSHCLLVTLASHHPAEFTPAVHASLDKFFSAVSTVLTSKYR

>civet

VLSSADKNNIKATWDKIGSHAGEYGAEALERTFISFPTTKTYFPHFDLSHGSAQVKAHGKKVADALTLAVGHLEDLPNALSALSDLHAYKLRVDPVNFKLLSHCLLVTLACHHPAEFTPAVHSALDKFFSAVSTVLTSKYR

>coati

VLSPADKTNIKSTWEKIGSHASEYGGEALERTFASFPTTKTYFPHFDLSPGSAQVKAHGKKVAEALTNAVAHLDDLPGALSTLSDLHAYKLRVDPVNFKFLSHCLLVTLASHHPAEFTPAVHASLDKFFSSVSTVLTSKYR

>raccoon

VLSPADKANIKATWDKIGGHAGEYGGEALERTFASFPTTKTYFPHFDLSPGSAQVKAHGKKVADALTLAVGHLDDLPGALSALSDLHAYKLRVDPVNFKLLSHCLLVTLACHHPAEFTPAVHASLDKFFTSVSTVLTSKYR

>honeybadger

VLSPSDKANVKATWDKIGGHAGEYGGEALERTFASFPTTKTYFPHFDLSPGSAQVKAHGKKVADALTNAVAHGDDLPMALSTLSDLHAYKLRVDPVNFKLLSHCLLVTLACHHPAEFTPAVHASLDKFFSTVSTVLTSKYR

>dog

VLSPADKTNIKSTWDKIGGHAGDYGGEALDRTFQSFPTTKTYFPHFDLSPGSAQVKAHGKKVADALTTAVAHLDDLPGALSALSDLHAYKLRVDPVNFKLLSHCLLVTLACHHPTEFTPAVHASLDKFFAAVSTVLTSKYR

>manedwolf

VLSPADKTNIKSTWDKIGGHAGDYGGEALDRTFQSFPTTKTYFPHFDLSPGSAQVKAHGKKVADALTTAVAHLDDLPGALSALSDLHAYKLRVDPVNFKLLSHCLLVTLACHHPTEFTPAVHASLDKFFTAVSTVLTSKYR

>crabeatingwolf

VLSPADKTNIKSTWDKIGGHAGDYGGEALDRTFQSFPTTKTYFPHFDLSPGSAQVKAHGKKVADALTTAVAHLDDLPGALSALSDLHAYKLRVDPVNFKLLSHCLLVTLACHHPTEFTPAVHASLDKFFTAVSTVLTSKYR

>redfox

VLSPADKTNIKSTWDKIGGHAGDYGGEALDRTFQSFPTTKTYFPHFDLSPGSAQVKAHGKKVADALTTAVAHLDDLPGALSALSDLHAYKLRVDPVNFKLLSHCLLVTLACHHPNEFTPAVHASLDKFFTAVSTVLTSKYR

>aardwolf

VLSSADKANIKATWDKIGGHGGEYGAEALERTFLCFPTTKTYFPHFDLSHGSAQVKAHGKKVADALAVAAAHLDDLPAALSALSDLHAYKLRVDPVNFKLLSHCLLVTLAAHHPAEFTPAVHASLDKFLSSVSTVLTSKYR

>hyena

VLSSADKANIKATWDKIGGHGGEYGAEALERTFLCFPTTKTYFPHFDLSHGSAQVKAHGKKVADALALAAAHLDDLPSALSALSDLHAYKLRVDPVNFKLLSHCLLVTLAAHHPAEFTPAVHSDLDKFLSSVSTVLTSKYR

>pikapri

VLSPADKANVKAAWGKVGGHAGEYGAEALERMFLSFPTTKTYFPHFDMSHGSAQVKAHGKKVADALTQAVDHLDDLPSALSALSDLHAHKLRVDPVNFKLLAHCLLVTLANHHPNEFTPAVHASLDKFLASVSTVLTSKYR

>pikacol

VLSPADKANVKAAWGKVGGHAGEYGAEALERMFLSFPTTKTYFPHFDMSHGSAQVKAHGKKVADALTQAVDHLDDLPGALSALSDLHAHKLRVDPVNFKLLAHCLLVTLANHHPNEFTPAVHASLDKFLASVSTVLTSKYR

>pikahyp

VLSAADKANVKAAWGKVGGHAGEYGAEALERMFLSFPTTKTYFPHFDVSHGSAQVKAHGKKVADALTQAVDHLDDLPGALSTLSDLHAHKLRVDPVNFKLLAHCLLVTLANHHPNEFTPAVHASLDKFLASVSTVLTSKYR

>pikadau

VLSPADKANVKAAWGKVGSHAGEYGAEALERMFLSFPTTKTYFPHLDVSHGSAQVKAHGKKVADALTQAVDHLDDLPGALSALSDLHAHKLRVDPVNFKLLAHCLLVTLANHHPNELTPAVHASLDKFLANVSTVLTSKYR

>pikacur

VLSPADKANVKAAWGKVGGHAGEYGAEALERMFLSFPTTKTYFPHFDVTHGSAQVKAHGKKVADALTQVVDHLDDLPGALSALSDLHAQKLRVDPVNFKLLAHCLLVTLANHHPNEFTPAVHASLDKFLANVSTVLTSKYR

>pikalad

VLSPADKANVKAAWGKVGGHAGEYGAEALERMFLSFPTTKTYFPTWDVSHGSAQVKAHGKKVADALTHAVEHLDDLPGALSTFSDLHAHKLRVDPVNFKLLAHCLLVTLANHHPNEFTPAVHASLDKFLANVSTVLTSKYR

>pikaruf

VLSAADKANVQAAWSKVGGHAGEYGAEALERMFLSFPTTKTYFPTWDVSHGSAQVKAHGKKVADALTHAVEHLDDLPGALSALSDLHAHKLRVDPVNFKLLAHCLLVTLANHHPNEFTPAVHASLDKFLANVSTVLTSKYR

>zokor

VLSPADKANVKAAWGKVGGHGGELGAEALERMFTSFPTTKTYFPHFDVSHGSAQVKAHGKKVADALTNAANHLDDLPSALSALSDLHAHKLRVDPVNFKLLSHCLLVTLANHHPNEFTPAVHASLDKFLANVSTVLTSKYR

>vole

VLSPADKANVKAAWGKVGSHAGEYGAEALERMFLSFPTTKTYFPHFDVSHGSAQVKAHGKKVADALTQAVDHLDDLPGALSALSDLHAHKLRVDPVNFKLLAHCLLVTLANHHPNELTPAVHASLDKFLANVSTVLTSKYR

>volepen

VLSGDDKSNLKTAWGKLGGHAGEYGAEALERMFVAYPTTKTYFPHFDVSHGSAQVKGHGKKVADALTTAVGHLDDLPGALSALSDLHAHKLRVDPVNFKLLSHCLLVTLANHLPADFTPAVHASLDKFLASVSTVLTSKYR

>voleoch

VLSGDDKTNIKTAWGKIGGHAGEFGAEALERMFVVYPTTKTYFPHFDVSHGSAQVKGHGKKVADALTTAVGHLDDLPGALSALSDLHAHKLRVDPVNFKLLSHCLLVTLANHIPAEFTPAVHASLDKFLASVSTVLTSKYR

>chipmunk

VLSPADKTNVKAAWEKVGGHGAAYGAEALERMFLSFPTTKTYFPHFDLSHGSAQVQGHGKKVADALANAAGHLDDLPSALSALSDLHAHKLRVDPVNFKLLSHCLLVTLAAHHPAEFTPAVHASLDKFLATVSTVLTSKYR

>chipmunkeast

VLSPADKTNLKAAWHKLGGHGGEYGAEALERMFATFPTTKTYFPHFDLSHGSAQVQGHGEKVADALLHAVGNLDDLPGALSALSDLHAHKLRVDPVNFKLLSHCLLVTLAAHHPAEFTPAVHASLDKFLATVSTVLTSKYR

>goldgrsquirrel

VLSPADKTNVKAAWEKIGGHGAAYGAEALERMFLSFPTTKTYFPHFDLSHGSAQIQGHGKKVADALANAAAHVDDLPGALSALSDLHAHKLRVDPVNFKLLSHCLLVTLAAHHPAEFTPAVHASLDKFLASVSTVLTSKYR

>gundi

VLSAADKTNVKAAWDKIGGHGGEYGAEALERMFLSFPTTKTYFPHFDVSHGSAQVKAHGKKVADALANAASHLDDLPNALSALSDLHAHKLRVDPVNFKLLSHCLLVTLACHHPAEFTPAVHASLDKFLATVATVLTSKYR

>mouse

VLSGEDKSNIKAAWGKIGGHGAEYGAEALERMFASFPTTKTYFPHFDVSHGSAQVKGHGKKVADALANAAGHLDDLPGALSALSDLHAHKLRVDPVNFKLLSHCLLVTLASHHPADFTPAVHASLDKFLASVSTVLTSKYR

>rat

VLSEEDKNNIKKAWVKIGNHAAEIGAETIGRLFIVFPSSKTYFPHFNTSEGSDQVKAHGKKVADALTNAASHLDDLPGALSTLSDLHAHKLRVDPVNFKFLSHCLLVTLASHHPGDFTPAMHASLDKFFASVSTVLTSKYR

>hamsterg

VLSAKDKTNISEAWGKIGGHAGEYGAEALERMFFVYPTTKTYFPHFDVSHGSAQVKGHGKKVADALTNAVGHLDDLPGALSALSDLHAHKLRVDPVNFKLLSHCLLVTLANHHPADFTPAVHASLDKFFASVSTVLTSKYR

>hamsterc

VLSAEDKNNIRTVWSKIGGHGAEYGAEALGRMFVTYPTTKTYFPHFDVSHGSAQVKAHGAKVADALAKAASHLDDLPGTLSSLSDLHAHELRVDPVNFKLLSHCLLVTLANHHPADITPAVHASLDKFLASVSTVLTSKYR

>deermouse

VLSADDKANIKAAWGKIGGHGAEYGAEALERMFCSFPTTKTYFPHFDVSHGSAQVKGHGAKVADALATAAGHLDDLPAALSALSDLHAHKLRVDPVNFKLLSHCLLVTLAAHLPSDFTPAVHASLDKFLASVSTVLTSKYR

>califmouse

VLSADDKANVKAAWGKLGGHGAEYGAEALGRMFCSFPTTKTYFPHFDVSHGSAQVKGHGAKVADALTTAAGHLDDLPGALSALSDLHAHKLRVDPVNFKLLSHCLLVTLAAHHPAEFTPAVHASLDKFLASVSTVLTSKYR

>whitefootmouse

VLSAEDKANVKAAWSKIGGHGAEYGAEALERMFSSFPTTKTYFPHFDVSHGSAQVKGHGAKVADALATAAGHLDDLPGALSALSDLHAHKLRVDPVNFKLLSHCLLVTLAAHHPAEFTPAVHASLDKFLASVSTVLTSKYR

>molerat

VLSPADKSNVKAAWDKIGGHGAQYGAEALTRMFLSFPTTKTYFHHFDLSPGSAQIQGHGKKVADALTTAVGHLDDLPSALSALSDLHAHKLRVDPVNFKLLSHCLLVTLAAHHPAEFTPAVHASLDKFLATVSTVLTSKYR

>moleratehr

VLSPEDKNHVRSTWDKIGGHGAEYGAEALERMFTSFPTTKTYFPHFDVSHGSAQVKAHGKKVADALANAAGHLDDLPGALSALSDLHAHKLRVDPVNFKLLSHCLLVTLANHHPAEFTPGVHASLDKFLASVSTVLTSKYR

>muskrat

VLSGEDKNNIKTAWGKIGGHAAEYGAEALERMFVVYPTTKTYFPHFDVSHGSGQVKAHGKKVADALTTAVGHLDDLPGALSALSDLHAHKLRVDPVNFKLLSHCLLVTLANHIPADFTPAVHASLDKFLASVSTVLTSKYR

>degu

VLSPADKTNVKTAWGKIGGHGAEYGAEALFRMFLSFPTTKTYFHHFDLSAGSAQIKSHGKKVSDALTTAVDHLDDLPTALSALSDLHAHKLRVDPVNFKLLSHCLLVTLSAHHPADFTPAVHASLDKFLATVSTVLTSKYR

>rabbiteur

VLSPADKTNIKTAWEKIGSHGGEYGAEALERMFLGFPTTKTYFPHFDLTHGSEQIKTHGKKVAEALTKAVGHLDDLPGALSALSDLHAHKLRVDPVNFKLLSHCLLVTLANHHPNEFTPAVHASLDKFLANVSTVLTSKYR

>hare

VLSPADKTNIKTAWEKIGSHGGEYGAEAVERMFLGFPTTKTYFPHFDFTHGSEQIKAHGKKVSEALTKAVGHLDDLPGALSALSDLHAHKLRVDPVNFKLLSHCLLVTLANHHPSEFTPAVHASLDKFLANVSTVLTSKYR

>marmot

VLSPADKTNVKAAWEKIGGHGAAYGAEALERMFLSFPTTKTYFPHFDLSHGSAQIQGHGKKVADALANAAAHVDDLPSALSALSDLHAHKLRVDPVNFKLLSHCLLVTLAAHHPAEFTPAVHASLDKFLASVSTVLTSKYR

>califleafbat

VLSAADKGNVKAAWDKVGGQAGEYGAEALERMFLSFPTTKTYFPHFDLAHGSAQVKGHGKKVADALTNAVGHMDDLPGALSALSDLHAYKLRVDPVNFKLLSHCLLVTLASHHPAEFTPAIHASLDKFFASVSTVLTSKYR

>flyingfox

VLSSTDKSNVKAAWDKVGGHVGEYGAEALERMFLSFPTTKTYFPHFDLAHGSSQVKAHGKKVGDALTNAVGHIDDLPGALSALSDLHAYKLRVDPVNFKLLSHCLLVTLASHLPSDFTPAVHASLDKFLASVSTVLTSKYR

>pallidbat

VLSPADKTNVKAAWDKVGGHAGDYGAEALERMFLSFPTTKTYFPHFDLSHGSAQVKGHGKKVGDALGNAVAHMDDLPGALSALSDLHAYKLRVDPVNFKLLSHCLLVTLACHHPGDFTPAVHASLDKFLASVSTVLVSKYR

>mexfreebat

VLSPEDKNNVKAAWSKVGGQAGDYGAEALERMFLSFPTTKTYFPHFDLSHGSAQVKGHGKKVGEALTTAVNHMDDLPGALSTLSDLHAYKLRVDPVNFKLLSHCLLVTLACHNPGEFTPAVHASLDKFLASVSTVLTSKYR

>rousetteaeg

VLSSADKTNIKAAWDKVGGNAGEYGAEALERMFLSFPTTKTYFPHFDLSHGSAQVKGHGKKVGDALTNAVGHLDDLPGALSALSDLHAYKLRVDPVNFKLLSHCLLVTLANHLPSDFTPAVHASLDKFLASVSTVLTSKYR

>greatfruitbat

VLSPADKTNVKAAWDKVGGNVGEYGAEALERMFLSFPTTKTYFPHFDLAHGSPQVKGHGKKVGDALTNAVSHIDDLPGALSALSDLHAYKLRVDPVNFKLLSHCLLVTLASHLPSDFTPAVHASLDKFLASVSTVLTSKYR

>sheathbat

VLSPADKTNVKAAWEKVGGHAGDYGAEALERMFLSFPTTKTYFPHFDLSHGSSQVKGHGKKVGDALGNAVAHMDDLPGALSALSDLHAYKLRVDPVNFKLLSHCLLVTLASHHAADFTPAVHASLDKFLASVSTVLTSKYR

>brandtbat

VLSPADKTNVKAAWDKVGAHAGDYGAEALERMFLSFPTTKTYFPHFDLSHGSAQVKGHGKKVGDALGNAVAHMDDLPGALSALSDLHAYKLRVDPVNFKLLSHCLLVTLACHHPNEFTPAIHASLDKFMASVSTVLVSKYR

>chocobat

VLSPADKSNVKAAWDKVGGSAGDYGAEALERMFLSFPTTKTYFPHFDLSHGSAQVKGHGKKVGDALGNAVAHLDDLPGALSALSDLHAHKLRVDPVNFKLLSHCLLVTVACHHPNDFTPAVHASLDKFLANVSTVLVSKYR

>cavemyotis

VLSPADKTNIKAAWDKVGAHAGDYGAEALERMFLSFPTTKTYFPHFDLSHGSAQVKGHGKKVGDALGNAVAHMDDLPGALSALSDLHAYKLRVDPVNFKLLSHCLLVTLACHLPGEFTPAIHASLDKFLASVSTVLVSKYR

>ghostbat

VLSPADKANVKAAWDKVGGQAGDYGAEALERMFLSFPTTKTYFPHFDLSHGSAQVKAHGKKVGDALSNAAGHLDDLPGALSALSDLHAYKLRVDPVNFKLLSHCLLVTLASHHAAEFTPAVHASLDKFLASVGTVLTSKYR

>greathorseshoebat

VLSPSDKSNVKAAWDKVGGNAGEYGAEALERMFLSFPTTKTYFPHFDLSHGSAQIKGHGKKVGDALTKAVGSIDDLAGALSALSDLHAHKLRVDPVNFKLLSHCLLVTLACHNPGEFTPAIHASLDKFLASVSTVLTSKYR

>indianflyfox

VLSSTDKSNVKAAWDKVGGNVGEYGAEALERMFLSFPTTKTYFPHFDLAHGSSQVKAHGKKVGDALTNAVGHIDDLPGALSALSDLHAYKLRVDPVNFKLLSHCLLVTLASHLPSDFTPAVHASLDKFLASVSTVLTSKYR

>grayflyfox

VLSSTDKSNVKAAWDKVGGNVGEYGAEALERMFLSFPTTKTYFPHFDLAHGSSQVKAHGKKVGDALTNAVGHMDDLPGALSALSDLHAYKLRVDPVNFKLLSHCLLVTLANHLPNDFTPAVHASLDKFLASVSTVLTSKYR

>brownbat

VLSPADKTNVKAAWDKVGAHAGDYGAEALERMFLSFPTTKTYFPHFDLSHGSAQVKGHGKKVGDALGNAVAHMDDLPGALSALSDLHAYKLRVDPVNFKLLSHCLLVTLACHHPNEFTPAIHASLDKFMASVSTVLVSKYR

>falsevampbat

VLSAADKANVKAAFDKVGGQAGDYGAEALERMFLSFPTTKTYFPHFDLSHGSAQVKAHGKKVGDALVNAVGHLDDLPGALSALSDLHAYKLRVDPVNFKLASNVLLVTLAVHVAAGFTPAVHASLDKFLASVGTVLTSKYR

>sloth

VLSAADKAHVKAFWTKIGGHAGEYGGEALERTFLSFPTTKTYFPHFDLSPGSAQVKAHGKKVGDALTLAVGHLDDLPGALSDLSDLHAHKLRVDPVNFKLLGHCVLVTLALHHPDAFTPAVHASLDKFITTVSTVLTSKYR

>armadillo

VLSAADKTHVKAFWGKVGGHAAEFGAEALERMFASFPPTKTYFSHMDLSHGSAQVKAHGKKVADALTLAVGHLDDLPGALSTLSDLHAHKLRVDPVNFKFLSHCLLVTLACHLPDDFTPAVHASMDKFMAGVSTVLVSKYR

>africanelephant

VLSDNDKTNVKATWSKVGDHASDYVAEALERMFFSFPTTKTYFPHFDLGHGSGQVKAHGKKVGEALTQAVGHLDDLPSALSALSDLHAHKLRVDPVNFKLLSHCLLVTLSSHQPTEFTPEVHASLDKFLSNVSTVLTSKYR

>asianelephant

VLSDKDKTNVKATWSKVGDHASDYVAEALERMFFSFPTTKTYFPHFDLSHGSGQVKGHGKKVGEALTQAVGHLDDLPSALSALSDLHAHKLRVDPVNFKLLSHCLLVTLSSHQPTEFTPEVHASLDKFLSNVSTVLTSKYR

>mammoth

VLSDNDKTNVKATWSKVGDHASDYVAEALERMFFSFPTTKTYFPHFDLSHGSGQVKGHGKKVGEALTQAVGHLDDLPSALSALSDLHAHKLRVDPVNFKLLSHCLLVTLSSHQPTEFTPEVHASLDKFLSNVSTVLTSKYR

>aardvark

VLSGDDKANVKAAWGKVGGKAGDYGAEALERTFLSFPPTKTYFPHFDLSHGSAQVKAHGKKVADALTHAVGHMDDLPGALSDLSDLHAHKLRVDPVNFKLLSHCLLVTLASHHPDDFTPAVHASLDKFLSNVSTVLTSKYR

>hyrax

VLSAADKNNVKGAWEKVGTHAGEYGAEALERMFLSFPTTKTYFPHFDLTHGSAQVKAHGQKVGAALTKAVGHLDDLPNALSDLSDLHAHKLRVDPVNFKLLSHCLLVTLSRHLPQEFTPAVHASLDKFFSNVSTVLTSKYR

>manateeamaz

VLSDEDKTNVKTFWGKIGTHTGEYGGEALERMFLSFPTTKTYFPHFDLSHGSGQIKAHGKKVADALTRAVGHLEDLPGTLSELSDLHAHRLRVDPVNFKLLSHCLLVTLSSHLREDFTPSVHASLDKFLSSVSTVLTSKYR

>elephantshrew

VLSAADKANVKAAWDKAGGNIGAYGGEALDRTFLSFPTTKTYFPHFDQTPGSKDIMNHGKKVADALTLAVNHLDDLPQALSALSDLHAHKLRVDPVNFKLLSHCLLVTLAAHLGKEFTPAIHASLDKFLSTVSTTLTSKYR

>tenrec

VLSAADKANVKAVWEKAGGNVGKYGGEALDRTFLSFPTTKTYFPHMDLTPGSADIMAHGKKVADALTLAVGHMDDLPGALSKLSDLHAYKLRVDPVNFKLLSHCLLVTLACHLGGDFTPAAHASLDKFLSSVSTVLTSKYR

>goldmole

VLSAADKANVKAVWEKAGGDVAKFGGEALNRTFLSFPTTKTYFSHMDLTPGSGDIMGHGKKVADALTVAVGHLDDLPGALSKLSDLHAYKLRVDPVNFKLLSHCLLVTLASHLPGDFSPPVHASMDKFLSTVSTVLTSKYR

>tupaiac

VLSPGDKSNIKAAWGKIGGQAPQYGAEALERMFLSFPTTKTYFPHFDMSHGSAQIQAHGKKVADALSTAVGHLDDLPTALSALSDLHAHKLRVDPANFKLLSHCILVTLACHHPGDFTPEIHASLDKFLANVSTVLTSKYR

>tupaiag

VLSPGDKSNIKAAWGKIGGQAPQYGAEALERMFLSFPTTKTYFPHFDMSHGSAQIQAHGKKVADALSTAVGHLDDLPTALSALSDLHAHKLRVDPANFKLLSHCILVTLACHHPGDFTPEIHASLDKFLANVSTVLTSKYR

>shorttailshrew

VLSASDKTNLKAAWDKLGGQAANYGAEALERTFASFPTTKTYFPHFDLSPGSAQVKGHGKKVADALTKAVGSLDDLPGALSALSDLHAHKLRVDPVNFKLLSHCLLVTLASHHPADFTPAVHASLDKFLATVSTVLTSKYR

>shrew

VLSANDKANIKAAWDKVGGQAGNYGAEALERTFASFPTTKTYFPHFDLSPGSAQVKGHGKKVADALTKAVGSLDDLPGALSALSDLHAHKLRVDPVNFKLLSHCLLVTLASHHPSEFTPAVHASLDKFLASVSTVLTSKYR

>moleora

VLYDTDKSNIKAAWQKVGTHAGEYGGEALERMFESFPTTKTYFPHFDLKHGSAQVKAHGNKVADALTNAVAHLDDLPGALNTLSDLHAHKLRVDPVNFKLLSHCLLVTLASHHPADFTPAMHASLDKFLATVSTVLTSKYR

>moleeur

VLSGTDKSNIKAAWDKVGAHAGEYGAEALERTFTSFPTTKTYFPHFDLSHGSAQVKAHGKKVADALTNAVGHLDDLPGAMSALSDLHAHKLRVDPVNFKLLSHCLLVTLACHHPNDFTPAVHASLDKFLATVSTVLTSKYR

>asianhouseshrew

VLSANDKANVKAAWDKVGGQAANYGAEALERTFASFPTTKTYFPHYDLSPGSAQVKAHGKKVADALTKAVGSMDDLPGALSALSDLHAHKLRVDPVNFKLLSHCLLVTLAAHHPADFTPAVHASLDKFLASVSTVLTSKYR

>starnosemole

VLSATDKSNIKAFWGKIGDHAGEYGAEALERTFAAFPTTKTYFPHFDLSHGSAQIKGHGKKVADALTNAVNHLDDLPGALSALSDLHAHKLRVDPVNFKLLSHCLLVTLACHHPSDISPAVHASLDKFLATVSTVLTSKYR

>taiwanshrew

VLSANDKANIKAAWDKIGANAGTYGAEALERTFASFPTTKTYFPHFDLNPGSAQVKGHGKKVADALTKAVGSLDDLPGALSALSDLHAHKLRVDPVNFKLLSHCLLVTLASHHPAEFTPAVHASLDKFMASVSTVLTSKYR

>moleaqu

VLYDTDKSNIKAAWQKVGTHAGEYGGEALERMFDCFPTTKTYFPHFDMSPGSAQVKAHGSKVAEALTKAVDHLDDLPGALNVLSDLHAHKLRVDPVNFKLLSHCLLVTLASHLSADFTPAVHASLDKFFATVSTVLTSKYR

>hedgehog

VLSATDKANVKTFWGKLGGHGGEYGGEALDRMFQAHPTTKTYFPHFDLNPGSAQVKGHGKKVADALTTAVNNLDDVPGALSALSDLHAHKLRVDPVNFKLLSHCLLVTLALHHPADFTPAVHASLDKFLATVATVLTSKYR

>greykangaroo

VLSAADKGHVKAIWGKVGGHAGEYAAEGLERTFHSFPTTKTYFPHFDLSHGSAQIQAHGKKIADALGQAVEHIDDLPGTLSKLSDLHAHKLRVDPVNFKLLSHCLLVTFAAHLGDAFTPEVHASLDKFLAAVSTVLTSKYR

>wallaby

VLSAADKGHVKGIWGKVGGHAGEYAAEGLERTFHSFPTTKTYFPHFDLSHGSAQIQAHGKKIADALGQAVEHIDDLPGTLSKLSDLHAHKLRVDPVNFKLLSHCLLVTFAAHLGDAFTPEVHASLDKFLAAVSTVLTSKYR

>tasdev

VLSGADKTNVKAVWSKVGGNAGAYAGEALTRTFLSFPTTKTYFPHFDLSPGSQDVKGHGQKVADALSQAVANLDDLPGTLSKLSDLHAHKLRVDPVNFKLLSHCLIVTLACHLSKDLTPEAHASMDKFFASVATVLTSKYR

>opossumvir

VLSANDKTNVKGAWSKVGGNSGAYMGEALYRTFLSFPTTKTYFPNYDFSAGSAQIKTQGQKIADAVGLAVAHLDDMPTALSSLSDLHAHELKVDPVNFKFLCHNVLVTMAAHLGKDFTPEIHASMDKFLASVSTVLTSKYR

>opossumdom

VLSAADKTNVKAAWSKVGGNSGAYMGEALYRTFLSFPPTKTYFPHFEFSAGSAQIKGQGQKIADAVSLAVAHMDDLATALSALSDLHAHNLKVDPVNFKFLCHNVLVTLASHLGKDFTPEIHASLDKFLALLSTVLTSKYR

>platypus

MLTDAEKKEVTALWGKAAGHGEEYGAEALERLFQAFPTTKTYFSHFDLSHGSAQIKAHGKKVADALSTAAGHFDDMDSALSALSDLHAHKLRVDPVNFKLLAHCILVVLARHCPGEFTPSAHAAMDKFLSKVATVLTSKYR

>echidna

VLTDAEKKEVTSLWGKASGHAEEYGAEALERLFLSFPTTKTYFSHMDLSKGSAQVKAHGKRVADALTTAAGHFNDMDSALSALSDLHAHKLRVDPVNFKLLAHCFLVVLARHHPAEFTPSAHAAMDKFLSRVATVLTSKYR

>chicken

VLSAADKNNVKGIFTKIAGHAEEYGAETLERMFTTYPPTKTYFPHFDLSHGSAQIKGHGKKVVAALIEAANHIDDIAGTLSKLSDLHAHKLRVDPVNFKLLGQCFLVVVAIHHPAALTPEVHASLDKFLCAVGTVLTAKYR

>japanquail

VLSAADKTNVKGIFAKIAGHAEEYGAEALDRMFTTYPQTKTYFPHFDVSHGSAQIKGHGKKVAAALVEAANHIDDIAGTLSKLSDLHAQKLRVDPVNFKLLGQCFLVVVAIHHPAALTPEVHASLDKFLCAVGTVLTAKYR

>turkey

VLSAADKNNVKGIFTKIAGHAEEYGAETLERMFITYPPTKTYFPHFDLSHGSAQIKGHGKKVVAALIEAVNHIDDIAGTLSKLSDLHAHKLRVDPVNFKLLGQCFLVVVAIHHPAALTPEVHASLDKFLCAVGTVLTAKYR

>pheasant

VLSAADKNNVKGIFTKIAGHAEEYGAEALERMFITYPSTKTYFPHFDLSHGSAQIKGHGKKVVAALIEAVNHIDDITGTLSKLSDLHAHKLRVDPVNFKLLGQCFLVVVAIHHPSALTPEVHASLDKFLCAVGTVLTAKYR

>francolin

VLSAADKNNVKGIFGKISSHAEDYGAEALERMFITYPSTKTYFPHFDLSHGSAQVKGHGKKVVAALIEAANHIDDIAGTLSKLSDLHAHKLRVDPVNFKLLGQCFLVVVAIHHPSALTPEVHASLDKFLCAVGNVLTAKYR

>ostrich

VLSGTDKTNVKGIFSKISSHAEEYGAETLERMFITYPQTKTYFPHFDLHHGSAQIKAHGKKVANALIEAVNHIDDISGALSKLSDLHAQKLRVDPVNFKLLGQCFLVVVAIHHPSALTPEVHASLDKFLCAVGAVLTAKYR

>rhea

VLSGPDKTNVKNVFAKIGGHADAYGAETLERMFTTYPQTKTYFPHFDLHHGSAQIKTHGKKVVSALIDAANNIDDIYGALSKLSDLHAQKLRVDPVNFKLLGQCFLVVVAIHHPSLLTPEVHASLDKFLCAVGAVLTAKYR

>punateal

VLSAADKTNVKGVFSKIGGHAEEYGAETLERMFIAYPQTKTYFPHFDLSHGSAQIKAHGKKVAAALVEAVNHIDDITGALSKLSDLHAQKLRVDPVNFKFLGHCFLVVVAIHHPAALTPEVHASLDKFMCAVGAVLTAKYR

>mallard

VLSAADKTNVKGVFSKIGGHAEEYGAETLERMFIAYPQTKTYFPHFDLSHGSAQIKAHGKKVAAALVEAVNHVDDIAGALSKLSDLHAQKLRVDPVNFKFLGHCFLVVVAIHHPAALTPEVHASLDKFMCAVGAVLTAKYR

>wigeon

VLSAADKTNVKGVFSKIGGHAEEYGAETLERMFIAYPQTKTYFPHFDLSHGSAQIKAHGKKVAAALVEAVNHIDDIAGALSKLSDLHAQKLRVDPVNFKFLGHCFLVVVAIHHPAALTPEVHASLDKFLCAVGAVLTAKYR

>shelduck

VLSAADKTNVKGVFSKIGGHADEYGAETLERMFIAYPQTKTYFPHFDLQHGSAQIKAHGKKVAAALVEAVNHIDDIAGALSKLSDLHAQKLRVDPVNFKFLGHCFLVVVAIHHPAALTPEVHASLDKFMCAVGTVLTAKYR

>bluewinggoose

VLSAADKTNVKGVFSKIGGHAEEYGAETLERMFIAYPQTKTYFPHFDLQHGSAQIKAHGKKVAAALVEAVNHIDDITGALSKLSDLHAQKLRVDPVNFKFLGHCFLVVVAIHHPAALTPEVHASLDKFMCAVGAVLTAKYR

>muscovyduck

VLSAADKTNVKGVFSKIGGHAEEYGAETLERMFIAYPQTKTYFPHFDLQHGSAQIKAHGKKVAAALVEAVNHIDDIAGALSKLSDLHAQKLRVDPVNFKFLGHCFLVVVAIHHPAALTPEVHASLDKFMCAVGAVLTAKYR

>cinnamonteal

VLSAADKTNVKGVFSKIGGHAEEYGAEALERMFIAYPQTKTYFPHFDLSHGSAQIKAHGKKVAAALVEAVNHIDDIAGALSKLSDLHAQKLRVDPVNFKFLGHCFLVVVAIHHPAALTPEVHASLDKFMCAVGAVLTAKYR

>capebarrengoose

VLSAADKTNVKGVFSKIGGHAEEYGAETLERMFIAYPQTKTYFPHFDLQHGSAQVKAHGKKVAAALVEAVNHIDDIAGALSKLSDLHAQKLRVDPVNFKFLGHCFLVVVAIHHPSALTPEVHASLDKFLCAVGSVLTAKYR

>graylag

VLSAADKTNVKGVFSKIGGHAEEYGAETLERMFTAYPQTKTYFPHFDLQHGSAQIKAHGKKVAAALVEAVNHIDDIAGALSKLSDLHAQKLRVDPVNFKFLGHCFLVVVAIHHPSALTPEVHASLDKFLCAVGTVLTAKYR

>blackheadduck

VLSAADKNNVKGVFSKIGGHADDYGAETLERMFIAYPQTKTYFPHFDLQHGSAQIKAHGKKVAAALVEAVNHIDDIAGALSKLSDLHAQKLRVDPVNFKFLGHCFLVVVAIHHPAALTPEVHASLDKFMCAVGAVLTAKYR

>canadagoose

VLSAADKTNVKGVFSKIGGHADEYGAETLERMFVAYPQTKTYFPHFDLQHGSAQIKAHGKKVAAALVEAVNHIDDIAGALSKLSDLHAQKLRVDPVNFKFLGHCFLVVVAIHHPSALTPEVHASLDKFLCAVGTVLTAKYR

>maskedduck

VLSAADKTNVKGIFSKIGGHADDYGAETLERMFVTYPQTKTYFPHFDLQHGSAQIKAHGKKVAAALVEAVNHIDDISGALSKLSDLHAQKLRVDPVNFKFLGHCFLVVVAIHHPAALTPEVHASLDKFMCAVGSVLTAKYR

>tuftedduck

VLSAADKTNVKGIFSKIGGHAEEYGAETLERMFITYPQTKTYFPHFDLQHGSAQIKAHGKKVAAALVEAVNHIDDIAGALSKLSDLHAQKLRVDPVNFRFLGHCFLVVLAIHHPSALTPEVHASLDKFMCAVGATLTAKYR

>torrentduck

VLSAADKTNVKGVFSKIGGHADDYGAETLERMFIAYPQTKTYFPHFDLQHGSAQIKAHGKKVAAALVEAVNHIDDITGALSKLSDLHAQKLRVDPVNFKFLGHCFLVVVAIHHPAALTPEVHASLDKFMCAVGAVLTAKYR

>orinocogoose

VLSAADKTNVKGIFSKIGGHADDYGAETLERMFIAYPQTKTYFPHFDLHHGSAQIKAHGKKVAAALVEAVNHIDDIAGALSKLSDLHAQKLRVDPVNFKFLGHCFLVVVAIHHPAALTPEVHASLDKFMCAVGAVLTAKYR

>coscorobaswan

VLSAADKTNVKGVFSKIGGHADDYGAETLERMFIAYPQTKTYFPHFDLQHGSAQIKAHGKKVAAALVEAVNHIDDIAGALSKLSDLHAQKLRVDPVNFKFLGHCFLVVVAIHHPSALTPEVHASLDKFLCAVGAVLTAKYR

>uplandgoose

VLSAADKTNVKGVFSKIGGHADDYGAETLERMFIAYPQTKTYFPHFDLHHGSAQIKAHGKKVAAALVEAVNHIDDIAGALSKLSDLHAQKLRVDPVNFKFLGHCFLVVVAIHHPAALTPEVHASLDKFMCAVGAVLTAKYR

>muskduck

VLSAADKTNVKGVFSKIGGHADDYGAETLERMFIAYPQTKTYFPHFDLQHGSAQIKAHGKKVAAALVEAVNHIDDIAGALSKLSDLHAQKLRVDPVNFKFLGHCFLVVVAIHHPSALTPEVHASLDKFMCAVGAVLTAKYR

>barheadgoose

VLSAADKTNVKGVFSKISGHAEEYGAETLERMFTAYPQTKTYFPHFDLQHGSAQIKAHGKKVVAALVEAVNHIDDIAGALSKLSDLHAQKLRVDPVNFKFLGHCFLVVVAIHHPSALTAEVHASLDKFLCAVGTVLTAKYR

>andeangoose

VLSAADKANVKGVFSKIGGHADDYGAETLERMFIAYPQTKTYFPHFDLHHGSAQIKAHGKKVAAALVEAVNHIDDITGALSKLSDLHAQKLRVDPVNFKFLGHCFLVVVAIHHPAALTPEVHASLDKFMCAVGAVLTAKYR

>blackswan

VLSAADKTNVKGVFSKIGSHADDYGAETLERMFIAYPQTKTYFPHFDLQHGSAQIKSHGKKVAAALVEAVNHIDDIAGALSKLSDLHAQKLRVDPVNFKFLGHCFLVVVAIHHPSALTPEVHASLDKFLCAVGSVLTAKYR

>trumpetswan

VLSAADKTNVKGVFSKIGGHADEYGAEALERMFITYPQTKTYFPHFDLQHGSAQVKAHGKKVAAALVEAVNHIDDIAGALSKLSDLHAQKLRVDPVNFKFLGHCFLVVVAIHHPSALTPEVHASLDKFLCAVGAVLTAKYR

>freckledduck

VLSAADKSNVKGVFSKIGGHADDYGAETLERMFIAYPQTKTYFPHFDLQHGSAQIKAHGKKVVAALVEAVNHIDDIAGALSKLSDLHAQKLRVDPVNFKFLGHCFLVVVAIHHPSALTPEVHASLDKFMCAVGAVLTAKYR

>whitewhistleduck

VLSAADKSNVKGVFSKIGGHADEYGAETLERMFATYPQTKTYFPHFDLQHGSAQIKAHGKKVAAALVEAVNHIDDIGGALSKLSDLHAQKLRVDPVNFKFLGHCFLVVVAIHHPSALTPEVHASLDKFMCAVGAVLTAKYR

>magpiegoose

VLSAADKGNVKTVFGKIGGHAEEYGAETLQRMFQTFPQTKTYFPHFDLQPGSAQIKAHGKKVAAALVEAANHIDDIAGALSKLSDLHAQKLRVDPVNFKFLGHCFLVVLAIHHPSLLTPEVHASMDKFLCAVATVLTAKYR

>rockhoppenguin

VLSANDKSNVKGVFSKISSHAEEYGAETLERMFTTYPQTKTYFPHFDLHHGSAQVKAHGKKVATALMEAANHIDDIAGALSKLSDLHAQKLRVDPVNFKLLGQCFLVVMAIHHPSALTPEVHASLDKFLCAVGNVLTSKYR

>emperorpenguin

VLSANDKSNVKSIFSKISSHAEEYGAETLERMFTTYPQTKTYFPHFDLHHGSAQVKAHGKKVAAALIEAANHIDDIAGALSKLSDLHAEKLRVDPVNFKLLGQCFMVVVAIHHPSALTPEIHASLDKFLCAVGNVLTSKYR

>macaw

VLSGSDKTNVKGIFSKIGGQAEDYGAEALERMFATFPQTKTYFPHFDVSPGSAQVKAHGKKVAAALVEAANHIDDIATALSKLSDLHAQKLRVDPVNFKLLGQCFLVVVAIHNPSALTPEVHASLDKFLCAVGNVLTAKYR

>roseringparakeet

VLSGTDKTNVKSIFSKIGGQADDYGAEALERMFVTYPQTKTYFPHFDVSPGSAQVKAHGKKVAGGLSEAANHIDDIATSLSKLSDLHAQKLRVDPVNFKLLGQCFLVVVAIHNPSALTPEAHASLDKFLCAVGLVLTAKYR

>treesparrow

VLSPADKSNVKGVFAKIGGQAEEYGADALERMFATYPQTKTYFPHFDLGKGSAQVKGHGKKVAAALVEAVNNIDDLAGALSKLSDLHAQKLRVDPVNFKLLGQCFLVVVATGNPALLTPEVHAPLDKFLCAVGTVLTAKYR

>zebrafinch

VLSAGDKSNVKAVFGKIGGQADEYGADALERMFATYPQTKTYFPHFDLGKGSAQVKGHGKKVAAALVEAANNVDDLAGALSKLSDLHAQKLRVDPVNFKLLGQCFLVVVATRNPSLLTPEVHASLDKFLCAVGTVLTAKYR

>starling

VLSASDKANVKAVFGKIGGQAEEFGAETLERMFATYPQTKTYFPHFDLGKGSAQVKGHGKKVAAALVEAANHVDDIAGALSKLSDLHAQKLRVDPVNFKLLGQCFLVVVASHNPALLTPEVHASLDKFLCAVGTVLTAKYR

>blackbird

VLSAADKTNVKSAFSKIGGQADEYGAETLERMFATYPQTKTYFPHFDLGKGSAQVKAHGKKVAAALVEAANAVDDIAGALSKLSDLHAQKLRVDPVNFKLLGQCFLVTVATHNPSLLTPEVHASLDKFLCAVGTVLTAKYR

>swift

VLSAADKTNVKGVFAKIGGQAEALGGEALARMFAAYPPTKTYFPHFDLSPGSAQVKAHGKKVASALVEAANNIDDIAGALSKLSDLHAQKLRVDPVNFKLLGHCFLVVVAIHHPSVLTPEVHASLDKFLCAVATVLTAKYR

>pigeon

VLSANDKSNVKAVFGKIGGQAGDLGGEALERLFITYPQTKTYFPHFDLSHGSAQIKGHGKKVAEALVEAANHIDDIAGALSKLSDLHAQKLRVDPVNFKLLGHCFLVVVAVHFPSLLTPEVHASLDKFVCAVGTVLTAKYR

>turtledove

VLSASDKSNVKAAFGKIGGQAGDLGGQALERMFITYPQTKTYFPHFDLSHGSAQIKGHGKKVAEALVEAANHIDDIAGALSKLSDLHAQKLRVDPVNFKLLGHCFLVVVAAHFPSLLTPEVHASLDKFVLAVGTVLTAKYR

>stork

VLSANDKSNVRGVFGKISAHADDYGAETLERMFTVHPTQKTYFPHFDLHRGSAQIKAHGKKVAGALLEAVNHIDDIAGALSKLSDLHAQKLRVDPVNFKLLGQCFLVVVAVHHPSLLTPEVHASLDKFLCTVSTVLTDKYR

>flamingorub

VLSSHDKSNVKGLFGKVGGHLEEYCAETLARMFAAYPQTKTYFPHFDLQPGSAQVKAHGKKVAGALAEAANHIDDIASALSKLSDLHQHKLRVDPVNFKLLAHCFLVVMAIHHPSLLTPEVHASLDKFLCAVGTVLTAKYR

>sunbeam

VLSAADKTNVKGLFGKIGGQADEYGAETLARMFATYPQTKTYFPHFDLSPGSAQVKGHGKKVAAALVEAVNNIDDIAGALSKLSDLHAQKLRVDPVNFKLLGQCFLVVVAIRNPAALTPEVHASLDKFLCAVGTVLTAKYR

>violstarfrontlet

VLSAADKTNVKGLFGKIGGQAEEYGAEALARMFATYPQTKTYFPHFDLSPGSAQVKGHGKKVAAALVEAVNNIDDMAGALSKLSDLHAQKLRVDPVNFKLLGQCFLVVVAIRNPAALTPEVHASLDKFLCAVGTVLTAKYR

>bronzyinca

VLSAADKTNVKGLFGKIGGQAEEYGAEALARMFATYPQTKTYFPHFDLSPGSAQVKGHGKKVAAALVEAANNIDDIAGALSKLSDLHAQKLRVDPVNFKLLGQCFLVVVAIRNPAALTPEVHASLDKFLCAVGTVLTAKYR

>speckhummingbird

VLSAADKTNVKGVFAKIGGQAEEYGAETLARMFATYPQTKTYFPHFDLSPGSAQVKGHGKKVAAALVEAVNNIDDIAGALSKLSDLHAQKLRVDPVNFKLLGQCFLVVVAIRNPAALTPEVHASLDKFLCAVGTVLTAKYR

>amazilia

VLSAADKTNVKGIFAKIGGQAEDYGAETLARMFATYPQTKTYFPHFDLSPGSAQVKGHGKKVAAALVEAVNNIDDIAGALSKLSDLHAQKLRVDPVNFKLLGQCFLVVVAIRNPAALTPEVHASLDKFLCAVGTVLTAKYR

>hillstar

VLSAADKSNVKGVFAKIGGQAEEYGAETLARMFATYPQTKTYFPHFDLSPGSAQVKGHGKKVAAALVEAVNNIDDIAGALSKLSDLHAQKLRVDPVNFKLLGQCFLVVVAIRNPAALTPEVHASLDKFLCAVGTVLTAKYR

>goldeagle

VLSANDKTNVKNVFTKISGHAEDYGAEALERMFTTYPPTKTYFPHFDLHHGSAQIKAHGKKVVGALIEAVNHIDDMAGALSKLSDLHAQKLRVDPVNFKLLGQCFLVVVAIHHPSVLTPEVHASLDKFLCAVGNVLTAKYR

>goshawk

VLSANDKTNVKNVFTKIGGHAEEYGAETLERMFTTYPPTKTYFPHFDLHHGSAQIKAHGKKVVGALIEAVNHIDDIAGALSKLSDLHAQKLRVDPVNFKLLGQCFLVVVAIHHPSVLTPEVHASLDKFLCAVGNVLTAKYR

>condor

VLSGSDKTNVKGVFAKIGGHAEDYGAETLERMFITYPQTKTYFPHFDLQHGSAQIKGHGKKVVGALIEAANHIDDIAASLSKLSDLHAQKLRVDPVNFKLLGQCFLVVVAIHHPSVLTPEVHASLDKFLCAVGNVLTAKYR

>cinereousvulture

VLSANDKTNVKTVFTKITGHAEDYGAETLERMFITYPPTKTYFPHFDLHHGSAQIKAHGKKVVGALIEAVNHIDDIAGALSKLSDLHAQKLRVDPVNFKLLGQCFLVVVAIHHPSVLTPEVHASLDKFLCAVGNVLTAKYR

>whiteheadvulture

VLSANDKTNVKTVFTKITGHAEDYGAETLERMFITYPPTKTYFPHFDLHHGSAQIKAHGKKVVGALIEAVNHIDDIAGALSKLSDLHAQKLRVDPVNFKLLGQCFLVVVAIHHPSVLTPEVHASLDKFLCAVGNVLSAKYR

>ruppellsvulture

VLSANDKTNVKNVFTKITGHAEDYGAETLERMFTTYPPTKTYFPHFDLHHGSAQIKAHGKKVVGALIEAVNHIDDIAGALSKLSDLHAQKLRVDPVNFKLLGQCFLVVVAIHHPSVLTPEVHASLDKFLCAVGNVLTAKYR

>skua

VLSGSDKNNVKGVFGKIGGHAEEYGAETLERMFATYPQTKTYFPHFDLQHGSAQVKAHGKKVAAALVEAANHIDDISGALSKLSDLHAQKLRVDPVNFKLLGQCFLVVVAIHHPSVLTPEVHASLDKFLCAVGNVLTAKYR

>blackheadgull

VLSGSDKTNVKGVFGKIGGHAEEYGAETLERMFATYPQTKTYFPHFDLQHGSAQVKAHGKKVAAALVEAANHIDDIAGALSKLSDLHAQKLRVDPVNFKLLGQCFLVVVAIHHPSVLTPEVHASLDKFLCAVGNVLTAKYR

>cormorant

VLSASDKTNVKGVFAKVGGSAEAYGAETLERMFTAYPQTKTYFPHFDLHHGSAQIKAHGKKVAAALVEAANHIDDIAGALSKLSDLHAQKLRVDPVNFKLLGHCFLVVVAIHHPTLLTPEVHASLDKFMCAVAKELTAKYR

>tuatara

MLSASDKANVKAIWSKVCVHAEEYGAETLERMFTVYPSTKTYFPHFDLTHGSAQVKAHGKKVVNAMGEAVNHLDDMAGALLKLSDLHAQKLRVDPVNFKLLAQCFLVVLGVHHPAALTPEVHASLDKFLCAVGLVLTAKYR

>indiancobra

VLTDEDKARVRASWVPVGKNAELYGSETLTRMFAAHPTTKTYFPHFDLSPGSNNLRAHGKKVIDAITEAVNNLDDVAGTLSKLSDLHAQKLRVDPVNFKLLAHCLLVTIAAHNGGVLKPEVIVSLDKFLGDLSKDLVSKYR

>seasnake

VLTEEDKARVRVAWVPVSKNAELYGAETLTRLFAAHPTTKTYFPHFDLSPGSNDLKVHGKKVIDALTEAVNNLDDVAGALSKLSDLHAQKLRVDPDNFQFLGLCLEVTIAAHSGGPLKPEVLLSVDKFLGQISKVLASRYR

>indigosnake

VLTEEDKSRVRAAWGPVSKNAELYGAETLTRLFTAYPATKTYFHHFDLSPGSSNLKTHGKKVIDAITEAVNNLDDVAGALSKLSDLHAQKLRVDPVNFKLLGHCLEVTIAAHNGGPLKPEVILSLDKFLCLVAKTLVSRYR

>iguana

VLTEDDKNHIRAIWGHVDNNPEAFGVEALTRLFLAYPATKTYFAHFDLNPGSAQIKAHGKKVVDALTQAVNNLDDIPDALAKLADLHAEKLRVDPVNFGLLGHCILVTIAAHNHGPLKADVALSMDKFLTKVAKTLVAHYR

>pondslider

VLNACDKANVKAVWNKVAAHVEEYGAETLERMFTVYPQTKTYFPHFDLHHGSAQIRTHGKKVLNALGEAVNHIDDLASALSKLSDIHAQTLRVDPVNFKFLNHCFLVVVAIHHPSVLTPEVHVSLDKFLSAVGTVLTSKYR

>paintedturtle

VLNAGDKANVKAVWNKVAAHVEEYGAETLERMFTVYPQTKTYFPHFDLHHGSAQIRTHGKKVLTALGEAVNHIDDLASALSKLSDIHAQTLRVDPVNFKFLNHCFLVVVAIHQPSVLTPEVHVSLDKFLSAVGTVLTSKYR

>aldabratortoise

VLTAGDKANVKTVWSKVGSHLEDYGSETLERLFVVYPSTKTYFPHFDLHHDSPQVRAHGKKVLSALGEAVNHIDDIPGALSKLSDLHAQNLRVDPVNFKLLNLCFVVVVGRHHPTILTPEVHVSLDKFLSAVAQNLTSKYR

>galapagostoroise

VLTAGDKANVKTVWSKVGSHLEEYGSETLERLFIVYPSTKTYFPHFDLHHDSAQVRAHGRKVLSALGEAVNHIDDIPGALSKLSDLHAQTLRVDPVNFKLLNLCFVVVVGRHHPTILTPEVHVSLDKFLSAVATALTSKYR

>chacotortoise

VLTAGDKANVKTLWSKVGSHLEEYGSETLERLFIVYPSTKTYFPHFDLHHDSAQVRAHGRKVLSALGEAVNHIDDIPGALSKLSDLHAQTLRVDPVNFKLLNLCFVVVVGRHHPTILTPEVHVSLDKFLSAVATALTSKYR

>yellowspotturtle

VLSPGDKANVKTVWSKVSGHVEDYGAETLERLFRVYPSTKTYFPHFDLHHDSAQIRTHGKKVLTAIGEAVSHIDDIASALSKLSDLHAQTLRVDPVNFKLLSHSFLVVLAVHAPSLLTPEVHVSLDKFLVAVSNVLTSKYR

>sideneckedturtle

VLSPGDKANVKTVWSKVSGHVEDYGAETLERLFRVYPSTKTYFPHFDLHHDSAQIRTHGKKVLTAIGEAVSHIDDIASALSKLSDLHAQTLRVDPVNFKLLSHSFLVVLAVHAPSLLTPEVHVSLDKFLVAVSNVLTSKYR

>loggerhead

VLSSGDKANVKSVWSKVQGHLEDYGAETLDRMFTVFPQTKTYFSHFDVHHGSTQIRSHGKKVMLALGDAVNHIDDIATALSALSDKHAHILRVDPVNFKLLSHCLLVVVARHHPTLFTPDVHVSLDKFMGTVSTVLTSKYR

>siamesecroc

VLSSDDKCNVKAVWCKVAGHLEEYGAEALERMFCAYPQTKIYFPHFDLSHGSAQIRAHGKKVFAALHEAVNHIDDLPGALCRLSELHAHSLRVDPVNFKFLAQCVLVVVAIHHPGSLTPEVHASLDKFLCAVSSVLTSKYR

>nilecroc

VLSSDDKCNVKAVWSKVAGHLEEYGAEALERMFCAYPQTKIYFPHFDLSHGSAQIRAHGKKVFAALHEAVNHIDDLPGALCRLSELHAHSLRVDPVNFKFLAQCVLVVVAIHHPGSLTPEVHASLDKFLCAVSSVLTSKYR

>alligators

VLSQEDKSNVKAIWGKASGHLEDYGAEVLERMFCAYPQTKIYFPHFDMSHGSPQIRAHGKKVFSALHEAVNHIDDLPGALCRLSELHAHSLRVDPVNFKFLSHCVLVVFAIHHPCSLSPEVHASLDKFLCAVSAVLTSKYR

>alligatorm

VLSMEDKSNVKAIWGKASGHLEEYGAEALERMFCAYPQTKIYFPHFDMSHNSAQIRAHGKKVFSALHEAVNHIDDLPGALCRLSELHAHSLRVDPVNFKFLAHCVLVVFAIHHPSALSPEIHASLDKFLCAVSAVLTSKYR

>caiman

VLSQEDKSHVKAIWGKVAGHLEEYGAESLERMFCAYPQTKIYFPHFDMSHNSAQIRGHGKKVFAALHDAVNHIDDLAGALCRLSDLHAHNLRVDPVNFKFLSQCILVVFGVHHPCSLTPEVHASLDKFLCAVSAMLTSKYR

>xenopusl

LLSADDKKHIKAIMPAIAAHGDKFGGEALYRMFIVNPKTKTYFPSFDFHHNSKQISAHGKKVVDALNEASNHLDNIAGSMSKLSDLHAYDLRVDPGNFPLLAHNILVVVAMNFPKQFDPATHKALDKFLATVSTVLTSKYR

>xenopusb

LLSADDKKHIKAIMPSIAAHGDKFGGEALYRMFLVNPKTKTYFPTFDFHHNSKQISAHGKKVVDALNEASNHLDNIAGSLSKLSDLHAYDLRVDPGNFPLLAHNILVVVAMNFPKQFDPATHKALDKFLATVSSVLTSKYR

>xenopust

HLTADDKKHIKAIWPSVAAHGDKYGGEALHRMFMCAPKTKTYFPDFDFSEHSKHILAHGKKVSDALNEACNHLDNIAGCLSKLSDLHAYDLRVDPGNFPLLAHQILVVVAIHFPKQFDPATHKALDKFLVSVSNVLTSKYR

>bullfrog

ALNCDDKAHIRAIWPCLASHAEQYGAEALHRMFLCHPQTKTYFPNFDFHANSAHLKNHGKKVMNALTDAVKHLDHPEASLSSLSDLHAFTLRVDPGNFALLSNNILVVVAVHHSDKLSYETHQALDKFLNVVSGLLTSKYR

>axlotl

TLTAEDKALVVVLWGKIAGHTDALGGEALDRLFACFGQSRTYFSHFDLSPGSADVKRHGGKVLNAVGEAAKHIDDLDMGLSKLSDLHAYNLRVDPGNFQLLSHCIQTVLAAHFPADFTPQAQAAWDKFLAAVSGVLTSKYR

>newtcri

KLSADDKHNVKAIWEHVKGHEEAIGAEALCRMFTSLPTTRTYFPTKDIKEGSSFLHSHGKKVMGALSNAVAHIDDIDGALSKLSDKHAEELMVDPANFPKLAHNILVVLGIHLKPHLTYSVHSSVDKFLATVGYVLASKYR

>coelacanth

GLTAADKTLIKSIWGKVEKETEAIGVEALVRLFKCFPQSKVYFDHFDLSPSSQKLHAHAKVVLGALTKAVNHLDNITDTLHDISLVHAKKLLVDPVNFELLGHCLEVALAAHFATDFTPEVHLAIDKFLYEVEKALFETYR

>lungfish

RFSQDDEVLIKEAWGLLHQIPNA-GGEALARMFSCYPGTKSYFFGHDFSANNEKVKHHGKKVVDAIGQGVQHLHDLSSCLHTLSEKHARELMVDPCNFQYLIEAIMTTIAAHYGEKFTPEINCAAEKCLGQIVHVLISLYR

>tuna

TLSDKDKSTVKALWGKISKSADAIGADALGRMLAVYPQTKTYFSHWDMSPGSGPVKAHGKKVMGGVALAVSKIDDLTTGLGDLSELHAFKMRVDPSNFKILSHCILVVVAKMFPKEFTPDAHVSLDKFLASVALALAERYR

>deepwaterdragon

SLSDKDKSAVKALWSKISKSSDAIGNDALSRMIVVYPQTKTYFSHWDVTPGSAHIKAHGIKVMGGIALAVSKIDDLTTGLSDLSEQHAFKLRVDPANFKILNHCILVVISIMFPKDFTPEAHVSLDKFLSAVALALAEKYR

>blackrockcod

SLSDKDKAAVKALWSKIGKSADAIGNDALSRMIVVYPQTKTYFSHWDVTPGSPHIKAHGKKVMGGIALAVSKIDDLKAGLSDLSEQHAYKLRVDPANFKILNHCILVVISTMFPKDFTPEAHVSLDKFLSGVALALAERYR

>emeraldrockcod

SLSDKDKAAVRALWSKIGKSADAIGNDALSRMIVVYPQTKTYFSHWDVTPGSPHIKAHGKKVMGGIALAVSKIDDLKTGLMELSEQHAYKLRVDPANFKILNHCILVVISTMFPKEFTPEAHVSLDKFLSGVALALAERYR

>duskyrockcod

SLSDKDKAAVRALWSKIGKSSDAIGNDALSRMIVVYPQTKIYFSHWDVTPGSPNIKAHGKKVMGGIALAVSKIDDLKTGLMELSEQHAYKLRVDPSNFKILNHCILVVISTMFPKEFTPEAHVSLDKFLSGVALALAERYR

>mawsondragon

SLSDKDKAAVKALWTTISKSSDAIGNDALSRMIVVYPQTKTYFSHWDVTPGSTHIRDHGKKVMGGISLAVSKIDDLKTGLFELSEQHAFKLRVDPANFKILNHCILVVIATMFPKEFTPEAHVSLDKFLSGVALALAERYR

>channelbullbleny

SLTEKDKAAVRALWGKISKSADAIGADALSRMLFVYPQTKTYFTHWDLSPGSVHVKKHGKNVMGGIALAVSKIDDLTNGLMELSEQHAYQLRVDPANFKILSHCILVVVSIMYPKDFTPEAHVSLDKFLSGVSLALAERYR

>patagonianbleny

SLSDKDKAAVKLLWSKISKSSDAIGNDALSRMIVVYPQTKTYFAHWDLSPGSPHVKAHGKTVMGGIALAVSKIDDLRAGLLDLSEQHAYKLRVDPANFKILSHCILVVISMMFPKEFTPEAHVSLDKFLSGVSLALSERYR

>antarctictoothfish

SLSDKDKAAVIALWNKIGKSADVIGNDALSRMIVVYPETKTYFSHWDLAPGSPHIKAHGKKVMGGIALAVTKIDDLKAGLSELSEQHAYKLRVDPSNFKILNHCILVVISIMFPKEFTPDAHVSLDKFLSGVALALAERYR

>saddleplunderfish

SLSDKDKSAVKALWSKINKSADVIGNDAVSRMIVVYPQTKTYFAHWDLTPGSTHIKAHGKKVMGGIALAVSKIDDLKAGLSNLSEQHAFKLRVDPANFKILNHCIMVVISSMFPKDFTPEAHVSLDKFLSAVALALAEKYR

>congolli

SLTDKDKATVKALWGKISKSADAVGADAVGRMIVVYPQTKTYFSHWDLAPNSPHVKTHGKTVMTGIALAVSKIDDLTNGLLELSEEHAYKMRVDPANFKILSHCMLVVIATMFPKEFTPEAHVCLDKFLCAVSLALSERYR

>yellowbellyrockcod

SLSVKDKAAVRALWSKISKSSDAIGNDALSRMIVVYPQTKTYFSHWDVTPGSAHIKAHGKKVMGGIALAVSKIDDLNAGLLELSEQHAYKLRVDPANFKILNHCILVVISTMFPKDFTPEAHVSLDKFLSGVALALAERYR

>sablefish

SLTEKDKATVKALWGKISKSSDAIGADALGRMLVVYPQTKTYFTHWDLSPNSAPVKNHGKNVMSGVALAVSKIDDLTAGLLDLSEQHAFTLRVDPANFKILSHCILVVLAIMFPKEFTPEVHVAMDKFFCGVSLALSEKYR

>artedidraco

SLSDKDKAAVKALWSKIAKSADVIGNDAVSRMIVVYPQTKTYFAHWDLTPGSTNIKAHGKKVMGGIALAVSKIDDLKAGLSDLSEQHAFKLRVDPANFKILNHCIMVVISSMFPKDFTPEAHISLDKFLSAVALALAEKYR

>baldnotothen

SLSEKNKAAVKALWSKIGKSSDAIGNDALSRMIVVYPQTKTYFSHWEVTPGSPHIKAHGKKVMGGIALAVTKIDDLKTGLSELSEQHAYKLRVDPANFKTLNHCILVVISTMFPKEFTPEAHVSLDKFLSGVALALADRYR

>ploughfish

SLSDKDKAAVRALWSTISKSSDAIGNDALSRMIVVYPQTKIYFSHWEVIPGSIHIKEHGKKVMGGIELAVSKIDDLKTGLFELSEQHAFKLRVDPGNFKILNHCILVVIATMFPKEFTPEAHVSLDKFLSGVALALAERYR

>eelpout

SLSDKDKAAVKAIWNKISKSADVIGADAMGRMLVVYPQTKTYFSHWDLSPNSAPVKNHGKTVMTGVALAVSNIDDMTTGLKALSEKHAFQLRVDPSNFKILSHCILVVIAMMYPKDFTPEAHVSMDKFFCGLSLALAEKYR

>wolffish

SLTAKDKDTVRAFWAKASGKAAEIGSDALSRMLVVYPQTKTYFSHWDLSPGSEPVKKHGKSVMGGVADAVMKIEDLNAGLLNLSELHAFTLRVDPANFKILSHNILVVMAIMFPKDFTPEVHVAMDKFLAALSRALAEKYR

>bluefingurnard

SLSDKDKNTVRALWAKISKSADVIGAEALARMLTVYPQTKTYFTHWDLSPSSTSVKNHGKNIMVGVSLAVSKMDDLTAGLLELSEKHAFQLRVDPANFKLLSHCLLVVISIMFPKEFGPEVHVSVDKFFANLALALSERYR

>orangespotgrouper

SLNDKDKAAVKALWAKISKSADTIGSNALTRMFTVYPQTKTYFSHWELTPGSVPVKNHGKKVMNGIGLAVAKIDDLVCGLLELSEQHAFQLRVDPSNFKILSHCILVELATTFPGEFTPEAHVSYDKFLSNVALALSERYR

>kelpsnailfish

SLSTKDKETVKDLWGHISASADAIGADALGRLLVVYPQTKIYFLHWDLSPNSPSVKNHGKNIMSGIALAVTKIDDLKSGLNALSEQHAFQLRVDPANFKLLSHCILVVLAIRFPHEFTPEAHVAMDKFFCGVSLALAEKYR

>yellowperch

SLSSKDKDAVKALWGKIADKAEEIGADALGRMLAVYPQTKTYFSHWDLSPGSAPVNKHGKTIMGGLVDAVASIDDLNAGLLALSELHAFTLRVDPANFKILSHCILVQLAVKFPKDFTPEVHLSYDKFFSAVARALAEKYR

>congereelcathodic

SLTAKDKTLVKTFWGKVKGKADAMGAEALGRMLVVYPQTKTYFAHWDQSPGSEPVKHGKKTIMGAVGDAVGKIDNLLGGLSALSEVHATKLAIDPGNFKLLSHCLLVTFAVNYPTDFTAEVHVAVDKFLAAVSAALADKYR

>brownmorayanodic

SLSTKDKAVVKGFWSKISGKSDEIGTEAVGRMLTVYPQTKAYFSHWETTPGSAPVKKHGARILGAINDAVNRIDDMAGALGSLSELHANKLCVDPANFKILAHCLMASICLFYPTDFTPEVHLSVDKFLQNLALALADRYR

>brownmoraycathodic

SLAPGDKTVVKKFWEKVGGQADEIGGEALSRMIAVYPPTRIYFSHWDLAPGSPSVKKHGKKIMKAVSDSVGKMDNLVGGLSALSDLHATRLHIDPSNFKILSHNILVTLAAHFPSDFTAEVHVAMDKFLSAVCAALSDKYR

>euroeelanodic

SLSAKDMAVVKGFWNKIAPKADEIGGEALGRMLRVFPQTKAYFAHWDTSPNSPEVKKHGALILATIGDVVNRIENMTTVLGSLSDLHAFKLRVDPANFKILGHNIMVVICMTFPNDFTPEVHLSVDKFFQNFTLALSERYR

>euroeelcathodic

SLTAKDKSLITGFWQKISSKADDLGAEALSRMIVVFPATKVYFSHWDLGPGSPSVKKHGKVIMAAVGDAVGKMNDLVGALSALSDLHAFKMRIDPGNFKTLSHNILVACAVNFPVDFTAEVHVAMDKFLAALGAALSDKYR

>carp

SLSAKDKAAVKDLWAKISGKADDIGQDALSRMLVVYPQTKTYFSHWDLSPGSAPVRKHGKTVMSGVAEAVSKIDDLTSGLLNLSELHAFQLRIDPANFKILSHNILVVLATMFPTDFTPEAHVAMDKFLCALSLALSEKYR

>zebrafish

SLSDTDKAVVKAIWAKISPKADEIGAEALARMLTVYPQTKTYFSHWDLSPGSGPVKKHGKTIMGAVGEAVSKIDDLVGGLAALSELHAFKLRVDPANFKILSHNVIVVIAMLFPADFTPEVHVSVDKFFNNLALALSEKYR

>fatheadminnow

SLSDKDKTTVKGLWGKISPKADEIGAEALGRMLTVYPQTKTYFAHWDLSPGSAPVRKHGKVIMGAVGEAVTKIDDLVGGLAALSELHAFKLRIDPANFRILAHNLIVVIAMLFPGDFTPDVHLSVDKFFQNLALALAEKYR

>goldfish

SLSDKDKAVVKALWAKIGSRADEIGAEALGRMLTVYPQTKTYFSHWDLSPGSGPVKKHGKTIMGAVGDAVSKIDDLVGALSSLSELHAFKLRIDPANFKILAHNVIVVIGMLFPGDFTPEVHMSVDKFFQNLALALSEKYR

>grasscarp

SLTARDKAVVKALWSKISSKADEIGAEALGRMLTVYPQTKTYFSHWDLSPGSGPVKKHGKVIVAAVGDAVSKIDDLAGGLAALSELRAFKLRVDPANFKILAHNLIVVIAMLFPADFSPEVHVSVDKFFQNLALALSDKYR

>largescaleloach

SLSEQDKSAVKAHWSKISSRSDDIGAEALGRMLXVYPQTKTYFSDWDLSPGSAPVKKHGKTIMGAVGEAVSKIDDLTGALSALSELHAFKLRIDPANFKILATNLIVVIGMLFPGDFSPEVHVSVDKFFQNLALALSEKYR

>weatherloach

SLSARDKSVVKALWGKISSRADDIGAEALGRMLTVYPQTKTYFSHWDLSPGSAPVKKHGKTIMGAVGEAESKIDEVTGSLAALSELHAFKLRIDPANFKILATNLIVVIGMLFPGDFSPEVDVSVDKFFQNLALALSEKYR

>brycon

SLSDKDKDAIKAFWAKISPKAEDIGADALARTLTVYPQTKTYFSHWDLSPGSAPVKKHGKTVMSGVAEAVSKIDDLTNGLLTLSELHAFQLRVDPANFKILSHNLLVVLAIQFPNDFTPEVHVSMDKFLSALALALSEKYR

>mexicantetra

SLTVDDMAAVKALWGKIGAKADEIGAEALGRMLTVYPQTKTYFAHWDLSPGSAPVKKHGKTIMGAVSAAVASIEDLPGAMSQLSELHAYKLRVDPSNFKILAHNIIVVVGMLFPGEFTPEVHVSLDKFLQNVAWCLAERYR

>electriceel

SLTAKSKSIVKAFWGKIGSRADDIGAEAFGRMLTVYPETKTYFASWDLSPGSAAVKKHGKTIMGGIAEAVGHIDDLTGGLASLSELHAFKLRVDPANFKILAHNLIVVLALFFPADFTPEVHMAVDKFFQNVASALSEKYR

>channelcatfish

SLTDIDKTLVKAFWGKVSGKADAIGHEALVRMLLVYPQTKIYFSHWDLSPGSESVKKHGRTVIGALNDAVVNIDDLVGGLSALSDLHAYKLRVEPGNFKILSHNFLVTLAINFPADFTPEVHIAMDKFLAAVSAALADKYR

>atlanticsalmon

SLTAKDKSVVKAFWGKISGKADVVGAEALGRMLTAYPQTKTYFSHWDLSPGSAPVKKHGSTIMGAIGNAVGLIDDLVGGLSALSDLHAFKLRVDPGNFKILSHNILVTLAIHFPADFTPEVHIAVDKFLAALSAALADKYR

>rainbowtrout

SLTAKDKSVVKAFWGKISGKADVVGAEALGRMLTAYPQTKTYFSHWDLSPGSGPVKKHGGIIMGAIGKAVGLMDDLVGGMSALSDLHAFNLRVDPGNFKILSHNILVTLAIHFPSDFTPEVHIAVDKFLAAVSAALADKYR

>pike

SLSAKDKANVKSIWTKIIAKSDDIGEQAFSRMLVVYPQTKTYFSHWDLKPGSAQVKKHGDTIMNKIDECVNHMEDLVPFLTSLSELHATKLRVDPANFKFLAHCLLVAVAAYLPNDFTPDIHLSVDKFLQLVALALAEKYR

>smelt

SLSSKDKATVKAFWAKVAPKTEEVGSDALSRMLVVYPQTKTYFSHWDLSPGSAPVRTHGKTIIEGVGEAVSKIEDLSNGLINLSELHAFQLRVDPANFKIFCHNIIVVLAILFPDDFTPEAHVSIDKFLAAVSLALQEKYR

>cod

SLSSKDKATVKEFFGKMSTRSDDIGAEALSRLVAVYPQTKSYFSHWDASPGSAPVRKHGITIMGGVYDAVTKIDDLKGGLLSLSELHAFMLRVDPVNFKLLAHCMLVCMSMVFPEEFTPQVHVAVDKFLAQLALALAEKYR

>medaka

SLTEKDKAAVKALWAKISKSADAIGADALSRMLLVYPQTKTYFSHWDTKAGSEPVKKHGKKIMGGVGLAVSKIDDLAAGLLELSELHAFKLRVDPANFKLLAHCLQVVIANMFPKDFTPEAHVACDKFLANVALALSEKYR

>amazonmolly

SLTDKDKAAVKALWAKISKGADAIGAEALARMLVVYPQTKTYFSHWDMSAGSGPVTKHGKKIMGGVALAVAKMDDLSGGLLELSELHAFQLRVDPANFKILAQCLQVVIAIMFPNDFTPEAHVALDKFLSNVALALSEKYR

>platy

SLTAKDKDTVKAFWGKISPKAGAIGADALGRMLVVYPQTKTYFSHWDMSPGSGPVKKHGATVMAGVADAVAKIDDLTAGLLTLSELHAFKLRVDPANFKILAHNLLLVLAISFPNDFTPEVHVAMDKFLSAVALALSEKYR

>fugu

SLSRTDKEAVKAIWAKMSKSIDVIGAEAFGRMLIAYPQTKIYFSEWDLRPASGPVKAHGKKVMGGIATAVASIDDLTCGLRELSERHAFTLKVDPANFRLLAHCILVVTAIMFPKDFTPEIHVSFDKFLAGVALALSDKYR

>turbot

SLTAKDKDAVRAFWSKVAPKAEDIGSDALSRMLVVYPQTKTYFTHWDLSPGSAPVRKHGKTVMRGVAEAVAKIDDLKAGLLELSELHAFTLRVDPANFKILSHNILVVLAIMFPKDFSPEVHVAMDKFLAALARALSEKYR

>flounder

SLSGKDKRVVKAIWEKMSSKSDVIGAEALGRMLVSYPQTKTYFSHWDLSPSSAPVRKHGATIMAAVGDAVGHMDDLQGFLSKYSELHAFKLRVDPTNFKILAHNMILVMAMYFPKDFTAEVHVSVDKFLQCLALALSEKYR

>tilapia

SLTEKDKAAVKALWAKISKSVDAIGAEALGRMLLVYPQTKTYFSHWDLTPGSAPVVSHGKQIMGGVTEAMSKIDNLRGGLLELSELHAFKLRVDPSNFKILAQTIMVVVAAMFPNDFTPEAHVAFDKFLAAVALGLSERYR

>brichardi

SLSEKDKAAVKALWAKVSKSLDTFGGEALGRMLLVYPQTKTYFSHWDLTPGSNPVMVHGKLILGGVTEAVSKIDNLRGGLLELSELHAFKLRVDPSNFKILAHTIMVVIATMFPKDFTPEAHVAFDKFLAAVALGLSEKYR

>burtoni

SLTEKDKAAVKALWAKVSKAVDTVGGEALGRMLLVYPQTKTYFSHWDLNPGSEPVMVHGKLILGGVTEAVSKIDNLSGGLLELSELHAFKLRVDPSNFKILAQSLMVVIATMFPKDFTPETHVAFDKFLAAVALGLSEKYR

>nyererei

SLTEKDKAAVKALWAKVSKVVDTVGGEALGRMLLVYPQTKTYFSHWDLTPGSEPVMVHGKLILGGVTEAVSKIDNLSGGLLELSELHAFKLRVDPSNFKILAQSFMVVIATMFPKDFTPETHVAFDKFLAAVALGLSEKYR

>mbuna

SLSGKDKAVVRNFWAKVSPKSAEIGGEALGRMLTAYPQTKIYFSHWDLSPSSAQVKKHGATIMAAIGDAVSKIDDLTGGLSALSELHAFKLRVDPANFRILSHNIILCMGMFFPADFTPEVHVSVDKFLQNLALALSEKYR

>spotcroaker

SLSATDKARVKALWDKIEGKSAELGAEALGRMLVSFPQTKIYFSEWDLGPQTPQVRNHGAVIMAAVGKAVKSIDNLVGGLSQLSELHAFKLRVDPANFKILAHNIILVISMYFPGDFTPEVHLSVDKFLACLALALSEKYR

>ghostshark

DYSAADRAELAALSKVLAQNAEAFGAEALARMFTVYAATKSYFKDYDFTAAAPSIKAHGAKVVTALAKACDHLDDLKTHLHKLAAFHGSELKVDPANFQYLSYCLEVALA-VHLTEFSPETHCALDKFLTNVCHELSSRYR

>houndshark

AFTACEKQTIGKIAQVLAKSPEAYGAECLARLFVTHPGSKSYF-EYDYSAAGAKVQVHGGKVIRAVVKAAEHVDDLHSHLETLALTHGKKLLVDPQNFPMLSECIIVTLA-THLTEFSPDTHCAVDKLLSAICQELSSRYR

>dogfish

VLSAADKTAIKHLTGSLRTNAEAWGAESLARMFATTPSTKTYFSKFDFSANGKRVKAHGGKVLNAVADATDHLDNVAGHLDPLAVLHGTTLCVDPHNFPLLTQCILVTLA-AHLTELKPETHCALDKFLCEVATALGSHYR

>electricray

VLSEGNKKAIKNLLQKIHSQTEVLGAEALARLFECHPQTKSYFPKFGFSANDKRVKHHGALVLKALVDTNKHLDDLPHHLNKLAEKHGKGLLVDPHNFKLFSDCIAVTLA-AHLQEFSPETHCAVDKFLEEVTYQLSSLYR

>redstingray

VLSSQNKKAIEELGNLIKANAEAWGADALARLFELHPQTKTYFSKFGFEACNEQVKKHGKRVMNALADATHHLDNLHLHLEDLARKHGENLLVDPHNFHLFADCIVVTLA-VNLQAFTPVTHCAVDKFLELVAYELSSCYR

>skate

VLSDANKQEIHHVAELIKAHAEAVGADALARLFELHPQTKTYFPNFGYHATDAPVKAHGAKVINAVLKAAEHLDDLPKHLEKLATKHGHELLVDPHNFVLFSDIIVVTLA-TRLPTFSPATHRAIDKFLEELVHQLSSKYR

Hemoglobin beta chain

>homo sapiens

VHLTPEEKSAVTALWGKVNVDEVGGEALGRLLVVYPWTQRFFESFGDLSTPDAVMGNPKVKAHGKKVLGAFSDGLAHLDNLKGTFATLSELHCDKLHVDPENFRLLGNVLVCVLAHHFGKEFTPPVQAAYQKVVAGVANALAHKYH

>pan troglodytes

VHLTPEEKSAVTALWGKVNVDEVGGEALGRLLVVYPWTQRFFESFGDLSTPDAVMGNPKVKAHGKKVLGAFSDGLAHLDNLKGTFATLSELHCDKLHVDPENFRLLGNVLVCVLAHHFGKEFTPPVQAAYQKVVAGVANALAHKYH

>pan paniscus

VHLTPEEKSAVTALWGKVNVDEVGGEALGRLLVVYPWTQRFFESFGDLSTPDAVMGNPKVKAHGKKVLGAFSDGLAHLDNLKGTFATLSELHCDKLHVDPENFRLLGNVLVCVLAHHFGKEFTPPVQAAYQKVVAGVANALAHKYH

>pongo abelii

VHLTPEEKSAVTALWGKVNVDEVGGEALGRLLVVYPWTQRFFESFGDLSTPDAVMGNPKVKAHGKKVLGAFSDGLAHLDNLKGTFAKLSELHCDKLHVDPENFRLLGNVLVCVLAHHFGKEFTPQVQAAYQKVVAGVANALAHKYH

>pongo pygmaeus

VHLTPEEKSAVTALWGKVNVDEVGGEALGRLLVVYPWTQRFFESFGDLSTPDAVMGNPKVKAHGKKVLGAFSDGLAHLDNLKGTFAKLSELHCDKLHVDPENFRLLGNVLVCVLAHHFGKEFTPQVQAAYQKVVAGVANALAHKYH

>gorilla gorilla

VHLTPEEKSAVTALWGKVNVDEVGGEALGRLLVVYPWTQRFFESFGDLSTPDAVMGNPKVKAHGKKVLGAFSDGLAHLDNLKGTFATLSELHCDKLHVDPENFKLLGNVLVCVLAHHFGKEFTPPVQAAYQKVVAGVANALAHKYH

>hylobartes lar

VHLTPEEKSAVTALWGKVNVDEVGGEALGRLLVVYPWTQRFFESFGDLSTPDAVMGNPKVKAHGKKVLGAFSDGLAHLDNLKGTFAQLSELHCDKLHVDPENFRLLGNVLVCVLAHHFGKEFTPQVQAAYQKVVAGVANALAHKYH

>nomascus leucogenys

VHLTPEEKSAVTALWGKVKVDEVGGEALGRLLVVYPWTQRFFESFGDLSTPDAVMGNPKVKAHGKKVLGAFSDGLAHLDNLKGTFAQLSELHCDKLHVDPENFRLLGNVLVCVLAHHFGKEFTPQVQAAYQKVVAGVANALAHKYH

>mandrillus sphinx

VHLTPEEKTAVTTLWGKVNVDEVGGEALGRLLVVYPWTQRFFDSFGDLSSPDAVMGNPKVKAHGKKVLGAFSDGLNHLDNLKGTFAQLSELHCDKLHVDPENFKLLGNVLVCVLAHHFGKEFTPQVQAAYQKVVAGVANALAHKYH

>papio anubis

VHLTPEEKNAVTALWGKVNVDEVGGEALGRLLVVYPWTQRFFDSFGDLSSPAAVMGNPKVKAHGKKVLGAFSDGLNHLDNLKGTFAQLSELHCDKLHVDPENFKLLGNVLVCVLAHHFGKEFTPQVQAAYQKVVAGVANALAHKYH

>theropithecus gelada

VHLTPEEKNAVTTLWGKVNVDEVGGEALGRLLVVYPWTQRFFDSFGDLSSPAAVMGNPKVKAHGKKVLGAFSDGLNHLDNLKGTFAQLSELHCDKLHVDPENFKLLGNVLVCVLAHHFGKEFTPQVQAAYQKVVAGVANALAHKYH

>cercocebus atys

VHLTPEEKVAVTTLWGKVNVDEVGGEALGRLLVVYPWTQRFFESFGDLSNPDAVMGNPKVKAHGKKVLGAFSDGLNHLDNLKGTFAQLSELHCDKLHVDPENFKLLGNVLVCVLAHHFGKEFTPQVQAAYQKVVAGVANALAHKYH

>Semnopithecus entellus

VHLTPEEKAAVTALWGKVNVDEVGGEALGRLLVVYPWTQRFFESFGDLSSPDAVMGNPKVKAHGKKVLGAFSDGLAHLDNLKGTFAQLSELHCDKLHVDPENFRLLGNVLVCVLAHHFGKEFTPQVQAAYQKVVAGVANALAHKYH

>callithrix jacchus

VHLTGEEKSAVTALWGKVNVDEVGGEALGRLLVVYPWTQRFFESFGDLSTPDAVMNNPKVKAHGKKVLGAFSDGLTHLDNLKGTFAHLSELHCDKLHVDPENFRLLGNVLVCVLAHHFGKEFTPVVQAAYQKVVAGVANALAHKYH

>callithrix argentata

VHLTGEEKSAVTALWGKVNVDEVGGEALGRLLVVYPWTQRFFESFGDLSTPDAVMNNPKVKAHGKKVLGAFSDGLTHLDNLKGTFAHLSELHCDKLHVDPENFRLLGNVLVCVLAHHFGKEFTPVVQAAYQKVVAGVANALAHKYH

>Macaca fuscata

VHLTPEEKNAVTTLWGKVNVDEVGGEALGRLLVVYPWTQRFFESFGDLSSPDAVMGNPKVKAHGKKVLGAFSDGLNHLDNLKGTFAQLSELHCDKLHVDPENFKLLGNVLVCVLAHHFGKEFTPQVQAAYQKVVAGVANALAHKYH

>Macaca fascicularis

VHLTPEEKNAVTTLWGKVNVDEVGGEALGRLLVVYPWTQRFFESFGDLSSPDAVMGNPKVKAHGKKVLGAFSDGLNHLDNLKGTFAQLSELHCDKLHVDPENFKLLGNVLVCVLAHHFGKEFTPQVQAAYQKVVAGVANALAHKYH

>Macaca mulatta

VHLTPEEKTAVTTLWGKVNVDEVGGEALGRLLVVYPWTQRFFDSFGDLSSPDAVMGNPKVKAHGKKVLGAFSDGLNHLDNLKGTFAQLSELHCDKLHVDPENFKLLGNVLVCVLAHHFGKEFTPQVQAAYQKVVAGVANALAHKYH

>Macaca nemestrina

VHLTPEEKNAVTTLWGKVNVDEVGGEALGRLLVVYPWTQRFFESFGDLSSPDAVMGNPKVKAHGKKVLGAFSDGLNHLDNLKGTFAQLSELHCDKLHVDPENFKLLGNVLVCVLAHHFGKEFTPQVQAAYQKVVAGVANALAHKYH

>Macaca speciosa

VHLTPEEKNAVTTLWGKVNVDEVGGEALGRLLVVYPWTQRFFESFGDLSSPDAVMGNPKVKAHGKKVLGAFSDGLNHLDNLKGTFAQLSELHCDKLHVDPENFKLLGNVLVCVLAHHFGKEFTPQVQAAYQKVVAGVANALAHKYH

>Ateles geoffroyi

VHLTGEEKAAVTALWGKVNVDEVGGEALGRLLVVYPWTQRFFESFGDLSTPDAVMSNPKVKAHGKKVLGAFSDGLAHLDNLKGTFAQLSELHCDKLHVDPENFRLLGNVLVCVLAHHFGKEFTPQLQAAYQKVVAGVANALAHKYH

>ateles fusciceps

VHLTGEEKSAVAALWGKVNVDEVGGEALGRLLVVYPWTQRFFESFGALSTPDAVMGNPKVKAHGKKVLGAFSDGLAHLDNLKGTFAQLSELHCDKLHVDPENFRLLGNVLVCVLARNFGKEFTPQVQAAFQKVVAGVATALAHKYH

>cebus apella

VHLTAEEKSAVTTLWGKVNVDEVGGEALGRLLVVYPWTQRFFDSFGDLSTPDAVMNNPKVKAHGKKVLGAFSDGLTHLDNLKGTFAQLSELHCDKLHVDPENFRLLGNVLVCVLAHHFGKEFTPQVQAAYQKVVAGVANALAHKYH

>cebus capucinus

VHLTAEEKSAVTTLWGKVNVDEVGGEALGRLLVVYPWTQRFFDSFGDLSTPDAVMNNPKVKAHGKKVLGAFSDGLTHLDNLKGTFAQLSELHCDKLHVDPENFRLLGNVLVCVLAHHFGKEFTPQVQAAYQKVVAGVATALAHKYH

>saguinus oedipus

VHLTGEEKSAVTTLWGKVNVEEVGGEALGRLLVVYPWTQRFFESFGDLSSPDAVMNNPKVKAHGKKVLGAFSDGLAHLDNLKGTFAQLSELHCDKLHVDPENFRLLGNVLVCVLAHHFGKEFTPQVQAAYQKVVAGVANALAHKYH

>saguinus fuscicollis

VHLTGEEKSAVTTLWGKVNVEEVGGEALGRLLVVYPWTQRFFESFGDLSSPDAVMGNPKVKAHGKKVLGAFSDGLAHLDNLKGTFAQLSELHCNKLHVDPENFRLLGNVLVCVLAHHFGKEFTPQVQAAYQKVVAGVANALAHKYH

>saguinus mystax

VHLTGEEKSAVTTLWGKVNVEEVGGEALGRLLVVYPWTQRFFDSFGDLSSPDAVMNNPKVKAHGKKVLGAFSDGLAHLDNLKGTFAQLSELHCDKLHVDPENFRLLGNVLVCVLAHHFGKEFTPQVQAAYQKVVAGVANALAHKYH

>Chlorocebus sabaeus

VHLTPEEKTAVTTLWGKVNVDEVGGEALGRLLVVYPWTQRFFESFGDLSSPDAVMGNPKVKAHGKKVLGAFSDGLAHLDNLKGTFAQLSELHCDKLHVDPENFKLLGNVLVCVLAHHFGKEFTPQVQAAYQKVVAGVANALAHKYH

>Piliocolobus badius

VHLTPDEKNAVTALWGKVNVDEVGGEALGRLLVVYPWTQRFFDSFGDLSTADAVMGNPKVKAHGKKVLGAFSDGLAHLDNLKGTFAQLSELHCDKLHVDPENFKLLGNVLVCVLAHHFGKEFTPQVQAAYQKVVAGVANALAHKYH

>Varecia variegata

TFLTPEENNHVTSLWGKVNVEKVGGEALGRLLVVYPWTQRFFESFGDLSSPDAIMGNPKVKAHGKKVLTAFSEGLHHLDNLKGTFAQLSELHCDKLHVDPQNFKLLGNVLVIVLAHHFGNDFSPQTQAAFQKVVTGVANALAHKYH

>Hapalemur griseus

TFLTPEENGHVTSLWGKVDVEKVGGEALGRLLVVYPWTQRFFESFGDLSTPSAIMGNPKVKAHGKKVLSAFSEGLHHLDNLKGTFAQLSELHCDKLHVDPQNFTLLGNVLVIVLAEHFGNAFSPPVQAAFQKVVTGVANALAHKYH

>Nycticebus coucang

VHLTGEEKSAVTALWGKVNVDDVGGEALGRLLVVYPWTQRFFESFGDLSSPSAVMGNPKVKAHGKKVLSAFSDGLNHLDNLKGTFAKLSELHCDKLHVDPENFRLLGNVLVVVLAHHFGKDFTPQVQSAYQKVVAGVANALAHKYH

>Loris tardigradus

VHLTGEEKSAVTGLWGKVNVEDVGGEALGRLLVVYPWTQRFFESFGDLSSPSAVMGNPKVKAHGKKVLSAFSDGLNHLDNLKGTFAKLSELHCDKLHVDPENFRLLGNVLVVVLAHHFGKDFTPQVQSAYQKVVAGVANALAHKYH

>Otolemur crassicaudatus

VHLTPDEKNAVCALWGKVNVEEVGGEALGRLLVVYPWTQRFFDSFGDLSSPSAVMGNPKVKAHGKKVLSAFSEGLNHLDNLKGTFAKLSELHCDKLHVDPENFRLLGNVLVVVLAHHFGKDFTPEVQAAYQKVVAGVATALAHKYH

>Tarsius sp.

VHLTADEKAAVTALWGKVDVEDVGGEALGRLLVVYPWTERFFDSFGDLSTPAAVMGDAKVKAHGKKVLNAFSEGMAHLDDLKGTFAKLSELHCDKLHVDPEDFRLLGDVLVCVLAHHFGKEFTPQVEAAYEKVVAGVATALAHKYH

>Cephalopachus bancanus

VHLTADEKAAVTALWGKVDVEDVGGEALGRLLVVYPWTQRFFDSFGDLSTPAAVMGNAKVKAHGKKVLNAFSEGMAHLDNLKGTFAKLSELHCDKLHVDPENFRLLGNVLVCVLAHHFGKEFTPQVQAAYQKVVAGVATALAHKYH

>Orcinus orca

VHLTGEEKSAVTALWGKVNVEEVGGEALGRLLVVYPWTQRFFESFGDLSTADAVMKNPNVKKHGKKVLASFGEGLKHLDDLKGTFAALSELHCDKLHVDPENFRLLGNVLVVVLARHFGKEFTPELQSAYQKVVAGVATALAHKYH

>Delphinus delphis

VHLTGEEKSTVTALWGKVNVEEVGGEALGRLLVVYPWTQRFFESFGDLSTADAVMKNPNVKKHGKKVLASFGEGLKHLDDLKGTFAALSELHCDKLHVDPENFRLLGNVLVVVLARHFGKEFTPEVQSAYQKVVAGVATALAHKYH

>Tursiops truncatus

VHLTGEEKSAVTALWGKVNVEEVGGEALGRLLVVYPWTQRFFESFGDLSTADAVMKNPNVKKHGQKVLASFGEGLKHLDDLKGTFAALSELHCDKLHVDPENFRLLGNVLVVVLARHFGKEFTPELQSAYQKVVAGVATALAHKYH

>Physeter catodon

VHLTGEEKSGLTALWAKVNVEEIGGEALGRLLVVYPWTQRFFEHFGDLSSADAVMKNPKVQAHGKKVLASFGEGLKHLDNLKGTFATLSELHCDKLHVDPENFRLLGNVLVVVLARHFGKEFTPELQTAYQKVVAGVANALAHKYH

>Balaenoptera acutorostrata

VHLTAEEKSAVTALWAKVNVEEVGGEALGRLLVVYPWTQRFFEAFGDLSTADAVMKNPKVKAHGKKVLASFSDGLKHLDDLKGTFATLSELHCDKLHVDPENFRLLGNVLVIVLARHFGKEFTPELQAAYQKVVAGVANALAHKYH

>Bubalus bubalis

-MLTAEEKAAVTAFWGKVHVDEVGGEALGRLLVVYPWTQRFFESFGDLSTADAVMNNPKVKAHGKKVLDSFSNGMKHLDDLKGTFAALSELHCDKLHVDPENFKLLGNVLVVVLARHFGKEFTPVLQADFQKVVTGVANALAHRYH

>Bos taurus

-MLTAEEKAAVTAFWGKVKVDEVGGEALGRLLVVYPWTQRFFESFGDLSTADAVMNNPKVKAHGKKVLDSFSNGMKHLDDLKGTFAALSELHCDKLHVDPENFKLLGNVLVVVLARNFGKEFTPVLQADFQKVVAGVANALAHRYH

>Bison bonasus

-MLTAEEKAAVTAFWGKVHVDEVGGEALGRLLVVYPWTQRFFESFGDLSSADAVMNNAKVKAHGKKVLDSFSNGMKHLDDLKGTFAALSELHCDKLHVDPENFKLLGNVLVVVLARHFGKEFTPVLQADFQKVVTGVANALAHRYH

>Bos frontalis

-MLTAEEKAAVTAFWGKVHVDEVGGEALGRLLVVYPWTQRFFESFGDLSTADAVMNNPKVKAHGKKVLDSFSNGMKHLDDLKGTFAALSELHCDKLHVDPENFKLLGNVLVVVLARHFGKEFTPVLQADFQKVVAGVANALAHRYH

>Bos grunniens

-MLTAEEKAAVTAFWGKVKVDEVGGEALGRLLVVYPWTQRFFESFGDLSTADAVMNNPKVKAHGKKVLDSFSEGMKHLDDLKGTFAALSELHCDKLHVDPENFKLLGNVLVVVLARNFGKEFTPVLQADFQKVVAGVANALAHRYH

>Hippopotamus amphibius

VHLTAEEKDAVLGLWGKVNVQEVGGEALGRLLVVYPWTQRFFESFGDLSSADAVMNNPKVKAHGKKVLDSFADGLKHLDNLKGTFAALSELHCDQLHVDPENFRLLGNELVVVLARTFGKEFTPELQAAYQKVVAGVANALAHRYH

>Ovis aries

-MLTAEEKAAVTGFWGKVKVDEVGAEALGRLLVVYPWTQRFFEHFGDLSNADAVMNNPKVKAHGKKVLDSFSNGMKHLDDLKGTFAQLSELHCDKLHVDPENFRLLGNVLVVVLARHHGNEFTPVLQADFQKVVAGVANALAHKYH

>Ammotragus lervia

-MLTAEEKAAVTGFWSKVKVDDVGAEALGRLLVVYPWTQRFFEHFGDLSSADAVMNNPKVKAHGKKVLDSFSNGMKHLDDLKGTFAHLSELHCDKLHVDPENFRLLGNVLVVVLARHHGSEFTPVLQADFQKVVTGVANALAHRYH

>Capra hircus

-MLTAEEKAAVTGFWGKVKVDEVGAEALGRLLVVYPWTQRFFEHFGDLSSADAVMNNAKVKAHGKKVLDSFSNGMKHLDDLKGTFAQLSELHCDKLHVDPENFRLLGNVLVVVLARHHGSEFTPVLQAEFQKVVAGVANALAHRYH

>Alces alces

-MLTAEEKAAVTAFWGKVKVDEVGGEALGRLLVVYPWTQRFFEHFGDLSTADAVMHNAKVKEHGKRVLDAFSEGLKHLDDLKGAFAKLSELHCDKLHVDPENFRLLGNVLVVVLARHFGKEFTPELQADYQKVVTGVANALAHRYH

>Rangifer tarandus

-MLTSEEKAAVTGFWGKVKVDEVGAEALGRLLVVYPWTQRFFEHFGDLSSADAIMHNDKVKAHGKRVLDAFSDGLKHLDDLKGAFAKLSELHCDKLHVDPENFRLLGNVLVVVLARHFGKDFTPVLQADYQKVVTGVANALAHRYH

>Camelus ferus

VHLSGDEKNAVHGLWSKVKVDEVGGEALGRLLVVYPWTRRFFESFGDLSTADAVMNNPKVKAHGSKVLNSFGDGLNHLDNLKGTYAKLSELHCDKLHVDPENFRLLGNVLVVVLARHFGKEFTPDLQAAYQKVVAGVANALAHRYH

>Vicugna pacos

VNLSGDEKNAVHGLWSKVKVDEVGGEALGRLLVVYPWTRRFFESFGDLSTADAVMNNPKVKAHGSKVLNSFGDGLSHLDNLKGTYAKLSELHCDKLHVDPENFRLLGNVLVVVLARHFGKEFTPDLQAAYQKVVAGVANALAHRYH

>Tragelaphus strepsiceros

-MLTAEEKAAVTAFWGKVKVDEVGGEALGRLLVVYPWTQRFFESFGDLSTADAVMNNPKVKAHGKKVLDSFSNGMKHLDDLKGTFAALSELHCDKLHVDPENFKLLGNVLVVVLARHFGKEFTPELQADYQKVVTGVANALAHRYH

>Sus scrofa

VHLSAEEKEAVLGLWGKVNVDEVGGEALGRLLVVYPWTQRFFESFGDLSNADAVMGNPKVKAHGKKVLQSFSDGLKHLDNLKGTFAKLSELHCDQLHVDPENFRLLGNVIVVVLARRLGHDFNPNVQAAFQKVVAGVANALAHKYH

>Ceratotherium simum

VELTAEEKAAVLALWDKVKEDEVGGEALGRLLVVYPWTQRFFDSFGDLSTPAAVMGNAKVKAHGKKVLHSFGDGVHHLDNLKGTFAALSELHCDKLHVDPENFRLLGNVLVVVLAKHFGKQFTPELQAAYQKVVAGVANALAHKYH

>Rhinoceros unicornis

VDLTAEEKAAVLALWGKVNEDEVGGEALGRLLVVYPWTQRFFDSFGDLSTPAAVLGNAKVKAHGKKVLHSFGDGVHNLDNLKGTYAALSELHCDKLHVDPENFRLLGNVLVVVLAQHFGQEFTPELQAAYQKVVAGVANALAHKYH

>Equus caballus

VQLSGEEKAAVLALWDKVNEEEVGGEALGRLLVVYPWTQRFFDSFGDLSNPGAVMGNPKVKAHGKKVLHSFGEGVHHLDNLKGTFAALSELHCDKLHVDPENFRLLGNVLVVVLARHFGKDFTPELQASYQKVVAGVANALAHKYH

>Equus zebra

VQLSGEEKAAVLALWDKVNEEEVGGEALGRLLVVYPWTQRFFDSFGDLSNPAAVMGNPKVKAHGKKVLHSFGEGVHHLDNLKGTFAQLSELHCDKLHVDPENFRLLGNVLVVVLARHFGKDFTPELQASYQKVVAGVANALAHKYH

>equus asinus

VQLSGEEKAAVLALWDKVNEEEVGGEALGRLLVVYPWTQRFFDSFGDLSNPGAVMGNPKVKAHGKKVLHSFGEGVHHLDNLKGTFAALSELHCDKLHVDPENFRLLGNVLVVVLARHFGKDFTPELQASYQKVVAGVANALAHKYH

>Equus hemionus

VQLSGEEKAAVLALWDKVNEEEVGGEALGRLLVVYPWTQRFFDSFGDLSNPAAVMGNPKVKAHGKKVLHSFGEGVHHLDNLKGTFAQLSELHCDKLHVDPENFRLLGNVLVVVLARHFGKDFTPELQASYQKVVAGVANALAHKYH

>Tapirus terrestris

VELTGEEKAAVLALWDKVDEDKVGGEALGRLLVVYPWTQRFFDSFGDLSTAAAVMGNPKVKAHGKKVLHSFGDGVHHLDDLKVTFAQLSELHCDKLHVDPENFRLLGNVLVVVLAQQFGKAFTPELQAAYQKVVAGVANALAHKYH

>Meles meles

VHLTAEEKSAVTSLWGKVNVDEVGGEALGRLLVVYPWTQRYFDSFGDLSTPDAVMGNPKVKAHGKKVLNSFSEGLKNLDNLKGTFAKLSELHCDKLHVDPENFKLLGNVLVCVLAHHFGKEFTPQVQAAYQKVVAGVANALAHKYH

>Mustela putorius

VHLTGEEKAAVTALWGKVNVDEVGGETLGRLLVVYPWTQRFFDSFGDLSSPDAVMSNPKVKAHGKKVLNSFSEGLKNLDNLKGTFAKLSELHCDKLHVDPENFKLLGNVLVCVLAHHFGKEFTPQVQAAYQKVVAGVANALAHKYH

>Lutra lutra

VHLTGEEKAAVTSLWGKVNVDEVGGEALGRLLVVYPWTQRFFDSFGDLSSPDAVMGNPKVKAHGKKVLNSFSEGLKNLDNLKGTFAKLSELHCDKLHVDPENFKLLGNVLVCVLAHHFGKEFTPQVQAAYQKVVAGVANALAHKYH

>Mustela lutreola

VHLTAEEKAAVTALWGKVNVDEVGGEALGRLLVVYPWTQRFFDSFGDLSSPDAVMGNPKVKAHGKKVLNSFSEGLKNLDNLKGTFAKLSELHCDKLHVDPENFKLLGNVLVCVLAHHFGKEFTPQVQAAYQKVVAGVATALAHKYH

>Martes foina

VHLTGEEKAAVTALWGKVNVDEVGGEALGRLLVVYPWTQRFFDSFGDLSSPDAVMGNPKVKAHGKKVLNSFSEGLKNLDNLKGTFAKLSELHCDKLHVDPENFKLLGNVLVCVLAHHFGKEFTPQVQAAYQKVVAGVANALAHKYH

>Pteronura brasiliensis

VHLTGEEKAAVTALWGKVNVDEVGGEALGRLLVVYPWTQRFFDSFGDLSSPDAVMGNPKVKAHGKKVLNSFSEGLKNLDNLKGTFAKLSELHCDKLHVDPENFKLLGNVLVCVLAHHFGKEFTPQVQAAYQKVVAGVANALAHKYH

>Phoca vitulina

VHLTGEEKSAVTALWGKVNVDEVGGEALGRLLVVYPWTQRFFDSFGDLSSADAIMGNPKVKAHGKKVLNSFSDGLKNLDNLKGTFAKLSELHCDKLHVDPENFKLLGNVLVCVLAHHFGKEFTPQVQAAYQKVVAGVANALAHKYH

>Arctocephalus galapagoensis

VHLTADEKAAVTALWGKVNVDEVGGEALGRLLVVYPWTQRFFDSFGDLSSPDAVMGNPKVKAHGKKVLNSFSDGLKNLDNLKGTFAKLSELHCDKLHVDPENFKLLGNVLVCVLAHHFGKEFTPQVQAAYQKVVAGVANALAHKYH

>Leptonychotes weddellii

VHLTAEEKSAVTALWGKVNVDEVGGEALGRLLVVYPWTQRFFDSFGDLSSPNAIMSNPKVKAHGKKVLNSFSDGLKNLDNLKGTFAKLSELHCDQLHVDPENFKLLGNVLVCVLAHHFGKEFTPQVQAAYQKVVAGVANALAHKYH

>Odobenus rosmarus

VHLTADEKAAVTALWGKVNVDEVGGEALGRLLVVYPWTQRFFDSFGDLSSPDAVMGNPKVKAHGKKVLNSFSDGLKNLDNLKGTFAKLSELHCDKLHVDPENFKLLGNVLVCVLAHHFGKEFTPQVQAAYQKVVAGVANALAHKYH

>Felis catus

-FLTAEEKGLVNGLWGKVNVDEVGGEALGRLLVVYPWTQRFFESFGDLSSADAIMSNAKVKAHGKKVLNSFSDGLKNIDDLKGAFAKLSELHCDKLHVDPENFRLLGNVLVCVLAHHFGHDFNPQVQAAFQKVVAGVANALAHKYH

>Lynx lynx

-FLTAEEKGLVNGLWGKVNVDEVGGEALGRLLVVYPWTQRFFQSFGDLSSADAIMGNSKVKAHGKKVLNSFSDGLKNIDDLKGAFAKLSELHCDKLHVDPENFRLLGNVLVCVLAHHFGHEFNPQVQAAFQKVVAGVANALAHKYH

>Panthera onca

-FLSAEEKGLVNGLWSKVNVDEVGGEALGRLLVVYPWTQRFFQSFGDLSSADAIMSNAKVKAHGKKVLNSFSDGLKNIDDLKGAFAKLSELHCDKLHVDPENFRLLGNVLVCVLAHHFGHEFNPQVQAAFQKVVAGVAKALAHRYH

>Panthera pardus orientalis

SFLSAEEKNLVSGLWGKVNVDEVGGEALGRLLVVYPWTQRFFQSFGDLSSADAIMSNAKVKAHGKKVLNSFSDGLKNIDDLKGAFAKLSELHCDKLHVDPENFRLLGNVLVCVLAHHFGHEFNPQVQAAFQKVVAGVASALAHRYH

>Panthera pardus saxicolor

SFLSAEEKGLVNGLWSKVNVDEVGGEALGRLLVVYPWTQRFFQSFGDLSSADAIMSNAKVKAHGKKVLNSFSDGLKNIDDLKGAFAKLSELHCDKLHVDPENFRLLGNVLVCVLAHHFGHEFNPQVQAAFQKVVAGVASALAHRYH

>Panthera leo

SFLSAEEKGLVNGLWSKVNVDEVGGEALGRLLVVYPWTQRFFQSFGDLSSADAIMSNAKVKAHGKKVLNSFSDGLKNIDDLKGAFAKLSELHCDKLHVDPENFRLLGNVLVCVLAHHFGHEFNPQVQAAFQKVVAGVASALAHKYH

>Panthera tigris altaica

SFLSAEEKGLVNGLWSKVNVDEVGGEALGRLLVVYPWTQRFFQSFGDLSSADAIMGNSKVKAHGKKVLNSFSDGLKNIDDLKGAFAKLSELHCDKLHVDPENFRLLGNVLVCVLAHHFGHEFNPQVQAAFQKVVAGVASALAHKYH

>Ailuropoda melanoleuca

VHLTGEEKAAVTGLWSKVNVDEVGGEALGRLLVVYPWTQRFFDSFGDLSTPDAVMNNPKVKAHGKKVLNSFSEGLKNLDNLKGTFAKLSELHCDKLHVDPENFKLLGNVLVCVLAHHFGKEFTPQVQAAYQKVVAGVANALAHKYH

>Ailurus fulgens

VHLTGEEKAAVTGLWSKVNVDEVGGEALGRLLVVYPWTQRFFDSFGDLSSPDAVMGNPKVKAHGKKVLNSFSEGLKNLDNLKGTFAKLSELHCDKLHVDPENFKLLGNVLVCVLAHHFGKEFTPQVQAAYQKVVAGVANALAHKYH

>Ursus maritimus

VHLTGEEKSLVTGLWGKVNVDEVGGEALGRLLVVYPWTQRFFDSFGDLSSADAIMNNPKVKAHGKKVLNSFSDGLKNLDNLKGTFAKLSELHCDKLHVDPENFKLLGNVLVCVLAHHFGKEFTPQVQAAYQKVVAGVANALAHKYH

>Paguma larvata

GFLTAEEKGLVNGLWGKVNVDEVGGEALGRLLVVYPWTQRFFQSFGDLSSADAIMHNSKVKAHGKKVLNSFSDGLKHVDDLKGTFAKLSELHCDKLHVDPENFKLLGNVLVCVLAHHFGKEFTPQVQAAYQKVVAGVASALAHRYH

>Nasua nasua

VHLTGEEKTAVTNLWAKVNVDEVGGEALGRLLVVYPWTQRFFESFGDLSSPDAIMGNPKVKAHGKKVLNSFSEGLKNLDNLKGTFAKLSELHCDKLHVDPENFRLLGNVLVCVLAHHFGKEFTPQVQAAYQKVVAGVANALAHKYH

>Procyon lotor

VHLTADEKTAVTTLWGKVNVEEVGGEALGRLLVVYPWTQRFFESFGDLSSADAIMGNPKVKAHGKKVLNSFSEGLKNLDNLKGTFAKLSELHCDKLHVDPENFRLLGNVLVCVLAHHFGKEFTPPVQAAYQKVVAGVANALAHKYH

>Mellivora capensis

VHLTAEEKAAVTALWGKVNVDEVGGEALGRLLVVYPWTQRFFDSFGDLSSPDAVMGNPKVKAHGKKVLNSFSEGLKNLDNLKGTFAKLSELHCDKLHVDPENFKLLGNVLVCVLAHHFGKEFTPQVQAAYQKVVAGVANALAHKYH

>Canis lupus familiaris

VHLTAEEKSLVSGLWGKVNVDEVGGEALGRLLIVYPWTQRFFDSFGDLSTPDAVMSNAKVKAHGKKVLNSFSDGLKNLDNLKGTFAKLSELHCDKLHVDPENFKLLGNVLVCVLAHHFGKEFTPQVQAAYQKVVAGVANALAHKYH

>Chrysocyon brachyurus

VHLTAEEKSLVSGLWGKVNVDEVGGEALGRLLIVYPWTQRFFDSFGDLSTPDAVMSNAKVKAHGKKVLNSFSDGLKNLDNLKGTFAKLSELHCDKLHVDPENFKLLGNVLVCVLAHHFGKEFTPQVQAAYQKVVAGVANALAHKYH

>Cerdocyon thous

VHLTAEEKSLVSGLWAKVNVDEVGGEALGRLLIVYPWTQRFFDSFGDLSTPDSVMSNAKVKAHGKKVLNSFSDGLKNLDNLKGTFAKLSELHCDKLHVDPENFKLLGNVLVCVLAHHFGKEFTPQVQAAYQKVVAGVANALAHKYH

>Vulpes vulpes

VHLTAEEKSLVTGLWGKVNVDEVGGEALGRLLIVYPWTQRFFDSFGDLSTPDAVMGNAKVKAHGKKVLNSFSDGLKNLDNLKGTFAKLSELHCDKLHVDPENFKLLGNVLVCVLAHHFGKEFTPQVQAAYQKVVAGVANALAHKYH

>Proteles cristatus

VHLTAEEKSLVNDLWSKVNVDEVGGEALGRLLVVYPWTQRFFQSFGDLSSADAIMGNGKVKAHGKKVLNSFSDGLKHIDDLKGTFAKLSELHCDKLHVDPENFKLLGNVLVCVLAHHFGNEFTPPVQAAYQKVVAGVANALAHKYH

>Crocuta crocuta

GFLTAEEKSLVNDLWSKVNVDEVGGEALGRLLVVYPWTQRFFQSFGDLSSADAIMGNSKVKAHGKKVLNSFSDGLKHIDDLKGTFAKLSELHCDKLHVDPENFKLLGNVLVCVLAHHFGNEFTPPVQAAYQKVVAGVANALAHKYH

>Ochotona princeps

VHLSAEEKAAVTALWGKVNVDEVGGETLGRLLVVYPWTQRFFETFGDLSSASAVMGNAKVKTHGKKVMNAFSEGLHHLDNLKGTFAKLSELHCDKLHVDPENFKLLGNVLVVVLSHHFGGEFTPQVQAAWQKVVSGVANALAHKYH

>Ochotona collaris

VHLSGEEKAAVTALWGKVNVDEVGGETLGRLLVVYPWTQRFFETFGDLSSASAVMGNPKVKAHGKKVMNAFSEGLHHLDNLKGTFAKLSELHCDKLHVDPENFKLLGNVLVVVLSHHFGGEFSPQAQAAWQKVVSGVANALAHKYH

>Ochotona hyperborea

VHLTGEEKAAVTALWGKVDVDEVGGETLGRLLVVYPWTQRFFDSFGDLSSASAVMGNAKVKAHGKKVMNAFSEGLHHLDNLKGTFAKLSELHCDKLHVDPENFKLLGNVLVVVLSHHFGKEFTPQAQAAWQRVVSGVANALAHKYH

>Ochotona dauurica

VHLTGEEKAVVTALWGKVDVDEVGGETLGRLLVVYPWTQRFFDSFGDLSSASAVMGNAKVKAHGKKVMNAFSEGLHHLDNLKGTFAKLSELHCDKLHVDPENFKLLGNVLVVVLSHHFGQEFTPQAQAAWQKVVSGVANALAHKYH

>Ochotona curzoniae

VHLSGEEKSAVLSLWGKVNVDEVGGETLGRLLVVFPWTQRFFDSFGDLSSPDAVMGNSKVKAHGKKVMNAFSEGLHHLDSLKGTFAKLSELHCDKLHVDPENFKLLGNVLVVVLSHHFGAEFTPQMQAAWQKVVSGVANALAHKYH

>Ochotona ladacensis

VHLTGEEKAAVTSLWGKVNVDDVGGETLGRLLVVYPWTQRFFDTFGDLSSPSAVMGNTKVKAHGKKVMNAFSEGLHHLDSLKGTFAKLSELHCDKLHVDPENFKLLGNVLVVVLSHHFGGEFSPQVQAAWQKVVSGVANALAHKYH

>Ochotona rufescens

VHLTSEEKATVTALWAKVDVDEVGAETLGRLLVVYPWTQRFFDSFGDLSNATAVMNNAKVKAHGKKVMNAFSEGLHHLDSLKGTFAKLSELHCDKLHVDPENFKLLGNVLVVVLSHHFGGEFSPQAQAAWQKVVSGVANALAHKYH

>Eospalax baileyi

VHLSGEEKAAVISLWGKVNVDEVGGETLGRLLVVYPWTQRFFESFGDLSFAFAIMGNPKVSAHGKKVLNSFSDGLKHLDNLKGTFSHLSELHCDKLHVDPENFKLLGNVIVIVLAHHLGKDFTPAAQAAFQKVVAGVATALAHKYH

>Microtus oeconomus

VHLSGEEKAAVFSLWGKVNVDEVGGETLGRLLVVYPWTQRFFDSFGDLSNATAVMGNAKVKAHGKKVMNAFSEGLHHLDSLKGTFAKLSELHCDKLHVDPENFKLLGNVLVVVLSHHFGGEFSPQVQAAWQKVVSGVANALAHKYH

>Microtus pennsylvanicus

VHLTDAEKAALSGLWGKANADAVGAEALGRLLVVYPWTQRFFEHFGDLSSASAVMGNPKVKAHGKKVLHAFADGLKHLDNLKGTFSALSELHCDKLHVDPENFRLLGNMLVLVLSHDLGKDFTPAAQAAFQKVVAGVASALAHKYH

>Microtus ochrogaster

VHLTDAEKAAISGLWGKVNADGVGAEALGRLLVVYPWTQRFFEHFGDLSSASAVMGNAQVKAHGKKVIHAFADGLKHLDNLKGTFSSLSELHCDKLHVDPENFRLLGNMIVIVLSHDLGKDFTPAAQAAFQKVVAGVASALAHKYH

>Tamias merriami

VHLTAEEKSAVAALWGKVNTDEVGGEALGRLLVVYPWTQRFFDSFGDLSSASAVMSNPKVKAHGKKVFDSFSNGLKHLDNLKGTFASLSELHCDKLHVDPENFKLLGNVLVVVLAHHLGKEFTPQVQSAFQKVVTGVANALAHKYH

>Tamias striatus

VHLTADEKVSLSSLWGKVNPDELGGEALGRLLLVYPWTQRFFDSFGDLSSAVAVMGNAKVKAHGKKVLDSFSDGLKHLDNLKGTFASLSELHCDKLHVDPENFKLLGNVLVLVLAHHLGKEFTPQAQGTFQKVVAGVANALAHKYH

>Callospermophilus lateralis

VHLSDGEKAAINAAWGKVNADEVGGEALGRLLVVYPWTQRFFDSFGDLSSASAVIGNPKVKAHGKKVIDSFSNGLKHLDNLKGTFASLSELHCDKLHVDPENFRLLGNVIVIVLAHHLGKDFTPQVQAAFQKVVTGVANALSHKYH

>Ctenodactylus gundi

VHLSAEEKAAVTGLWGKVNVEEVGGEALGRLLVVYPWTQRFFESFGDLSSAAAVMGNPKVKAHGKKVLTSFSEGLSHLDNLKGTFAKLSELHCDKLHVDPENFRLLGNMIVITLAHHYGPEFGPQTQAAFQKVVAGVANALAHKYH

>Mus musculus

VHLTDAEKAAVSCLWGKVNADEVGGEALGRLLVVYPWTQRYFSSFGDLSSADAIMGNAKVKAHGKKVITAFSDGLNHLDNLKGTFASLSELHCDKLHVDPENFRLLGNMIVIVLGHHLGKDFTPAAQAAFQKVVAGVAAALAHKYH

>Rattus norvegicus

VHLTDAEKAAVNALWGKVNPDDVGGEALGRLLVVYPWTQRYFDSFGDLSSASAIMGNPKVKAHGKKVINAFNDGLKHLDNLKGTFAHLSELHCDKLHVDPENFRLLGNMIVIVLGHHLGKEFTPCAQAAFQKVVAGVASALAHKYH

>Mesocricetus auratus

VHLTDAEKALVTGLWGKVNADAVGAEALGRLLVVYPWTQRFFEHFGDLSSASAVMNNPQVKAHGKKVIHSFADGLKHLDNLKGAFSSLSELHCDKLHVDPENFKLLGNMIIIVLSHDLGKDFTPSAQSAFHKVVAGVANALAHKYH

>Cricetulus griseus

VHLTDAEKALVTGLWGKVNADAVGAEALGRLLVVYPWTQRFFEHFGDLSLPVAVMNNPQVKAHGKKVIHSFADGLKHLDNLKGAFSSLSELHCDKLHVDPENFKLLGNMIIIVLIHDLGKDFTPSAQSAFHKVVAGVANALAHKYH

>Peromyscus maniculatus

VHLTDAEKALVTGLWGKVKPEEIGGEALGRLLAVYPWTQRFFDSFGDLSSASAIMGNAKVKAHGKKVIDSFGEGLKHLDNLKGTFASLSELHCDKLHVDPENFKLLGNMIVIVMAHHLGKDFTPAAQAAYQKVVAGVATALAHKYH

>Peromyscus californicus

VHLTDAEKALVTGLWGKVKPDELGGEALGRLLVGYPWTQRFFDSFGDLSSASALMGNPKVKAHGKKVLDSFSEGLKHLDNLKGTFASLSELHCDKLHVDPENFKLLGNMLVLVLAHLLGKDFTPAAQAAYQKVVAGVATALAHKYH

>Peromyscus leucopus

VHLTDAEKALVTGLWGKVKPEEIGGEALGRLLAVYPWTQRFFDSFGDLSSASAIMGNAKVKAHGKKVIDSFGEGLKHLDNLKGTFASLSELHCDKLHVDPENFKLLGNMIVIVMAHHLGKDFTPAAQSAYQKVVAGVATALAHKYH

>Heterocephalus glaber

VHLSNEEKAAVTSLWGKVNVEETGGEALGRLLVVYPWTQRFFEHFGDLSSPSAIMGNPKVKAHGAKVLSSFSEALNHLDNLKGTFAKLSELHCDKLHVDPENFRLLGDVIVIVLAQHHGHDLTPTVQAAFQKVVAGVAHALGHKYH

>Nannospalax ehrenbergi

VHLTDAEKAAVSGLWSKVNVDEIGGEALGRLLVVYPWTQRFFDSFGDLSSPSAVMSNPKVKAHGKKVLNSFSEGLKHLDNLKGTFSSLSELHCDKLHVDPENFKLLGNVIVVVLAHHLGKDFTPAAQAAFQKVVAGVATALAHKYH

>Ondatra zibethicus

VHLTDAEKAAISGLWGKVNADGVGAEALGRLLVVYPWTQRFFEHFGDLSSSSAVMGNAKVKSHGKKVITAFADGLKHLDNLKGTFSALSELHCDKLHVDPENFKLLGNMIVIVLSHDLGKDFTPDAQSAFQKVVTGVATALGHKYH

>Octodon degus

VHLTGEEKAAVTGLWAKVNVEEIGGEALGRLLVVYPWTQRFFDSFGDLSSASAIMGNPKVKAHGAKVLHSFSEGLNHLDNLKGTFAKLSELHCDKLHVDPENFRLLGNMIVIALAHHHGAEFTPPVQAAFQKVVAGVANALAHKYH

>Oryctolagus cuniculus

VHLSSEEKSAVTALWGKVNVEEVGGEALGRLLVVYPWTQRFFESFGDLSSANAVMNNPKVKAHGKKVLAAFSEGLSHLDNLKGTFAKLSELHCDKLHVDPENFRLLGNVLVIVLSHHFGKEFTPQVQAAYQKVVAGVANALAHKYH

>Lepus europaeus

VHLSGEEKSAVTALWGKVNVEEVGGETLGRLLVVYPWTQRFFESFGDLSTASAVMGNPKVKAHGKKVLAAFSEGLSHLDNLKGTFAKLSELHCDKLHVDPENFRLLGNVLVIVLSHHFGKEFTPQVQAAYQKVVAGVANALAHKYH

>Marmota marmota marmota

VHLSDGEKNAISTAWGKVNAADIGAEALGRLLVVYPWTQRFFDSFGDLSSASAVMGNAKVKAHGKKVIDSFSNGLKHLDNLKGTFASLSELHCDKLHVDPENFKLLGNMIVIVMAHHLGKDFTPEAQAAFQKVVAGVANALAHKYH

>Macrotus californicus

VHLTGEEKSTVSALWGKVNVEEIGGEALGRLLVVYPWTQRFFDSFGDLSSPSAVFGNAKVKSHGKKVLDSFSNGMQHLDNLKGTFAKLSELHCDKLHVDPENFRLLGNVLVVVLARNFGKEFTPQVQAAYQKVVAGVATALAHKYH

>Pteropus alecto

VHLSGEEKAAVTGLWGKVKVDEVGGEALGRLLVVYPWTQRFFDSFGDLSSASAVMGNPKVKAHGKKVLDSFSEGLQHLDNLKGTFAKLSELHCDKLHVDPENFRLLGNVLVCVLARHFGKEFTPQVQAAYQKVVAGVANALAHKYH

>Antrozous pallidus

VHLTADEKSAVTGLWGKVNVEEVGGEALGRLLVVYPWTQRFFESFGDLSNAGAVMGNAKVKAHGKKVLNAFSDGLKNLDNLKGTFAKLSELHCDKLHVDPENFRLLGNVLMIVLARHFGKEFCPPVQAAFQKVSLGVATALGHKYH

>Tadarida brasiliensis

VHLSGEEKGAVTALWGKVNQEEVGGEALGRLLVVYPWTQRFFDSFGDLSSASAVMGNAKVKAHGKKVLNSFSDGLKNLDNLKGAFAKLSELHCDKLHVDPENFKLLGNVLVVVLARTFGKEFTPPVQSAFQKVAAGVATALAHKYH

>Rousettus aegyptiacus

VHLSGEEKAAVTALWGKVKVEEVGGEALGRLLVVYPWTQRFFDSFGDLSSASAVMSNPKVKAHGKKVLDSFSEGLQHLDSLKGTFAKLSELHCDKLHVDPENFRLLGNVLVCVLARHFGKEFTPQVQAAYQKVVAGVATALAHKYH

>Cynopterus sphinx

VHLSGEEKSAVTSLWGKVKVDEVGGEALGRLLVVYPWTQRFFDSFGDLSSASAVMGNAKVKAHGKKVLDSFSEGLQHLDSLKGTFAKLSELHCDKLHVDPENFRLLGNVLVVVLARHFGKEFTPQLQAAYQKVVAGVATALAHKYH

>Taphozous georgianus

VHLTADEKAAVTGLWGKVNVDEVGGEALGRLLVVYPWTQRFFDSFGDLSAASAVMGNPKVKAHGKKVLNSFSDGLKNLDNLKGTYAKLSELHCDKLHVDPENFRLLGNVLVCVLARHFGKEFTPQVQAAYQKVVSGVATALAHKYH

>Myotis brandtii

VHLTADEKAAVSGLWGKVNVDEVGGEALGRLLVVYPWTQRFFTSFGDLSNAAAVMGNSKVKAHGKKVLNSFGEGLKNLDNLKGTFASLSELHCDKLHVDPENFKLLGNVLVIVLARHFGKEFTPQVQGAFQKLALGVATALAHKYH

>Chalinolobus morio

VHLTGEEKAAVTGLWGKVNVDEVGGEALGRLLVVYPWTQRFFDSFGDLSNAGAVMGNAKVKAHGKKVLNAFGEGLKNLDNLKGTFAKLSELHCDKLHVDPENFRLLGNVLVVVLARHFGKDFTPPVQAAFQKLALGVATALAHKYH

>Myotis velifer

VHLTADEKAAVSGLWGKVNVDEVGGEALGRLLVVYPWTQRFFTSFGDLSNAAAVMGNSKVKAHGKKVLNSFGEGLKNVDNLKGTFASLSELHCDKLHVDPENFRLLGNVLVIVLARHFGKEFTPQVQGAFQKLALGVATALAHKYH

>Macroderma gigas

VHLTGEEKAAVTGLWGKVNVEEVGGEALGRLLVVYPWTQRFFDSFGDLSSPSAVMGNPKVKAHGKKVLNSFSDGLKNLDNLKGTFAKLSELHCDKLHVDPENFRLLGNVLVCVLARHFGKEFTPQVQAAYQKVVAGVATALAHKYH

>Rhinolophus ferrumequinum

VHLTGEEKGIVTGLWGKVNVDEVGGEALGRLLVVYPWTQRFFDSFGDLSSAAAVMGNAKVKAHGKKVLDSFSEGLKNLDNLKGTFAKLSELHCDKLHVDPENFRLLGNVLVCVLARNFGKEFTPQVQAAYQKVVVGVATALAHKYH

>Pteropus Giganteus

VHLSGEEKAAVTGLWGKVKVDEVGGEALGRLLVVYPWTQRFFDSFGDLSSASAVMGNPKVKAHGKKVLDSFSEGLQHLDNLKGTFAKLSELHCDKLHVDPENFRLLGNVLVCVLARHFGKEFTPQVQAAYQKVVAGVANALAHKYH

>Pteropus poliocephalus

VHLSGEEKAAVTGLWGKVKVDEVGGEALGRLLVVYPWTQRFFDSFGDLSSAPAVMGNPKVKAHGKKVLDSFSEGLQHLDNLKGTFAKLSELHCDKLHVDPENFRLLGNVLVCVLARHFGKEFTPQVQAAYQKVVAGVANALAHKYH

>Myotis lucifugus

VHLTADEKAAVSGLWGKVNVDEVGGEALGRLLVVYPWTQRFFTSFGDLSNAAAVMGNSKVKAHGKKVLNSFGEGLKNVDNLKGTFASLSELHCDKLHVDPENFKLLGNVLVVVLARHFGKEFTPQVQGAFQKLALGVATALAHKYH

>Megaderma lyra

VHLTNEEKTAVIGLWGKVNVEEVGGEALGRLLVVYPWTQRFFESFGDLSSPSAIMGNPKVKAHGKKVLNSFSEGLKNLDNLKGTFAKLSELHCDKLHVDPENFRLLGYILLCVLARHFGKEFTPQVQAAYQKVVAGVATALAHKYH

>Bradypus tridactylus

VHLADDEKAAVSALWHKVHVEEFGGEALGRLLVVYPWTSRFFESFGDLSSADAVFSNAKVKAHGKKVLTSFGEGLKHLDDLKGTYAHLSELHCDKLHVDPENFKLLGNVLVIVLARHFGKEFTPQLQAAYQKVTTGVSTALAHKYH

>Dasypus novemcinctus

VNLTSDEKTAVLALWNKVDVEDCGGEALGRLLVVYPWTQRFFESFGDLSTPAAVFANAKVKAHGKKVLTSFGEGMNHLDNLKGTFAKLSELHCDKLHVDPENFKLLGNMLVVVLARHFGKEFDWHMHACFQKVVAGVANALAHKYH

>Loxodonta africana

VNLTAAEKTQVTNLWGKVNVKELGGEALSRLLVVYPWTRRFFEHFGDLSTAEAVLHNAKVLAHGEKVLTSFGEGLKHLDNLKGTFADLSELHCDKLHVDPENFRLLGNVLVIVLARHFGKEFTPDVQAAYEKVVAGVANALAHKYH

>Elephas maximus

VNLTAAEKTQVTNLWGKVNVKELGGEALSRLLVVYPWTRRFFEHFGDLSTADAVLHNAKVLAHGEKVLTSFGEGLKHLDNLKGTFADLSELHCDKLHVDPENFRLLGNVLVIVLARHFGKEFTPDVQAAYEKVVAGVANALAHKYH

>Mammuthus primigenius

VNLTAAEKTQVANLWGKVNVKELGGEALSRLLVVYPWTRRFFEHFGDLSTADAVLHNAKVLAHGEKVLTSFGEGLKHLDNLKGTFSDLSELHCDKLHVDPQNFRLLGNVLVIVLARHFGKEFTPDVQAAYEKVVAGVANALAHKYH

>Orycteropus afer

V-LTADEKALVSSLWCKMNVDEAGAEALGRMLVVYPWTQRFFDHFGDLSSASAVMGNAKVKAHGKKVLNSFSDGLKHLDDLKGTFAHLSELHCDKLHVDPENFRLLGNVLVCVMARHLGPEFTPQAQAAYQKVVAGVANALAHKYH

>Procavia capensis

VHLTDAEKAAVTGLWGKVKVDEYGGEALGRLLVVYPWTQRFFEHFGDLSNADAIMHNPKVLAHGKKVLSSFGDGLNHLDNLKGTFAQLSELHCDKLHVDPENFRLLGNVLVVVLARHFHEEFTPDVQAAFQKVVTGVANALAHKYH

>Trichechus inunguis

VHLTPEEKALVIGLWAKVNVKEYGGEALGRLLVVYPWTQRFFEHFGDLSSASAIMNNPKVKAHGEKVFTSFGDGLKHLEDLKGAFAELSELHCDKLHVDPENFRLLGNVLVCVLARHFGKEFSPEAQAAYQKVVAGVANALAHKYH

>Elephantulus edwardii

VHLSDAEKALVNGIWSKVDVDKLGGQALGCLLIVYPWTQRFFDSFGDLSSADAIIKNPKVAAHGKKVVNSFSEGMKHLDDLKGTFAQLSELHCDKLHVDPENFRLLGNVIVRVIARHLGSEFTPQAQAAFQKVVTGVANALAHKYH

>Echinops telfairi

VHMTDAEKKLVTTMWGKLDVDAAGAETLGRVLVVYPWTQRFFGHFGDLSSACAVMDNPKVQAHGKKVLHSLGDGLNHLDDLKHFYAALSELHCDKLHVDPENFRLLGNVLVCVMSRHFGAEFTPQVQAAYQKVVAGVANALAHKYH

>Chrysochloris asiatica

VHLTAGEKSLVASTWSKVNVDEVGGEVLGRLLVVYPWTQRFFTSFGDLSSASAIMGNAKVQAHGKKVLKSFAEGLNHLDDLKGAFAQLSELHCDKLHVDPENFRLLGNVLMCVMARHLRADFTPEAQAACQKVVAGVATALAHKYH

>Tupaia chinensis

VHLSGEEKAAVTGLWGKVDLEKVGGQSLGSLLIVYPWTQRFFDSFGDLSSPSAVMSNAKVKAHGKKVLTSFSDGLNHLDNLKGTFAKLSELHCDKLHVDPENFRLLGNVLVRVLACNFGPEFTPQVQAAFQKVVAGVANALAHKYH

>Tupaia glis

VHLSGEEKAAVTGLWGKVDLEKVGGQSLGSLLIVYPWTQRFFDSFGDLSSPSAVMSNPKVKAHGKKVLTSFSDGLNHLDNLKGTFAKLSELHCDKLHVDPENFRLLGNVLVRVLACNFGPEFTPQVQAAFQKVVAGVANALAHKYH

>Blarina brevicauda

VHLTAEEKSLVTGLWGKVNVEEAGGEALGRLLVVYPWTQRFFDSFGDLSSASAVMGNPKVKAHGKKVLQSMGDGLANLDNLKGTFAKLSELHCDKLHVDPENFRLLGNVLVVVLARHFGKEFTPPVQAAFQKVVAGVATALAHKYH

>Sorex araneus

VHLSGEEKGVVTGLWGKVNVDEVGGEALGRLLVVYPWTQRFFDSFGDLSSASAIMGNPKVKAHGKKVLQSLGDGINNLDNLKGTFAKLSELHCDKLHVDPENFRLLGNVLVCVLARHFGKEFTPSVQAAFQKMVAGVAAALAHKYH

>Scapanus orarius

VHLSAEEKGLVTGLWGKVNVDDVGAEALGRLLVVYPWTQRFFDSFGDLSSAGAIMGNPKVKAHGKKVANSISDGIKNLDNLKGTYAKLSELHCDKLHVDPENFRLLGNVLVCVMARTLGKEFTPHAQAAFQKMVLGVATALAHKYH

>Talpa europaea

VHLSGEEKGLVTGMWGKVNVDEVGGEALGRLLVVYPWTQRFFDSFGDLSSASAIMGNAKVKAHGKKVANSITDGVKNLDNLKGTYAKLSELHCDKLHVDPENFRLLGNVLVCVLARNLGKEFTPQAQAAFQKVVLGVATALAHKYH

>Suncus murinus

VHLSGEEKACVTGLWGKVNEDEVGAEALGRLLVVYPWTQRFFDSFGDLSSASAVMGNPKVKAHGKKVLHSLGEGVANLDNLKGTFAKLSELHCDKLHVDPENFRLLGNVLVVVLASKFGKEFTPPVQAAFQKVVAGVANALAHKYH

>Condylura cristata

VHLSGEEKGLVTSMWGKVNVDEVGGEALGRLLVVYPWTQRFFDSFGDLSSASAIMGNAKVKAHGKKVAHSITDGIKNLDNLKGTYAKLSELHCDKLHVDPENFRLLGNVLVCVLARNLGKEFTPQAQAAFQKVVLGVATALAHKYH

>Episoriculus fumidus

VHMSGEEKSAITALWGKVNVDDVGGEALGRLLVVYPWTQRFFDSFGDLSSASAVMGNPKVKAHGKKVLMSMADGVNNMDNLKGTFAKLSELHCDKLHVDPENFRLLGNVLVVVLARHFGKEFTPTVQAAFQKLVAGVATALAHKYH

>Scalopus aquaticus

VHMSAEEKGIVTSMWGKVNVDDIGAEALGRLLVVYPWTQRFFDSFGDLSSPAAIMGNPKVKAHGKKVAHSISDGIKNLDNLKGTYAKLSELHCDKLHVDPENFRLLGNVLVCVLARNLGKEFTPHARTAFQKMVLEVAAALAHKYH

>Erinaceus europaeus

VHLTAEEKALVTGLWGKVKVEEFGGEALGRLLVVYPWTQRFFDSFGDLSSADAVMGNPKVKAHGAKVLQSMGDGIKNLDNLKGTFSKLSELHCDKLHVDPENFRLLGNVLVCVLARHFGKDFTPAAQAAFQKVVAGVANALAAKYH

>Macropus giganteus

VHLTAEEKNAITSLWGKVAIEQTGGEALGRLLIVYPWTSRFFDHFGDLSNAKAVMANPKVLAHGAKVLVAFGDAIKNLDNLKGTFAKLSELHCDKLHVDPENFKLLGNIIVICLAEHFGKEFTIDTQVAWQKLVAGVANALAHKYH

>Macropus eugenii

VHLTAEEKNAITSLWGKVAIEQTGGEALGRLLIVYPWTSRFFDHFGDLSNAKAVMSNPKVLAHGAKVLVAFGDAIKNLDNLKGTFAKLSELHCDKLHVDPENFKLLGNIIVICLAEHFGKEFTIDAQVAWQKLVAGVANALAHKYH

>Sarcophilus harrisii

VHLSGEEKGYINAIWSKVSIDQTGAEALGRLLIVYPWTSRFFDHFGDLSSAKSVMGNAKVQGHGAKVLTSFGDAVKNMDNLKGTFAKLSELHCDKLHVDPENFKLLGNILVICLAEHFGKEFTPEVQAAWQKLVAGVATALAHKYH

>Didelphis virginiana

VHLTSEEKNCITTIWSKVQVDQTGGEALGRMLVVYPWTTRFFGSFGDLSSPGAVMSNSKVQAHGAKVLTSFGEAVKHLDNLKGTYAKLSELHCDKLHVDPENFKMLGNIIVICLAEHFGKDFTPECQVAWQKLVAGVAHALAHKYH

>Monodelphis domestica

VHLTPEEKNCITSLWSKVAVDQTGGEALGRMLVVYPWTTRFFGSFGDLSSAGAVMSNAKVQAHGAKVLTSFGEAVKHLDNLKGTYAKLSELHCDKLHVDPENFKMLGNIIVICLAEHFGKEFTPECQVAWQKLVAGVAHALAHKYH

>Ornithorhynchus anatinus

VHLSGGEKSAVTNLWGKVNINELGGEALGRLLVVYPWTQRFFEAFGDLSSAGAVMGNPKVKAHGAKVLTSFGDALKNLDDLKGTFAKLSELHCDKLHVDPENFNRLGNVLIVVLARHFSKDFSPEVQAAWQKLVSGVAHALGHKYH

>Tachyglossus aculeatus

VHLSGSEKTAVTNLWGHVNVNELGGEALGRLLVVYPWTQRFFESFGDLSSADAVMGNAKVKAHGAKVLTSFGDALKNLDNLKGTFAKLSELHCDKLHVDPENFNRLGNVLVVVLARHFSKEFTPEAQAAWQKLVSGVSHALAHKYH

>Gallus gallus

VHWTAEEKQLITGLWGKVNVAECGAEALARLLIVYPWTQRFFASFGNLSSPTAILGNPMVRAHGKKVLTSFGDAVKNLDNIKNTFSQLSELHCDKLHVDPENFRLLGDILIIVLAAHFSKDFTPECQAAWQKLVRVVAHALARKYH

>Coturnix japonica

VHWSAEEKQLITGLWGKVNVAECGAEALARLLIVYPWTQRFFASFGNLSSPTAILGNPMVRAHGKKVLTSFGDAVKNLDNIKNTFSQLSELHCDKLHVDPENFRLLGDILIIVLAAHFTKDFTPECQAAWQKLVRVVAHALARKYH

>Meleagris gallopavo

VHWSAEEKQLITGLWGKVNVADCGAEALARLLIVYPWTQRFFASFGNLSSPTAILGNPMVRAHGKKVLTSFGDAVKNLDNIKNTFSQLSELHCDKLHVDPENFRLLGDILIIVLAAHFSKDFTPECQAAWQKLVRVVAHALARKYH

>Phasianus colchicus

VHWSAEEKQLITGLWGKVNVADCGAEALARLLIVYPWTQRFFASFGNLSSPTAILGNPMVRAHGKKVLTSFGDAVKNLDNIKNTFSQLSELHCDKLHVDPENFRLLGDILIIVLAAHFSKDFTPECQAAWQKLVRVVAHALARKYH

>Francolinus pondicerianus

VHWTAEEKQLITGLWGKVNVAECGAEALARLLIVYPWTQRFFASFGNLSSPTAILGNPMVRAHGKKVLTSFGDAVKNLDNIKNTFSQLSELHCDKLHVDPENFRLLGDILIIVLAAHFSKDFTPDCQAAWQKLVRVVAHALARKYH

>Struthio camelus

VQWSAEEKQLISGLWGKVNVADCGAEALARLLIVYPWTQRFFASFGNLSSPTAILGNPMVRAHGKKVLTSFGDAVKNLDNIKNTFAQLSELHCDKLHVDPENFRLLGDILIIVLAAHFTKEFTPECQAAWQKLVRVVAHALARKYH

>Rhea americana

VQWTAEEKQLITGLWGKVNVADCGAEALARLLIVYPWTQRFFASFGNLSSPTAILGNPMVRAHGKKVLTSFGDAVKNLDNIKNTFAQLSELHCDKLHVDPENFRLLGDILIIVLAAHFAKDFTPECQAAWQKLVRVVAHALARKYH

>Anas puna

VHWTAEEKQLITGLWGKVNVADCGAEALARLLIVYPWTQRFFASFGNLSSATAITGNPMVRAHGKKVLTSFGDAVKNLDNIKNTFAQLSELHCEKLHVDPENFRLLGDILIIVLAAHFTKDFTPECQAAWQKLVRVVAHALARKYH

>Anas Platyrhynchos

VHWTAEEKQLITGLWGKVNVADCGAEALARLLIVYPWTQRFFASFGNLSSPTAILGNPMVRAHGKKVLTSFGDAVKNLDNIKNTFAQLSELHCDKLHVDPENFRLLGDILIIVLAAHFTKDFTPECQAAWQKLVRVVAHALARKYH

>Anas penelope

VHWTAEEKQLITGLWGKVNVADCGAEALARLLIVYPWTQRFFASFGNLSSPTAILGNPMVRAHGKKVLTSFGDAVKNLDNIKNTFAQLSELHCDKLHVDPENFRLLGDILIIVLAAHFTKDFTPECQAAWQKLVRVVAHALARKYH

>Tadorna ferruginea

VHWTAEEKQLITGLWGKVNVADCGAEALARLLIVYPWTQRFFASFGNLSSPTAILGNPMVRAHGKKVLTSFGDAVKNLDNIKNTFAQLSELHCEKLHVDPENFRLLGDILIIVLAAHFTKDFTPDCQAAWQKLVRVVAHALARKYH

>Cyanochen cyanopterus

VHWTAEEKQLITGLWGKVNVADCGAEALARLLIVYPWTQRFFASFGNLSSPTAILGNPMVRAHGKKVLTSFGDAVKNLDNIKNTFAQLSELHCDKLHVDPENFRLLGDILIIVLASHFTKDFTPDCQAAWQKLVRVVAHALARKYH

>Cairina moschata

VHWTAEEKQLITGLWGKVNVADCGAEALARLLIVYPWTQRFFASFGNLSSPTAILGNPMVRAHGKKVLTSFGDAVKNLDNIKNTFAQLSELHCDKLHVDPENFRLLGDILIIVLAAHFTKDFTPDCQAAWQKLVRVVAHALARKYH

>Anas cyanoptera

VHWTAEEKQLITGLWGKVNVADCGAEALARLLIVYPWTQRFFASFGNLSSATAITGNPMVRAHGKKVLTSFGDAVKNLDNIKNTFAQLSELHCDKLHVDPENFRLLGDILIIVLAAHFTKDFTPECQAAWQKLVRVVAHALARKYH

>Cereopsis novaehollandiae

VHWTAEEKQLITGLWGKVNVADCGAEALARLLIVYPWTQRFFSSFGNLSSPTAILGNPMVRAHGKKVLTSFGDAVKNLDNIKNTFAQLSELHCDKLHVDPENFRLLGDILIIVLAAHFAKDFTPDCQAAWQKLVRVVAHALARKYH

>Anser anser

VHWSAEEKQLIAGLWGKVNVADCGAEALARLLIVYPWTQRFFSSFGNLSSPTAILGNPMVRAHGKKVLTSFGDAVKNLDNIKNTFAQLSELHCDKLHVDPENFRLLGDILIIVLAAHFAKEFTPECQAAWQKLVRVVAHALARKYH

>Heteronetta atricapilla

VHWTAEEKQLITGIWGKVNVADCGAEALARLLIVYPWTQRFFSSFGNLSSPTAILGNPMVRAHGKKVLTSFGDAVKNLDNIKNTFAQLSELHCDKLHVDPENFRLLGDILIIVLAAHFSKDFTPDCQAAWQKLVRVVAHALARKYH

>Branta canadensis

VHWTAEEKQLITGLWGKVNVADCGAEALARLLIVYPWTQRFFSSFGNLSSPTAILGNPMVRAHGKKVLTSFGDAVKNLDNIKNTFAQLSELHCDKLHVDPENFRLLGDILIIVLAAHFAKDFTPDCQAAWQKLVRVVAHALARKYH

>Nomonyx dominicus

VHWTAEEKQLITGLWGKVNVADCGAEALARLLIVYPWTQRFFSSFGNLSSPTAILGNPMVRAHGKKVLTSFGDAVKNLDNIKNTFAQLSELHCDKLHVDPENFRLLGDILIIVLAAHFSKDFTPDCQAAWQKLVRVVAHALARKYH

>Aythya fuligula

VHWTAEEKQIITGLWGKVNVADCGAEALARLLIVYPWTQRFFSSFGNLSSPTAILGNPMVRAHGKKVLTSFGDAVKNLDNIKNTFAQLSELHCDKLHVDPENFRLLGDILIVVLAAHFSKEFTPECQAAWQKLVRVVAHALARKYH

>Merganetta armata

VHWTAEEKQLITGLWGKVNVADCGAEALARLLIVYPWTQRFFASFGNLSSPTAILGNPMVRTHGKKVLTSFGDAVKNLDNIKNTFAQLSELHCDKLHVDPENFRLLGDILVIVLAAHFSKDFTPDCQAAWQKLVRVVAHALARKYH

>Neochen jubata

VHWTAEEKQLITGLWGKVNVADCGAEALARLLIVYPWTQRFFASFGNLSSPTAISGNPMVRAHGKKVLTSFGDAVKNLDNIKNTFAQLSELHCDKLHVDPENFRLLGDILIIVLAAHFTKDFTPDCQAAWQKLVRVVAHALARKYH

>Coscoroba coscoroba

VHWTAEEKQLITGLWGKVNVADCGAEALARLLIVYPWTQRFFSSFGNLSSPTAILGNPMVRAHGKKVLTSFGDAVKNLDNIKNTFAQLSELHCDKLHVDPENFRLLGDILIIVLAAHFTKDFTPDCQAAWQKLVRVVAHALARKYH

>Chloephaga picta

VHWTAEEKQLITGLWGKVNVADCGAEALARLLIVYPWTQRFFASFGNLSSPTAISGNPMVRAHGKKVLTSFGDAVKNLDNIKNTFAQLSELHCDKLHVDPENFRLLGDILIIVLAAHFTKDFTPDCQAAWQKLVRVVAHALARKYH

>Biziura lobata

VHWTAEEKQLITGLWGKVNVADCGAEALARLLIVYPWTQRFFASFGNLSSPTAILGNPMVRAHGKKVLTSFGDAVKNLDNIKNTFAQLSELHCDKLHVDPENFRLLGDILIIVLAAHFAKDFTPDCQAAWQKLVRVVAHALARKYH

>Anser indicus

VHWTAEEKQLITGLWGKVNVADCGAEALARLLIVYPWTQRFFSSFGNLSSPTAILGNPMVRAHGKKVLTSFGDAVKNLDNIKNTFAQLSELHCDKLHVDPENFRLLGDILIIVLAAHFAKEFTPDCQAAWQKLVRVVAHALARKYH

>Chloephaga melanoptera

VHWTAEEKQLITGLWGKVNVADCGAEALARLLIVYPWTQRFFASFGNLSSPTAISGNPMVRAHGKKVLTSFGDAVKNLDNIKNTFSQLSELHCDKLHVDPENFRLLGDILIIVLAAHFTKDFTPDCQAAWQKLVRVVAHALARKYH

>Cygnus atratus

VHWTAEEKQLITGLWGKVNVADCGAEALARLLIVYPWTQRFFSSFGNLSSPTAILGNPMVRAHGKKVLTSFGDAVKNLDNIKNTFAQLSELHCDKLHVDPENFRLLGDILIIVLAAHFAKDFTPDCQAAWQKLVRVVAHALARKYH

>Cygnus buccinator

VHWTAEEKQLITGLWGKVNVADCGAEALARLLIVYPWTQRFFSSFGNLSSPTAILGNPMVRAHGKKVLTSFGDAVKNLDNIKNTFAQLSELHCDKLHVDPENFRLLGDILIIVLAAHFAKDFTPDCQAAWQKLVRVVAHALARKYH

>Stictonetta naevosa

VHWTAEEKQLITGLWGKVNVADCGAEALARLLIVYPWTQRFFASFGNLSSPTAISGNPMVRAHGKKVLTSFGDAVKNLDNIKNTFAQLSELHCDKLHVDPENFRLLGDILIIVLASHFSKDFTPDCQAAWQKLVRVVAHALARKYH

>Dendrocygna viduata

VHWSAEEKQLITGLWGKVNVADCGAEALARLLIVYPWTQRFFASFGNLSSATAISGNPMVRAHGKKVLTSFGEAVKNLDNIKNTFAQLSELHCDKLHVDPENFRLLGDILIIVLAAHFTKDFTPECQAAWQKLVRVVAHALARKYH

>Anseranas semipalmata

VHWSAEEKQLITGLWGKVNVADCGAEALARLLIVYPWTERFFSSFGNLSSPTAIIGNPMVRAHGKKVLTSFGEAVKNLDNIKNTFAQLSELHCDKLHVDPENFRLLGDILIIVLAAHFSKDFTPDCQAAWQKLVRVVAHALARKYH

>Eudyptes chrysocome

VHWSAEEKQLITGLWGKVNVAQCGGEALARLLIVYPWTQRFFSSFGNLSSPSAILGNPMVRAHGKKVLTSFGDAVKNMDNIKNTFAQLSELHCDKLHVDPENFRLLGDILIIVLAAHFAKDFTPECQAAWEKLVRVVAHALARKYH

>Aptenodytes forsteri

VHWSAEEKQLITGLWGKVNVAECGAEALARLLIVYPWTQRFFASFGNLSSPAAVLANPMVRAHGKKVLTSFGDAVKNMDNIKNTFAQLSELHCDKLHVDPENFRLLGDILIIVLAAHFSKDFTPDCQAAAQKLVRVVAHALARKYH

>Ara ararauna

VHWTAEEKQLITGLWGKVNVADCGAEALARLLIVYPWTQRFFASFGNLSSPTAILGNPMVRAHGKKVLTSFGEAVKNLDNIKNTFAQLSELHCDKLHVDPENFRLLGDILIIVLAAHFGKDFTPECQAALQKLVRVVAHALARKYH

>Psittacula krameri

VHWSAEEKQLITGLWGKVNVAECGAEALARLLIVYPWTQRFFTSFGNLSSASAVLGNPNVRAHGKKVLTSFGEAVKNLDNIKNTFAQLSELHCDKLHVDPENFRLLGDILIIVLAGHFGKDFTPDCQAAWQKLVRAVAHALARKYH

>Passer montanus

VQWTAEEKQLITGLWGKVNVAECGGEALARLLIVYPWTQRFFASFGNLSSPTAVLGNPKVQAHGKKVLTSFGEAVKNLDSIKNTFSQLSELHCDKLHVDPENFRLLGDILVVVLAAHFGKDFTPDCQAAWQKLVRVVAHALARKYH

>Taeniopygia guttata

VQWTAEEKQLITGLWGKVNVAECGGEALARLLIVYPWTQRFFASFGNLSSPTAVLGNPKVQAHGKKVLTSFGEAVKNLDSIKNTFSQLSELHCDKLHVDPENFRLLGDILVVVLAAHFGKDFTPDCQAAWQKLVRVVAHALARKYH

>Sturnus vulgaris

VQWTAEEKQLITGLWGKVNVAECGAEALARLLIVYPWTQRFFASFGNLSSPTAVLGNPKVQAHGKKVLTSFGDAVKNLDSIKNTFSQLSELHCDKLHVDPENFRLLGDILVVVLAAHFGKDFTPDCQAAWQKLVRVVAHALARKYH

>Turdus merula

VQWTAEEKQLITGLWGKVNVAECGGEALARLLIVYPWTQRFFASFGNLSSPTAVLGNPKVQAHGKKVLTSFGEAVKNLDSIKGTFAQLSELHCDKLHVDPENFRLLGDILVVVLAAHFGKDFTPDCQAAWQKLVRVVAHALARKYH

>Apus apus

VQWTAEEKQLITGLWGKVNVADCGAEALARLLIVYPWTQRFFASFGNLSSATAVIGNPMVRAHGKKVLTSFGEAVKNLDSIKSTFAQLSELHCDKLHVDPENFRLLGDILIIVLAAHFSKDFTPEAQQAWAKLVRAVAHALARKYH

>Columba livia

VHWSAEEKQLITSIWGKVNVADCGAEALARLLIVYPWTQRFFSSFGNLSSATAISGNPNVKAHGKKVLTSFGDAVKNLDNIKGTFAQLSELHCDKLHVDPENFRLLGDILVIILAAHFGKDFTPECQAAWQKLVRVVAHALARKYH

>Streptopelia orientalis

VHWSAEEKQLITSIWGKVNVADCGAEALARLLIVYPWTQRFFASFGNLSSATAISGNPNVKAHGKKVLTSFGDAVKNLDNIKGTFAQLSELHCNKLHVDPENFKLLGDILVIVLAAHFGKDFTPECQAAWQKLVRVVAHALARKYH

>Ciconia ciconia

VHWTAEEKQLITGLWGKVNVDECGAEALARLLIVYPWTQRFFASFGNLATASAITGNAMVHAHGKKVLTSFGEAVKNLDNIKNTFAQLSELHCDKLHVDPENFKLLGDILIIVLAAHFGKDFTPDCQAAWKKLVRVVAHALARKYH

>Phoenicopterus ruber

VHWSAEEKQLITSLWGKVNVADCGAEALARLLIVYPWTQRFFASFGNLSSPTAILGNPMVRAHGKKVLTSFGEAVKNLDNIKNTFAQLSELHCDKLHVDPENFRLLGDILIIVLAAHFAKDFTPECQAAWQKLVRVVAHALARKYH

>Aglaeactis castelnaudii

VHWTAEEKQLITGLWGKVNVEQCGAEALARLLIVYPWTQRFFASFGNLSSPTAVLGNPMVRAHGKKVLTSFGEAVKNLDSIKSTFAQLSELHCDKLHVDPENFRLLGDILIIVLAAHFAKDFTPECQAVWQKLVRAVAHALARKYH

>Coeligena violifer

VHWTAEEKQLITSLWGKVNVEQCGAEALARLLIVYPWTQRFFASFGNLSSPTAVLGNPMVRAHGKKVLTSFGEAVKNLDSIKSTFAQLSELHCDKLHVDPENFRLLGDILIIVLAAHFAKDLTPECQAVWQKLVRAVAHALARKYH

>Coeligena coeligena

VHWTAEEKQLITGLWGKVNVEQCGAEALARLLIVYPWTQRFFASFGNLSSPSAVLGNPNVRAHGKKVLTSFGEAVKNLDSIKGTFAQLSELHCDKLHVDPENFRLLGDILIIVLAAHFAKDFTPECQAVWQKLVRAVAHALARKYH

>Adelomyia melanogenys

VHWTAEEKQLITGLWGKVNVEQCGAEALARLLIVYPWTQRFFASFGNLSSPTAVLGNPMVRAHGKKVLTSFGEAVKNLDSIKGTFAQLSELHCDKLHVDPENFRLLGDILIIVLAAHFAKDFTPECQAVWQKLVRAVAHALARKYH

>Amazilia amazilia

VHWTAEEKQLITGLWGKVNVAECGAEALARLLIVYPWTQRFFASFGNLSSPTAVLGNPMVRAHGRKVLTSFGEAVKNLDSIKGTFAQLSELHCDKLHVDPENFRLLGDILIIVLAAHFAKDFTPECQAVWQKLVRAVAHALARKYH

>Oreotrochilus estella

VHWTAEEKQLITSLWGKVNVEQCGAEALARLLIVYPWTQRFFASFGNLSSPTAVLGNPMVRAHGKKVLTSFGEAVKNLDSIKSTFAQLSELHCDKLHVDPENFRLLGDILIIVLAAHFAKDFTPECQAVWQKLVRAVAHALARKYH

>Aquila chrysaetos

VHWTAEEKQLITGLWGKVNVADCGAEALARLLIVYPWTQRFFASFGNLSSPTAIIGNPMVRAHGKKVLTSFGEAVKNLDNIKNTFAQLSELHCDKLHVDPENFRLLGDILIIVLAAHFTKDFSPDCQAAWQKLVRAVAHALARKYH

>Accipiter gentilis

VQWAAEEKQLITGLWGKVNVADCGAEALARLLIVYPWTQRFFASFGNLSSATAVLGNPMVRAHGKKVLTSFGEAVKNLDNIKNTFAQLSELHCDKLHVDPENFRLLGDILIVVLAAHFGKDFSPDCQAAWQKLVRAVAHALARKYH

>Vultur gryphus

VHWSAEEKQLITGLWGKVNVAECGAEALARLLIVYPWTQRFFASFGNLSSPTAIIGNPMVRAHGKKVLTSFGEAVKNLDNIKNTFAQLSELHCEKLHVDPENFRLLGDILIIVLAAHFAKDFTPDCQAAWQKLVRAVAHALARKYH

>Aegypius monachus

VHWTAEEKQLITGLWGKVNVADCGAEALARLLIVYPWTQRFFASFGNLSSPTAIIGNPMVRAHGKKVLTSFGEAVKNLDNIKNTFAQLSELHCDKLHVDPENFRLLGDILIIVLAAHFGKDFSPDCQAAWQKLVRAVAHALARKYH

>Trigonoceps occipitalis

VHWTAEEKQLITGLWGKVNVADCGAEALARLLIVYPWTQRFFASFGNLSSPTAIIGNPMVRAHGKKVLTSFGEAVKNLDNIKNTFAQLSELHCDKLHVDPENFRLLGDILIIVLAAHFGKDFSPDCQAAWQKLVRAVAHALARKYH

>Gyps rueppellii

VHWTAEEKQLITGLWGKVNVADCGAEALARLLIVYPWTQRFFASFGNLSSPTAIIGNPMVRAHGKKVLTSFGEAVKNLDNIKNTFAQLSELHCDKLHVDPENFRLLGDILIIVLAAHFGKDFSPDCQAAWQKLVRAVAHALARKYH

>Stercorarius maccormicki

VHWSAEEKQLITGLWGKVNVADCGAEALARLLIVYPWTQRFFSSFGNLSSPTAIIGNPMVRAHGKKVLTSFGEAVKNLDNIKNTFAQLSELHCDKLHVDPENFRLLGDILIIVLAAHFAKEFTPDCQAAWQKLVRVVAHALARKYH

>Chroicocephalus ridibundus

VHWSAEEKQLITGLWGKVNVADCGAEALARLLIVYPWTQRFFASFGNLSSPTAINGNPMVRAHGKKVLTSFGEAVKNLDNIKNTFAQLSELHCDKLHVDPENFRLLGDILIIVLAAHFAKDFTPDSQAAWQKLVRVVAHALARKYH

>Phalacrocorax carbo

VHWTAEEKQLITGLWGKVNVAECGAEALARLLIVYPWTQRFFASFGNLSSATAITGNPMVRAHGKKVLTSFGEAVKNLDNIKATFAQLSELHCDKLHVDPENFRLLGDILIIVLAAHFAKDFTPECQAAWQKLVGAVAHALARKYH

>Sphenodon punctatus

VHWTAEEKQLVTSLWTKVNVDECGGEALGRLLIVYPWTQRFFSSFGNLSSSTAICGNPRVKAHGKKVFTSFGEAVKNLDNIKATYAKLSELHCEKLHVDPQNFNLLGDIFIIVLAAHFGKDFTPACQAAWQKLVRVVAHALAYHYH

>Naja naja

VHWSAEEKQLITSLWAKVDVPEVGAATLGKMMVMYPWTQRFFAHFGNLSGPSALCGNPQVRAHGKKVLTSFGEALKHLDNVKETFAKLSELHFDKLHVDPENFKLLGNVLIIVLAGHHGKEFTPSTHASFQKLVNVVAHALARRYH

>Microcephalophis gracilis

VHWSAEEKQLITGLWGKVDVAEVGGATLGKLLVVFPWTQRFFAHFGNLSSANAIICNPVVKAHGKKVLTSFGEAIKHLDSIKETFAKLSELHCEKLHVDPENFRLLGNILIIVLAGHHGKEFTPSTHAAFQKLVRAVAHSLARVYH

>Drymarchon melanurus erebennus

VHWTAEEKSAITSIWNKVDVPAVGSEALSRLLIVYPWTQRFFTSFGNLSNAAAIQSNAQVKAHGKKVFTAFGDAVKNPEAVKETFAKLSELHCDKLHVDPINFKLLGQILITVLAAHFGKEFTPHVQASYQKLVSVVAHALAHRYH

>Iguana iguana

VHWTAEEKQLITCLWGKVDVPTVGADALAGMLVMYPWTQRFFADFGNLSSATAICGNPKVRAHGKKVLTAFGDAIKNLDNIKDTFAKLSELHCDKLHVDPENFKLLGNVLIIVLAGHYGKDFTPACHAAYQKLVNVVAHALARRYH

>Trachemys scripta

VHWTAEEKQFITGLWGKVNVEECGGEALARLLIVYPWTQRFFSTFGNLSNAEAILHNPHVRAHGKKVLTSFGEAVKNLDHIKQTFATLSKLHCEKLHVDPENFKLLGNVLIIVMASHFTKEFTPACQAAWQKLVSAVAHALALGYH

>Chrysemys picta

VHWTADEKQLITSLWGKVNVEECGSEALARLLIVYPWTQRFFSTFGNLSNAEAILHNPHVHAHGKKVLTSFGEAVKNLDHIKQTFATLSKLHCEKLHVDPENFKLLGNVLIIVLASHFTKEFTPACQAAWQKLVSAVAHALALGYH

>Aldabrachelys gigantea

VHWTSEEKQYITSLWAKVNVGEVGGEALARLLIVYPWTQRFFASFGNLSSANAILHNAKVLAHGQKVLTSFGEAVKNLDNIKKTFAQLSELHCEKLHVDPENFKLLGNILIIVLATHFPKEFTPASQAAWTKLVNAVAHALALGYH

>Chelonoidis nigra

VHWTPEEKQYITSLWAKVNVEEVGGEALARLLIVYPWTQRFFSSFGNLSSASAILHNAKVLAHGKKVLTSFGDAVKNLDNIKKTFAQLSELHCEKLHVDPENFKLLGNILIIVLATRFPKEFTPASQAAWTKLVNAVAHALALGYH

>Chelonoidis chilensis

VHWTPEEKQYITSLWAKVNVEEIGGEALARLLIVYPWTQRFFSSFGNLSSASAILHNAKVLAHGKKVLTSFGDAVKNLDNIKKTFAQLSELHCEKLHVDPENFKLLGNILIIVLATRFPKEFTPACQAAWTKLVQAVAHALALGYH

>Podocnemis unifilis

SDFTQEERQFIVNLWGRVDVEQIGAEALARLLIVYPWTQRFFSSFGNLSSPSAILHNAKVHAHGKKVLTSFGEAVKNLDQIKQTFAQLSELHSDKLHVDPENFKLLGNILIIVLAAHFGKDFTPASQAAWQKLVSAVAHALALRYH

>Chelonia pleurodira

SDFTQEERQFIVNLWGRVDVEQIGAEALARLLIVYPWTQRFFSSFGNLSSPSAILHNAKVHAHGKKVLTSFGEAVKNLDQIKQTFAQLSELHSDKLHVDPENFKLLGNILIIVLAAHFGKDFTPASQAAWQKLVSAVAHALALRYH

>Caretta caretta

THWTAEERHYITSMWDKINVAEIGGESLARMLIVYPWTQKFFSDFGNLTSSSAIMHNVKIQEHGKKVLNSFGSAVKNMDHIKETFADLSKLHCETLHVDPENFKLLGSILIIVLAMHFGKEPTPTWQAAWQKLVSAVAHALTLQYH

>Crocodylus siamensis

SAFNPHEKQLIGDLWHKVDVAHCGGEALSRMLIVYPWKRRYFENFGDISNAQAIIHNEKVQAHGKKVLASFGEAVKHLDSIRAHFANLSKLHCEKFHVDPENFKLLGDIIIIVLAAHYPKDFGLECHAAYQKLVRQVAAALAAEYH

>Crocodylus niloticus

ASFDPHEKQLIGDLWHKVDVAHCGGEALSRMLIVYPWKRRYFENFGDISNAQAIMHNEKVQAHGKKVLASFGEAVCHLDGIRAHFANLSKLHCEKLHVDPENFKLLGDIIIIVLAAHYPKDFGLECHAAYQKLVRQVAAALAAEYH

>Alligator sinensis

ASFNAHEKKLIVELWAKVDVAQCGADALSRMLIVYPWKRRYFEHFGKMCNAHDILHNSKVQEHGKKVLASFGEAVKHLDNIKGHFANLSKLHCEKFHVDPENFKLLGDIIIIVLAAHHPDDFSMECHAAFQKLVRQVAAALAAEYH

>Alligator mississippiensis

ASFDAHERKFIVDLWAKVDVAQCGADALSRMLIVYPWKRRYFEHFGKMCNAHDILHNSKVQEHGKKVLASFGEAVKHLDNIKGHFANLSKLHCEKFHVDPENFKLLGDIIIIVLAAHHPEDFSVECHAAFQKLVRQVAAALAAEYH

>Caiman crocodilus

SPFSAHEEKLIVDLWAKVDVASCGGDALSRMLIIYPWKRRYFEHFGKLSTDQDVLHNEKIREHGKKVLASFGEAVKHLDNIKGHFAHLSKLHFEKFHVDCENFKLLGDIIIVVLGMHHPKDFTLQTHAAFQKLVRHVAAALSAEYH

>Xenopus laevis

VHWTAEEKAAITSVWQKVNVEHDGHDALGRLLIVYPWTQRYFSNFGNLSNSAAVAGNAKVQAHGKKVLSAVGNAISHIDSVKSSLQQLSKIHATELFVDPENFKRFGGVLVIVLGAKLGTAFTPKVQAAWEKFIAVLVDGLSQGYN

>Xenopus borealis

MGLTAHDRQLINSTWGKVCAKTIGKEALGRLLWTYPWTQRYFSSFGNLNSADAVFHNEAVAAHGEKVVTSIGEAIKHMDDIKGYYAQLSKYHSETLHVDPCNFKRFGGCLSISLARQFHEEYTPELHAAYEHLFDAIADALGKGYH

>Xenopus (Silurana) tropicalis

VHWTAEEKATIASVWGKVDIEQDGHDALSRLLVVYPWTQRYFSSFGNLSNVSAVSGNVKVKAHGNKVLSAVGSAIQHLDDVKSHLKGLSKSHAEDLHVDPENFKRLADVLVIVLAAKLGSAFTPQVQAVWEKLNATLVAALSHGYF

>Rana catesbeiana

VHWTAEEKAVINSVWQKVDVEQDGHEALTRLFIVYPWTQRYFSTFGDLSSPAAIAGNPKVHAHGKKILGAIDNAIHHLDNVKGTLHDLSEEHANQLHVDPENFRRLGEVLIVVLGAKLGKAFSPQVQHVWEKFIAVLVDALSHSYH

>Ambystoma mexicanum

VHLTAEERKDVGAILGKVNVDALGGQCLARLMCVYPWSRRYFPDFGDMSTCDAICHNARVLAHGAKVMRSVCEATKHLDNLQEYYADLSSTHCLKLFVDPQNFKLFGRIVVVCLAQTLQTEFTWHKQLAFEKLMRAVAHALSHSYH

>Triturus cristatus

VHLTAEDRKEIAAILGKVNVDSLGGQCLARLIVVNPWSRRYFHDFGDLSSCDAICRNPKVLAHGAKVMRSIVEATKHLDNLREYYADLSVTHSLKFYVDPENFKLFSGIVIVCLALTLQTDFSCHKQLAFEKLMKGVSHALGHGY-

>Latimeria chalumnae

VHWTETERATIETVYQKLHLDEVGREALTRLFIVYPWTTRYFKSFGDLSSSKAIASNPKVTEHGLKVMNKLTEAIHNLDHIKDLFHKLSEKHFHELHVDPQNFKLLSKCLIIVLATKLGKQLTPDVQATWEKLLSVVVAALSREYH

>Lepidosiren paradoxa

VHWEDAEKQYIVSVFSKIDVDHVGANTLERVLIVFPWTKRYFNSFGDLSSPGAIKHNNKVSAHGRKVLAAIIECTRHFGNIKGHLANLSHLHSEKLHVDPHNFRVLGQCLRIELAAALGKEFTPERNAYFQKFMDVISHSLGREYH

>Thunnus thynnus

VEWTQQERSIIAGFIANLNYEDIGPKALARCLIVYPWTQRYFGAYGDLSTPDAIKGNAKIAAHGVKVLHGLDRAVKNMDNINEAYSELSVLHSDKLHVDPDNFRILGDCLTVVIAANLGDAFTVETQCAFQKFLAVVVFALGRKYH

>Bathydraco marri

VNWSDTERAIITDIFSHLDYDDIGPKALSRCLIVYPWTQRHFSGFGNLHNADAILGNANVAAHGIKVLHGLDRGVKNMDNIVAAYTELSILHSEKLHVDPDNFKLLSDCITIVLAAKMGKAFTAEIQAAFQKFLGAVVSALGKQYH

>Notothenia coriiceps

VNWSDSERAIITDIFSHMDYDDIGPKALSRCLIVYPWTQRHFSGFGNLYNAEAILGNANVAAHGIKVLHGLDRGVKNMDKIVDAYAELSMLHSEKLHVDPDNFKLLSDCITIVVAAKMGHAFTPEIQGAFQKFLAVVVSALGKQYH

>Trematomus bernacchii

VEWTDKERSIISDIFSHMDYDDIGPKALSRCLIVYPWTQRHFSGFGNLYNAEAIIGNANVAAHGIKVLHGLDRGVKNMDNIAATYADLSTLHSEKLHVDPDNFKLLSDCITIVLAAKMGHAFTAETQGAFQKFLAVVVSALGKQYH

>Trematomus newnesi

VEWTDKERTIISDIFSHMDYDDIGPKALSRCLIVYPWTQRHFSGFGNLYNAEAIIGNANVAAHGIKVLHGLDRGMKNMDNIADAYTDLSTLHSEKLHVDPDNFKLLSDCITIVLAAKMGHAFTAETQGAFQKFLAAVVSALGKQYH

>Cygnodraco mawsoni

VKWSKTELTIINDIFSHLDYDDIGPKALSRCLIVYPWTQRHFSGFGNLYNAEAIIGNANVAAHGIKVLHGLDRGLKNMDNIVDAYAELSTLHSEKLHVDPDNFKLLSDCITIVLAAKLGKAFTAETQAAFQKFLAVVVSALGKQYH

>Cottoperca gobio

VEWTDFERATIKDVFSKIEYEVVGPAALARCLVVYPWTQRYFGNFGNLYNAAAITGNPKVAKHGITILHGLDKAVKNMDDIRNTYAELSVLHSEKLHVDPDNFKLLADCLTIVVAAQMGKAFTGEIQAAFQKFLAVVVSSLGRQYH

>Eleginops maclovinus

VEWTDQERATISSIFGSLDYDDIGPKALSRCLIVYPWTQRHFGSFGNLYNAEAIIGNQKVAAHGIKVLHGLDRAVKNMDNIKEIYAELSILHSEKLHVDPDNFKLLADCLTIVVAAKMGSGFNPGTQATFQKFLAVVVSALGKQYH

>Dissostichus mawsoni

VHWTEKERSIITGIFSHMDYEDIGPKALCRCLVVYPWTQRYFSCFGNLYNAEAIMGNANVAAHGIKVLHGLDRGVKNMDNIAATYAELSILHSEKLHVDPDNFKLLSDCITIVVAAKMGHAFNAETQAAFQKFLAVVVSALGKQYH

>Pogonophryne scotti

VQWSESERTIINGIFSHLDYDDLGPKAFSRCLIVYPWTQRYFSSFGNLHNAEAIMGNANVAAHGIKVLHGLDLGLKHMDDIMGAYAELSSLHSEKLHVDPDNFKLLSDCIIIAVAAKLGNAFTPETQAAFHKFLAVVVSALGKQYH

>Pseudaphritis urvillii

VVWTNEERSIISSIFSNLDYDDIGPKALCRCLIVYPWTQRHFTTFGNLYTPEAIMTNSKVAEHGVKVLHGLDRAVKNMDNIKATYYDLSILHSEKLHVDPDNFKLLSDCLTIVVAAKMGSGFTPETQAAFQKFLAVVVSALGKQYH

>Gobionotothen gibberifrons

VEWTDKERAIITDIFSHMDYDDIGPKALSRCLIVYPWTQRHFSGFGNLYNAEAIIGNANVAAHGIKVLHGLDRGVKNMDNIAATYAELSTLHSEKLHVDPDNFKLLSDCITIVVAAKLGHAFTAETQGALQKFLAVVVSALGKQYH

>Anoplopoma fimbria

VVWSDNERAIINSIFSGLDYEEVGPSALGRCLVVYPWTKRHFTNFGNLSNAAAIQGNPKVAAHGIKVLHGLDMALKNMDNIKATYADLSVLHSEKLHVDPDNFRVLSDCLTIVVAARMGNAFTPETQAAFQKFLAVVVSALGKQYH

>Artedidraco orianae

VQWSDSERTIINGIFSQLDYDDLGPKAFSRCLIVYPWTQRYFSSFGNLDNAEAIMGNANVAAHGIKVLHGLDRGVKNMDDIMGVYAELSSLHSEKLHVDPDNFKLLSDCITIVVAAKLGNAFTPETQAAFQKFLGAVVMFLGKQYH

>Pagothenia borchgrevinki

VEWTDKERSIISDIFSHLDYEDIGPKALSRCLIVYPWTQRHFSGFGNLYNAESIIGNANVAAHGIKVLHGLDRGLKNMDNIEATYADLSTLHSEKLHVDPDNFKLLADCITIVLAAKMGQAFTAEIQGAFQKFLAVVVSALGKQYH

>Gymnodraco acuticeps

VNWTKTEKATITDIFSHLDYDDIGPKALSRCLIVYPWTQRYFSGFGNLYNAAAIIGNAKVAEHGIKVLHGLDLGLKKMDNIEAAYADLSSLHSEKLHVDPDNFKLLSDCITIVLAAKLGSAFTAETQATFQKFLGAVMSALGKQYH

>Lycodes reticulatus

VKWTDKERAVILGIFSGLDYEDIGPKALVRCLIVYPWTQRYFGAFGNLSSAAAISGNLKIAAHGVKVLHGLDMALQHMDNIMETYADLSILHSETLHVDPDNFKLLADCLTITIAAKMGHCFTPDTQIAFHKFLAVVVSALGKQY-

>Anarhichas minor

VKWSDKERAVIISIFAGLDYEDIGPKALSRCLIVYPWTQRYFGSFGNLSTPAAIMGNPKIAAHGIKVLHGLDRGVKNMDNIKDAYTELSILHSETLHVDPDNFKLLADCLTIVVAAKMGCAFTPDTQLAFQKFLAVVVSALGKQYH

>Chelidonichthys kumu

VEWTDFERATIQDIFSKMDYETVGPATLTRTVIVYPWTLRYFAKFGNICSTAAILGNKEIAKHGTTILHGLDRGVKNMDDIKNTYAELSKLHSEKLHVDPDNFRLLSDCLTIVVAAKMGKDFTGEVQAAFQKFLSVVVNSLGRQYH

>Epinephelus coioides

VEWTDKERATIQDLFSKIDYDAVGQATLCRCLIVYPWTQRYFAKFGNLYNAAAIMGNADVAKHGSIILHGLDRAMKNMDNIKAAYTDLSTLHSETLHVDPDNFRLLSDCLAIVLAARMGKNFTPEIQATIQKFMAVVVSALGRQYH

>Liparis tunicatus

VHWTDFERSTIKDIFAKIDYDCVGPAAFARCLIVYPWTQRYFGNFGNLFNAAAIIGNPNVAKHGITIMHGLERGVKNLDHLTETYEELSVLHSEKLHVDPDNFKLISDCLTIVVASRLGKAFTGEVQAALQKFLAVVVFSLGKQYH

>Perca flavescens

VVWTDFERATIADIFSKLDYEAVGGATLARCLIVYPWTQRYFGNFGNLYNAAAIMGNPMIAKHGTTILHGLDRAVKNMDNIKATYAELSVLHSEKLHVDPDNFKLLSDCLTIVVAAQLGKAFSGEVQAAFQKFLSVVVSALGKQYH

>Conger conger

VQWSSSERSTISTLWGKINVAEIGPQALARVLIVYPWTQRYFGKFGDLSSVAAIVGNANGAKHGRTVLQALGQAVNNMDNIKGTYAKLSQKHSEELNVDPDNFRLLGDCLTVVLATKFGAEFPPEVQAVWQKFVAVVVSALSRQYF

>Gymnothorax unicolor anodic

VEWTDGERTAILTLWKKINVEEIGAQAMGRLLIVYPWTHRHFASFGNLSTPSAIMSNDKVAKHGATVMGGLDKAIKNMDDIKNAYRDLSVMHSEKLHVDPDNFRLLSECITLCVAAKFGKEFNADVHEAWYKFLMAVTSALARQYH

>Gymnothorax unicolor cathodic

VEWSSSERSTITSLWGKVIPAEIGPVAFARVLIVYPWTQRYFGNFGDLSNIAAISGNAKVAAHGKVVLDGVDKAVKNLDNIKGAYTSLSLLHSEKLNVDPDNFKLLGDCLTIVLATKFGAEFTPKVQAVWQKFLIVLIHALSRQYF

>Anguilla anguilla anodic

VEWTEDERTAIKSKWLKINIEEIGPQAMRRLLIVCPWTQRHFANFGNLSTAAAIMNNDKVAKHGTTVMGGLDRAIQNMDDIKNAYRQLSVMHSEKLHVDPDNFRLLAEHITLCMAAKFGTEFTADVQEAWQKFLMAVTSALARQYH

>Anguilla anguilla cathodic

VEWSASERSTITSLWGKINVAEIGPQALARVLIVYPWTQRYFGKFGDLSNAAAIQGNAKVAAHGKVVLGALEKAVKNMDDVKGTYSKLSQLHNEKLNVDPDNFRLLGDCLTIVLATKLGAGFPAEIQAVWQKFVAVVVSALSKQYF

>Cyprinus carpio

VEWTDAERSAIIGLWGKLNPDELGPQALARCLIVYPWTQRYFASFGNLSSPAAIMGNPKVAAHGRTVMGGLERAIKNMDNIKATYAPLSVMHSEKLHVDPDNFRLLADCITVCVAMKFGSGFNADVQEAWQKFLCVVVSALCRQYH

>Danio rerio

VEWTDAERTAILGLWGKLNIDEIGPQALSRCLIVYPWTQRYFATFGNLSSPAAIMGNPKVAAHGRTVMGGLERAIKNMDNVKNTYAALSVMHSEKLHVDPDNFRLLADCITVCAAMKFGAGFNADVQEAWQKFLAVVVSALCRQYH

>Pimephales promelas

VEWTDFERATIQDVFSKINYDVVGPQALARCLIVYPWTQRYFGNFGNLYNAAAIMGNPMVAAHGKTVLGGLDRAVKNMDNIKATYAELSVLHSEKLHVDPDNFRLLADCLTIVVAAQLGAAFTPAVQAAFQKFIAVVVSALGRQYH

>Carassius auratus

VEWTDAERSAIIGLWGKLNPDELGPQALARCLIVYPWTQRYFATFGNLSSPAAIMGNPKVAAHGRTVMGGLERAIKNMDNIKATYAPLSVMHSEKLHVDPDNFRLLADCITVCAAMKFGSGFNADVQEAWQKFLSVVVSALCRQYH

>Ctenopharyngodon idella

VEWTDDERTAILGLWGKLNIDEIGPQALSRCLIVYPWTQRYFATFGNLSSPAAIIGNPKVAAHGKTVMGGLERAIKNLDNIKATYSALSVMHSEKLHVDPDNFRLLADCITVCAAMKFGSGFNADVQEAWQKFLSVVVSALCRQYH

>Paramisgurnus dabryanus

VEWTDAERHSITGVWGKISVDEIGPQALARCLIVYPWTQRYFAAFGNLSSAAAIMGNPKVSAHGKVVMGGLERAIKNLDNIKDTYAALSIMHSEKLHVDPDNFRLLGDCITVCVAMKLGSVFTPDVHEAWQKFLSVVVSALCRQYH

>Misgurnus anguillicaudatus

VEWTDPERSAIIGLWGKLNPDELGPQALARCLIVYPWTQRYFASFGNLSSPAAIMDNPKVAAHGRTVMGGLERAIKNMDNIKATYAPLSVMHSEKLRVDPDNFRLLADYITVCAAMKFGSGFSANVQEAWQKFLSVVVSALCRQYH

>Brycon cephalus

VEWSTAERSAIAGLWGKISVDEIGPQALSRLLIVYPWTQRHFAAFGNLSSPAAINGNPKVAHHGKVVMGGLERAIKNMDNIKAAYSSLSVMHSEKLHVDPDNFRLLADCITVCVAMKFGSAFTPDVQEAWQKFLAVVVAALSR-YH

>Astyanax mexicanus

VEWTDFERATIQDIFSKMDYESVGHNALARCLVVYPWTQRYFGNFGNLYNAAAIMGNPKVAAHGVVVLHGLDRAVKNMDNIKATYAELSVLHSEKLHVDPDNFRLLADCLTIVVASRLGTGFTADVQAAFQKFLAVVGAALRKQYH

>Electrophorus electricus

VELTEAQRGAIVNLWGHLSPDEIGPQALARLLIVYPWTQRYFASFGNISSAAAIMGNPKVAAHGKVVVGALDKAVKNLNNIKGTYAALSTIHSEKLHVDPDNFRLLAESFTVSVAMKLGSGFNAETQHALAKFLAEVVSALGKQYH

>Ictalurus punctatus

VHWTDAERHIIADLWGKINHDEIGGQALARLLIVYPWTQRYFSSFGNLSNAAAIIGNPKVAAHGKVVLGGLTKAVQNLDNIKGIYTQLSTLHSEKLHVDPSNFTLLGDTFTVTLAANFGSVFTPEVHETWQKFLNVVVAALGKQYH

>Salmo salar

VDWTDAERSAIVGLWGKISVDEIGPQALARLLIVSPWTQRHFSTFGNLSTPAAIMGNPAVAKHGKTVMHGLDRAVQNLDDIKNAYTALSVMHSEKLHVDPDNFRLLADCITVCVAAKLGTVFSADIQEAFQKFLSVVVSALGRQYH

>Oncorhynchus mykiss

VDWTDAERSAIVGLWGKISVDEIGPQALARLLIVSPWTQRHFSTFGNLSTPAAIMGNPAVAKHGKTVMHGLDRAVQNLDDIKNTYTTLSVMHSEKLHVDPDNFRLLADCITVCVAAKLGAVFSADTQEAFQKFLAVVVSALGRQYH

>Esox lucius

VQWTAAERKAIASVWGSISADEIGPQSVARLLIVFPWTRRYFSSFGNLADSAAILGNPKVANHGKTVMKALDKAVQNLDNIKKTYTALSVTHSEKLHVDPDNFKLLSECITVCIAAKLGTVFDAYTHEAFYKFMCVVVSALSKQYH

>Osmerus mordax

VEWTEFERTTITSIFAQIDYDNVGPKALSRTLVVYPWTQRYFGNFGNLYNAAAIEGNKMVAAHGVVVIHGLERGWKNMDNIRETYSELSHLHSEKLHVDPDNFKLFADCLIIVLAAEMGSAFTAEIQGACQKFLNVVMSALGRQYH

>Gadus morhua

VEWTDSERAIITSIFSNLDYEEIGRKSLCRRLIVYPWTQRYFGGFGNLYNAETILCNPLIAAHGTKILHGLDRALKNMDDIKNTYAELSLLHSDKLHVDPDNFRLLADCLTVVIAAKMGPAFTVETQVAWQKFLSVVVSALGRQYH

>Oryzias latipes

VTWTDFERATIQDIFSKIDYDIVGPDALSRCLIVYPWTQRYFGSFGNLYNAAAIASNPKVAAHGKVVLGGLEKALKNMDDIKQAYADLSVLHSEKLQVDPDNFKLLADCLTIVVASQLGKDFTPEVHAAFAKFLAVVVAALRKQYH

>Poecilia formosa

VKWSDFERAAIQDIFSKIDYDVVGSAALSRCLIVYPWTQRYFGSFGNLYNAAAITSNPKVAAHGKVIMAGLEKALKNMDDIKTTYKDLSVLHSEKLQVDPDNFNLLGDCLTIVVAGQLGAAFTPEVQGAFQKFLAVVVASLRKQYH

>Xiphophorus maculatus

VKWSDFERAAIQDIFSKINYDVVGCAALSRCLIVYPWTQRYFGSFGNLYNAAAITSNPKVAAHGKVIMAGLEKAVKNMDDIKTTYKDLSVLHSEKLQVDPDNFNLLADCLTIVVAGQMGAAFTPEVHGAFQKFLAVVVASLRKQYH

>Takifugu rubripes

VEWTDQERTIISNIFSTLDYEDVGSKSLIRCLIVYPWTQRYFAGFGNLYNAEAIKNNPNIAKHGVTVLHGLDRAVKNMDNIKETYKELSELHSEKLHVDPDNFKLLSDCLTIVVATKMGSKFTPEIQATFQKFLAVVVSALGRQYH

>Scophthalmus maximus

VEWTDQERSVITSIFDNLDYDDIGPKALCRCLIVYPWTLRYFSSFGNLYNSEAIKNNKKIADHGIKVLHGLDRAVKNMDNIKATYAELSILHSDTLHVDPDNFRLLADCLTIVIAAKMGSAFTPEKQATWQKFLAVVVSALGRQYH

>Platichthys flesus

VQWTDSERSSIIALWGKIDVGEIGPQALTRLLIVYPWTQRHFSTFGDLSTTAAILGSEKVAKHGKTVMGGLERAVKSLDDIKGVYSALSTMHSEKLHVDPDNFRLLAECISVCVAAKFGSVFTAEVQEAWQKFLSVVVSALGRQYH

>Oreochromis niloticus

VEWSEKERSIITNIFSNLDYEDVGPKALVRCLVVYPWTQRYFASFGNLYNAEAISTNPKVAAHGIKVLHGLDRAVKNMDNIKATYAELSVLHSEKLHVDPDNFKLLSDCLTIVVAGKLGSAFTPEVQATFQKFLAVVVSALGKQYH

>Neolamprologus brichardi

VEWTDAERSAIIGLWGKIDVDEIGPQALSRLLIVYPWGQRYFKAFGDLSTNAAILGNPKVAQHGRTVMGGLENAVKNLDNIKQTYAKLSVMHSEKLHVDPDNFRVLAECISLCVGAKFGSVFTPEVQEAWQKFLSVVVSALGRQYH

>Haplochromis burtoni

VEWTDAERKAIIGLWGKIDVGEIGPQALSRLLIVYPWGQRYFKAFGDLSTNAAIMGNPKVAQHGKTVMGGLENAVKNLDNIKQTYAKLSAMHSEKLHVDPDNFRVLAECISLCVAAKFGSVFTAEVQEAWQKFLSVVVSALGRQYH

>Pundamilia nyererei

VEWTDAERKAIIGLWGKIDVGEIGPQALSRLLIVYPWGQRYFKAFGDLSTNAAIMGNPKVAQHGRTVMGGLENAVKNLDNIKQTYAKLSAMHSEKLHVDPDNFRVLAECISLCVAAKFGSVFTAEVQEAWQKFLSVVVSALGRQYH

>Maylandia zebra

VEWTDAERSAIINLWGKIDVGEIGPQALSRLLIVYPWGQRYFQAFGDLSTNAAIMGNPKVAQHGRTVMGGLENAVKNLDNITQTYAKLSAMHSEKLHVDPDNFRVLAECISLCVAAKFGSVFTAEVQEAWQKFLSVVVSALGRQYH

>Leiostomus xanthurus

VDWTDAERAAIKALWGKIDVGEIGPQALSRLLIVYPWTQRHFKGFGNISTNAAILGNAKVAEHGKTVMGGLDRAVQNMDNIKNVYKQLSIKHSEKIHVDPDNFRLLGEIITMCVGAKFGSAFTPEIHEAWQKFLAVVVSALGRQYH

>Callorhinchus milii

VQWSQAELDVIQGKWAALDPEKFGGKALARMFVVYPWTKRYFGKFG----GRFKASDSVVMEHGAKVMGKMQVAAKDPEKIKEIFEYLSKRHSDTIHVDPENFKLLGSCMLVEMAMTKG-DWSPEIEAINRKFVDVSIAALSRKYH

>Mustelus griseus

VHWTQEERDEISKTFQGTDMKTVVTQALDRMFKVYPWTNRYFQK----------RTDFRSSIHAGIVVGALQDAVKHMDDVKTLFKDLSKKHADDLHVDPGSFHLLTDCIIVELAYLRKDCFTPHIQGIWDKFFEVVIDAISKQYH

>Squalus acanthias

VHWTGEEKALVNAVWTKTDHQAVVAKALERLFVVYPWTKTYFVKFN----GKFHASDSTVQTHAGKVVSALTVAYNHIDDVKPHFVELSKKHYEELHVDPENFKLLANCLEVELGHALHKEFTPEVQAAWSKFSNVVVDALSKGYH

>Torpedo marmorata

VSLTDEEIRLIQHIWSNVNVVEITAKALERVFYVYPWTTRLFTSFN----HNFKASDKQVHDHAVNVSNAISAAIGDLHDINKNFSALSTKHQKKLGVDTSNFMLLGQAFLVELAALEKDKFTPQYHKAALKLFEVVTEALSCQYH

>Dasyatis akajei

VKLSEDQEHYIKGVWKDVDHKQITAKALERVFVVYPWTTRLFSKLQ----GLFSANDIGVQQHADKVQRALGEAIDDLKKVEINFQNLSGKHQ-EIGVDTQNFKLLGQTFMVELALHYKKTFRPKEHAAAYKFFRLVAEALSSNYH

>Bathyraja eatonii

VKITDKKAAYITGIWSKLDKKVTTAHALERVFTVYPWTTRLFKSFN----GHFKAGDSGVQGHAEKVVGALDTAVLHLHDIDAGYKKLSEKHQ-LIGVDTQNFKLLGQAFLVELAILFKEGFTPELHEAAYKFFLAVAGGLSSQYH

Alpha-synuclein

>mouse Mus musculus

MDVFMKGLSKAKEGVVAAAEKTKQGVAEAAGKTKEGVLYVGSKTKEGVVHGVTTVAEKTKEQVTNVGGAVVTGVTAVAQKTVEGAGNIAAATGFVKKDQMGK-GEEGYPQEGILE---DMPVDPGSEAYEMPSEEGYQDYEPEA

>rat rattus norvergicus

MDVFMKGLSKAKEGVVAAAEKTKQGVAEAAGKTKEGVLYVGSKTKEGVVHGVTTVAEKTKEQVTNVGGAVVTGVTAVAQKTVEGAGNIAAATGFVKKDQMGK-GEEGYPQEGILE---DMPVDPSSEAYEMPSEEGYQDYEPEA

>g.hamster Mesocricetus auratus

MDVFMKGLSKAKEGVVAAAEKTKQGVAEAAGKTKEGVLYVGSKTKEGVVHGVTTVAEKTKEQVTNVGGAVVTGVTAVAQKTVEGAGNIAAATGFVKKDKTGR-GEEGYPQEGILE---DMPVEPGSEAYEMPSEEGYQDYEPEA

>c.hamster Cricetulus griseus

MDVFMKGLSKAKEGVVAAAEKTKQGVAEAAGKTKEGVLYVGSKTKEGVVHGVTTVAEKTKEQVTNVGGAVVTGVTAVAQKTVEGAGNIAAATGFVKKDQLGK-GEEGYPQEGILE---DMPVDPGSEAYEMPSEEGYQDYEPEA

>jerboa Jaculus jaculus

MDVFMKGLSKAKDGVVAAAEKTKQGVAEAAGKTKEGVLYVGSKTKEGVVHGVTTVAEKTKEQVTNVGGAVVTGVTAVAQKTVEGAGNIAAATGFVKKDQMGK-TEEGSPQEGILG---DMPVDPDNEAYEMPSEEGYQDYEPEA

>human Homo sapiens

MDVFMKGLSKAKEGVVAAAEKTKQGVAEAAGKTKEGVLYVGSKTKEGVVHGVATVAEKTKEQVTNVGGAVVTGVTAVAQKTVEGAGSIAAATGFVKKDQLGK-NEEGAPQEGILE---DMPVDPDNEAYEMPSEEGYQDYEPEA

>chimpanzee Pan troglodytes

MDVFMKGLSKAKEGVVAAAEKTKQGVAEAAGKTKEGVLYVGSKTKEGVVHGVATVAEKTKEQVTNVGGAVVTGVTAVAQKTVEGAGSIAAATGFVKKDQLGK-NEEGAPQEGILE---DMPVDPDNEAYEMPSEEGYQDYEPEA

>gibbon Nomascus leucogenys

MDVFMKGLSKAKEGVVAAAEKTKQGVAEAAGKTKEGVLYVCSKTKEGVVHGVATVAEKTKEQVTNVGGAVVTGVTAVAQKTVEGAGSIAAATGFVKKDQLGK-SEEGAPQEGILE---DMPVDPDNEAYEMPSEEGYQDYEPEA

>orangutan Pongo abelii

MDVFMKGLSKAKEGVVAAAEKTKQGVAEAAGKTKEGVLYVGSKTKEGVVHGVATVAEKTKEQVTNVGGAVVTGVTAVAQKTVEGAGSIAAATGFVKKDQLGK-NEEGATQEGILE---DMPVDPDNEAYEMPSEEGYQDYEPEA

>baboon Papio anubis

MDVFMKGLSKAKEGVVAAAEKTKQGVAEAAGKTKEGVLYVGSKTKEGVVHGVATVAEKTKEQVTNVGGAVVTGVTAVAQKTVEGAGSIAAATGFVKKDQLGK-NEEGTPQEGILQ---DMPVDPDNEAYEMPSEEGYQDYEPEA

>rhesus Macaca mulatta

MDVFMKGLSKAKEGVVAAAEKTKQGVAEAAGKTKEGVLYVGSKTKEGVVHGVATVAEKTKEQVTNVGGAVVTGVTAVAQKTVEGAGSIAAATGFIKKDQLGK-NEEGAPQEGILQ---DMPVDPDNEAYEMPSEEGYQDYEPEA

>greenmonkey Chlorocebus sabaeus

MDVFMKGLSKAKEGVVAAAEKTKQGVAEAAGKTKEGVLYVGSKTKEGVVHGVATVAEKTKEQVTNVGGAVVTGVTAVAQKTVEGAGSIAAATGFVKKDQLGK-NEEGAPQEGILQ---DMPVDPDNEAYEMPSEEGYQDYEPEA

>sq.monkey Saimiri boliviensis

MDVFMKGLSKAKEGVVAAAEKTKQGVAEAAGKTKEGVLYVGSKTKEGVVHGVATVAEKTKEQVTNVGGAVVTGVTAVAQKTVEGAGNIAAATGFVKKDHLGK-SEEGAPQEGILE---DMPVDPDNEAYEMPSEEGYQDYEPEA

>marmoset Callithrix jacchus

MDVFMKGLSKAKEGVVAAAEKTKQGVAEAAGKTKEGVLYVGSKTKEGVVHGVTTVAEKTKEQVTNVGGAVVTGVTAVAQKTVEGAGNIAAATGFVKKDHLGK-SEEGAPQEGILE---DMPVDPDNEAYEMPSEEGYQDYEPEA

>treeshrew Tupaia chinensis

MDVFMKGLSKAKEGVVAAAEKTKQGVAEAAGKTKEGVLYVGSKTKEGVVHGVTTVAEKTKEQVTNVGGAVVTGVTAVAQKTVEGAGSIAAATGFGKKDQLGK-SEEGAPQEGILE---DMPVDPDNEAYEMPSEEGYQDYEPEA

>galago Otolemur garnettii

MDIFKKGLSKAKEGVVAAAEKTKQGVAEAAGKTKEGVLYVGSKTKEGVVHGVTTVAEKTKEQVTNVGGAVVTGVTAVAQKTVEGAGSIAAATGFGKKDQFGK-NDEGAPQEGILE---DMPVDPDSEAYEMPSEEGYQDYEPEA

>orca Orcinus orca

MDVFMKGLSKAKEGVVAAAEKTKQGVAEAAEKTKEGVLYVGSKTKEGVVHGVTTVAEKTKEQVTNVGEAVVTGVTAVAQKTVEGAGSIAAATGFGKKDQLGK-NDEGASQEGILE---DTPVDPDNEAYEMPSEEGYQDYEPEA

>baiji Lipotes vexillifer

MDVFMKGLSKAKEGVVAAAEKTKQGVAEAAEKTKEGVLYVGSKTKEGVVHGVTTVAEKTKEQVTNVGEAVVTGVTAVAQKTVEGAGSIAAATGFGRKDQLGK-NDEGASQEGILE---DTPVEPDNEAYEMPSEEGYQDYEPEA

>minkewhale Balaenoptera acutorostrata scammoni

MDVFMKGLSKAKEGVVAAAEKTKQGVAEAAEKTKEGVLYVGSKTKEGVVHGVTTVAEKTKEQVTNVGEAVVTGVTAVAQKTVEGAGSIAAATGFGKKDQLGK-S-EGASQEGILE---DTPVDPDNEAYEMPSEEGYQDYEPEA

>pig Sus scrofa

MDVFMKGLSKAKEGVVAAAEKTKQGVAEAAGKTKEGVLYVGSKTKEGVVHGVTTVAEKTKEQVTNVGEAVVTGVTAVAQKTVEGAGSIAAATGFGKKDQLGK-NEEGAPQEGILE---DMPVDPDNEAYEMPSEEGYQDYEPEA

>panda Ailuropoda melanoleuca

MDVFMKGLSKAKEGVVAAAEKTKQGVAEAAGKTKEGVLYVGSKTKEGVVHGVTTVAEKTKEQVTNVGEAVVTGVTAVAQKTVEGAGSIAAATGFGKKDQLGK-SEEGGPQEGILE---DMPVDPDNEAYEMPSEEGYQDYEPEA

>ferret Mustela putorius furo

MDVFMKGLSKAKEGVVAAAEKTKQGVAEAAGKTKEGVLYVGSKTKEGVVHGVTTVAEKTKEQVTNVGEAVVTGVTAVAQKTVEGAGSIAAATGFGKKDQLGK-SEEGGPQEGILE---DMPVDPDNEAYEMPSEEGYQDYEPEA

>cat Felis catus

MDVFMKGLSKAKEGVVAAAEKTKQGVAEAAGKTKEGVLYVGSKTKEGVVHGVTTVAEKTKEQVTNVGEAVVTGVTAVAQKTVEGAGSIAAATGFGKKDQLGK-NEEGGPQEGILE---DMPVDPDNEAYEMPSEEGYQDYEPEA

>dog Canis lupus familiaris

MDVFMKGLSKAKEGVVAAAEKTKQGVAEAAGKTKEGVLYVGSKTKEGVVHGVTTVAEKTKEQVTNVGEAVVTGVTAVAQKTVEGAGSIAAATGFGKKDQLGK-SEEGGPQEGILE---DMPVDPDNEAYEMPSEEGYQDYEPEA

>cow Bos taurus

MDVFMKGLSKAKEGVVAAAEKTKQGVAEAAGRTKEGVLYVGSKTKEGVVHGVTTVAEKTKEQVTNVGEAVVTGVTAVAQKTVEGAGSIAAATGFGKKDHMGK-GEEGASQEGILE---DMPVDPDNEAYEMPSEEGYQDYEPEA

>yak Bos mutus

MDVFMKGLSKAKEGVVAAAEKTKQGVAEAAGRTKEGVLYVGSKTKEGVVHGVTTVAEKTKEQVTNVGEAVVTGVTAVAQKTVEGAGSIAAATGFGKKDHMGK-GEEGASQEGILE---DMPVDPDNEAYEMPSEEGYQDYEPEA

>waterbuffalo

MDVFMKGLSKAKEGVVAAAEKTKQGVAEAAGKTKEGVLYVGSKTKEGVVHGVTTVAEKTKEQVTNVGEAVVTGVTAVAQKTVEGAGSIAAATGFGRKDHLGK-GEEGASQEGILE---DMPVDPDNEAYEMPSEEGYQDYEPEA

>sheep Ovis aries

MDVFMKGLSKAKEGVVAAAEKTKQGVAEAAGKTKEGVLYVGSKTKEGVVHGVTTVAEKTKEQVTNVGEAVVTGVTAVAQKTVEGAGSIAAATGFGKKDHLGK-GEEGASQEGILE---DMPVDPDNEAYEMPSEEGYQDYEPEA

>goat Capra hircus

MDVFMKGLSKAKEGVVAAAEKTKQGVAEAAGKTKEGVLYVGSKTKEGVVHGVTTVAEKTKEQVTNVGEAVVTGVTAVAQKTVEGAGSIAAATGFGKKDHLGK-GEEGASQEGILE---DMPVDPDNEAYEMPSEEGYQDYEPEA

>rhinoceros Ceratotherium simum simum

MDVIKKGLSKAKEGVVAAAEKTKQGVAEAAGKTKEGVLYVGSKTKEGVVHGVTTVAEKTKEQVTNVGEAVVTGVTAVAQKTVEGAGSIAAATGFGKKDQLGK-SEEGAPQEGILE---DMPVDPDNEAYEMP-EEGYQDYEPEA

>camel Camelus ferus

MDVLMKGLSKAKEGVVAAAEKTKQGVAEAAGKTKEGVLYVGSKTKEGVVHGVTTVAEKTKEQVTNVGEAVVTGVTAVAQKTVEGAGSIVAATGFGKKDQLSK-SEEGAPQEGMLE---DVPAEPDNEAYEMPSEEGYQDYEPEA

>alpaca Vicugna pacos

MDVLMKGLSKAKEGVVAAAEKTKQGVAEAAGKTKEGVLYVGSKTKEGVVHGVTTVAEKTKEQVTNVGEAVVTGVTAVAQKTVEGAGSIAAATGFGKKDQLSK-SEEGAAQEGVLE---DVPTEPDNEAYEMPSEEGYQDYEPEA

>horse Equus caballus

MDVFMKGLSKAKEGVVAAAEKTKQGVAEAAGKTKEGVLYVGSKTKEGVVHGVTTVAEKTKEQVTNVGEAVVTGVTAVAQKTVEGAESIAAATGFGKKDHLGK-SEEGAAQEGILE---DMPVDPDNEAYEMPSEEGYQDYEPEA

>bat Myotis brandtii

MDAFMKGLSKAKEGVVAAAEKTKQGVAEAAGKTREGVLYVGSRTKEGVVHGVTTVAEKTKEQVTNVGEAVVTGVTAVAQRTVEGAGNIAAATGFGRKDQVGK-SEEGAPQEGILE---DVPGEPD-ETYEMPSEGGYQDYEPEA

>bigbrownbat Eptesicus fuscus

MDAFMKGLSKAKEGVVAAAEKTKQGVAEAAGKTKEGVLYVGSRTKEGVVHGVTTVAEKTKEQVTNVGEAVVTGVTAVAQRTVEGAGNIAAATGFGRKDQLGK-SEEGAPQEGILE---DVPGEPDNETYEMPSEGAYQDYEPEA

>flyingfox Pteropus alecto

MDVFMKGLSKAKEGVVAAAEKTKQGVAEAAGKTKEGVLYVGSKTKEGVVHGVTTVAEKTKEQVTNVGEAVVTGVTAVAQKTVEGAGSIAAATGFGRKDQLGK-SEEGAPQEGILE---DMPMDPENETYEMPSEEGYQDYEPEA

>mole Condylura cristata

MDVFMKGLSKAKEGVVAAAEKTKQGVAEAAGKTKEGVLYVGSKTKEGVVHGVTTVAEKTKEQVTNVGEAVVTGVTAVAQKTVEGAGSIAAATGFGKKDQFGK-SEEGVPQEGILE---DMPVDPDSEAYEMPSEEGYQDYEPEA

>elephantshrew Elephantulus edwardii

MDVFMKGLSKAKEGVVAAAEKTKQGVAEAAGKTKEGVLYVGSKTKEGVVHGVTTVAEKTKEQVTNVGEAVVTGVTAVAQKTVEGAGNIAAATGFGKKDQMGK-GEEGVSQEGILE---DMPVDPDNETYEMPSEEGYQDYEPEA

>armadillo Dasypus novemcinctus

MDVFMKGLSKAKEGVVAAAEKTKQGVAEAAGKTKEGVLYVGSKTKEGVVHGVTTVAEKTKEQVTNVGEAVVTGVTAVAQKTVEGAGSIAAATGFGKKDQLGK-SEEGGPQEGILE---DMPVDPDSEAYEMPSEEGYQDYEPET

>elephant Loxodonta africana

MDVFMKGLSKAKEGVVAAAEKTKQGVAEAAGKTKEGVLYVGSKTKEGVVHGVTTVAEKTKEQVTNVGEAVVTGVTAVAQKTVEGAGSIAAATGFGKKDQMGK-GEEGAPQEGILE---NVPVDPDNEAYEMPSEEGYQDYEPEA

>manatee Trichechus manatus latirostris

MDVFMKGLSKAKEGVVAAAEKTKQGVAEAAEKTKEGVLYVGSKTKEGVVHGVTTVAEKTKEQVTNVGEAVVTGVTAVAQKTAEGAGTIVAATGFGKKDQMGK-NEEGASQEGILQ---DMPVDPDNEAYEMPSEEGYQDYEPEA

>guineapig Cavia porcellus

MDVFMKGLSKAKEGVVAAAEKTKQGVAEAAGKTKEGVLYVGSKTKEGVVHGVTTVAEKTKEQVTNVGGAVVTGVTAVAHKTVEGAGNIAAATGFVRKDQLGK-NEEGSSQEGILE---DMPVDPDNEAYEMPSEEGYQDYEPEA

>degu Octodon degus

MDVFMKGLSKAKEGVVAAAEKTKQGVAEAAGKTKEGVLYVGSKTKEGVVHGVTTVAEKTKEQVTNVGGAVVTGVTAVAQKTVEGAGNIAAATGFVKKDQLGK-SEEGSPQEGILE---DMPVDPDSEAYEMPSEEGYQDYEPEA

>gr.squirrel Ictidomys tridecemlineatus

MDVFMKGLSKAKEGVVAAAEKTKQGVAEAAGKTKEGVLYVGSKTKEGVVHGVTTVAEKTKEQVTNVGGAVVTGVTAVAQKTVEGAGNIAAATGFVKKDQLGK-SEEGSPQEGILE---DMPVEPDNEAYEMPSEEGYQDYEPEA

>chinchilla Chinchilla lanigera

MDVFMKGLSKAKEGVVAAAEKTKQGVAEAAGKTKEGVLYVGSKTKEGVVHGVTTVAEKTKEQVTNVGGAVVTGVTAVAQKTVEGAGNIAAATGFVKKDQLGK-SEEGSPQEGILE---DMPVEPDSEAYEMPSEEGYQDYEPEA

>molerat Heterocephalus glaber

MDVFMKGLSKAKEGVVAAAEKTKQGVAEAAGKTKEGVLYVGSKTKEGVVHGVTTVAEKTKEQVTNVGGAVVTGVTAVAQKTVEGAGNIAAATGFVKKDQLGK-SEEGSPQEGILE---DMPVDPDNEAYEMPSEEGYQDYEPEA

>lessermolerat Nannospalax galili

MDVFMKGLSKAKEGVVAAAEKTKQGVAEAAGKTKEGVLYVGSKTKEGVVHGVTTVAEKTKEQVTNVGGAVVTGVTAVAQKTVEGAGNIAAATGFVKKD---K-GEEGSPQEGVLG---DMPVDPDSEAYEMPPEEGYQDYEPEA

>rabbit Oryctolagus cuniculus

MDVFMKGLSKAKEGVVAAAEKTKQGVAEAAGKTKEGVLYVGSKTKEGVVHGVTTVAEKTKEQVTNVGEAVVTGVTAVAQKTVEGAGSIAAATGFVKKDQQGK-SEEGAPQEGILE---AMPMDPDSEAYEMPSEEGYQDYEPEA

>pika Ochotona princeps

MDVFMKGLSKAKEGVVAAAEKTKQGVAEAAGKTKEGVLYVGSKTKEGVVHGVTTVAEKTKEQVTNVGGAVVTGVTAVAQKTVEGAGNIAAATGFVKKDQQGK-SEEGASQEGILE---DMPVDPDNEAYEMPSEEGYQDYEPEA

>tenrec Echinops telfairi

MDVFMKGLSKAKEGVVAAAEKTKQGVAEAAGKTKEGVLYVGSKTKEGVVHGVTTVAEKTKEQVTNVGEAVVTGVTAVAQKTVEGAGNIAAATGFGKKDQTGK-NEEGVPQEGILE---DMPTDPDNEAYEMPSEEGYQDYEPEA

>pigeon Columba livia

MDVFMKGLSKAKEGVVAAAEKTKQGVAEAAGKTKEGVLYVGSRTKEGVVHGVTTVAEKTKEQVSNVGGAVVTGVTAVAQKTVEGAGNIAAATGLVKKDPLAKQNEEGFLQEGMVNNA-DVPVDPENEAYEMPPEEEYQDYEPEA

>flycatcher Ficedula albicollis

MDVFMKGLSKAKEGVVAAAEKTKQGVAEAAGKTKEGVLYVGSRTKEGVVHGVTTVAEKTKEQVSNVGGAVVTGVTAVAQKTVEGAGNIAAATGLVKKDQLAKQNEEGFLQEGMVNNT-GVPVDPESEAYEMPPEEEYQDYEPEA

>sparrow Zonotrichia albicollis

MDVFMKGLSKAKEGVVAAAEKTKQGVAEAAGKTKEGVLYVGSRTKEGVVHGVTTVAEKTKEQVSNVGGAVVTGVTAVAQKTVEGAGNIAAATGLVKKDQLAKQNEEGFLQEGMVNNT-GAPVDPENEAYEMPPEEEYQDYEPEA

>zebrafinch Taeniopygia guttata

MDVFMKGLSKAKEGVVAAAEKTKQGVAEAAGKTKEGVLYVGSRTKEGVVHGVTTVAEKTKEQVSNVGGAVVTGVTAVAQKTVEGAGNIAAATGLVKKDQLAKQNEEGFLQEGMVNNT-GVAVDPENEAYEMPPEEEYQDYEPEA

>mallard Anas platyrhynchos

MDVFMKGLNKAKEGVVAAAEKTKQGVAEAAGKTKEGVLYVGSRTKEGVVHGVTTVAEKTKEQVSNVGGAVVTGVTAVAQKTVEGAGNIAAATGLVKKDHLAKQNEEGFLQEGMVNNT-DVPVDPENEAYEMPPEEEYQDYEPEA

>parakeet Melopsittacus undulatus

MDVFMKGLSKAKEGVVAAAEKTKQGVAEAAGKTKEGVLYVGSRTKEGVVHGVTTVAEKTKEQVSNVGGAVVTGVTAVAQKTVEGAGNIAAATGLVKKDQFAKQNEEGFLQEGMVNNT-NVAVDPENEAYEMPPEEEYQDYEPEA

>gr.tit Pseudopodoces humilis

MDVFMKGLSKAKEGVVAAAEKTKQGVAEAAGKTKEGVLYVGSRTKEGVVHGVTTVAEKTKEQVSNVGGAVVTGVTAVAQKTVEGAGNIAAATGLVKKDQLAKQNEEGFLQEGMVNNT-GVPVDPENEAYEMPPEEEYQDYEPEA

>falcon Falco peregrinus

MDVFMKGLSKAKEGVVAAAEKTKQGVAEAAGKTKEGVLYVGSRTKEGVVHGVTTVAEKTKEQVSNVGGAVVTGVTAVAQKTVEGAGNIAAATGLVKKDQLAKQNEEGFLQEGMVNNT-DVPVDPENEAYEMPPEEEYQDYEPEA

>hummingbird Calypte anna

MDVFMKGLSKAKEGVVAAAEKTKQGVAEAAGKTKEGVLYVGSRTKEGVVHGVTTVAEKTKEQVSNVGGAVVTGVTAVAQKTVEGAGSIAAATGLVKKDQLGKQNEEGFLQEGMVNNA-DVAMDPENEAYEMPPEEEYQDYEPEA

>manakin Manacus vitellinus

MDVFMKGLSKAKEGVVAAAEKTKQGVAEAAGKTKEGVLYVGSRTKEGVVHGVTTVAEKTKEQVSNVGGAVVTGVTAVAQKTVEGAGNIAAATGLVKKDQLAKQNEEGFLQEGMVNNS-DVPVDPENEAYEMPPEEEYQDYEPEA

>emp.penguin Aptenodytes forsteri

MDVFMKGLSKAKEGVVAAAEKTKQGVAEAAGKTKEGVLYVGSRTKEGVVQGVTTVAEKTKEQVSNVGGAVVTGVTAVAQKTVEGAGNIAAATGLVKKDQLAKQNEEGFLQEGMVNNT-DVPVDPENEAYEMPPEEEYQDYEPEA

>cuckoo Cuculus canorus

MDVFMKGLSKAKEGVVAAAEKTKQGVAEAAGKTKEGVLYVGSRTKEGVVHGVTTVAEKTKEQVSNVGGAVVTGVTAVAQKTVEGAGNIAAATGLVKKDQLAKQNEEGFLQEGMVNNT-DVPVDPENEAYEMPPEEDYQDYEPEA

>woodpecker Picoides pubescens

MDVFMKGLSKAKEGVVAAAEKTKQGVAEAAGKTKEGVLYVGSRTKEGVVHGVTTVAEKTKEQVSNVGGAVVTGVTAVAQKTVEGAGNIAAATGLVKKDQLAKQNEEGFVQEGMVNNA-DLPVDPENEAYEMPPEEDYQDYEPEA

>alligator Alligator sinensis

MDVFMKGLSKAKEGVVAAAEKTKQGVAEAAGKTKEGVLYMGSRTKEGVVHGVTTVAEKTKEQVSNVGGAVVTGVTAVAQKTVEGAGNIAAATGLVKKDQLAKQNEEGIPQEGMMDNT-DMPVDTENEAYEMPPEEKYQDYEPEA

>alligator Alligator mississippensis

MDVFMKGLSKAKEGVVAAAEKTKQGVAEAAGKTKEGVLYMGSRTKEGVVHGVTTVAEKTKEQVSNVGGAVVTGVTAVAQKTVEGAGNIAAATGLVKKDQLAKQNEEGIPQEGMMDNT-DMPVDTENEAYEMPPEEKYQDYEPEA

>anole Anolis carolinensis

MDVFMKGFNKAKDGVVAAAEKTKQGVAEAAGKTKEGVLYVGSKTRDGVVQGVTTVAEKTKEQVSNVGGAVVTGVTAVAQKTVEGAGNFAAATGFVKKDQLGKQ-DEGLPQEGMMANT-DMPVDPENEAYEMPPEEEYQDYEPEA

>burmesepython Python bivittatus

MDVFMKGFNKAKEGVVAAAEKTKQGVAEAAGKTKEGVLYVGSKTKEGVVHGVTTVAEKTKEQVSNVGGAVVTGVTAVAQKTVEGAGNIAAATGFVKKDQLAKQNEEGLTQEGIMDNT-DP---AENEAYEMPPEEEYQDYEPEA

>pa.turtle Chrysemys picta bellii

MDVFMKGLSKAKDGVVAAAEKTKQGVAEAAGKTKEGVLYVGSRTRDGVVHGVTTVAEKTKEQVSNVGGAVVTGVTAVAQKTVEGAGNIAAATGLVKKDQLAKQNEEGISQEGMMDNT-DMPVDPDNEAYEMPPEEEYQDYEPEA

>gr.seaturtle Chelonia mydas

MDVFMKGLSKAKEGVVAAAEKTKQGVAEAAGKTKEGVLYVGSRTRDGVVHGVTTVAEKTKEQVSNVGGAVVTGVTAVAQKTVEGAGTIPPATGLVKKDQLAKQNEEGISQEGMMDNT-DMPVDPDNEAYEMPPEEEYQDYEPEA

>frog Xenopus laevis

MDVFMKGLSKAKEGVVAAAEKTKQGVAEAAGKTKEGVLYVGSKTKEGVVHGVTTVAEKTKEQVSNVGGAVVTGVTAVAHKTVEGAGNFAAATGLVKKDQ--K-NESGFGPEGTMENSENMPVNPNNETYEMPPEEEYQDYDPEA

>axolotl

MDVFMKGLSKAKEGVVAAAEKTKQGVAEAAGKTKESVLYMGSKTRDGVVHGVTTVAEKTKEQVSHVGGAMVTGVTAVAHKTVEGAGNIAAATGLVRKDQMAKQNDDGFIHEGTMSNADNTLVDPDNEAYEMPPEEEYQDYDRQA

>coelacanth Latimeria chalumnae

MDMLMKGLSKAKEGVVAAAEKTKQGVAEAAGKTKEGVLYMGSKTKEGVVQGVTTVAEKTKEQVSNVGGAVVTGVTAVAQKTVEGAGNIAAATGLVKKDQLNKENEEGVHQKGMIENTENLPLDPASENYEMPPEEEYQDYQPDA

Glucocerebrosidase

>mouse

AQPCIPKSFGYS-SVVCVCNASYCDSLDPVTL--PALGTFSRYESTRRGRRMELSVGAIQANR-TGTGLLLTLQPEKK---FQKVKGFGGAMTDATALNILALSPPTQKLLLRSYFST-NGIEYNIIRVPMASCDFSIRVYTYADTPNDFQLSNFSLPEEDTKLKIPLIHQALKMSSRPISLFASPWTSPTWLKTNGRVNGKGSLKGQPGDIFHQTWANYFVKFLDAYAKYGLRFWAVTAENEPTAGL-FTGYPFQCLGFTPEHQRDFISRDLGPALANSSH-DVKLLMLDDQRLLLPRWAEVVLSDPEAAKYVHGIAVHWYMDFLAPAKATLGETHRLFPNTMLFASEACVGSKFWE--QSVRLGSWDRGMQYSHSIITNLLYHVTG--------WTDWNLALNPEGGPNWVRNFV--DSPIIVDIPKDAFYKQPMFYHLGHFSKFIPEGSQRVALVASES---TDLETVALLRPDG--SAVVVVLNRSSEDVPLTISDPD-LGFLETVSPGYSIHTYLWRRQ--

>rat

AQPCIPKSFGYS-SVVCVCNATYCDSLDPLTL--PALGTFSRYESTRSGRRMELSTGSIQANR-TGTGLLLTLQPEEK---FQKVKGFGGAMTDATALNILALSPPAQKLLLKSYFSS-EGIEYNIIRVPMASCDFSIRIYTYADTPNDFQLSNFSLPEEDTKLKIPLIHRALKMSPRPISLFASPWTSPTWLKTNGAVNGKGSLKGHPGDIYHEAWANYFVKFLDAYATHNIKFWAVTAENEPSAGL-FTGYPFQCLGFTAEHQRDFISHDLGPALANSSH-DVKLLILDDQRLLLPRWAQVVLSDPEAAKYVHGIAVHWYMDFLAPAKATLGETHRLFPNMMLFASEACVGSKFWE--QSVRLGSWDRGMQYSHSIITNLLYHVTG--------WTDWNLALNPEGGPNWVRNFV--DSPIIVDIPKDTFYKQPMFYHLGHFSKFIPEGSQRVGLVASEK---TDLETVALIRPDG--SAVVVVLNRSSKDVPLTISDPA-LGFMETISPGYSIHTYLWRRQ--

>g.hamster

AQPCIPKSFGYS-SVVCVCNATYCDSLDPLTL--PAAGSFSRYESTRSGRRMELSVGSIQANR-TGTGLLLTLQPEER---FQKVKGFGGAMTDATALNILALSPSTQNLLLKSYFSS-EGIEYNIIRVPMASCDFSIRIYTYADTPNDFQLSNFSLPEEDTKLKIPLIHRALEMSPRPISLFASPWTSPTWLKTNGAVNGKGSLKGQPGDIYHQTWANYFVKFLDAYARHKLKFWAVTVENEPSAGL-ITGYPFQCLGFTAEQQRDFIARDLGPALSNSSH-DVQLFMLDDQRLLLPRWAQVVLSDPNAAKYVHGIAVHWYMDFLAPAKLTLGETHRLFPNTTLFATEACVGSKFWE--QSVRLGSWGRGMQYSHSIITNLLYHVAG--------WTDWNLALNPEGGPNWVRNFV--DSPIIVDIPKDTFYKQPMFYHLGHFSKFIPEGSQRVGLVASEK---NDLDTVALIRPDG--SAVVVVLNRSSKDVPLTISDPG-LGFLETISPSYSIHTYLWRRQ--

>c.hamster

AQPCIPKSFGYS-SVVCVCNATYCDSLDPLTL--PAAGTFSRYESTRSGRRMELSVGSIQANR-TGTGLLLTLRPEER---FQKVKGFGGAMTDATALNILALSPSAQNLLLKSYFSS-EGIEYNIIRVPMASCDFSIRIYTYADTPNDFQLSNFSLTEEDTKLKIPLIHRALKMSPRPISLLASPWTSPTWLKTNGAVNGKGSLKGQAGDVYHRTWANYFVKFLDAYAMYKIKFWAVTVENEPSAGL-ITGYPFQCLGFTAEQQRDFIARDLGPALTNSSH-DVQLFMLDDQRLLLPRWAQVVLSDPEAAKYVHGIAVHWYMDFLAPAKPTLGETHRLFPNTTLFASEACVGSKFWE--QSVRLGSWSRGMQYSHSIITNLLYHVAG--------WTDWNLALNPEGGPNWVRNFV--DSPIIVDIPKDTFYKQPMFYHLGHFSKFIPEGSQRVGLVASEK---NDLDTVALIRPDG--SAVVVVLNRSSKDVPLTISDPG-LGFLETISPSYSIHTYLWRRQ--

>jerboa

ARPCVPKSFGYS-SVVCVCNATYCDSLDPLTL--PAPGTFSRYESTRSGRRMELSVGKIQANR-TGSGLLLTLQPEEK---FQKVKGFGGAMTDAAALNILSLSPAAQDLLLKSYFSK-EGIEYNIIRVPMASCDFSIRIYTYADTPDDFQLNNFSLPEEDTKLKIPLIHRALQLAQRPVSLFASPWTAPTWLKTNGAVNGKGTLKGQPGDIYHQTWANYFVKFLTAYAGYKLKFWAVTVENEPSAGL-ITGYPFQCLGFTAEHQRDFIARDLGPILANSTHRDIKLLMLDDQRLLLPRWAQVVLADPEAAKYVHGIAVHWYLDFLAPAKATLGETHRLFPNTMLFASEACVGSKFWE--QSVRLGSWDRGTRYSHSIITNLLYYVAG--------WTDWNLALNPEGGPNWVRNFV--DSPIIVDTAKDTFYKQPMFYHLGHFSKFIPEGSQRVGLVASEE---HDLDTVALMRPDG--SVVVVVLNRSSKDVPLTISDPA-LGFLETISPGYSIHTYLWRRQ--

>human

ARPCIPKSFGYS-SVVCVCNATYCDSFDPPTF--PALGTFSRYESTRSGRRMELSMGPIQANH-TGTGLLLTLQPEQK---FQKVKGFGGAMTDAAALNILALSPPAQNLLLKSYFSE-EGIGYNIIRVPMASCDFSIRTYTYADTPDDFQLHNFSLPEEDTKLKIPLIHRALQLAQRPVSLLASPWTSPTWLKTNGAVNGKGSLKGQPGDIYHQTWARYFVKFLDAYAEHKLQFWAVTAENEPSAGL-LSGYPFQCLGFTPEHQRDFIARDLGPTLANSTHHNVRLLMLDDQRLLLPHWAKVVLTDPEAAKYVHGIAVHWYLDFLAPAKATLGETHRLFPNTMLFASEACVGSKFWE--QSVRLGSWDRGMQYSHSIITNLLYHVVG--------WTDWNLALNPEGGPNWVRNFV--DSPIIVDITKDTFYKQPMFYHLGHFSKFIPEGSQRVGLVASQK---NDLDAVALMHPDG--SAVVVVLNRSSKDVPLTIKDPA-VGFLETISPGYSIHTYLWRRQ--

>chimp

ARPCIPKSFGYS-SVVCVCNATYCDSFDPPTF--PALGTFSRYESTRSGRRMELSMGTIQANH-TGTGLLLTLQPEQK---FQKVKGFGGAMTDAAALNILALSPPAQNLLLKSYFSE-EGIGYNIIRVPMASCDFSIRTYTYADTPDDFQLHNFSLPEEDTKLKIPLIHRALQLAQRPVSLLASPWTSPTWLKTNGAVNGKGSLKGQPGDIYHQTWARYFVKFLDAYAEHKLQFWAVTAENEPSAGL-LSGYPFQCLGFTPEHQRDFIARDLGPTLANSTHHNVRLLMLDDQRLLLPHWAKVVLTDPEAAKYVHGIAVHWYLDFLAPAKATLGETHRLFPNTMLFASEACVGSKFWE--QSVRLGSWDRGMQYSHSIITNLLYHVVG--------WTDWNLALNPEGGPNWVRNFV--DSPIIVDITKDTFYKQPMFYHLGHFSKFIPEGSQRVGLVASQK---NDLDAVALMHPDG--SAVVVVLNRSSKDVPLTIKDPA-VGFLETISPGYSIHTYLWRRQ--

>gibbon

ARPCIPKSFGYS-SVVCVCNATYCDSLDPPTF--PALGTFSRYESTRSGRRMELSTGAIQANH-TGTGLLLTLQPEQK---FQKVKGFGGAMTDAAALNILALSPPAQNLLLKSYFSE-EGIGYNIIRVPMASCDFSIRTYTYADTPDDFQLHNFSLPEEDTKLKIPLIHRALQLAQRPVSLLASPWTSPTWLKTNGAVNGKGSLKGQPGDIYHQTWARYFVKFLDAYAEHKLQFWAVTAENEPSAGL-LSGYPFQCLGFTPEHQRDFIARDLGPTLANSTHHNVRLLVLDDQRLLLPHWAKVVLTDPEAAKYVHGIAVHWYLDFLAPAKATLGETHRLFPNTMLFASEACVGSKFWE--QSVRLGSWDRGMQYSHSIITNLLYHVVG--------WTDWNLALNPEGGPNWVRNFV--DSPIIVDITKDTFYKQPMFYHLGHFSKFIPEGSQRVGLVASQK---NDLDTVALMHPDG--SAVVVVLNRSSKDVPLTIKDPA-VGFLETISPGYSIHTYLWRRQ--

>orangutan

ARPCIPKSFGYS-SVVCVCNATYCDSLDPLTF--PALGTFSRYESTRSGRRMELSTGTIQANH-TGTGLLLTLQPEQK---FQKVKGFGGAMTDAAALNILALSPPAQNLLLKSYFSE-EGIGYNIIRVPMASCDFSIRTYTYADTPDDFQLHNFSLPEEDTKLKIPLIHRALQLARRPVSLLASPWTSPTWLKTNGAVNGKGSLKGQPGDIYHQTWARYFVKFLDAYAEHKLQFWAVTAENEPSAGL-LSGYPFQCLGFTPEHQRDFIARDLGPTLANSTHHNVRLLMLDDQRLLLPHWAKVVLTDPEAAKYVHGIAVHWYLDFLAPAKATLGETHHLFPNTMLFASEACVGSKFWE--QSVRLGSWDRGMQYSHSIITNLLYHVVG--------WTDWNLALNPEGGPNWVRNFV--DSPIIVDITKDTFYKQPMFYHLGHFSKFIPEGSQRVGLVASQK---NDLDTVALMHPDG--SAVVVVLNRSSKDVPLTIKDPA-VGFLETISPGYSIHTYLWRRQ--

>baboon

ARPCIPKSFGYS-SVVCVCNATYCDSLEPLTF--PALGTFSRYESTRSGRRMELSTGTIQANR-TGTGLLLTLQPEQK---FQKVKGFGGAMTDAAALNILALSPPAQNLLLKSYFSE-EGIGYNIIRVPMASCDFSIRTYTYADTPDDFQLHNFSLPEEDTKLKIPLIHRALQLAQRPVSLLASPWTSPTWLKTNGAVNGKGSLKGQPGDIYHQTWARYFVKFLDAYAEHKLQFWAVTAENEPSAGL-LSGYPFQCLGFTPEHQRDFIARDLGPALANSTHHNVRLLMLDDQRLLLPHWAKVVLTDPEAAKYVHGIAVHWYLDFLAPAKATLGETHRLFPNTMLFASEACVGSKFWE--QSVRLGSWDRGMQYSHSIITNLLYHVVG--------WTDWNLALNPEGGPNWVRNFV--DSPVIVDITKDTFYKQPMFYHLGHFSKFIPEGSQRVGLVASQK---NDLDTVALMHPDG--SAVVVVLNRSSKDVPLTIKDPA-VGFLETISPGYSIHTYLWRRQ--

>rhesus

ARPCIPKSFGYS-SVVCVCNATYCDSLEPLTF--PALGTFSRYESTRSGRRMELSTGTIQANR-TGTGLLLTLQPEQK---FQKVKGFGGAMTDAAALNILALSPPAQNLLLKSYFSE-EGIGYNIIRVPMASCDFSIRTYTYADTPDDFQLHNFSLPEEDTKLKIPLIHRALQLAQHPVSLLASPWTSPTWLKTNGAVNGKGSLKGQPGDIYHQTWARYFVKFLDAYAEHKLQFWAVTAENEPSAGL-LSGYPFQCLGFTPEHQRDFIARDLGPTLANSTHHNVRLLMLDDQRLLLPHWAKVVLTDPEAAKYVHGIAVHWYLDFLAPAKATLGETHRLFPNTMLFASEACVGSKFWE--QSVRLGSWDRGMQYSHSIITNLLYHVVG--------WTDWNLALNPEGGPNWVRNFV--DSPVIVDITKDTFYKQPMFYHLGHFSKFIPEGSQRVGLVASQK---NDLDTVALMHPDG--STVVVVLNRSSKDVPLTIKDPA-VGFLETISPGYSIHTYLWCLQ--

>greenmonkey

ARPCIPKSFGYS-SVVCVCNATYCDSLEPLTF--PALGTFSRYESTRSGRRMELSTGTIQANR-TGTGLLLTLQPEQK---FQKVKGFGGAMTDAAALNILALSPPAQNLLLKSYFSE-EGIGYNIIGVPMASCDFSIRTYTYADTPDHFQLHNCSLPEEDTKLKIPLIHRALQLAQRPVSLLASPWTSPTWLKTNGAVNGKGSLKGQPGDIYHQTWARYFVKFLDAYAEHKLQFWAVTAENEPSAGL-LSGYPFQCLGFTPEHQRDFIARDLGPTLANSTHHNVRLLMLDDQRLLLPHWAKVVLTDPEAAKYVHGIAVHWYLDFLAPAKATLGETHRLFPNTMLFASEACVGSKFWE--QSVRLGSWDRGMQYSHSIITNLLYHVVR--------WTDWNLPHNPEGGPNWVRNFV--DSPVIVDITKDTFYKQPMFYHLGHFSKFIPEGSQRVGLVASQK---NDLDTVALMHPDG--SAVVVVLNRSSKDVPLTIKDPA-VGFLETISPGYSIHTYLWRRQ--

>sq.monkey

ARPCIPKSFGYS-SVVCVCNATYCDSVDPLTF--PALGTFSRYESTRSGRRMELSTGTIQANR-TGTGLLLTLQPEQK---FQKIKGFGGAMTDAAALNILALSLPAQNLLLKSYFSE-EGIEYNIIRVPMASCDFSIRVYTYADTPDDFQLYNFSLPEEDTRLKIPLIHRALQLSHRPISLFASPWTSPTWLKTNGAVNGKGSLKGQPGSIYHQTWARYFVKFLDAYAEHKLQFWAVTAENEPSAGL-FSGYPFQCLGFTPEHQRDFIARDLGPTLANSTHRNVRLLMLDDQRLLLPHWAQVVLTDPEAAQYVDGIAVHWYLDFLAPAKATLGETHRLFPNTMLFASEACVGSKFWE--QSVRLGSWDRGMQYSHSIITNLLYHVVG--------WTDWNLALNPEGGPNWVRNFV--DSPIIVDITKDTFYKQPMFYHLGHFSKFIPEGSQRVGLVASEK---NDLDTVALMHPDG--SAVVVVLNRSSKDVPLTIEDPA-VGFLETISPGYSIHTYVWRRQ--

>marmoset

ARPCIPKSFGYS-SVVCVCNATYCDSVDPLTF--PALGTFSRYESTRSGRRMELSTGTIQANR-TGTGLLLTLQPEQK---FQKIKGFGGAMTDAAALNILALSLPAQNLLLKSYFSE-EGIEYNIIRVPMASCDFSIRIYTYADTPDDFQLYNFSLPEEDTRLKIPLIHRALQLSQRPISLCASPWTSPTWLKTNGAVNGKGSLKGQPGDTYHQTWARYFVKFLDAYAEHKLQFWAVTAENEPSAGL-LSGYPFQCLGFTPEHQRDFIARDLGPTLANSTHRNVRLLMLDDQRLLLPHWAQVVLTDPEAAKYVDGIAVHWYLDFLAPAKATLGETHRLFPNTMLFASEACVGSKFWE--QSVRLGSWDRGTQYSHSIITNLLYHVVG--------WTDWNLALNPEGGPNWVRNFV--DSPIIVDITKDTFYKQPMFYHLGHFSKFIPEGSQRVGLVASEK---NDLEAVALMHPDG--SAVVVVLNRSSKDVPVTIEDPA-VGFLETISPGYSIHTYLWRRQ--

>treeshrew

ARPCIPKSFGYS-SVVCVCNATYCDSLDPLTF--PALGTFSRYESTRSGRRMELSTGTMQANR-TGTGLLLTLKPEQK---FQTLKGFGGAMTDAAALNILALSPPVQNLLLKSYFSE-EGIEYNIIRVPMASCDFSIRTYTYADTPDDFQLHNFSLPDEDTKMKIPLIHRALRLAQRPVSLLASPWTSPTWLKTNGAVNGKGSLKGQPGDIYHQTWANYFVRFLDAYAEYKLQFWAVTAENEPSAGL-ISGYPFQCLGFTPEHQRDFIARDLGPTLANSTHRNVRLLMLDDQRLLLPRWAQVVLADPEAAKYVHGIAVHWYLDFLAPAKATLGETHRLFPDTMLFASEACVGSKFWE--QSVRLGSWDRGMQYSHSIITNLLYHVVG--------WTDWNLALNPEGGPNWVRNFV--DSPIIVDIAKDTFYKQPMFYHLGHFSKFIPEGSQRVGLDASEK---NDLEAVALTHPDG--SAVVVVLNRSSKDVPLTIKDPA-VGFLETMSPGYSIQTYLWRRQ--

>galago

ARPCIPKSFGYS-SVVCVCNATYCDSLDPLTL--PAPGTFSRYESTRSGRRMELSTGTIEANH-TGTGLLLTLQPEQR---FQKMKGFGGAMTDAAALNILALSPPAQDLLLKSYFSE-EGIEYNIIRVPMASCDFSIRIYTYADTPDDFQLHNFSLPEEDTKLKIPLIHRALKLSQRHVSLFASPWTSPTWLKTNGAVNGKGSLKGQPGDIYHQSWAKYFVKFLDAYAEQKLQFWAVTAENEPSAGL-MSGYPFQCLGFTPEHQRDFIARDLGPTLANSTHRNVHLLMLDDNRLLLPHWAQVVLKDPEAAKYVHGIAVHWYLDFLAPAEATLGETHRLFPNTMLFASEACVGSKFWE--QSVRLGSWDRGMQYSHSIITNLLYHVVG--------WTDWNLALNPEGGPNWVRNFV--DSPIIVDINKDTFYKQPMFYHLGHFSKFIPEGSQRVGLVASEK---SHLDTVALIHPDGS-AAVAVVLNRSSKDVPLTIKDPA-LGFLKTISPGYSIQTYLWRRQ--

>orca

ARPCSPKSFGYS-SVVCVCNATYCDSLDPLAL--PDPGTFSRFESTRSGRRMELSLGTIQANR-TGTGLLLTLQPDQK---FQKVKGFGGAVTDAAALNILALSPPARDLLLKSYFSK-EGIEYNIMRVPMASCDFSIRTYTYADTPDDFQLLNFSLPEEDVKLKIPLIHRALELAQRPVSLFASPWTSPTWLKTNGAVNGKGTLKGHPGDLYHQTWARYFVKFLDAYAEHKLQFWAVTAENEPSAGL-FSGYPFQCLGFTPEHQRDFIARDLGPTLANSTHRNVRLLMLDDQRLLLPHWAQVVLADPEAAKYVHGIAVHWYLDFLAPAKATLGETHRLFPDTMLFASEACVGSKFWE--QSVRLGSWDRGMQYSHSIITNLLYHVVG--------WTDWNLALNPEGGPNWVRNFV--DSPIIVDIAKDTFYKQPMFYHLGHFSKFIPEGSQRVGLVASEK---NDLDTVALIHPDG--SAVVVVLNRSSKDVPLTIKDPA-VGFVEAISPGYSIHTYLWRRQ--

>baiji

ARPCSPKSFGYS-SVVCVCNATYCDSLDPLTL--PDPGTFSCFESTRSGRRMELSLGTIQANR-TGTGLLLTLQPDQK---FQKVKGFGGAVTDAAALNILALSPPARDLLLKSYFSK-EGIEYNIMRVPMASCDFSIRTYTYADTPDDFQLLNFSLPEEDVKLKIPLIHQALELAQRPVSLFASPWTSPTWLKTNGAVNGKGTLKGHPGDLYHQTWARYFVKFLDAYAEHKLQFWAVTAENEPSAGL-FSGYPFQCLGFTPEHQRDFIARDLGPTLANSTHRNVRLLMLDDQRLLLPHWAQVVLADPEAAKYVHGIAVHWYLDFLAPAKATLGETHRLFPDTMLFASEACVGSKFWE--QSVRLGSWDRGMQYSHSIITNLLYHVVG--------WTDWNLALNPEGGPNWVRNFV--DSPIIVDIAKDTFYKQPMFYHLGHFSKFIPEGSQRVGLVASEK---NDLDTVALIHPDG--SAVVVVLNRSSMDVPLTIKDPA-VGFVETLSPGYSIHTYLWRRQ--

>minkewhale

ARPCSPKSFGYS-SVVCVCNATYCDSLDPLTL--PDPGTFSRFESTRSGRRMELSLGTIQANR-TGTGLLLTLQPDQK---FQKVKGFGGAMTDAAALNILALSPPARDLLLKSYFSK-EGIEYNIMRVPMASCDFSIRTYTYADTPDDFQLLNFSLPEEDVKLKIPLIHQALELAQRPVSLFASPWTSPTWLKTNGAVNGKGTLKGHPGDLYHQTWARYFVKFLDAYAEHKLQFWAVTAENEPSAGL-FSGYPFQCLGFTPEHQRDFIARDLGPTLANSTHRNVRLLMLDDQRLLLPHWAQVVLADPEAAKYVHGIAVHWYLDFLAPAKATLGETHRLFPDTMLFASEACVGSKFWE--QSVRLGSWDRGVQYSHSIITNLLYHVVG--------WTDWNLALNPEGGPNWVRNFV--DSPIIVDIAKDTFYKQPMFYHLGHFSKFIPEGSQRVGLVASEK---NDLDTVALIHPDG--SAVVVVLNRSSKDVPLTIKDPA-VGFVETISPGYSIHTYLWRRQ--

>pig

ARPCSPKSFGYS-SVVCVCNATYCDSLDPLTL--PDPGTFSRFESTRSGRRMELSLGTFQANR-TSKGLLLTLQPDQK---FQKVKGFGGAMTDAAALNILALSPQARNLLLKSYFSE-EGIEYNIIRVPMASCDFSIRIYTYADTPDDFQLLNFSLPEEDVKLKIPLIHQALKMAQRPVSLFASPWTSPTWLKTNGAVNGKGTLKGHPGDRYHQTWAKYFVKFLDAYAEHNLHFWAVTAENEPSAGL-FTGYPFQCLGFTPEHQRDFIARDPGPTLANSTHRNVRLLMLDDQRLLLPHWAQVVLADPEAAKYVHGIAVHWYLDFLAPAKATLGETHRLFPNTMLFASEACVGSKFWE--QSVRLGSWDRGVQYSHSIITNLLYHVVG--------WTDWNLALNPEGGPNWVRNFV--DSPIIVDISKDTFYKQPMFYHLGHFSKFIPEGSQRVGLAASEK---NNLDTVALLRPDG--SAVVVVLNRSSKDVPLTIKDPA-LGFLETISPSYSIHTYLWHRK--

>panda

ARPCSPRSFGYS-SVVCMCNATYCDSLDPLTP--PAPGTFSRYESTRSGRRMELSLGTIQANR-TGTGLLLTLQPDQK---FQKVKGFGGAMTDAAALNILALSPPARNLLLKSYFSE-EGIEYNIIRVPMASCDFSVRTYTYDDSPGDFQLRNFSLPEEDVALKIPLIHQALELARRPVSLFASPWTSPTWLKTNGAVNGKGSLKGQPGDLYHQTWARYFVKFLDAYAEHRLRFWAVTAENEPSAGL-LSGYPFQCLGFTPEHQRDFIARDLGPALANSTHHDIRLLILDDQRLLLPHWARVVLADPEAAKYVHGIAVHWYLDFLAPAKATLGETHRLFPNTMLFASEACVGSKFWE--QSVRLGSWDRGVQYSHSIITNLLYHVAG--------WTDWNLALNLEGGPNWVRNFV--DSPIIVDIAKDTFYKQPMFYHLGHFSKFIPEGSQRVGLQASKR---NGLDTVALMRPDG--SAVVVVLNRSPKDVPLTIEDPA-VGFVETLSPGYSIHTYVWHRQ--

>ferret

ARPCIPKSFGYS-SVVCVCNATYCDSLDPLTL--PAPGTFSRYESTRSGRRMERSLGTIRANR-TGTGLLLTLQPDQK---FQKVKGFGGAMTDAAALNILALSPPARDLLLKSYFSE-EGIEYNIIRVPMASCDFSVRTYTYDDTPDDFQLRDFSLPEEDVKLKIPLIHQALALARRPVSLFASPWTSPTWLKTNGAVNGKGSLKGQPGDIYHQTWARYFVKFLDVYAEHKLRFWAVTAENEPSAGL-LSGYPFQCLGFTPEHQRDFIARDLGPALANSTHRDTRLLILDDQRLLLPHWAQVVLADPEAAKYVHGVAVHWYLDFLAPAKATLGETHRLFPDIMLFASEACVGSKFWE--QSVRLGSWDRGVQYSHSIITNLLYHVAG--------WTDWNLALNPEGGPNWVRNFV--DSPIIVDIAKDTFYKQPMFYHLGHFSKFIPEGSQRVGLLASQK---NSLDTVALTRPDG--SAVVVVLNRSSKDVPLTIEDPG-VGFVETLSPGHSIHTYLWSRP--

>cat

ARPCSPKSFGYS-SVVCVCNATYCDSLDPLTL--PAPGTFGRYESTRSGRRMELSLGPIRANR-TGTGLLLTLQPDQK---FQKVKGFGGAMTDAAALNILALSPPARNSLLKSYFSE-EGIEYNIIRVPMASCDFSIRTYTYDDSPDDFQLRDFILPEEDVKLKIPLIHQALELAPRPVSLFASPWTSPTWLKTNGAVNGKGSLKGQPGDLYHQTWARYFVKFLDAYAEHKLRFWAVTAENEPSAGL-FSGYPFQCLGFTPEHQRDFIARDLGPTLANSTHRDVRLLILDDQRLLLPRWAQVVLADPEAAKYVHGIAVHWYLDFLAPAKATLGETHRLFPDTMLFASEACVGSKFWE--QSVRLGSWDRGMQYSHSIITNLLYHVVG--------WTDWNLALNPEGGPNWVRNFV--DSPIIVDITKDTFYKQPMFYHLGHFSKFIPEGSQRVGLLASEK---NGLDTVALTRPDG--SAVLVVLNRSPKDVPLTIEDPA-VGFMETLSPGYSIHTYLWRRQ--

>dog

ARPCIPKSFGYS-SVVCVCNATYCDSLDPLAL--PAPGTFSQYESTRSGRRMELSLGTIRANH-TGTGLLLTLQPDQK---FQKVKGFGGAMTDAAALNILALSPPARNLLLKSYFSA-EGIEYNIIRVPMASCDFSIRTYTYDDTHDDFQLHNFSLPEEDIKLKIPLIHQALELAQHPVSLFASPWTSPTWLKTNGAVNGKGSLKGQPGDLYHQTWARYFVKFLDAYAEHKLQFWAVTAENEPSAGL-FSGYPFQCLGFTPEHQRDFIARDLGPALANSTHHDIRLLILDDQRLLLPHWAQVVLADPEAAKYVHGIAVHWYLDFLAPAKATLGETHRLFPNTMLFASEACVGSKFWE--QSVRLGSWDRGVQYSHSIITNLLYHVVG--------WTDWNLALNLEGGPNWVRNFV--DSPIIVDITKDVFYKQPMFYHLGHFSKFIPEGSQRVGLAASKK---NGLDTVALTRPDG--SAVVVVLNRSSKDVPLTIKDPA-VGFVETLSPAYSIHTYLWRRQ--

>cow

ARPCSPKSFGYS-SVVCVCNGTYCDSLDPLTL--PDPGTFSRFESTRSGRRMELSLGTIQANR-TGTGLLLTLQPDQK---FQKVKGFGGAMTDAAALNILALSPAARNLLLKSYFSE-EGIEYNIIRVPMASCDFSIRTYTYDDSPDDFQLLNFSLPEEDVKLKIPLIHQALELANRSVSLFASPWTSPTWLKTNGAVNGKGTLKGQAGDLYHKTWARYFVKFLDAYAEHKLRFWAVTAENEPTAGL-LTGYPFQCLGFTPEHQRDFIARDLGPILANSTHRDVRLLMLDDQRLLLPRWAQVVLADPEAAKYVHGIAVHWYLDFLAPAKATLGETHRLFPNTMLFASEACVGSKFWE--QSVRLGSWDRGMRYSHSIITNLLYHVVG--------WTDWNLALNPEGGPNWVRNFV--DSPIIVDIAKDTFYKQPMFYHLGHFSKFIPEGSQRVGLVASKK---SDLDTVALLRPDG--SAVAVVLNRSSKDVPLTIKDPA-VGFMETVSPGYSIHTYLWRRQ--

>yak

ARPCSPKSFGYS-SVVCVCNGTYCDSLDPLTL--PDPGTFSRFESTRSGRRMELSLGTIQANR-TGTGLLLTLQPDQK---FQKVKGFGGAMTDAAALNILALSPAARNLLLKSYFSEEGGIEYNIIRVPMASCDFSIRTYTYDDSPDDFQLLNFSLPEEDVKLKIPLIHQALELANRSVSLFASPWTSPTWLKTNGAVNGKGTLKGQAGDLYHKTWARYFVKFLDAYAEHKLRFWAVTAENEPTAGL-LTGYPFQCLGFTPEHQRDFIARDLGPILANSTHRDVRLLMLDDQRLLLPRWAQVVLADPEAAKYVHGIAVHWYLDFLAPAKATLGETHRLFPNTMLFASEACVGSKFWE--QSVRLGSWDRGMRYSHSIITNLLYHVVG--------WTDWNLALNPEGGPNWVRNFV--DSPIIVDIAKDTFYKQPMFYHLGHFSKFIPEGSHRVGLVASKK---SDLDTVALLRPAG--SAVGVVP--SSKDVPLTIKDPA-VGFMETVSPGYSIHTYLWRRQ--

>waterbuffalo

ARPCSPKSFGYS-SVVCVCNGTYCDSLDPLTL--PDPGTFSRFESTRSGRRMELSLGTIQANR-TGTGLLLTLQPDQK---FQKVKGFGGAMTDAAALNILALSPAARNLLLKSYFSE-EGIEYNIIRVPMASCDFSIRTYTYDDSPDDFQLLNFSLPEEDVKLKIPLIHQALELANRSVSLFASPWTSPTWLKTNGAVNGKGTLKGQPGDLYHKTWARYFVKFLDAYAEHKLRFWAVTAENEPTAGL-LTGYPFQCLGFTPEHQRDFIARDLGPILANSTHRDVRLLMLDDQRLLLPRWAQVVLADPEAAKYVHGIAVHWYLDFLAPAKATLGETHRLFPNTMLFASEACVGSKFWE--QSVRLGSWDRGMRYSHSIITNLLYHVVG--------WTDWNLALNPEGGPNWVRNFV--DSPIIVDIAKDTFYKQPMFYHLGHFSKFIPEGSQRVGLVATKK---SDLDVVALLRPDG--SAVAVVLNRSSKDVPLTIKDPA-VGFMETVSPGYSIHTYLWRRQ--

>sheep

ARPCIPKSFGYS-SVVCVCNATYCDSLDPLTL--PDPGTFSRFESTRSGRRMELSLGTVQANR-TGTGLLLTLQPDQK---FQKVKGFGGAMTDAASLNILALSPAARNLLLKSYFSE-EGIEYNIIRVPMASCDFSIRTYTYDDSPDDFQLLNFSLPEEDVKLKIPLIHQALELANRSVSLFASPWTSPTWLKTNGAVNGKGTLKGQPGDLYHKTWARYFVKFLDAYAEHKLRFWAVTAENEPTAGL-LSGYPFQCLGFTPEHQRDFIARDLGPILANSTHRDVRLLMLDDQRLLLPRWAQVVLADPEAAKYVHGIAVHWYLDFLAPAKATLGETHRLFPNTMLFASEACVGSKFWE--QSVRLGSWDRGMQYSHSIITNLLYHVVG--------WTDWNLALNPEGGPNWVRNFV--DSPIIVDIAKDTFYKQPMFYHLGHFSKFILEGSQRVGLVASEK---SDLDTVALFRPDG--SAVGVVLNRSSKDVPLTIKDPA-VGFMETVSPGYSIHTYLWRRQ--

>goat

ARPCIPKSFGYS-SVVCVCNATYCDSLDPLTL--PDPGTFSRFESTRSGRRMELSLGTIQANR-TGTGLLLTLQPDQK---FQKVKGFGGAVTDAASLNILALSPAARNLLLKSYFSE-EGIEYNIIRVPMASCDFSIRTYTYDDSPDDFQLLNFSLPEEDVKLKIPLIHQALELANRSVSLFASPWTSPTWLKTNGAVNGKGTLKGQPGDLYHKTWARYFVKFLDAYAEHKLRFWAVTAENEPTAGL-LSGYPFQCLGFTPEHQRDFIARDLGPILANSTHRDVRLLMLDDQRLLLPRWAQVVLADPEAAKYVHGIAVHWYLDFLAPAKATLGETHRLFPNTMLFASEACVGSKFWE--QSVRLGSWDRGMQYSHSIITNLLYHVVG--------WTDWNLALNPEGGPNWVRNFV--DSPIIVDIAKDTFYKQPMFYHLGHFSKFIPEGSQRVGLVASEK---SDLDTVALFRPDG--SAVAVVLNRSSKDVPLTIKDPA-VGFMETVSPGYSIHTYLWRRQ--

>rhino

ARPCSPKTFGYS-SVVCVCNATYCDSLDPLTL--PATGTFSRYESTRSGRRMELSQGTIQANR-TGTGLLLTLQPDQK---FQKVKGFGGAMTDAAALNILALSPPARDLLLKSYFSE-EGIEYNIIRVPMASCDFSIRIYTYADTPDDFQLHNFSLPEEDVKLKIPLIHQALNLAQRPVSLFASPWTSPTWLKTNGKVNGKGSLKGQPGDLYHQTWARYFVKFLDAYAEHKLQFWAVTAENEPSAGL-ISGYPFQCLGFTPEHQRDFIARDLGPTLANSTHHNVRLLILDDQRLLLPRWAQVVLEDPEAAKYVHGIAVHWYLDFLAPAKATLGETHRLFPNTTLFASEACVGSKFWE--QSVRLGSWDRGMQYSHSIITNLLYHVVG--------WTDWNLALNPEGGPNWVRNFV--DSPIIVDITKDTFYKQPMFYHLGHFSKFIPEGSQRVGLVASEK---TNLDTVALMHPDG--SAVVVVLNRSSKDVPLTIKDPA-VGFLETISPGYSIQTYLWRRQ--

>camel

ARPCIPKSFGYS-SVVCVCNATYCDSLDPLTL--PDPGTFSRFESTRSGRRMELSLGNFQANR-TGTGLLLTLQPDQK---FQKVKGFGGAMTDAAALNILALSLPARNLLLKSYFSE-EGIEYNIIRVPMASCDFSIRVYTYADAPDDFQLLNFSLPEEDVKLKIPLIHQAQELAKRHISLFASPWTSPTWLKTNGKVNGKGSLKGHPGDLYHQTWARYFIKFLDAYAKHKLQFWAVTAENEPSAGL-FSGYPFQCLGFTPEHQRDFIARDLGPILANSTHRDVRLLILDDQRLLLPHWAQVVLTDPEAAKYIHGIAVHWYLDFLAPAKATLGETHRLFPDTMLFASEACVGSKFWE--QSVRLGSWDRGMQYSHSIITNLLYHVVG--------WTDWNLALNPEGGPNWVRNFV--DSPIIVDITKDTFYKQPMFYHLGHFSKFIPEGSQRVGLVASKK---NDLDTVALMHPDG--SAVVVVLNRSSKDVPLTIKDPA-MGFLETISPGYSIHTYLWRRQ--

>alpaca

ARPCIPKSFGYS-SVVCVCNATYCDSLDPLTL--PDPGTFSRFESTRSGRRMELSLGNFQANR-TGTGLLLTLQPDQK---FQKVKGFGGAMTDAAALNILALSPPARNLLLKSYFSE-EGIEYNIIRVPMASCDFSIRVYTYADAPDDFQLLNFSLPEEDVKLKIPLIHQAQELAKRHVSLFASPWTSPTWLKTNGKVNGKGSLKGHPGDLYHQTWARYFIKFLDAYAEHKLQFWAVTAENEPSAGL-FSGYPFQCLGFTPEHQRDFIARDLGPILANSTHRDVRLLILDDQRLLLPHWAQVVLTDPEAAKYIHGIAVHWYLDFLAPAKATLGETHRLFPDTMLFASEACVGSKFWE--QSVRLGSWDRGMQYSHSIITNLLYHVVG--------WTDWNLALNPEGGPNWVRNFV--DSPIIVDITKDTFYKQPMFYHLGHFSKFIPEGSQRVGLVASKK---NDLDTVALMHPDG--SAVVVVLNRSSKDVPLTIKDPA-MGFLETISPGYSIHTYLWRRQ--

>horse

ARPCSPKSFGYS-SVVCVCNATYCDSLDPLTL--PAPGTFSRYESTRSGRRMELSLGAIQANR-TGTGLLLTLQPDQK---FQKVKGFGGAMTDAAALNILALSPAARNLLLKSYFSE-EGIEYNIIRVPMASCDFSIRVYTYADTPDDFQLHNFSLPEEDVKLKIPLIHQALELSQRPISLFASPWTSPTWLKTNGAVNGKGSLKGQPGDRYHQTWAKYFVKFLDAYAEHKLQFWAVTTENEPSAGL-ISGYPFQCLGFTPEHQRDFIARDLGPTLANSTHRNVRVLMLDDQRLLLPRWAQVVLADPEAAKYVHGIAVHWYLDFLAPAKATLGETHRLFPDMMLFASEACVGSKFWE--QSVRLGSWDRGVQYSHSIITNLLYHVAG--------WTDWNLALNPEGGPNWVRNFV--DSPIIVDIAKDTFYKQPMFYHLGHFSKFIPEGSQRVGLDASEK---TNLDTVALMHPDG--SAVVVVLNRSSKDVPLTIKDPA-VGFLETVSPGYSIHTYLWRRQ--

>bat

ARPCNPKNFGYS-SVVCVCNATYCDSLDPLTL--PAPGTFTRYESTRSGRRMERSLGTIQANQ-TGTGLLLTLQPEQK---FQKIKGFGGAMTDAAALNILALSPPARNLLLKSYFSE-EGIEYNIIRVPMASCDFSIRIYTYADTPDDFQLHNFSLPEEDVKLKIPLIHQALEMSQRPVSLFASPWTSPTWLKTNGAVNGRGSLKGQPGDLYHQTWARYFVKFLDAYAEHKLQFWAVTAENEPSAGL-FSGYPFQCLGFTPEHQRDFIARDLGPTLANSAHRGVRLLMLDDQRLLLPHWAEVVLADPEAAKYVHGIAVHWYLDFLAPAKATLGETHRLFPDTMLFASEACVGSKFWE--QSVRLGSWDRGMQYSHSIITNLLYHVVG--------WTDWNLALNPEGGPNWVRNFV--DSPIIVDIAKDTFYKQPMFYHLGHFSKFIPEGSQRVGLVASEK---NDLEAVALMRPDG--SAVVVVLNRSSKDVPLTIKDPA-LGFLETVSPAYSIHTYLWRRQ--

>bigbrownbat

ARPCNPKNFGYS-SVVCVCNATYCDSLDPLTL--PAPGTFTRYESTRSGRRMELSLGTIQANH-SGTGLLLTLQPEQK---FQKIKGFGGAMTDAAALNILALSPPARNLLLKSYFSE-EGIEYNIIRVPMASCDFSIRIYTYADTPDDFQLHNFSLPEEDVKLKIPLIHQALEMSQRPVSLFASPWTSPTWLKTNGAVNGRGSLKGQPGDLYHQTWARYFVKFLDAYAEHKLQFWAVTAENEPSAGL-FSGYPFQCLGFTPEHQRDFIARDLGPTLANSTHRGVRLLMLDDQRLLLPHWAQVVLADPEAAKYVHGIAVHWYLDFLAPAKATLGETHRLFPDTMLFASEACVGSKFWE--QSVRLGSWDRGMQYSHSIITNLLYHVVG--------WTDWNLALNPEGGPNWVRNFV--DSPIIVDITKDTFYKQPMFYHLGHFSKFIPEGSQRVGLVASEK---TDLETVALTRPDG--SAVVVVLNRSSKDVPLTIKDPA-LGFLETVSPAYSIHTYLWRRQ--

>flyingfox

ARPCSPQSFGYS-SVVCVCNATYCDSLDPLTL--PAPGTFSRYESTRSGRRMELSLGTVQARR-TRTGLLLTLQPDQK---FQQIKGFGGAMTDAAALNMFALSPPARNLLLKSYFSE-EGIEYNIIRVPMASCDFSIRIYTYADTPDDFELHNFSLSEEDVKLKIPLIHQALEMAQRPVSLFASPWTAPTWLKTNGAVNGRGSLKGRPGDRYHQTWARYFIKFLDAYAEHRLRFWAVTAENEPSAGL-LSGYPFQCLGFTPEHQRDFIARDLGPTLANSTHRGVRLLMMDDQRVLLPHWAQVVLADPEAAKYVHGIAVHWYLDFLAPAKATLGETHRLFPDTMLFASEACAGFEFWE--QSVQLGSWDRGMQYSHSIITNLLYHVVG--------WTDWNLALNLEGGPNWVRNFV--DSPIIVDIAKDTFYKQPMFYHLGHFSKFIPEGSQRVGLAASEK---SDLDTVALVRPDG--SAVVVVLNRSSKDVPVTIQDPA-VGFLETISPAYSIHTYLWRRQ--

>mole

TRPCSPKSFGYS-SVVCVCNATYCDSLDPLTL--PALGTFSRYESTRSGRRMELSVGSFQANR-AGTGLLLTLQPEQK---FQRVKGFGGAMTDAAALNILGLSPPVRDLLLRSYFSE-EGIEYNIMRVPMASCDFSIRTYTYADTPDDFELQNFSLPDEDVKLKIPLIHQALQLTRRPISLFASPWTSPTWLKTNGAVNGKGTLKGQPGDRYHQTWAKYFVKFLDAYAEHKLKFWAVTAENEPSAGL-FSGYPFQCLGFTPEHQRDFIARDLGPTLANSTHRDVLLLMLDDQRLLLPHWAEVVLADPEAAKHVHGIAVHWYLDFLAPAKATLGETHRLFPNTMLFASEACVGSKFWE--QSVRLGSWDRGMQYSHSIITNLLYHVVG--------WTDWNLALNPEGGPNWVRNFV--DSPIIVDIAKDTFYKQPMFYHLGHFSKFIPEGSQRVGLVASEK---SDLETVALTHPDG--SAVVVVLNRSSKDVPLTIEDPA-LGFLETISPGYSIHTYLWRRQCQ

>elephantshrew

ARPCIPKSFGYS-SVVCVCNATYCDSLDPLAL--PASGTFSLYESTRSGRRMEMSMGTIQTSR-TGKGLLLTLEPEQK---FQKIKGFGGAITDAAALNILALSPAAQNLLLRSYFSE-EGIEYNIIRVPMGSCDFSIHTSTYDDVVDDFQLDNFSLLDEDVKLKIPLIHRALELTQRPISLFASPWTSPTWLKTNGAVNGKGSLKGHPGDIYHQTWAKYFVKFLDAYATYNIRFWAVTAENEPSAGL-FAGYPFQCLGFTPQHQRDFIAYDLGPALANSSHSDVRLLMLDDQRLLLPHWAQVVLENPEAAKYVHGIAVHWYLDYLAPAKATLGETHRLFPDMMLFSSEACVGSKFWE--QSVRLGSWDRGMQYSHSIITNLLYHVVG--------WTDWNIALNLEGGPNWVRNFV--DSPIIVDITKDTFYKQPMFYHLGHFSKFIPEGSQRVGLVASEK---TNLETVALIRPDG--SAVVVVLNRSSKDVPLTIKDPA-MGFLETMAPGYSIHTYLWRRQ--

>armadillo

ARPCVPKSFGYS-SVVCVCNATYCDSLDPLTL--PAPGTFSRYESTRSGRRMELSVGTFQANR-TGTGLLLTLHPEQK---FQKVKGFGGAMTDAAALNILALSPPAQNLLLKSYFSE-EGIEYNIIRVPMASCDFSIRVYTYADTPDDFQLHNFSLPEEDTKLKIPLIHRALDLTQRHISLFASPWTSPTWLKTNGAVNGRGTLKGQPGDIYHQTWANYFLKFLDAYAQHKLQFWAITAENEPSAGL-FAGYPFQCLGFTPEHQRDFIARDLGPALANSTHRNVLLLMLDDQRLLLPHWAQVVLADPEAAKYVHGIAVHWYLDFLAPAKATLGETHRLFPDTMLFASEACVGSKFWE--QSVRLGSWDRGMQYSHSIITNLLYHVAG--------WTDWNLALNPEGGPNWVRNFV--DSPIIVDIAKDTFYKQPMFYHLGHFSKFIPEGSQRVGLVASKN---NDLDTVALLHPDG--SAVLVVLNRSSKDVPLTIKDPA-VGFLETVAPGYSIHTYLWRRP--

>elephant

ARPCIPQSFGYS-SVVCVCNATYCDSLDPLTL--PTLGNFSLYESTRSGRRMELSMGTVQTSR-TGTGLLLTLQPEQK---FQKVKGFGGAMTDAAALNILALSPAVQNLLLKSYFSE-EGIEYNIIRVPMASCDFSIRIYTYADTPDDFQLCNFSLPEEDTKLKIPLIHRVLELTQRPISLFASPWTSPTWLKTNGAVNGKGTLKGQPGDIYHQTWARYFVKFLDAYAEHNLRFWAVTAENEPSAGL-FSGYPFQCLGFTPEHQRDFIARDLGPTLANSTHRDVRLLILDDQRLLLPHWAQVVLADAEAAKYVHGIAVHWYLDFLAPAKATLGATHRLFPNTMLFASEACVGSKFWE--QSVRLGSWNRGVQYSHSIITNLLYHVVG--------WTDWNLALNPEGGPNWVRNFV--DSPIIVDITKDTFYKQPMFYHLGHFSKFIPEGSQRVGLIASEK---NDLDTVALVRPDG--AAVVVVLNRSSKDVPLTIKDPA-VGFLETMVPGYSIHTYLWRRQ--

>manatee

ARPCIPQSFGYS-SVVCVCNATYCDSLDPLTL--PALGNFSLYESTRSGRRMELSTGTVQTSR-TGTGLLLTLQPEQK---FQKVKGFGGAMTDAAALNILALSPAVQNLLLKSYFSE-EGIEYNIIRVPMASCDFSIRTYTYADITNDFQLHNFSLPEEDTKLKIPLIHRALEMTQRPISLFASPWTSPTWLKTNEAVNGKGSLKGQPGDIYHQTWARYFVKFLDAYAEHKLRFWAVTAENEPTAGL-LSGYPFQCLGFTPEHQRDFIARDLGPILANSTHRDVHLLILDDQRLLLPGWAQVVLADPEAAKYVHGIAVHWYLDYLAPAKATLGETHRLFPTTMLFASEACVGSKFWE--QSVRLGSWDRGTQYSHSIITNLLYHVVG--------WTDWNLALNPEGGPNWVRNFV--DSPIIVDITKDTFYKQPMFYHLGHFSKFIPEGSQRVGLVASAK---NDLDTVALVRPDG--SAVVVVLNRSSKDAPLTIADPA-VGFLETMVPGYSIHTYLWRRQ--

>guineapig

AHPCIPKSFGYS-SVVCVCNATYCDSLDPVTF--PALGNFSRYESTRSGRRMELSVGAFQANR-TGTGLLLTLQPEEK---FQKVKGFGGAMTDAAALNILALSPPAQDLLLKSYFSK-EGIEYNIIRVPMASCDFSIRTYTYDDTPDDFQLHNFSLAEEDTKLKIPLIHRALQMSQRSVSLFASPWTSPTWLKTNGAVNGKGSLKGQPGDIYHQTWARYFVKFLDAYAAYNLKFWAVTVENEPTAGL-MSGYPFQCLGFTPEHQRDFIARDLGPTLANSTHRDIRLIMLDDNRLLLPHWAQVVLTDQEAAKYVHGIAVHWYLDFLAPAKATLGATHRLFPDTMLFASEACVGSKFWE--QSVRLGSWDRGMQYSTSIITNLLYHVVG--------WTDWNLALNPEGGPNWVRNFV--DSPIIVDIAKDTFYKQPMFYHLGHFSKFIPEGSQRVGLVASEE---NDLEAVALLRPDG--SAVVVVLNRSSRDVPLTIKDPA-IGFLETISPGYSIHTYLWRRQ--

>degu

AYPCIPKSFGYS-SVVCVCNATYCDSLDPVTF--PALGKFSRYESTRSGRRMEQSVGEFQANR-SGTGLLLTLQPEEK---FQKVKGFGGAMTDAAALNILALSPPAQNLLLKSYFSK-EGIEYNIIRVPMASCDFSIRTYTYDDTPQDFQLHNFSLTEEDTKLKIPLIHRALQMSQRPVSLFASPWTSPTWLKTNGAVNGKGTLKGQPGDIYHQTWAKYFVKFLDAYAEHKLKFWAVTVENEPSAGL-MSGYPFQCLGFTPEHQRDFIARDLGPTLANSTHRDVQLIMLDDNRLLLPHWAQVVLTDQEAAKYVHGIAVHWYLDFLAPAKATLGATHRLFPDTMLFASEACVGSKFWE--QSVRLGSWDRGMQYSNSIITNLLYHVVG--------WTDWNLALNPEGGPNWVRNFV--DSPIIVDIAKDTFYKQPMFYHLGHFSKFIPEGSQRVGLVASEE---NNLDAVALLNPDG--CAVVVVLNRSSRDVPLTIKDPD-MGFLETIAPGYSIHTYLWRRQ--

>gr.squirrel

ARPCIPKSFGYS-SVVCVCNATYCDSLDPLTF--PALGTFSRYESTRSGRRMELSTGTFQANH-TGTGLLLTLKPEEK---FQKVKGFGGAMTDAAALNILALSPSAQSLLLKSYFST-EGIEYNIIRVPMASCDFSISTYTYADTPDDFQLHNFSLAEEDIKLKIPLIHQALKLAHRPVSLFASPWTSPTWLKTNGAVNGKGSLKGHPGDIYHQTWAKYFVKFLDAYAAYGLKFWAVTAENEPSAGL-ISGYPFQCLGFTAEHQRDFIARDLGPALANSTHRDVQLFMLDDQRLLLPRWAQVVLADPEAAKYIHGIAVHWYLDFLAPAKSTLGATHRLFPNMTLFASEACVGSKFWE--QSVRLGSWDRGMQYSHSIITNLLYHVTG--------WTDWNFALNPEGGPNWVRNFV--DSPIIVDIAKDVFYKQPMFYHLGHFSKFIPEGSQRVGLEASEK---TELETVALMHPDG--SAVVVVLNRSSKDVPFTIKDPA-VGSLETISPGYSIHTYLWRRQ--

>chinchilla

AYPCVPKSFGYS-SVVCVCNATYCDSLDPLTL--PALGTFSRYESTRSGRRMELSVGDFQANR-TGTGLLLTLQPEEK---FQKVKGFGGAMTDAAALNILALSPPAQNLLLKSYFSK-EGIEYNIIRVPMASCDFSIRTYTYDDTPDDFQLHNFSLTEEDTKLKIPLIHRALQMSQRPVSLFASPWTSPTWLKTNGAVNGKGTLKGTPGDIYHQTWAKYFVKFLDAYAEHKLKFWAVTVENEPSAGL-MSGYPFQCLGFTPEHQRDFIARDLGPTLANSTHRDIQLIMLDDNRLLLPHWAQVVLTDQQAAKYVSGIAVHWYLDFLAPAKATLGATHRLFPDTMLFASEACVGSKFWE--QSVRLGSWDRGMQYSNSIITNLLYHVVG--------WTDWNLALNPEGGPNWVRNFV--DSPIIVDIAKDTFYKQPMFYHLGHFSKFIPEGSQRVGLVASEE---NDLEAVALLHPDG--SAVVVVLNRSSRDVRLSIKDPA-IGFLETISPGYSIHTYLWRRQ--

>molerat

ARPCIPKSFGYS-SVVCVCNATYCDSLDPLTF--PAMGTFSRYESTRSGRRMELSVGDFRSNR-TGTGLLLTLQPEEK---FQKVKGFGGAMTDAAALNILALSPSAQNLLLESYFSK-EGIEYNIIRVPMASCDFSIRTYTYDDTPGDFQLHNFSLTEEDTKLKIPLIHRALQMSQRPVSLFASPWTSPTWLKTNGAVNGKGTLKGQPGDIYHQTWAKYFVKFLDAYAEHRLKFWAVTVENEPSAGL-MSGYPFQCLGFTPEHQRDFIARDLGPTLANSTHHDVQLIMLDDNRLLLPRWAQVVLGDPEAAKYVRGVAVHWYLDFLAPAKSTLGATHRLFPDTMLFASEACVGSKFWE--QSVRLGSWDRGTQYSSSVITNLLYHVVG--------WTDWNLALNPEGGPNWVRNFV--DSPIIVDIGKDTFYKQPMFYHLGHFSKFIPEGSHRVGLAASED---SDLEAVALLHPDG--SAVVVVLNRSSRDVPLTIQDPA-LGFLETISPGHSIHTYLWRRQ--

>lessermolerat

ARPCIPKSFGYS-SVVCVCNATYCDSLDPLTL--PAPGTFSRYESTRSGRRMELSVGSIQANR-TGTGLLLTLQPEEK---FQQVKGFGGAMTDAAALNILALSPSAQNLLLKSYFSE-EGIEYNIIRVPMASCDFSIRIYTYADTPNDYQLYNFSLPEEDTKLKIPLIHQALQMAQRPVSLFASPWTSPTWLKTNGAVNGKGSLKGQPGDIYHQTWAKYFVKFLDAYAEHKLKFWAVTVENEPSAGL-ISGYPFQCLGFTAEHQRDFIARDLGPTLANSTHHNVQLLMLDDQRLLLPRWAQVVLADPEAAKYVHGIAVHWYLDFLAPAKATLGETHRLFPNTMLFASEACVGSKFWE--QSVRLGSWDRGMQYSHSIITNLLYHVVG--------WTDWNLALNPEGGPNWVRNFV--DSPIIVDIAKDTFYKQPMFYHLGHFSKFIPEGSQRVGLVASEK---NDLDTVALIRPDG--SAVVVVLNRSSKDVPLTISDPA-LGFLETISPGYSIHTYLWHRQ--

>rabbit

ARPCIAKSFGYS-SVVCVCNATYCDSLDPLTL--PALGTFSRYESTRSGRRMELSTGTFQANR-TGTGLLLTLQPEEK---FQKIKGFGGAMTDAAALNILALSPAAQDLLLKSYFSK-EGIEYNIIRVPMASCDFSIRVYTYADTPDDFLLQNFSLPEEDTKLKIPLIHRALQMAQRPVSLFASPWTSPTWLKTNGAVNGKGSIKGQPGDLYHQIWARYFVKFLDAYAEHKLQFWAVTAENEPSAGL-ISGYPFQCLGFTPEHQRDFIARDLGPALANSTHRDVRLLILDDQRLLLPYWAQVVLTDPEAAKYVHGIAVHWYLDFLAPAKATLGATHRLFPNTTLFASEACVGSKFWE--QSVRLGSWDRGTQYSHSIITNLLYHVVG--------WTDWNLALNPEGGPNWVRNFV--DSPIIVDIAKDTFYKQPMFYHLGHFSKFIPEGSQRVGLLASAK---NNLDTVALMRPDG--SAVVVVLNRSSKDVPLTIEDPA-MGFLETISPGYSIHTYLWRRQ--

>pika

ARPCIPKSFGYS-SVVCVCNATYCDSLDPLTF--PALGTFSRYESTRSGRRMELSTGTFQANR-TGAELLLTLQPEEK---FQKVKGFGGAMTDAAALNILALSPSAQDLLLKSYFSK-EGIEYNIIRVPMASCDFSIRTSTYADTPEDFQLHNFSLLEEDTKLKIPLIHRALQMAQRPVSLFASPWTSPTWLKTNGAVNGKGTLKGQPGNIYHQTWARYFVKFLDAYAENNLWFWAVTAENEPSAGM-ISGYPFQCLGFTAEHQRDFIARDLGPALANSSHRDVRLLILDDQRLLLPHWAKVVLTDPEAAKYVHGIAVHWYLDFLAPAKATLGATHRLFPNITLFASEACVGSKFWE--QSVRLGSWDRGMQYSHSIITNLLYHVVG--------WTDWNLALNLEGGPNWVRNFV--DSPIIVDITKDTFYKQPMFYHLGHFSKFIPEGSQRVGLMASDK---NDLDTVALLHPNG--SAVVVVLNRSSKDVPLTIKDPA-MGFLETISPGYSIHTYVWRRQ--

>tenrec

ACPCIPQSFGYS-SVVCVCNATHCDSLDPLKL--PAPGTFSLYETTRSGRRMELSQGTIQTSH-TGTGLLLTLQPEQK---FQKVKGFGGAMTDAASLNILALSPTVQNKLLKSYFSE-EGIEYNIIRVPMGSCDFSIRTFTYADTPDDFQLSNFSLLEEDTKLKIPLIHRALKMTQRPISLFASPWTSPTWLKTNGAVNGKGSLKGQPGDIYHQTWAKYFVKFLDAYAEHNLRFWAVTAENEPSAGL-ISGYPFQCLGFTAEHQRDFIARDLGPTLANSTHRDVQLLMLDDQRLLLPHWANVVLKDPEAAKYVHGIAVHWYLDFLAPAKSTLGETHRLFPDTMLFASEACVGSKFWE--QSVRLGSWSRGVQYSQSIITNLLYHVAG--------WMDWNLALNPEGGPNWVRNFV--DSPIIVDITKDTFYKQPMFYHLGHFSKFIPEGSQRVGLAASEK---TELEAVALIRPDG--SAVVVVLNRSSKDMPFTIEDPA-VGFLQTMAPGNSIHTYLWRRQ--

>pigeon

GRPCNAKDFGHG-SQVCACSATYCDTLDPVVL--PAPGTYVKYESSKAGKRLERSQGSFRQNA-KTPDFHLTLNTAQR---YQKVKGFGGSVTDSAAINIQSLSKDAQNHLLRSYFSE-EGIEYNLVRVPMASTDFSVRLYTYADAVGDFELKHFNLTEEDTQMKIPILQAAQAMAKRPLSLYASPWTSPVWMKTNGAMTGRGTLKGSPGDKYHQAWAKYFIRFLDEYAKHNLTFWAVTAGNEPTAGQ-IVFYPFQCLGFSPEHQRDFIARDLGPALANSSHRHVRLIILDDQRVMLPYWAQVVLKDPVAAGYISGIGIHWYLDFLAPIDLTLSITHHLFPDYFLLSTEASTGSYFWE--PRVVLGDWDRGSKYSHSILTVLNHFVTG--------WTDWNLALDLQGGPNWVKNYV--DSPVIVDSSKDVFYKQPMFYHLGHFSKFIPEGSQRVGLSVSKKCRWCNLEHSAFLRPDG--AVVLVVLNRSPADVSFGISDPR-VGSIEATAPSDSIQTFLWKQPA-

>flycatcher

GRPCDAKDFGHG-SLVCACSATYCDTLDPLVL--PAPGSYVKYESSKAGKRLERSEGSFQHNT-ESPDFHLTLDTAQR---YQKVKGFGGSITDAAAINIQSLSKDAQNHLLRSYFSE-EGIEYNLVRVPMASTDFSIRLYTYADAEGDFELRHFNLTEEDTRMKIPILQAAQAVAKRPLSLYASPWTSPVWMKTNGAMTGRGTLKGSPGDKYHRAWAKYFIRFLDEYAKHNLTFWAVTAGNEPTAGE-IVFYPFQCLGFSPEHQRDFIAQDLGPALANSSHRHVQLIILDDQRVMLPYWAEVVLKDPVAASYISGIGIHWYLDFLAPIDLTLSITHHLFPDYFLLSTEASTGSYFWE--PRVVLGGWDRGSKYSHSILTNLNNYVTG--------WTDWNLALDMEGGPNWSKNYV--DSPVIVDSSKDIFYKQPMFYHLGHFSKFIPEGSQRVGLAVSKRCRRCDLEHSAFLRPDG--AVVLVVLNRSPVDVSFGVSDPR-VGFIEAVAPSDSIQTFLWRQPA-

>sparrow

------------------CNATYCDTLDPLVL--PAHGYYVKYESSKAGKRLERSEGKFQHKL-CAPDVVLRLDTTQR---FQRVKGFGGSITDAAAINILSLPERAQDHLLRSYFSE-EGLEYNLIRLPMASCDFSLHAYTYDDVPYDYELTHFALRDEDTKLKIPLLHRASAMSKRPLSLYASPWTSPTWLKTSESYVGKGTLKGQAGDKYHKTWANYFVRFLDEYAKHNVTFWAVTAENEPTAGL-INNYPFQCLGFTAEQQRDFIARDLGPALANSSHRHVQLIILDDNRLHLPHWARVVLEDEQAARYVHGIGIHWYLDFIGPIQDTVLPTHELFPDYFILATEACIGAHFWE--RDVILGCWERGNQYSHSILTNLNHFVAG--------WTDWNLALDLEGGPNWVKNYV--DSPIIVDSSEGIFYKQPMFYHMGHFSKFIPEGSQRVGLVASRESKKTALEYTAFLRPDG--AVVVVVLNRSPQDITFGLAD-T-VGLLAAVAPANSIQTYVWQRQ--

>zebrafinch

----------------CVCNATFCDTLDPLVL--PASGYFVKYESSKAGKRLERSEGKFQRRL-CPSDVVLTLDTTQR---FQKVKGFGGSITDAAAINILSLPEGAQDHLLRSYFSE-EGLEYNLIRLPMASCDFSLHAYTYDDVPYDYELAHFSLRDEDTKLKIPLLHRASAMSKRPLSLYASPWTSPTWLKTSESYVGKGTLKGQAGDKYHKTWANYFVRFLDEYAKHNVTFWAVTAENEPTAGL-INNYPFQCLGFTAEQQRDFIARDLGPALANSSHRHVQLIILDDNRLHLPHWARVVLEDEGAARYVHGIGIHWYLDFIGPIQDTVLPTHELFPDYFILATEACIGAHFWE--RDVILGCWERGNQYSHSILTNLNHYVSGGTGRAHAQWTDWNLALDLEGGPNWVKNYV--DSPIIVDSSEGIFYKQPMFYHMGHFSKFIPEGSQRVGLVASRESKKTALEYTAFLRPDG--AVVVVVLNRSPQDITFGLAEPS-AWWQPWLRPTPS--NLLWQRQ--

>mallard

ARPCSPKYFGRD-SMVCVCNATYCDTLDPVVL--PAPGTFVKYESSKAGKRLERSEGSFQRSL-RAPDLVLTVDTTQR---YQKIKGFGGSVTDSAAINILSLPEKAQDHLLRSYFSE-EGLEYNLVRIPMASCDFSLHAYTYDDVPYDYELTHFSLRDEDTKLKIPILHRASAMAKRPLSLYASPWTSPAWMKTSESFIGKGTLKGQAGDKYHKTWANYFVRFLDEYAKHNLTFWAVTAENEPSAGL-INNYPFQCLGFTAEQQRDFIARDLGPALANSSHRNVQLIILDDNRLHLPHWAKVVLKDPVAASYISGIGIHWYLDFIGPIQDTVVPTHELFPDYFILATEACIGAHFWE--RDVILGCWERGNQYSHSILTNLNHFVAG--------WTDWNLALDLEGGPNWVKNYV--DSPVIVDSSKDVFYKQPMFYHMGHFSKFIPEGSQRVGLVASKESKKTDLEYSAFVRPDG--AVVVVVLNRSLQNVTFGLAD-T-VGLIEAVAPASSIQTYLWRRQ--

>parakeet

--------------MVCVCNATYCDTLDPVVL--PAPGTYIKYESSKAGKRLERSQGSFQRSL-RAPDLVLTVDTAQR---YQEVKGFGGSITDAAAINILSLPEAAQDHLLRSYFSE-EGLEYNLVRLPMASCDFSLHAYTYDDVPFDYELKHFSLRDEDTKLKIPLLHRALAMSKRPLSLYASPWTSPTWMKTSESYVGKGTLKGQAGDKYHKTWANYFVRFLDEYAKHNLTFWAVTAENEPSAGL-INNYPFQCLGFTAEQQRDFIARDLGPALANSSHRGIRLIILDDNRLHLPHWAKVVLEDEEAARYVHGIGIHWYLDFIGPIKDTVVPTHELFPDYFILATEACIGAHFWE--RDVILGCWERGNQYSHSILMNLNHFVSG--------WTDWNLALDLEGGPNWVKNYV--DSPVIVDSSEGIFYKQPMFYHMGHFSKFIPEGSQRVGLVASKESKKTDLEYTAFLRPDG--AMVVVVLNRSPQSITFGLAD-S-VGLIAAEAPASSIQTYLWQRQ--

>groundtit

ARPCIPKDFGHG-SLVCVCNATYCDTLDPLVL--PTPGSYVKYESSKAGKRLERSEGRFQSSL-RTPGLLLTLNISTL---YQHVKGFGGSLSDAAAMNILKLSQPAQDNLLRSYFSE-SGIEYNLIRVPMACSDFSVRPYSYDDVPDDYELKHFRLADEDVKMKIPLLRRTLAMSKRPLLLFASPWTAPAWMRSNGDVRGKGTLKGKAGDKYHKSWANYFIKFLDEYAKHNVTFWAVTAQNEPLAGL-LTPPQAPTIAFTAAQQRDFIAQDLGPALAHSPHR-TRLLMLDDQRIHLPHWAKVVLGNATAARYVAGLAVHWYLDAIVPPGCSLEATHKLFPDHFLLYTEACTGFFTFR--FAVSLGCWERGDHYSYSILTVMNHFVSG--------WTDWNLVLDLEGGPNWVKNFV--DSPVIVDGSKDVFYKQPMFYHMGHFSKFIPEGSRRVGLHSSRRCLLCQLEHVAVLRPDG--ALVLVVLNRFGRDVPFGIRDPA-MGFIETVAPAHSIQTYLWRQQ--

>falcon

-RPCDAKDFGHG-SMVCVCNATYCDTLDPVVL--PAPGTYVKYESSKAGKRLERSEGSFQHDP-ETPGLLLTLNVSSL---YQHVKGFGGSLSDAAALNILALSPPAQDNLLRSYFSK-SGIEYNLVRLPMACSDFSVRPYSYDDVPHDYELKHFRLAEEDVKMKIPLLHRASAMSRRPLSLYASPWTSPAWMKSNGDVRGKGTLKGQAGDKYHKTWANYFVKFLDEYAKHNVTFWAVTAQNEPLAAL-LTPPQFPTIAFTALQQRDFVIHDLGPALARSPHP-TRLIILDDQRIHLPHWAKVILGNATGARYVAGVGVHWYLDSIVPASCSLDATHKLFPDHFLLYTEACSGFLTLR--SSVSLGCWERGDHYSHSILTVLNHFVAG--------WTDWNLALDLEGGPNWVKNYNYVDSPIIVDSSKDVFYKQPMFYHLGHFSKFIPEGSRRVGLHSSRRCLLCQLEHVAALRPDG--TLVLVVLNRFGLDVPFGIRDPA-VGFINTVAPANSIQTYLWRQH--

>hummingbird

-HPCDAKDFGHG-SLVCACSATYCDTLDPVVL--PAPGTYIKYESSKAGKRLERSEGTFQPNA-GSTDFHLTLDTTQR---YQKVKGFGGSVTDSAAINIQSLSQDAQNNLLRSYFSE-EGIEYNLVRVPMASTDFSVRLYTYADAVGDFELKHFNLTEEDTQMKIPILQAAQAMAKRPLSLYASPWTSPVWMKTNGAMTGRGTLKGSPGDKYHQAWAKYFIRFLDEYAKYNLTFWAVTAGNEPTAGE-IVFYPFQCLGFSPEHQRDFIARDLGPALANSSHHNVQLIILDDQRVMLPYWAQVVLKDPVASSYISGIGIHWYLDFLAPIDLTLSITHHLFPDYFLLSTEASTGSYFWE--PRVVLGGWDRGSKYSHSILTNLNNYVTG--------WTDWNLALNMEGGPNWSKNYV--DSPIIVDSSKDIFYKQPMFYHMGHFSKFIPEGSQRVGLSVSKKCHRCDLEHSAFLRPDG--TVVLVVLNRSTTDVSFGISDPR-VGFIEDTAPGDSIQTFLWQQPA-

>manakin

GRPCDAKDFGHG-SLVCACSATYCDTLDPLVL--PPPGSYVKYESSKAGKRLERSEGRFQHNA-ESPDFHLTLDTAQR---YQKVKGFGGSITDAAAINIQSLSQAAQNHLLRSYFSE-EGIEYNLVRVPMASTDFSVRLYTYADAEGDFELRHFNLTEEDTRMKIPILRAAQAVAKRPLSLYASPWTSPVWMKTNGAMTGRGTLKGNPGDKYHKAWAKYFIRFLDEYAKHNLTFWAVTAGNEPTAGE-IVFYPFQCLGFSPEHQRDFIAQDLGPALANSSHRHVQLIILDDQRVMLPYWAQVVLKDPVAASYISGIGIHWYLDFLAPIDLTLSITHHLFPDYFLLSTEASTGSYFWE--PRVVLGGWDRGSKYSHSILTNLNNYVTG--------WTDWNLALDMEGGPNWSKNYV--DSPIIVDSSKDIFYKQPMFYHLGHFSKFIPEGSQRVGLAVSKKCHRCDLEHSAFLRPDG--TVVLVVLNRSPTDVSFGISDPR-IGFIEATAPSDSIQTFLWKQPA-

>emp.penguin

---------------VCVCNATYCDTLDPVVL--PALGTYVKYESSKAGKRLERSEGSFQRSL-RAPGLLLTLNVSTM---YQHVKGFGGSLSDAAALNILGLSRPAQDNLLRSYFSE-SGIEYNLIRLPMACSDFSVRPYSYDDVPHDYELKHFRLAEEDLKMKIPLLHRASAMSRWPLSLYASPWTSPAWMKSNGDVRGKGTLKGQAGDKYHKTWANYFIKFLDEYAKHNVTFWAVTAQNEPLAAL-LTHPQFPTIAFTAAQQRDFIVRDLGPALAR-SPHHTQLIILDDQRINLPHWAKVVLGNATAAHYVAGMGVHWYLDGIVPASCSLEATHKLFPDHFLLYTEACSGFITLR--FSVSLGCWERGNRYSHSILTVLNHFVAG--------WTDWNLALDLEGGPNWVKNYV--DSPVIVDSSKDVFYKQPMFYHLGHFSKFIPEGSRRVGLHSSRRCLICQLEHVAILRPDG--ALVLVVLNR-------------------------------------

>cuckoo

---------------VCVCNATYCDMLDPVVV--PPLGTYVKYESSKAGNRLERSEGSFQRSL-HAPDLVLTVDTTQR---FQKLKGFGGSLTDAAAINILSLPEMAQEHLLRSYFSE-EGLEYNLVRLPMASCDFSLHTYTYDDVPYDYELTHFSLQDEDTKLKIPLMHRALAMAKRPLSLCASPWTSPTWMKTSESFVGKGTLKGQAGDKYHKTWANYFVRFLDEYAKHNLTFWAVTAENEPSAGL-INNYPFQCLGFTAEQQRDFIARDLGPALANSSYRDVQLIILDDNRLHLPHWAKVVLEDEEAARYVHGIGIHWYLDFIGPIQDTVVPTHELFPDYFILATEACIGSHFWE--RDVVLGCWERGNQYSHSILMNLNHFVAG--------WIDWNLALDLEGGPSWVKNYV--DSPIIVDSSEGVFYKQPMFYHLGHFSKFIPEGSQRVGLLASKESKKTELEYTALLRPDG--TVVVVVLNR-------------------------------------

>woodpecker

---------------VCVCNATYCDTLDPVVL--PARGTYLKYESSKAGKRLERSEGRFERSL-QAPDLVLTVDTTQR---YQKVKGFGGSVTDAAAINLLSLPEAAQDHLLRSYFSE-EGLEYNLVRLPMASCDFSLHAYTYDDVPYDYELKHFRLRDEDTKLKASGQEAALAMSKQPVSLIASPWTSPAWMKTSESYVGKGTLKGQAGDKYHKTWANYFVRFLDEYSKHNLTFWAVTAENEPTAGL-INNYPFQCLGFTAEQQRDFIARDLGPALANSSHPHVQLIILDDNRLHLPHWAKVVLEDEQAARYVHGIGIHWYLDFIGPIQDTVVPTHELFPDYFILATEASIGAHFWE--RDVILGCWERGNQYSHSILMNLNNYVTG--------WIDWNLALDLEGGPNWVKNYV--DSPVIVDRSEGIFYKQPMFYHMGHFSKFIPEGSQRVGLVASKESKKTELEYTAFLRPDG--ALVVVVLNR-------------------------------------

>alligators

SHPCNARNFGHG-LLVCECSATYCDTLDPVTL--PPAGTFLMYESNKAGKRLERSKGGVQRSL-TDPGFRLTLNTTER---YQKVKGFGGSITDSAAINILSLSQEAQNKLLRSYFSE-EGIEYNLIRVPMASCDFSVRLYTYADMRGDFALKHFSLTEEDTKMKIPILQHVQAMSRKPVSLYASPWTSPTWMKTNGAMTGRGTLKGKPGDKYHKTWANYFIKFLDEYAKHNLTFWAVTSGNEPTAGE-IVFYPFQCLGFSPEHQRDFIARDLGPALANSSHKGIQLIMLDDQRMMLPYWAEVVLKDPVAASYINGIGIHWYLDFLAPIDLTLSMTHHLFPNYYLLSTEASTGSYFWE--PRVILGGWDRGSKYSHSILTNLNNYVTG--------WTDWNLALDLEGGPNWSKNYV--DSPIIVDSSKDLFYKQPMFYHMGHFSKFLPEGSQRVGLAAEQKCYQCELEYSAFLRPDG--AAVVVVLNRTPKDISFVISDPG-IGFIDAKAPADSVQTYLWMRQKG

>alligatorm

SHPCNARNFGHG-SLVCECSATYCDTLDPVTL--PPAGTFLMYESNKAGKRLERSKGGVQRSL-TAPGFRLTLNTTER---YQKVKGFGGSITDSAAINILSLSQEAQNKLLRSYFSE-EGIEYNLIRVPMASCDFSVRLYTYADMRGDFALKHFSLTEEDTKMKIPILQHAQAMSRKPVSLYASPWTSPTWMKTNGAMTGRGTLKGKPGDKYHKTWANYFIKFLDEYAKHNLTFWAVTSGNEPTAGE-IVFYPFQCLGFSPEHQRDFIARDLGPALANSSHKGIQLIMLDDQRVMLPYWAEVVLKDPVAASYINGIGIHWYLDFLAPIDLTLSMTHHLFPNYYLLSTEASTGSYFWE--PRVILGGWDRGSKYSHSILTNLNNYVTG--------WTDWNLALDLEGGPNWSKNYV--DSPIIVDSSKDLFYKQPMFYHMGHFSKFLPEGSQRVGLAAEQKCYQCELEYSAFLRPDG--AAVVVVLNRTPKDISFVISDPG-IGFIDAEAPADSVQTYLWMRQKG

>anole

SRPCAPRFLSAR-AMVCVCNATYCDTMDPVSI--PAVGRFVKYESTSAGGRLERSEGVIQPST-SGSGLRYTYNPFVH---YQRIKGFGGSLTDAAAINIMRLSPAAQDHLLRSYFSE-EAIEYNVLRLPMASCDFSVRPYSYAISARRLRLAVLQPGLRDTDLRIPLLHRFVAAAKRHLSLIGSPWSAPGWLRTNNQVMGKARLKGEPGDIHHKTWARYFVRFLDEYACHNVTFWAVTAQNEPSSSVFIPMKNFPMTQFSPEKQRDFIIADLGPALAASRHKDVLLIIHDDQRIHLPNWANVVIGNSSAARYVAGIGIHWYLDSVTPPELTLEATYLLFPDFFLLYTESCNGFRVWE--PRVDLGSWERGVRYSQNILTNLNSFIVG--------WIDWNLVLDMEGGPNYVKNHV--DSPIIVNPLRDEFYKQPMFYHLAHFSKFIPEGSIRVAMTKNFEITGCQLQSTGFVRPDG--IAVVVVLNKNPEDVPFCISDPD-VGHIEAVATANSIQTYLWQRPSG

>python

GRPCSPRNFGHG-SLVCECGANYCDTLEPVSL--PALGSFVKYESNKAGHRLERSEGPLLDNP-PKKDDLVLQLKVFQK--YQKIKGFGGALTDSAAINILALSAKSQKNLLKSYFST-EGIEYTLVRVPMASCDFSVRLYTYADVEDDFELNNFSLTEEDIKMKIPILQQAQAVAPRPLSLYASPWTSPVWMKTNGAMTGRGTLKGKPGDRYHKTWANYFIRFLDEYAKYNLTFWAVTAGNEPTAGD-LVFYPFQCLGFSPEHQRDFIAQDLGPALANSTYKGIELIILDDQRVMLPYWAEVVLKDPEAAQYINGIGIHWYLDFLAPIDLTLSITHHLFPKYYLISTEASTGSYFWE--PRVVLGGWDRGSKYSHSILTNLNNYVTG--------WTDWNLVLDMQGGPNWSKNYV--DSPVIVDKDKDVFYKQPMFYHMAHFSKFLPEGTQRIEIQKNSW---SNLEFSAFLRPDG--SAAVVVLNRASKDIPFGISDPG-VGYVEAVATADSIQTYLWQRPTE

>turtle

GRPCNAKNFGHD-SLVCECNASYCDTLDPVVV--PALGTYAKYESSKAGKRLERSEGRFQSDS-TAPDLLLKLDTAQQ---YQKVKGFGGALTDSAAMNIMALSPGAQTNLIKSYFSE-EGIEYNLVRIPMASCDFSVRLYTYDDSEGDFELRNFSLTEEDTKLKIPILQHAQAVARTPLSLYASPWTSPIWLKTNGAMTGRGTLKGKPGDKYHKTWANYFIRFLDEYAKHNLTFWAVTAGNEPTAGE-IVFYPFQCLGFSPEHQRDFIAQDLGPALANSSHKGIQLIMLDDQRVMLPYWAEVILNDPVAASFINGIGIHWYLDFLAPIDLTLSITHNLFPGYYLLSTEASTGSYFWE--PRVVLGGWDRGSKYSHSILTNLNNYVTG--------WTDWNLALDLEGGPNWSKNYV--DSPVIVDSSKDLFYKQPMFYHMGHFSKFVPEGSQRVRLVISKKCYKCNLEYAAFLRPDG--AAVLVVLNRYPTDVPFGISDPG-VGFIEAVAPADSIQTYLWRR---

>gr.seaturtle

SRPCSPQYFGHD-LMVCECNATYCDMLDPVVV--PAVGTYAKYESSKAGKRLEHSEGRFQSDS-MAPDLVLKLDTTQQ---YQKVKGFGGSVTDSAAMNILSLSKETQRHLLASYFTE-EGIEYNLLRIPMASCDFSTHPYSYDDTPYDYQLLNFGLKDEDTKLKIPILHRAMALSKKPLSLVASPWSSPVWMKTSGEMKGKGSLKGKPGDKYHKTWANYFIRFLDEYTKHNLTFWAVTAQNEPTAGL-INNYPFQCLGFTAEHQRDFIAQDLGPALANSSHKGIRLIMLDDNRVLLPHWAKVVLGDPNAARYVHGIGVHWYLDFIAPIEDTVLPTHDLFPDYFILATEACTGSHFWE--RDVILGCWDRGNQYSHSILTNLNNFVSG--------WIDWNLALDLEGGPNWVQNLV--DSPVIVDRKRDLFYKQPMFYHIGHFSKFIPEGSQHVGLVVSKKSCKCNIEYAAFLRPDG--AAVLVVLNRYSTDVPFGISDPG-VGFIEALVPADSIQTYLWRRQEF

>xenopus

GRLCAPLNFGQS-SVVCQCNATYCDTLDPIVV--PSVGNFSVYETSQSGKRLQVTSGTFTKRQPSPMDLVLTLNDKKK---FQTIKGFGGAVTDSAALNILSLSDETKENLLRSYFSE-EGIGYNILRVPMGSCDFSTRIYTYLDTEGDFSMKTFSLQVEDTKLKIPLIQKAKELSNRSISLFASPWTSPPWMKTNGAITGKGTLKGKPGDQYHKTWANYFIRFLDEYAKLNVTFWAVTVENEPTAGL-VTDYPFQSLGFTPEHMRDFIASDLGPAFANSSHKQVKIMILDDNRLLLPYWAKVILSDLKAARYVHGIAVHWYLDAIVPADVTLGRTHQLYPDYFLFASEACTGFTPWN--KGVQLGCWDRGNQYSHRIIEDLNYYVTG--------WTDWNLALDIEGGPTWVENNV--DSPIIVDLSKDVFYKQPMFYHMAHFSKFIPEGSRRVGLDLNQG---SQLETVAFLSPDGS-VAVVVVLNRESVDVKFLISDPS-LGVIDTVSPANSIQTYIWRRQ--

>axolotl

GNMCAPVSFGHN-SVVCMCNSTYCDTVGPLVI--PTMGNYTKYESSLAGKRLENTKGSLQENR-TTPGLVLTLNLKKR---YQIIKGFGGAVTDASSINILSLSPQTQKNLLKAYFSE-EGIEYNVLRVPMASCDFSMRVYTYDDTADDFELKNFSLQEEDLKLKIPVIQQALAISKKDIALIASPWSSPVWLKTNNAMIGKGTLKGMPGDKYHKTWANYFIRFLDEYAKHNLTFWAVTAENEPTAGT-LTGYSFQALGFTPEHQRDFIASDLGPAFATSAHGKVKLLILDDQRVLLPYWAKVVLSDLRAAKYIHGIAVHWYLDAIVPADLTLGATHQLFPEYFLFATEACNGFLFWD--KGVRLGCWDRGNKYSHSIIEDLTHYVTG--------WTDWNLALNAKGGPNWVNNFA--DSPVIVDPAKDVFYKQPMFYHIAHFSKFIPEGSQRVGLDASEA---SVLETVAVLRPDG--AAVVIVLNRTPADVPFVIFDPH-VGNINTISPANSIQTYLWQQH--

>coelacanth

EKPCAPLNFGQS-SVVCVCSATYCDTLDPLKI--PSVGSYIAYETNKAGKRVEGMKGAIQKNI-TDPGLKFTLNTAKR---YQRIKGFGGTVTDAAADNILSLTPATQQNLLRSYFSE-EGIEYNVIRVPMASCDFSARVYTYDDSPGDFDLKNFSLAMEDLELKIPVIQQALKLSKRPIHLFASPWTAPSWLKTNNAAIGKGTLKGEPGDKYHKTWANYFVRFLDEYAKFNLTFWAVTTENEPTAGF-FTNYSFQCLGFTPEHMRDFIVSDLGPALKGSNHKDVQLMLLDDQRLLLPYWAKVVLNDANVAQYIHGIAVHWYFDSFVPARATLGATHELFPDYYLFATEACTGTFPWD--RGVQLGSWERGCAYSHDIIEDLNYYVTG--------WTDWNIALNLKGGPNWVENFV--DSPVIVDPSKDVFYKQPMFYHMAHFSKFIPEDSQRVGLDASKD---TELETVTFLCPDG--AAVLVVLNQSPVDVKFAIWDPE-VGYIDSISPADSIKTFLWNR---

Beta-synuclein

>human Homo sapiens

MDVFMKGLSMAKEGVVAAAEKTKQGVTEAAEKTKEGVLYVGSKTREGVVQGVASVAEKTKEQASHLGGAVFSGAGNIAAATGLVKREEFPTDLK-PEEVAQEAAEEPLIEPLMEPEGESYEDPPQEEYQEYEPEA

>chimpanzee Pan troglodytes

MDVFMKGLSMAKEGVVAAAEKTKQGVTEAAEKTKEGVLYVGSKTREGVVQGVASVAEKTKEQASHLGGAVFSGAGNIAAATGLVKREEFPTDLK-PEEVAQEAAEEPLIEPLMEPEGESYEDPPQEEYQEYEPEA

>orangutan Pongo abelii

MDVFMKGLSMAKEGVVAAAEKTKQGVTEAAEKTKEGVLYVGSKTREGVVQGVASVAEKTKEQASHLGGAVFSGAGNIAAATGLVKREEFPTDLK-PEEVAQEAAEEPLIEPLMEPEGESYEDPPQEEYQEYEPEA

>gibbon

MDVFMKGLSMAKEGVVAAAEKTKQGVTEAAEKTKEGVLYVGSKTREGVVQGVASVAEKTKEQASHLGGAVFSGAGNIAAATGLVKREEFPTDLK-PEEVAQEAAEEPLIEPLMEPEGESYEDPPQEEYQEYEPEA

>baboon Papio anubis

MDVFMKGLSMAKEGVVAAAEKTKQGVTEAAEKTKEGVLYVGSKTREGVVQGVASVAEKTKEQASHLGGAVFSGAGNIAAATGLVKREEFPTDLK-PEEVAQEAAEEPLIEPLMEPEGESYEDPPQEEYQEYEPEA

>rhesus Macaca mulatta

MDVFMKGLSMAKEGVVAAAEKTKQGVTEAAEKTKEGVLYVGSKTREGVVQGVASVAEKTKEQASHLGGAVFSGAGNIAAATGLVKREEFPTDLK-PEEVAQEAAEEPLIEPLMEPEGESYEDPPQEEYQEYEPEA

>gr.monkey Chlorocebus sabaeus

MDVFMKGLSMAKEGVVAAAEKTKQGVTEAAEKTKEGVLYVGSKTREGVVQGVASVAEKTKEQASHLGGAVFSGAGNIAAATGLVKREEFPTDLK-PEEVAQEAAEEPLIEPLMEPEGESYEDPPQEEYQEYEPEA

>sq.monkey Saimiri boliviensis

MDVFMKGLSMAKEGVVAAAEKTKQGVTEAAEKTKEGVLYVGSKTREGVVQGVASVAEKTKEQASHLGGAVFSGAGNIAAATGLVKREEFPTDLK-PEEVAQEAAEEPLIEPLMEPEGESYEDPSQEEYQEYEPEA

>marmoset Callithrix jacchus

MDVFMKGLSMAKEGVVAAAEKTKQGVTEAAEKTKEGVLYVGSKTREGVVQGVASVAEKTKEQASHLGGAVFSGAGNIAAATGLVKREEFPTDLK-PEEVAQEAAEEPLIEPLMEPEGESYEDPPQEEYQEYEPEA

>galago Otolemur garnettii

MDVFMKGLSMAKEGVVAAAEKTKQGVTEAAEKTKEGVLYVGSKTREGVVQGVASVAEKTKEQASHLGGAVFSGAGNIAAATGLVKKEEFPTTMK-PEEVAQEAAEEPLIEPLMEPEGESYEETPQEEYQEYEPEA

>treeshrew Tupaia chinensis

MDVFMKGLSMAKEGVVAGAEKTKQGVTEAAEKTKEGVLYVGSKTREGVVQGVASVAEKTKEQASHLGGAVFSGAGNIAAATGLVKKEEFPTDLK-PEEVAQEAAEEPLIEPLVEPEGESYEEPPQEEYQEYEPEA

>mouse Mus musculus

MDVFMKGLSMAKEGVVAAAEKTKQGVTEAAEKTKEGVLYVGSKTS-GVVQGVASVAEKTKEQASHLGGAVFSGAGNIAAATGLVKKEEFPTDLK-PEEVAQEAAEEPLIEPLMEPEGESYEDSPQEEYQEYEPEA

>rat Rattus norvegicus

MDVFMKGLSMAKEGVVAAAEKTKQGVTEAAEKTKEGVLYVGSKTKEGVVQGVASVAEKTKEQASHLGGAVFSGAGNIAAATGLVKKEEFPTDLK-AKEVAQEAAEEPLIEPLMEPEGESYEDSPQEEYQEYEPEA

>g.hamster Mesocricetus auratus

MDVFMKGLSMAKEGVVAAAEKTKQGVTEAAEKTKEGVLYVGSKTKEGVVQGVASVAEKTKEQASHLGGAVFSGAGNIAAATGLVKKEEFPTDLK-PEEVAQEAAEEPLIEPLMAPEGESYEETPQEEYQEYEPEA

>c.hamster Cricetulus griseus

MDVFMKGLSMAKEGVVAAAEKTKQGVTEAAEKTKEGVLYVGSKTKEGVVQGVASVAEKTKEQASHLGGAVFSGAGNIAAATGLVKKEEFPTDLK-PEEVAQEAAEEPLIEPLMEPEGESYEETPQEEYQEYEPEA

>jerboa jaculus jaculus

MDVFMKGLSMAKEGVVAAAEKTKQGVTEAAEKTKEGVLYVGNKTREGVVQGVASVAEKTKEQASHLGGAVFSGAGNIAAATGLVKKEEFPTDLK-PEEVAQEAGEEPLIEPLMEPEGESYEETPQEDYQEYEPEA

>guineapig Cavia porcellus

MDVFMKGLSMAKEGVVAAAEKTKQGVTEAAEKTKEGVLYVGSKTREGVVQGVASVAEKTKEQASHLGGAVFSGAGNIAAATGLVKKEEFPTDLK-PEEVAQEAAEEPLIEPLMEPEGESYEESPQEEYQEYEPEA

>degu Octodon degus

MDVFMKGLSMAKEGVVAAAEKTKQGVTEAAEKTKEGVLYVGSKTREGVVQGVASVAEKTKEQASHLGGAVFSGAGNIAAATGLVKKEEFPTDLK-PEEVAQEAAEEPLIEPLMEPEGESYEEPPQEEYQEYEPEA

>gr.squirrel Ictidomys tridecemlineatus

MDVFMKGLSMAKEGVVAAAEKTKQGVTEAAEKTKEGVLYVGSKTREGVVQGVASVAEKTKEQASHLGGAVFSGAGNIAAATGLVKKEEFPTDLK-PEEVAQEAAEEPLIEPLMEPEGENYEEPPQEEYQEYEPEA

>chinchilla Chinchilla lanigera

MDVFMKGLSMAKEGVVAAAEKTKQGVTEAAEKTKEGVLYVGSKTREGVVQGVASVAEKTKEQASHLGGAVFSGAGNIAAATGLVKKEEFPTDLK-PEEVAQEAAEEPLIEPLMEPEGESYEEPPQEEYQEYEPEA

>molerat Heterocephalus glaber

MDVFMKGLSMAKEGVVAAAEKTKQGVTEAAEKTKEGVLYVGSKTREGVVQGVASVAEKTKEQASHLGGAVFSGAGNIAAATGLVKKEEFPSDLK-PEEVAQEAAEEPLIEPLMEPEGESYEEPPQEEYQEYEPEA

>rabbit Oryctolagus cuniculus

MDVFMKGLSMAKEGVVAAAEKTKQGVTEAAEKTKEGVLYVGSKTREGVVQGVASVAEKTKEQASHLGGAVFSGAGNIAAATGLVKREEFPTDLK-PEEVAQEAAEEPLIEPLMEPEGESYEEPPQEDYQEYEPEA

>pika Ochotona princeps

MDVFMKGLSMAKEGVVAAAEKTKQGVTEAAEKTKEGVLYVGSKTREGVVQGVASVAEKTKEQASHLGGAVFSGAGNIAAATGLVKKEEFPTDLK-PEEVAQEAAEEPLIEPLMEPEGESYEEPPQEEYQEYEPEA

>panda Ailuropoda melanoleuca

MDVFMKGLSMAKEGVVAAAEKTKQGVTEAAEKTKEGVLYVGSKTREGVVQGVASVAEKTKEQASHLGGAVFSGAGNIVAATGLVKKEEFPTDLK-PEEVAQEAAEEPLIEPLMEPEGESYEEPPQEEYQEYEPEA

>ferret Mustela putorius furo

MDVFMKGLSMAKEGVVAAAEKTKQGVTEAAEKTKEGVLYVGSKTREGVVQGVASVAEKTKEQASHLGGAVFSGAGNIAAATGLVKKEEFPADLKQPEEVAQEAAEEPLIEPLMEPEGESYEEPPQEEYQEYEPEA

>cat Felis cattus

MDVFMKGLSMAKEGVVAAAEKTKQGVTEAAEKTKEGVLYVGSKTREGVVQGVASVAEKTKEQASHLGGAVFSGAGNIAAATGLVKKEEFPTDLK-PEEVAQEAAEEPLIEPLMEPEGESYEEPPQEEYQEYEPEA

>dog Canis lupus familiaris

MDVFMKGLSMAKEGVVAAAEKTKQGVTEAAEKTKEGVLYVGSKTREGVVQGVASVAEKTKEQASHLGGAVFSGAGNIAAATGLVKKEEFPADLK-PEEVAQEAAEEPLIEPLMEPEGESYEEPPQEEYQEYEPEA

>orca Orcinus orca

MDVFMKGLSMAKEGVVAAAEKTKQGVTEAAEKTKEGVLYVGSKTREGVVQGVASVAEKTKEQASHLGGAVFSGAGNIAAATGLVKKEEFPTDLK-PEEVAQEAAEEPLIEPLMEPEGESYEEPPQEEYREYEPEA

>baiji Lipotes vexillifer

MDVFMKGLSMAKEGVVAAAEKTKQGVTEAAEKTKEGVLYVGSKTREGVVQGVASVAEKTKEQASHLGGAVFSGAGNIAAATGLVKKEESPTGMK-PEEVAQEAAEEPLIEPLVEPEGESYEEPPQEEYREYEPEA

>minkewhale Balaenoptera acutorostrata scammoni

MDVFMKGLSMAKEGVVAAAEKTKQGVTEAAEKTKEGVLYVGSKTREGVVQGVASVAEKTKEQASHLGGAVFSGAGNIAAATGLVKKEEFPTDLK-PEEVAQEAAEEPLIEPLMEPEGESYEEPPQEEYREYEPEA

>pig Sus scrofa

MDVFMKGLSMAKEGVVAAAEKTKQGVTEAAEKTKEGVLYVGSKTREGVVQGVASVAEKTKEQASHLGGAVFSGAGNIAAATGLVKKEEFPADLK-PEEVAQEAAEEPLIEPLMEP-GESYEEPPQEEYQEYEPEA

>cow Bos taurus

MDVFMKGLSMAKEGVVAAAEKTKQGVTEAAEKTKEGVLYVGSKTREGVVQGVASVAEKTKEQASHLGGAVFSGAGNIAAATGLVKKEEFPTDLK-PEEVAQEAAEEPLIEPLMEPEGESYEEQPQEEYQEYEPEA

>yak Bos mutus

MDVFMKGLSMAKEGVVAAAEKTKQGVTEAAEKTKEGVLYVGSKTREGVVQGVASVAEKTKEQASHLGGAVFSGAGNIAAATGLVKKEEFPTDLK-PEEVAQEAAEEPLIEPLMEPEGESYEEQPQEEYQEYEPEA

>waterbuffalo Bubalus bubalis

MDVFMKGLSMAKEGVVAAAEKTKQGVTEAAEKTKEGVLYVGSKTREGVVQGVASVAEKTKEQASHLGGAVFSGAGNIAAATGLVKKEEFPTDLK-PEEVAQEAAEEPLIEPLMEPEGESYEEQPQEEYQEYEPEA

>sheep Ovis aries

MDVFMKGLSMAKEGVVAAAEKTKQGVTEAAEKTKEGVLYVGSKTREGVVQGVASVAEKTKEQASHLGGAVFSGAGNIAAATGLVKKEEFPTDLK-PEEVAQEAAEEPLIEPLMEPEGESYEEQPQEEYQEYEPEA

>camel Camelus bactrianus

MDVFMKGLSMAKEGVVAAAEKTKQGVTEAAEKTKEGVLYVGSKTREGVVQGVASVAEKTKEQASHLGGAVFSGAGNIAAATGLVKKEEFPTDLK-PEEVAQEAAEEPLIEPLMEPEGESYEEAPQEEYQEYEPEA

>alpaca Vicugna pacos

MDVFMKGLSMAKEGVVAAAEKTKQGVTEAAEKTKEGVLYVGSKTREGVVQGVASVAEKTKEQASHLGGAVFSGAGNIAAATGLVKKEEFPTDLK-PEEVAQEASEEPLIEPLMEPEGESYEEAPQEEYQEYEPEA

>horse Equus przewalskii

MDVFMKGLSMAKEGVVAAAEKTKQGVTEAAEKTKEGVLYVGSKTREGVVQGVASVAEKTKEQASHLGGAVFSGAGNIAAATGLVKREEFPTDLK-PEEVAQEAAEEPLIEPLMEPEGESYEEPPQEEYQEYEPEA

>rhino Ceratotherium simum

MDVFMKGLSMAKEGVVAAAEKTKQGVTEAAEKTKEGVLYVGSKTREGVVQGVASVAEKTKEQASHLGGAVFSGAGNIAAATGLVKKEEFPTDLK-PEEVAQEAAEEPLTEPLMEPEGESYEEPPQEEYQEYEPEA

>bat Myotis brandtii

MDVFMKGLSMAKEGVVAAAEKTKQGVTEAAEKTKEGVLYVGNKTREGVVQGVTSVAEKTKEQASHLGGAMFSGAGNIAAATGLMKKEEFPTDLK-PEEVAQEAAEEPLIEPLMEPEGVSYEESPQEEYQEYEPEA

>bigbrownbat Eptesicus fuscus

MDVFMKGLSMAKEGVVAAAEKTKQGVTEAAEKTKEGVLYVGNKTREGVVQGVTSVAEKTKEQASHLGGAMFSGAGNIAAATGLMKKEEFPTDLK-PEEVAQEAAEEPLIEPLMEPEGESYEDSPQEEYQEYEPEA

>flyingfox Pteropus alecto

MDVFMKGLSMAKEGVVAAAEKTKQGVTEAAEKTKEGVLYVGSKTREGVVQGVASVAEKTKEQASHLGGAVFSGAGNIAAATGLVKKEEFPTDLK-PEEVAQEAAEEPLIEPLMEPEGESYEEPPQEEYQEYEPEA

>mole Condylura cristata

MDVFMKGLSMAKEGVVAAAEKTKQGVTEAAEKTKEGVLYVGSKTREGVVQGVASVAEKTKEQASHLGGAVFSGAGNIAAATGLVKKEEFPTDLK-PEEVAQEAAEEPLIEPMMEPEGESYEEPPQEEYQEYEPEA

>manatee Trichechus manatus latirostris

MDMFMKGLSMAKEGVVAAAEKTKQGVTEAAEKTKEGVLYVGSKTREGVVQGVASVAEKTKEQASHLGGAVFSGAGNIAAATGLVKKEEFPTDLK-PEEVAQEAAEEPLIEPLMEPEGESYKELPQEEYQEYEPEA

>tenrec Echinops telfairi

MDMFMKGLSMAKEGVVAAAEKTKQGVTEAAEKTKEGVLYVGSKTREGVVQGVASVAEKTKEQASHLGGAVFSGAGNIAAATGLVKKEEFPTDLK-PEEVAQEAGEEQLIEPLMEPEGEGYEEQPQEEYQEYEPEA

>elephantshrew Elephantulus edwardii

MDMFMKGLSMAKEGVVAAAEKTKQGVTEAAEKTKEGVLYVGSKTREGVVQGVASVAEKTKEQASHLGGAVFSGAGNIAAATGLVKKEEFPTDLK-PEEVAQEAAEEPLIEPLMEPEGESYEEPPQEEYQEYEPEA

>armadillo Dasypus novemcinctus

MDMFMKGLSMAKEGVVAAAEKTKQGVTEAAEKTKEGVLYVGSKTREGVVQGVASVAEKTKEQASHLGGAVFSGAGNIAAATGLVKKEEFPTDLK-PEEVAQEAAEEPLIEPLMEPEGESYEEPLQEEYQEYEPEA

>zebrafinch Taeniopygia guttata

MEVFMKGLSKAKEGVVAAAEKTKQGVAEAAEKTKEGVLYVGSKTQ-GVVQGVTSVAEKAKEQASQLGEAAFSGAGNIAAATGLVKKEEFPADLK-AEEVAQEAVEEPLVEPLLEPEGENYEEPPQEEYQEYEPEA

>groundtit Pseudopodoces humilis

MEVFMKGLSKAKEGVVAAAEKTKQGVAEAAEKTKEGVLYVGSKTQ-GVVQGVTSVAEKAKEQASQLGEAAFSGAGNIAAATGLVKKEEFPADLK-AEEVAQEAVEEPLVEPLLEPEGENYEEPPQEEYQEYEPEA

>alligator Alligator mississippiensis

MDVFMKGLSKAKEGVVAAAEKTKQGVAEAAEKTKEGVLYMGSKTQ-GVVQGVTSVAEKTKEQASQLGGAVFSGAGNIAAATGLVKKEEFPTDLK-PEEVAQEAVEEPLIEPLLEPEGESYEEPPQEDYQEYEPEA

>anole Anolis carolinensis

MDVFMKGLSKAKEGVAAAAEKTKEGVAAAAEKTKEGVLYVGSKTREGV-AGVASVAEKTKEQASQLGGAVFSGAGNIAAATGLVKKDEFPTDLK-PEEVGQEAVEEPLIEPLLEPEGENYEEPPQEEYQEYEPEA

>burmesepython Python bivittatus

MDVFMKGLSKAKEGVVAAAEKTKEGVAVAAEKTKEGVLYVGSKTREGVVQGVASVAEKTKEQASQLGGAVFSGAGNIAAATGLVKKEEFPTDSK-PEEVGQEAVEEPLIEPLLEPESENYEESPQEEYQEYEPEA

>p.turtle Chrysemys picta bellii

MDAFMKGLSKAKEGVVAAAEKTKQGVAEAAEKTKEGVLYVGSKTQ-GVVQGVTSVAEKTKEQASQLGGAVISGAGNIAAATGLVKKEEFPTDLK-PEEVGQEAVEEPLTDPLLEPEGENYDEPPQ-DYQEYEPEA

>xenopus Xenopus laevis

MDVLMKGFSKAKEGVVAAAEKTKQGVAEAAEKTKEGVLYMGSKTRDGVVQGVTSVAEMTKEQASQLGGAVMSGAGNIAAATGLVKKDEFPTDIK-PEEEGQEALEEPAAEPLLELEGENYEEAPQDDYQEYEPEA

>coelacanth Latimeria chalumnae

MDVLMKGLSKAKEGVVAAAEKTKQGVAEAAEKTKEGVLYVGSKTKEGV-QSVASVAEKTKEQASQLGGAVFSGAGNIAAATGLVKKDEFPTDIK-PEEVAQEAVEEPMVEPMVEPEGETYEEAPQGDYQEYEPEA
