# Supplementary material for: Did α-Synuclein and Glucocerebrosidase Coevolve? Implications for Parkinson’s Disease
Source: PLoS One. 2015 Jul 27;10(7):e0133863. doi: 10.1371/journal.pone.0133863 (PMC4516260; doi:10.1371/journal.pone.0133863)
Supplement: S2 Text — Sequence errors and the importance of the pseudocount correction. (DOCX) [file pone.0133863.s008.docx]

**Appendix I. Sequence errors and the importance of the pseudocount correction**

The correlation terms used in correlated mutation analyses often contain an additional variable, the pseudocount *λ*.[[1](#_ENREF_1),[2](#_ENREF_2)] The pseudocount can be thought of as an artificial minimum number that each amino acid occurs at each position in a multiple sequence alignment. For example, if a particular amino acid occurs *k* times at position *i* in an alignment of *s* sequences, the frequency for that amino acid to be used in the correlation term would be *c’*(*k* + *λ*)/*s*, where *c’* is a renormalization factor such that the sum of all amino acid frequencies at each position remains equal to one. The rationale for using a pseudocount variable has typically been that it helps reduce spurious statistical fluctuations due to insufficient sampling arising from the finite size of the multiple sequence alignment.[[1](#_ENREF_1)] According to this rationale, the pseudocount value should be less than one, that is, it should be equal to one divided by the factor increase in the number of sequences needed in order to see an occurrence of the rare amino acid in that position. Empirically, some analyses have found that pseudocount values greater than one produce more accurate predictions of residue contacts, however.[[3](#_ENREF_3)]

One kind of sequencing error to which MI Z-value analysis is especially vulnerable is when the error occurs for an invariant residue. Then any other residue where a mutation occurs for just that species will show a high MI Z-value with the erroneous residue. For example, in the analysis of α-syn and GCase in the main text, if no pseudocount is used (*λ* = 0), the highest MI Z-value occurs for α-syn residue 41 and GCase residue 279. Residue 41 is invariant except for the gibbon sequence, which has a Cys instead of a Gly residue. GCase residue 279 has either Met or Ile, except for gibbon which has Val and axolotl and coelacanth which have Leu.

α-syn 41 GGCGGGGGGGGGGGGGGGGGGGGGGGGGGGGGGGGG

GGGGGGGGGGGGGGGGGGGGGGGGGGGGGGGGGGGG

GCase 279 MMVMMMMMMMMMIMMMMMMMMMIIIIIIMMMMMMMM

MIIMIMMMMIIMMMIIIIIIIIIIIMIMMIIMMILL

Val or Leu for Met or Ile are conservative substitutions, so there is no obvious reason to suspect that Val279 in gibbon GCase is a sequencing error. On the other hand, no other species has Cys in the otherwise invariant position 41 of α-syn, so Cys41 in the gibbon sequence is suspicious. Furthermore, the protein sequence entry for gibbon α-syn includes “PREDICTED: LOW QUALITY PROTEIN” in its title, so Cys41 might be an error due to low quality sequencing. Using a pseudocount reduces the MI Z-value in this case; for example, using *λ* = 1 means every non-Gly, non-Cys amino acid has a frequency of ~1/72 at position 41, where 72 is the number of species. This reduces the significance of the single Cys41 occurrence, with its MI Z-value ranking falling from 1st to 10th. Using *λ* = 1.5 drops its rank dramatically, to 2813rd. In effect, by using a pseudocount of 1.5, correlated mutations that occur only in one species have a greatly reduced effect on the MI Z-value. For this study *λ* = 1.5 is used.

1. Buslje CM, Santos J, Delfino JM, Nielsen M (2009) Correction for phylogeny, small number of observations and data redundancy improves the identification of coevolving amino acid pairs using mutual information. Bioinformatics 25: 1125-1131.

2. Jeong CS, Kim D (2012) Reliable and robust detection of coevolving protein residues. Protein Eng Des Sel 25: 705-713.

3. Morcos F, Pagnani A, Lunt B, Bertolino A, Marks DS, et al. (2011) Direct-coupling analysis of residue coevolution captures native contacts across many protein families. Proc Natl Acad Sci U S A 108: E1293-1301.
