# Supplementary material for: Did α-Synuclein and Glucocerebrosidase Coevolve? Implications for Parkinson’s Disease
Source: PLoS One. 2015 Jul 27;10(7):e0133863. doi: 10.1371/journal.pone.0133863 (PMC4516260; doi:10.1371/journal.pone.0133863)
Supplement: S3 Text — Probability estimate of a chance correlation. (DOCX) [file pone.0133863.s009.docx]

**Appendix II. Probability estimate of a chance correlation**

It is possible that the [α-syn 53, GCase 115] correlation arose from uncorrelated mutations, that is, from a random mutation for α-syn 53 in a common ancestor of apes, Old World monkeys and S. boliviensis, and a subsequent GCase 115 mutation in a later common ancestor of apes and Old World monkeys. To get a rough idea of the probability involved, consider the simplified case where we have a species and 71 of its direct ancestors. Consider the situation where a mutation occurs only once in one particular ancestor, to represent the E115G mutation in the common ancestor of apes and Old World monkeys. There are 111 residue positions of GCase in the 72 species where only one mutation occurs. For simplicity, assume the mutation rate at these 111 positions is 1/72. So the probability that we get just one instance of a mutation at any of these positions in that one particular ancestor is

$$111\left[ {\frac{1}{72}\left( 1-\frac{1}{72} \right)}^{71} \right]\left[ 1-\frac{1}{72}\left( 1-\frac{1}{72} \right)^{71} \right]^{110}=0.32$$

where (1/72)(1-1/72)^71^ is the probability of just one mutation in the particular ancestor for a particular residue position, and (1-[(1/72)(1-1/72)^71^])^110^ is the probability that the same mutation pattern does not occur at any of the other 110 positions, since as mentioned in the main text, the [α-syn 53, GCase 115] pattern does not occur for any other residue pairs. Thus, there could be a roughly 1 in 3 probability that the correlation is due to chance.
